# Non-apoptotic cell death induction via sapogenin based supramolecular particles

**DOI:** 10.1038/s41598-022-17977-4

**Published:** 2022-08-16

**Authors:** Göklem Üner, Erdal Bedir, Onur Serçinoğlu, Petek Ballar Kırmızıbayrak

**Affiliations:** 1 Department of Bioengineering, Faculty of Engineering, İzmir Institute of Technology, 35430 Urla, İzmir, Turkey; 2 Department of Bioengineering, Faculty of Engineering, Gebze Technical University, 41400 Gebze, Kocaeli, Turkey; 3 Department of Biochemistry, Faculty of Pharmacy, Ege University, 35100 Bornova, İzmir, Turkey

**Keywords:** Cancer, Cell biology, Chemical biology, Drug discovery, Medicinal chemistry

## Abstract

The discovery of novel chemotherapeutics that act through different mechanisms is critical for dealing with tumor heterogeneity and therapeutic resistance. We previously reported a saponin analog (AG-08) that induces non-canonical necrotic cell death and is auspicious for cancer therapy. Here, we describe that the key element in triggering this unique cell death mechanism of AG-08 is its ability to form supramolecular particles. These self-assembled particles are internalized via a different endocytosis pathway than those previously described. Microarray analysis suggested that AG-08 supramolecular structures affect several cell signaling pathways, including unfolded protein response, immune response, and oxidative stress. Finally, through investigation of its 18 analogs, we further determined the structural features required for the formation of particulate structures and the stimulation of the unprecedented cell death mechanism of AG-08. The unique results of AG-08 indicated that supramolecular assemblies of small molecules are promising for the field of anticancer drug development, although they have widely been accepted as nuisance in drug discovery studies.

## Introduction

After the discovery of regulated necrosis, the induction of necrotic cell death has attracted huge attention, and numerous studies state that necrotic death inducers possess a high potential for cancer treatment. In a recent study, we reported a new semi-synthetic saponin analog (AG-08) triggering necrotic cell death along with unprecedented pathways, including enhancement of global proteolysis and several alterations in lysosomal function and physiology^1^. The subsequent studies revealed an unexpected property of AG-08: after one freeze–thaw cycle of its solution, AG-08 lost its cytotoxicity without any change in its chemical structure. Thus, we readily suspected the formation of self-assembly supramolecular structures. Several studies reported that small molecules could form colloidal aggregates in aqueous environments. As they non-specifically bind and inhibit enzymes, these colloidal structures are described as one of the reasons for the false-positive results in bioactivity screenings^2–4^. Interestingly, the colloidal aggregate formation has also been reported for some FDA-approved drugs such as fulvestrant, lapatinib, and sorafenib. While the colloids of these drugs non-specifically inhibit enzymes in cell-free conditions, their activities were lost during cytotoxicity screening studies. On the other hand, several small compounds of natural origin (adenine, phenylalanine, cysteine, tyrosine, adenine, uracil, and orotic acids) were stated to gain cytotoxic properties after forming amyloid-like supramolecular structures at high concentrations. These supramolecular structures are suggested to cause various metabolic disorders such as phenylketonuria^5^. Similarly, the side effects of the artificial sweetener, viz. aspartame (a small peptide), were reported to originate due to the formation of amyloid-like structures at high doses^6^.

Small molecules that form aggregates are generally excluded from further drug development studies due to the false-positive results in cell-free enzyme inhibition tests, the unwanted toxicities, and the loss of biological activities. Thus, there is huge gap in the literature regarding the physicochemical properties and biological activities of small molecule-based supramolecular assemblies. There is only a limited number of studies indicating that some of these structures may have unique properties. Their protein binding properties or incorporation of other molecules into their supramolecular structure may reflect the potential of these small molecules as being candidates for protein and drug transport^3,7^. It has also been reported that some small molecules forming aggregates may have fascinating biological activities. For instance, supramolecular structures formed by a molecule called 1543 activate caspases and induce apoptosis along with general proteolysis^8^. Similarly, a compound, which contains a naphthyl group and two phenylalanine residues, self-assembles into a nanofiber structure at a relatively high concentration (320–340 µm) and induces apoptosis via disruption of the dynamics of microtubules^9^. Nanotubes formed by betulinic acid in the mixture of ethanol and water induce reactive oxygen species (ROS) mediated apoptosis accompanied with an increase in the levels of pro-inflammatory cytokines^10^. Additionally, ursolic acid forms aggregates and induces IL-1ß expression by binding to the CD36 receptor. Interestingly, seven derivatives of ursolic acid were also determined to form aggregates, but they did not cause any change in IL-1ß synthesis^11^. These results indicate that small molecule-based aggregates have unique characters and activities.

Here, we report that a saponin analog AG-08 self-assembles into supramolecular structures responsible for its intriguing cell death-inducing activity. After internalization via non-canonical endocytosis, AG-08 supramolecular particles mainly alter the expression of genes involved in Unfolded Protein Response (UPR), heat stress, and immune response. Moreover, to see the initial structure–activity relationships (SAR), we prepared 18 analogs, four of which exhibited similar bioactivity profiles as in AG-08. Collectively, our results indicate that both the biophysical properties and the unique bioactivity through a distinct cell death pathway make AG-08 an exciting molecule for future research. In a general perspective, this study has provided new evidence that small molecule-based supramolecular assemblies should not be neglected with prejudgment as they may hold great opportunities for drug development studies.

## Results

### AG-08 forms self-assembly particles

**Figure 1:**
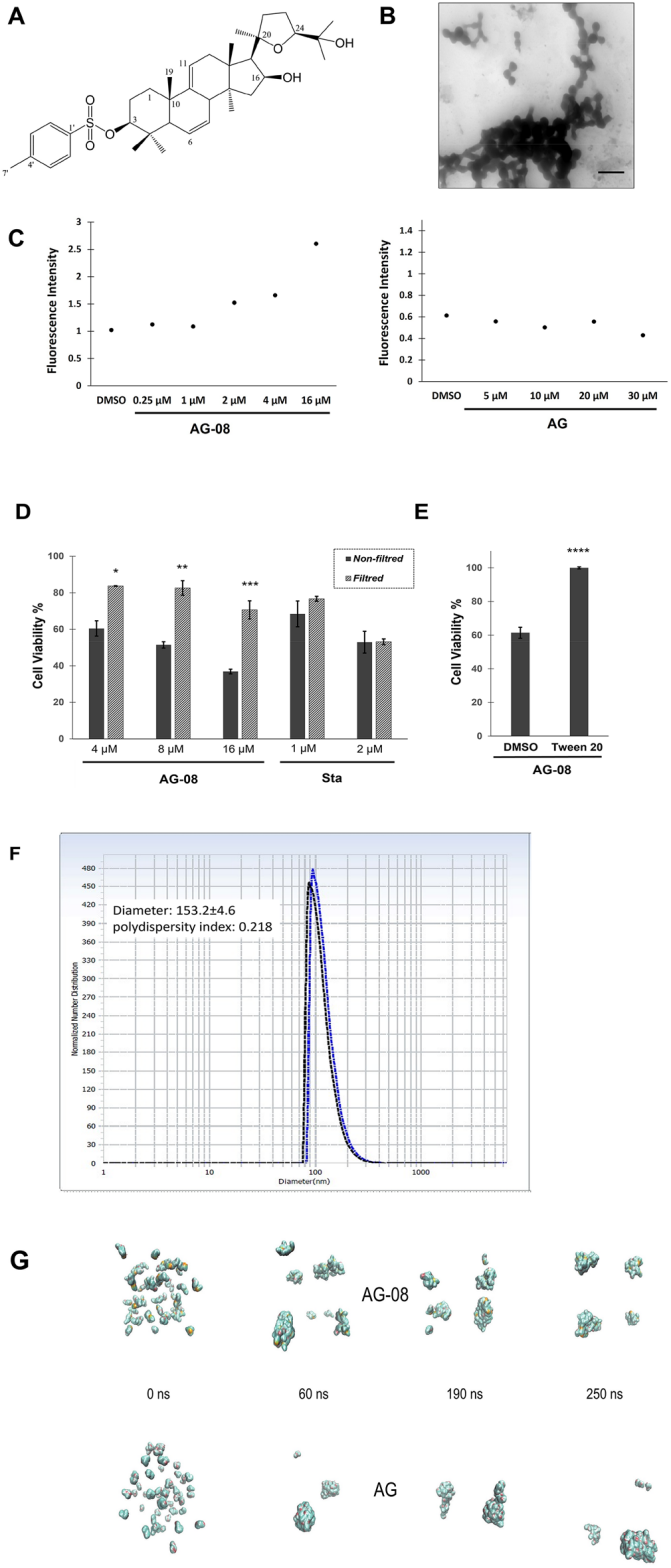
AG-08 mediated cytotoxicity relies on the supramolecular structures. (**A**) Structure of AG-08. (**B**) The scanning transmission electron micrograph of 16 µM AG-08 in PBS. Figure is a representative image of stained sample with uranyl acetate (5683 × magnification; scale bar 600 nm). (**C**) After 3 h incubation of Nile red solution with AG-08 or AG, fluorescence intensity of Nile red was measured. (**D**) AG-08, Staurosporine (Sta) or vehicle samples prepared in the cell condition medium was filtrated by 220 nm filter and HCC1937 cells were exposed to the filtrates. After 24 h, the viability of cells was measured and represented as a percentage of that of the vehicle control. The error bars represent mean value ± s.d. (n = 3). *p < 0.05, **p < 0.005, ***p < 0.001. (**E**) Tween 20 (1:40,000) was added to the fresh medium before addition of 8 μM AG-08 or vehicle. Then cell medium was replaced with this fresh medium. Cell viability was detected after 24 h incubation. Error bars are the standard deviations (n = 3; ****p < 0.0001) (**F**) Hydrodynamic diameter of AG-08 based supramolecular structures (**G**) AG and AG-08 form aggregates at 60 nM concentration in MD simulations.

In our previous studies, we discovered that AG-08 (Fig. 1A) induced necrosis via enhanced global proteolysis involving calpains, cathepsins, and caspases. Strikingly, the cytotoxic activity of AG-08 was found to be lost after even one freeze–thaw cycle without any change in its chemical structure. Since the freeze–thaw cycle was reported to affect molecular clusters^12^, we suspected the formation of supramolecular structures. Therefore, to ascertain that AG-08 formed particles in neutral buffer/medium, we performed a STEM microscopy analysis and revealed that nano-sized circular particles were merged to form micro-sized supramolecular structures (Fig. 1B). Diameter of AG-08 based supramolecular structures were detected as 153.2 ± 4.6 (Fig. 1F). Next, the minimum concentration of AG-08 required for the particle formation was determined by using Nile red staining. Nile red is widely used to monitor supramolecular structures such as micelles or aggregates as it is nearly non-fluorescent in aqueous conditions but tends to associate with hydrophobic domain within the assemblies and emits intense fluorescence^13,14^. The critical assembly concentration of AG-08 was found to be approximately 2 µM (Fig. 1C). Interestingly, when the particle formation of the parent compound (AG) was investigated via Nile red, no fluorescence intensity even at 30 µM was observed (Fig. 1C). This finding indicates that AG was not capable of forming ordinary supramolecular structures interacting with Nile red.

To verify that the activity of AG-08 arises from its supramolecular structures, freshly prepared AG-08 solutions were filtered using 220 nm-filters to remove AG-08 supramolecular particles from medium, and the filtrates were analyzed for their cytotoxicity. While filtrates of staurosporine solution, a well-known apoptosis inducer, still effectively caused cell death, the filtered AG-08 sample no longer exhibited any cytotoxicity (Fig. 1D). Next, Tween-20 was utilized as a non-ionic detergent to block particle formation, and AG-08-mediated cell death was inhibited as expected (Fig. 1E). Collectively, our findings proved that AG-08 but not the parent compound, forms self-assembly particles, and those particles are responsible for the previously reported distinct cell death signaling.

### AG-08 shows a higher propensity to self-aggregate in molecular dynamics simulations

Next, we tested whether the observed difference in terms of the aggregation propensity of AG and AG-08 may be confirmed from a theoretical perspective as well. To this end, we used molecular dynamics (MD) simulations that have been successfully used to distinguish aggregators from non-aggregators^15^. The principle underlying this method is that when randomly placed in a simulation box containing solvent molecules, the self-aggregator will begin to make non-bonded contacts with copies of itself instead of the surrounding waters, while the non-aggregator will remain dispersed throughout the simulation box. Given the fact that the aggregation behavior of small molecules is also highly concentration-dependent, the number of molecules, as well as the size of the simulation box, should be carefully chosen to mimic experimentally obtained critical assembly concentrations. However, it is not always possible to test desired concentration ranges with MD simulation, as lower concentrations require longer simulation times (and hence higher computational capacity) to allow copies of molecules to clash into each other^16^. The systems we could afford to construct for AG and AG-08 thus included concentrations significantly higher than the critical assembly concentration ranges observed (Table 1). So, although the results do not be easily compared to experimental observations, it is still conceivable that an analysis of intermolecular contacts, even at these high concentrations, may yield useful insight into the self-aggregation behavior differences between the molecules.

**Table 1:** Molecular systems used in MD simulations and the number of each contact type observed at 250 ns of simulation time.

	AG	AG-08
Number of molecules	59	55
Concentration	~ 60 mM	~ 60 mM
Water molecules	41,704	41,388
Box size	1331 nm^3^	1331 nm^3^
Contact type* (at 250 ns)		
vdw_clash	20	207
vdw	38	86
Proximal	4499	6516
hbond	3	31
w_hbond	125	119
Aromatic	0	98
Hydrophobic	991	1627
Polar	24	108
w_polar	95	161
Total	4557	6809

MD simulations were allowed to run to cover 250 ns of simulation time for each system. Our results indicated that both molecules could self-assemble at the studied concentrations and formed aggregates already at 5 ns of simulation time. As time progressed, the aggregates were observed to grow larger but never fully reached a single large aggregate (Fig. 1G). Accordingly, AG could start to form aggregates at 60 nM. Despite the similarity in aggregation behavior of AG and AG-08 in MD simulations, an analysis of intermolecular contacts indicated that the aggregates of AG-08 may be more tightly connected to each other than those of AG. This is evident from the non-bonded interaction numbers within the formed aggregates, which were computed using Arpeggio based on the aggregate structures obtained at 250 ns^17^. Accordingly, a higher number of non-bonded interactions were observed between copies of AG-08, including Van der Waals interactions, hydrogen bonds, as well as hydrophobic and polar interactions (Table 1).

### Interrelationship between AG-08 particles and endocytosis

**Figure 2:**
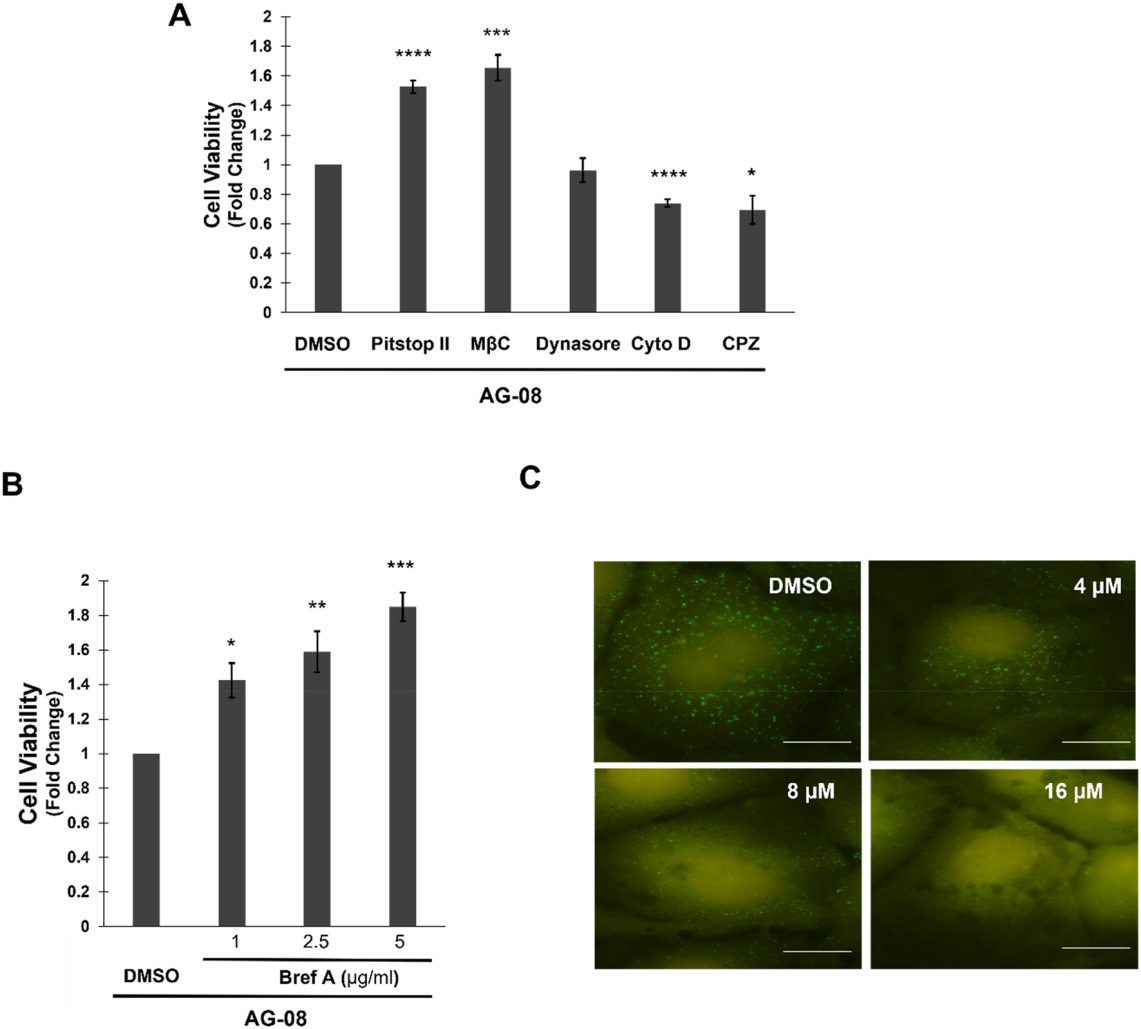
AG-08 is related with endosomal pathway. (**A**) After pre-treatment with 20 µM pitstop II, 0.5 mM methyl-β-cyclodextrin (MβC), 80 µM dynasore, 20 µM cytochalasin d (Cyto D) or 20 µM chlorpromazine (CPZ) for 1 h, HCC1937 cells were treated with 8 µM AG-08 for 24 h. Then cell viability was determined by using WST-1. Reported values were normalized on cells treated with only AG-08. Error bars are the standard deviations (n = 3). p-values were calculated with respect to AG-08 treated cells by two-tailed equal variance Student’s t-test (*p < 0.05, ***p < 0.001, ****p < 0.0001) (**B**) HCC1937 cells were treated with brefeldin A (Bref A) for 1 h and then treated with 8 µM AG-08 for 24 h. Error bars represent standard deviations (n = 3). p-values were calculated with respect to AG-08-treated cells (*p < 0.05, **p < 0.005, ***p < 0.001). (**C**) Following AG-08 treatment for 16 h, EEA1 proteins were stained using the anti-EEA1 antibody in HCC1937 cells (scale bar = 25 µm).

Next, the internalization of AG-08 via endocytosis, which is a transport process responsible for the cellular uptake of particles, was assessed by utilizing several endocytosis inhibitors. Our results revealed that methyl β-cyclodextrin, targeting cholesterol-related endocytosis through cholesterol depletion^18^, and pitstop II, an inhibitor of both clathrin-dependent and independent endocytosis^19,20^, effectively protected cells against AG-08 mediated cell death (Fig. 2A). Intriguingly, dynasore, a dynamin inhibitor^21^, cytochalasin D, an inhibitor of actin polymerization responsible for membrane ruffling^22^, and chlorpromazine, a specific inhibitor of clathrin-dependent endocytosis^23^ that prevents coated pit assembly at the cell surface, failed to rescue cells against AG-08-mediated cell death (Fig. 2A). Notably, the protection of cells from AG-08-mediated cell death by methyl β-cyclodextrin and pitstop II was found to be dose-dependent (Fig. S1A–E). These results suggest that AG-08 utilizes clathrin, dynamin, and actin-independent but cholesterol dependent non-canonical endocytosis pathway. In addition, Brefeldin A, an inhibitor of vesicular trafficking, reduced AG-08 dependent cell death, suggesting AG-08 particles should be transported via vesicles in cells (Fig. 2B).

To further understand the interrelationship between AG-08 and endocytosis, EAA1 (early endosome antigen 1), an early endosome marker, was stained in control and AG-08-treated cells. A dramatic decrease in EEA1 positive vesicles was observed by AG-08 treatment (Fig. 2C and S1F).

### General response to AG-08 particles

**Figure 3:**
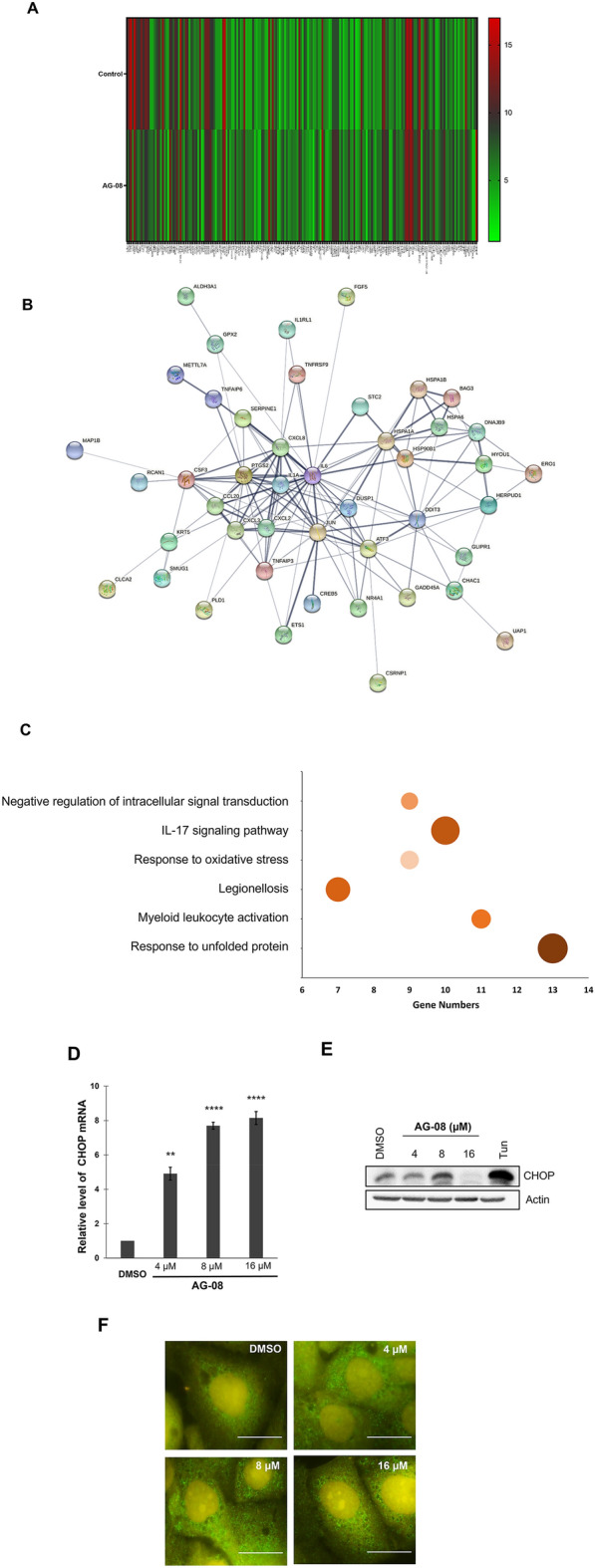
mRNA microarray analysis was used to identify differentially expressed genes via AG-08 treatment. (**A**) Heatmap representing the expressions of 193 genes with significant different expression in AG-08 treated cells compared with control. Heatmap was generated by GraphPad Prism version 8 (https://www.graphpad.com/scientific-software/prism/). (**B**) Protein–protein network analysis was formed via STRING. The thickness of the connecting lines indicates the strength of data support for physical and/or functional associations. (**C**) Pathway enrichment was determined using GO, KEGG, and Reactome pathway analysis. The figure displays six terms with the smallest p-values. Size of circle represents the significance of enrichment. (**D**) The protein levels of CHOP were determined via IB. 0.5 µg/ml tunicamycin (Tun) was used as positive control. The experiments were repeated three times independently; with one representative result shown. (**E**) mRNA level of the CHOP was investigated using quantitative PCR (q-RTPCR). Error bars represent standard error (s.e.). p-values were calculated with respect to vehicle-treated cells by one-way Anova test. (**p < 0.005, ****p < 0.0001). (**F**) The expression and localization of Calnexin protein were evaluated using immunofluorescence via anti-Calnexin antibody (scale bar 25 µm).

To investigate the overall cellular response to AG-08 treatment, microarray gene expression analysis of cells treated with vehicle and AG-08 treated groups was performed. The genes with a fold change were ≥ 3.0, and p-value ≤ 0.05 (unpaired t-test) were considered significantly regulated via AG-08 treatment. The expression of 193 genes (78 were upregulated and 115 were downregulated) was significantly affected by AG-08 treatment (Fig. 3A, Table S1). Next, protein–protein interaction (PPI) network analysis was evaluated by the STRING (Search Tool for the Retrieval of Interacting Genes/Proteins) network. The constructed PPI network is shown in Fig. 3B. There were 61 nodes and 145 edges in the network (PPI enrichment p-value < 1 × 10^–16^). The number of interactions was significantly higher than expected for a random set of proteins. Then, enrichment was performed using GO and KEGG pathway analyses. Selected six GO and KEGG terms with the smallest p-values were shown in Fig. 3C. The results of enrichment analysis revealed a noticeable variation in the expression of proteins in pathways associated with unfolded proteins response, immune system, and oxidative response. Consistently, Reactome analysis also showed significant enrichment in heat stress-related pathways.

### AG-08 particles induce UPR

Microarray results showed that one of the pathways induced by AG-08 supramolecular particles is Unfolded Protein Response (UPR) which is known to be activated after prolonged endoplasmic reticulum (ER) stress^24^. Thus, we aimed to investigate the induction of ER stress upon AG-08 treatment. Consistent with microarray results, our data verified that CHOP (DDIT3), a known ER stress marker, was upregulated in a dose-dependent manner in AG-08 treated cells (Fig. 3D) (Table 2). Interestingly, CHOP protein level was enhanced by 8 µM AG-08 treatment but surprisingly decreased by 16 µM (Fig. 3E). Since similar results were observed in the p62 protein levels in the previous study^1^, we suggested that the CHOP levels were induced due to ER stress, but the enhancement of global proteolysis by the prolonged or higher dose treatments of AG-08 might lead to degradation of full-length CHOP protein. Concomitantly, the expression level of calnexin, a chaperone localized in the ER upregulated during ER stress^25^ was analyzed, and the intensity of calnexin staining was found to be significantly increased in AG-08 treated cells (Fig. 3F). Collectively, all these data confirmed that AG-08 particles induce ER stress.

**Table 2:** Upregulation of UPR genes with AG-08 treatment.

Genes	Fold changes
CXCL8	9.38
DDIT3 (CHOP)	7.57
DAW1	5.06
HSP90B1	4.89
DNAJB9	4.59
HERPUD1	4.06
ATF3	3.61
HYOU1	3.14

### Synthesis of analogs

**Figure 4:**
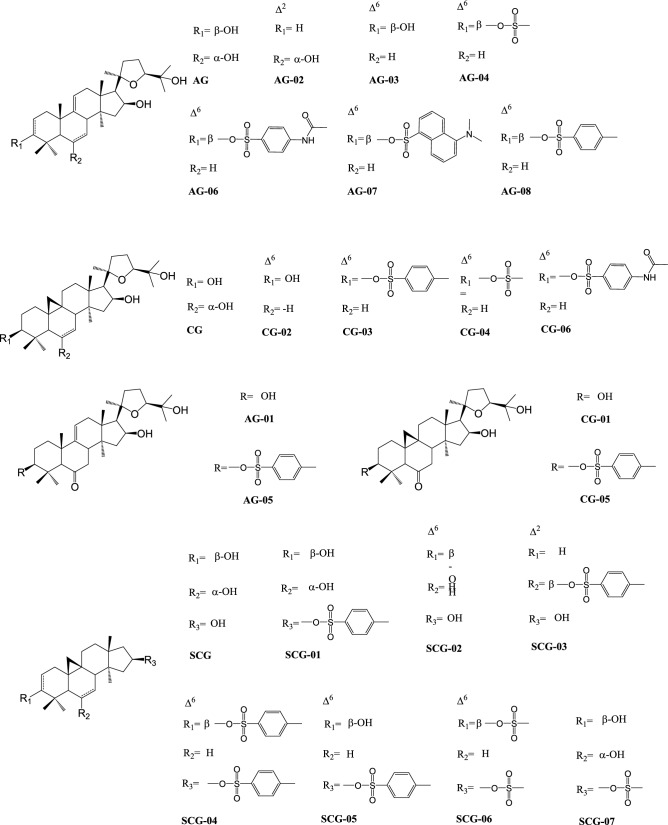
Structures of the AG-08 derivatives.

We next aimed to further characterize the relation between the chemical structure of AG-08 and its capacity of supramolecular assembly formation together with its biological activities by preparing further derivatives. In this regard, 18 analogs (Fig. 4) were synthesized from 20(27)-octanor-cycloastragenol (SCG), cycloastragenol (CG), and astragenol (AG) by the methods shown in the method section. The details and characterization of which are shown in Supplementary Information. The cytotoxic activities of the semi-synthetic analogs were evaluated against four human cancer cell lines, namely HeLa, HCC1937, MCF7, and A549 as well as against MRC-5 as a normal cell line. Molecules with IC_50_ values less than 20 µM were considered as potent cytotoxic molecules and included in subsequent studies. The compounds other than CG-03, CG-06, AG-05, and CG-05 did not exhibit promising cytotoxicity (IC_50_ > 20 µM) (Table 3). While AG-05 (between 1.55 and 5.9 µM) and CG-05 (between 1.85 and 6.1 µM) showed relatively higher cytotoxic activity, the potency of AG-06 (between 5.7 and 19.07 µM) and CG-03 (between 3.65 and 12.87 µM) were notably lower than AG-08.

**Table 3:** IC_50_ values (µM) of the analogs and their parent molecules.

	HeLa	HCC1937	MRC-5	MCF7	A549
AG-01	> 30	> 30	> 30	> 30	> 30
AG-02	42.6	46.08	39.9	32.1	30.5
AG-03	33.42	20.31	24.4	21.76	25.53
AG-04	> 50	> 50	29.55	22.05	27.5
AG-05	5.9	4.28	1.55	2.55	2.02
AG-06	10.81	10.02	19.07	9.34	5.7
AG-07	34.7	38.3	7.4	ND	38.2
AG-08	5.7	3.81^1^	2.58	3.78	2.3^1^
CG-01	> 30	> 30	> 30	> 30	> 30
CG-02	43.94	32.48	33.37	21.2	21.08
CG-03	12.87	6.13	5.29	5.5	3.65
CG-04	> 50	> 50	> 50	> 50	> 50
CG-05	6.1	2.66	2.58	3.85	1.85
CG-06	> 50	38.1	> 50	41.3	> 50
SCG-01	> 50	> 50	> 50	> 50	> 50
SCG-02	> 50	> 50	> 50	> 50	> 50
SCG-03	> 50	> 50	> 50	> 50	> 50
SCG-04	> 50	> 50	> 50	> 50	> 50
SCG-05	> 50	> 50	> 50	> 50	> 50
SCG-06	> 50	> 50	> 50	> 50	> 50
SCG-07	> 50	> 50	> 50	> 50	> 50
AG	> 30	> 30	> 30	> 30	> 30
CG	> 50	> 50	> 50	> 50	> 50
SCG	> 50	> 50	> 50	> 50	> 50

*ND* not determined.

**Figure 5:**
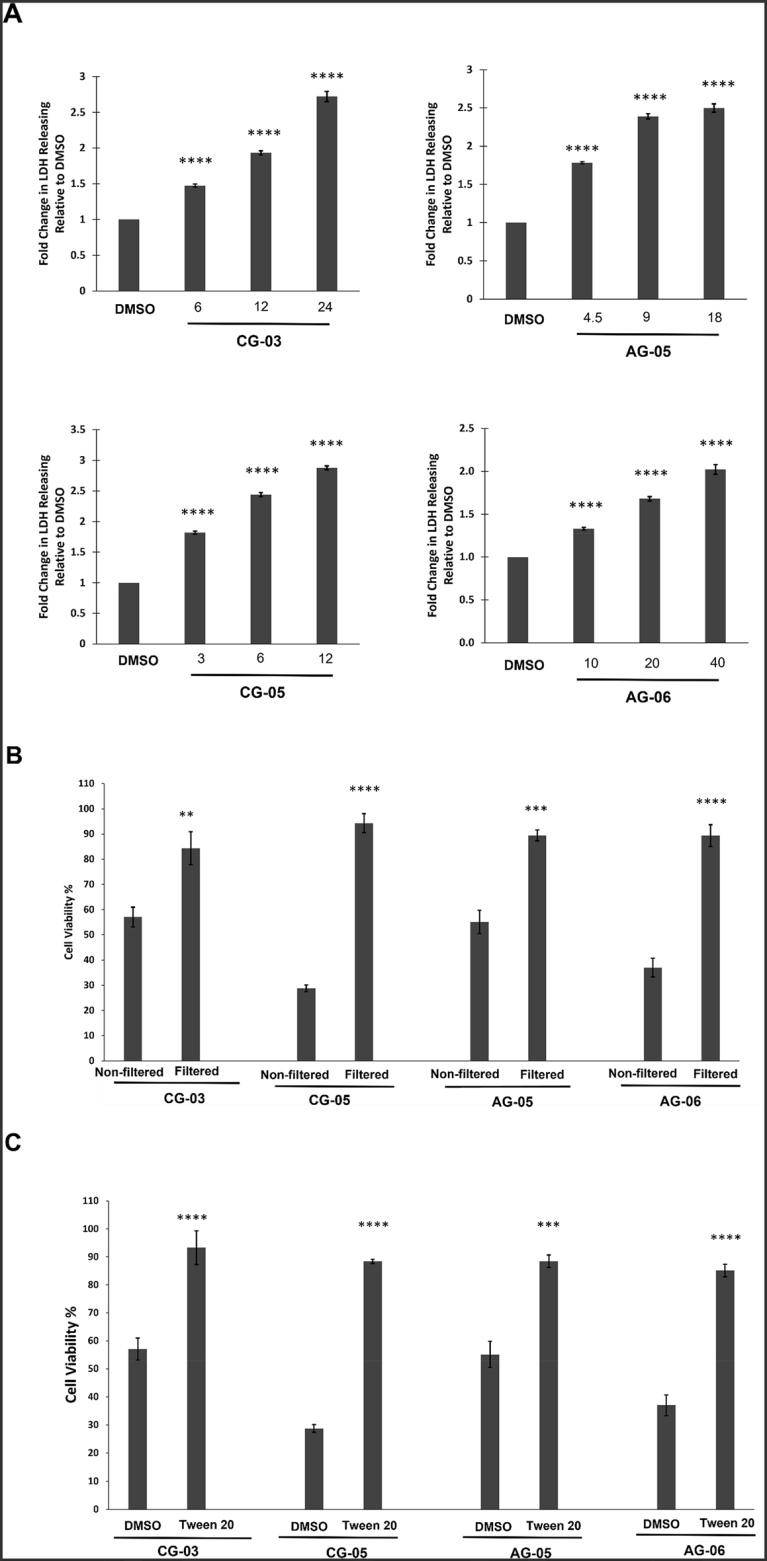
The cytotoxic activity of analogs depends on particle formation like AG-08. (**A**) LDH release was determined following the treatment of HCC1937 cells with 1-, 2- and 4-fold of IC_50_ values of cytotoxic compounds or vehicle (DMSO) for 16 h. The level of released LDH was calculated as fold change relative to vehicle. (**B**) Compounds (2-fold of IC_50_ values; 12 µM CG-03, 6 µM CG-05, 9 µM AG-05 and 20 µM AG-06) or vehicle solutions prepared in cell condition medium was filtered by 0.22 µm filter and replaced with normal medium. After 24 h, cell viability was measured. (**C**) The cell medium was replaced with fresh medium containing Tween 20 (1:40,000) and compounds (2-fold of IC_50_ values; 12 µM CG-03, 6 µM CG-05, 9 µM AG-05, and 20 µM AG-06) or vehicle. Cell viability after 24 h was measured. The reported values correspond to mean values ± standard deviation (± s.d) of three experiments. ***p < 0.001, ****p < 0.0001.

Next, the Lactate Dehydrogenase (LDH) release assay, which was used previously to demonstrate necrotic cell death for AG-08^1^ was performed, and as shown in Fig. 5A, all the tested compounds caused substantial release of LDH in a dose-dependent manner. After LDH assay, immunoblotting was used to determine whether bioactive AG-08 analogs alter the expression of proteins known as autophagy and cell death markers in a similar manner to AG-08. Indeed, it was found that the effects of these compounds on the protein profiles of LC3, Atg-7, and caspase 3 were similar to those of AG-08 (Fig. S2). Consequently, our results showed that AG-08, CG-03, CG-06, AG-05, and CG-05 induced necrotic cell death through a similar mechanism of action.

Lastly, we aimed to evaluate whether the cytotoxicity of novel analogs also is also dependent on particle formation. As expected, filtration of samples via 220 nm filters and utilizing Tween-20 to block particle formation disrupted the cytotoxic activity of all the analogs (Fig. 5B,C). Thus, the results substantiate that the new analogs, viz. CG-03, CG-06, AG-05, and CG-05, also form supramolecular assemblies that are responsible for their actions.

## Discussion

It has long been known that small molecules can form self-assembly higher-order structures via non-covalent associations. However, when detected, they are mostly excluded from drug development studies since self-assembly structures are reported as a cause of false-positive results in enzyme inhibition assays. Moreover, their unwanted toxicities and the loss of existing biological activity reinforce their recognition as nuisance for drug discovery. On the contrary, several reports have also suggested that these structures may provide unique biological activities, independent of their soluble forms; thereof, they have the potential to form novel drug development platforms. We have previously reported that AG-08, a semi-synthetic sapogenin, has a unique mechanism of activity, including autophagy/lysosome impairment and enhancement of proteolysis leading to necrotic cell death. In this recent study, we characterized the supramolecular structure of AG-08 and revealed that these structures, not its soluble form, were responsible for its biological activity. Furthermore, we identified that AG-08 supramolecular particles were internalized via non-canonical endocytosis depending on cholesterol, but not actin and dynamin. The internalization pattern of AG-08 is not typical because most of the characterized endocytosis pathways require dynamin and/or actin. On the other hand, similar to AG-08, some viruses like LCMV have been reported to enter cells using a non-canonical pathway independent of key endocytosis elements such as clathrin, caveolin, flotillin, Arf6, dynamin-2, and actin^26^ but dependent on cholesterol^27^. AG-08 treatment also diminished the number of EEA1 positive vesicles indicating the inhibition of EEA1 recruitment to the early endosomes. Given the inhibition of early endosome function and the role of EEA1 in tethering and/or docking of early endosome, AG-08 might be inhibiting EEA1 recruitment following its internalization through the early endosome or might bypass early endosomes and go directly to late endosomes, as reported for LCMV^27^. In line with that, the protection of cells against AG-08-mediated cell death by the inhibitor of intracellular vesicular trafficking, brefeldin A, further suggests that AG-08 particles are transported via vesicles.

The microarray analysis and further validation studies have revealed that AG-08 supramolecular particles induced a strong UPR. In our previous study, we reported that AG-08 triggers the activation of calpains and caspases as well as an increase in the activity of cathepsins in the cytoplasm. Both induction of UPR and protease activity by AG-08 supramolecular particles are highly resembling those induced by protein aggregates. For instance, the activation of calpains and caspases was reported as one of the cellular changes in Huntington’s disease, where proteases are possibly activated to cope with the aggregates, although they cleave the huntingtin aggregates into more toxic smaller fragments^28–30^. Hence, based on the above-mentioned results, it can be suggested that the proteases activated by AG-08 particles contribute to cell death through the cleavage of essential cellular components, and enhanced proteasomal activity is a similar feature of protein aggregates and AG-08 particles.

As the interpatient variability along with the inter-and intra-tumoral heterogeneity limits the success of cancer treatments, the discovery of different treatment approaches and/or alternative drugs such as small molecule-based anticancer drugs are highly critical, especially in expanding the current chemotherapeutic repertoire and increasing options for combination therapies. The results of AG-08 signify once again that supramolecular structures formed by small molecules may have paved the way for their use for anticancer therapy. Moreover, the induction of necrotic cell death via AG-08 supramolecular particles is also intriguing for cancer research, as non-apoptotic cell death has attracted great attention to cope with the resistance to apoptotic cell death, one of the main problems of current chemotherapeutic agents. The necrotic cell death is highly specified for intra-tumoral therapy, as it can activate antitumor immune response. Strikingly, PV-10, a necrosis triggering agent that was designated as an orphan drug in neuroblastoma, melanoma, and hepatocellular carcinoma, has undergone clinical trials for the treatment of different cancers and has been reported to cause a reduction in both injected and untreated tumors and metastasis sites^31,32^. Tigilanol tiglate (EBC-46), which is a diterpenoid with necrotic properties, has also recently been approved by the FDA-CVM for veterinary use for intra-tumoral therapy. Furthermore, positive results have been reported from phase I studies of EBC-46 in patients with accessible cutaneous, subcutaneous, or nodal tumors refractory to conventional therapy^33^. Consistent with the signature of necrotic cell death, our microarray results verified that the immune system-related genes were significantly altered by AG-08 treatment. Therefore, we speculate that AG-08 particles have a high potential for the treatment of cancer via intra-tumoral injection.

Our computer-based study has shown that AG-08 could form much stronger non-covalent bonds than AG, the parent compound. In parallel, AG-08 formed supramolecular structures that encapsulated Nile red dye while AG failed to do so. Additionally, 18 analogs prepared from AG, CG, and SCG allowed us to understand the role of residues in the formation of these particles accounting for the biological activity. Among them, CG-03, AG-05, AG-06, and CG-05 were identified as cytotoxic compounds that formed supramolecular particles and induced necrotic cell death. The results also facilitated us to deduce the structure − activity relationships: Firstly, the importance of the existence of a tosyl group at C-3 was evaluated: (i) when the tosyl group was replaced with non-aromatic mesylate group (AG-04 and CG-04), the activity was lost; (ii) AG-07 was prepared by addition of a bulky naphthalene sulfonyl chloride (dansyl chloride) to AG, which again caused a loss of activity; iii) N-Acetylsulfanilyl substitution led to a decrease in activity with AG skeleton (AG-06) while resulting in complete activity loss in CG analog (CG-06). Thus, it is concluded that a specific sulfonyl ester (tosylate) is required for bioactivity towards cancer cells. The double bond at C-6, forming readily during tosylation reaction of AG, CG, and SCG, was dispensable because the compounds with C-6 double bond and no tosyl group at C-3 did not exhibit antiproliferative effect (AG-02 and CG-02). On the other hand, the presence of a keto functionality at C-6 afforded higher activity in CG and AG analogs, viz. CG-05 and AG-05. Secondly, we prepared similar SCG analogs, missing the side chain extending from C-17 position. The SCG analogs showed no cytotoxicity suggesting that tosyl substitution by itself was not sufficient for activity alone, and intact sapogenin skeleton was required, including 20,24-epoxy side chain. All these findings imply that certain chemical features must be met to lead the sapogenin skeleton to form stable supramolecular particles and to show antiproliferative effects.From a particle chemistry and point of view, it is worth noting that some of the known biologically active sapogenins may act as supramolecular structures rather than their soluble forms. At relatively high concentrations (> 50 mM), betulinic acid forms fibrils inducing apoptosis, while ursolic acid forms aggregates at lower concentrations (4–20 mM) and trigger IL-1beta release without cell death. Here, we report a unique sapogenin (AG-08) and its derivatives that are also able to form supramolecular particles. However, the mechanism put forward makes our derivatives exceptional compared to widely encountered and well-known sapogenin aggregators including betulinic acid, oleanolic acid, and ursolic acid. On the other hand, these sapogenin based particles may cause systemic toxic effects restricting their exploitation that should be considered carefully in drug discovery efforts demanding targeted delivery or local administration.

## Conclusion

The work described here reveals that AG-08 and four of its analogs form particles that trigger necrotic cell death with an unprecedented mechanism and indicates that the small compound-based supramolecular structures have the capability to activate/suppress different cellular signals in the cell. Therefore, we demonstrated that distinct compounds could form unique supramolecular structures at low concentrations and have potential to become drug candidates with unique activities. We expect AG-08 and its analogs to attract attention to small molecule based supramolecular structures in drug development studies.

## Methods

### Synthesis

20(27)-octanor-cycloastragenol (SCG), CG, CG-01 and AG-01 was donated by Bionorm Natural Products (Turkey). AG was synthesized from cycloastragenol via performing a previously reported procedure^1^.

### Reaction of AG with p-TsCl

100 mg AG was dissolved in pyridine and 450 mg *p*-TsCl (p-Tosyl Chloride, Acros Organics) reagent was added. Reaction was continued for 6 h at room temperature and quenched by addition of distilled water. Following the extraction via 500 ml ethyl acetate (3 times), the extract was evaporated at 60 °C in the rotary evaporator. Crude product was purified on a silica gel chromatography column (cyclohexane:EtOAc; 7:3) to give **AG-02** (3.8 mg lyophilized white-off powder), **AG-03** (6.5 mg lyophilized white-off powder) and **AG-08** (25 mg lyophilized white-off powder)^1^. For **AG-02**; ^1^H NMR (400 MHz, CDCl_3_): δ = 5.5 (ddd J = 10.1, 5.4, 3 Hz, 1H), 5.31 (m, 1H), 5.3 (m, 1H) , 4.72 (ddd, J = 7.9, 7.9, 6.3 Hz, 1 H), 4.06 (ddd, J = 10.8, 10.8, 3.9 Hz, 1H), 3.75 (dd, J = 8.1, 6.2 Hz, 1H), 2.59 (q, J = 10.4 Hz, 1H), 2.4 (m, 1H), 2.36 (d, J = 7.8, 1H), 2.14 (m, 1H), 2.11 (m, 1H), 2.05 (m, 1H), 2.04 (m, 1H), 2 (m, 2H), 1.89 (m, 1H), 1.86 (m, 1H), 1.6 (m, 1H), 1.51 (dd, J = 12.8, 6.2, 1H), 1.44 (m, 1H), 1.3 (s, 3H), 1.28 (m, 1H), 1.23 (s, 3H), 1.2 (s, 3H), 1.17 (s, 3H), 1.14 (s, 3H), 1.05 (s, 3H), 0.94 (s, 3H), 0.79 (s, 3H). ^13^C NMR (100 MHz, CDCl_3_): δ = 145.8, 139.6, 120.3, 116.3, 87.2, 81.5, 73.40, 72.0, 70.2, 56.2, 55.4, 45.1, 44.3, 43.9, 40.9, 40.3, 38.6, 37.9, 37.7, 36.0, 34.9, 34.6, 29.8, 28.0, 27.8, 26.7, 25.9, 23.7, 19.2, 18.1. HRMS (ESI): *m/z* 495.34667 [M + Na]^+^ (C_30_H_48_O_4_Na, calcd. 495.34503). For **AG-03**; ^1^H NMR (400 MHz, CDCl_3_): 5.71 (m, 1H), 5.57 (dt, J = 10.2, 3.2 Hz, 1H), 5.16 (brs, 1H), 4.69 (m, 1H), 3.75 (t, J = 7.2 Hz, 1H), 3.23 (dd, J = 11.8, 4.8 Hz, 1H), 2.84 (brs, 1H), 2.56 (m, 1H), 2.24 (dd, J = 11.8, 6.1 Hz 1H), 2.07 (m, 1H), 2.04 (m, 1H), 2.0 (m, 2H), 1.92 (m, 1H), 1.78 (m, 2H), 1.70 (d J = 12.2, 1H), 1.59 (m, 2H), 1.58 (m, 1H), 1.57 (m, 1H), 1.29 (s, 3H), 1.23 (s, 3H), 1.14 (s, 3H), 1.01 (s, 6H), 0.98 (s, 3H), 0.83 (s, 3H), 0.64 (s, 3H). ^13^C NMR (100 MHz, CDCl_3_) δ = 145.9, 128.9, 127.3, 113.6, 87.3, 81.8, 79.3, 73.6, 72.2, 56.6, 52.2, 44.8, 44.3, 43.9, 43.8, 39.2, 38.8, 38.2, 34.8, 34.4, 28.2, 28.2, 28.2, 28.2, 26.9, 26.1, 20.4, 19.1, 18.9, 16.0. HRMS (ESI): *m/z* 495.34894 [M + Na]^+^ (C_30_H_48_O_4_Na, calcd. 495.34503).

### Reaction of AG with MsCl

MsCl (140 µL) and TEA (150 µL) was added to a solution of AG (150 mg) in dichloromethane. The reaction mixture was stirred at 0 °C for 3 h. The reaction solvent was then removed under reduced pressure, and the residue was purified using silica gel column chromatography (*n*-hexane:EtOAc; 75:25) to give **AG-04** (10.5 mg lyophilized white-off powder). ^1^H NMR (400 MHz, CDCl_3_): δ = 5.67 (dt, J = 10.1, 2.1 Hz, 1H), 5.63 (m, 1H), 5.17 (dt, J = 5.3, 2.5 Hz, 1H), 4.71 (ddd J = 7.7, 7.7, 5.9 Hz, 1H), 4.35 (dd, J = 1.6, 4.9 Hz, 1H), 3.76 (dd, J = 7.2, 7.2 Hz, 1H), 3.03 (s, 3H), 2.85 (brs, 1H), 2.57 (q, J = 10.6 Hz, 1H), 2.24 (d, J = 7.6 Hz, 1H), 2.2 (m, 1H), 2.08 (m, 1H), 2.06 (m, 1H), 2.05 (m, 1H), 2 (m, 2H), 1.96 (m, 1H), 1.81 (brs, 1H), 1.65 (m, 2H), 1.64 (m, 1H), 1.57 (m, 1H), 1.3 (s, 3H), 1.24 (s, 3H), 1.15 (s, 3H), (1.05 s, 6H), 0.98 (s, 3H), 0.92 (s, 3H), 0.67 (s, 3H). ^13^C NMR (100 MHz, CDCl_3_): δ = 144.8, 129.42, 126.1, 113.9, 90.2, 87.0, 81.6, 73.3, 72.0, 56.4, 52.2, 44.6, 44.0, 43.7, 43.5, 39.0, 38.6, 38.3, 37.9, 34.6, 33.9, 28.1, 27.9, 27.8, 26.7, 25.8, 25.7, 20.1, 18.8, 18.7, 16.5. HRMS (ESI): *m/z* 573.32873 [M + Na]^+^ (C_31_H_50_O_6_SNa, calcd. 573.32258).

### Reaction of AG-01 with p-TsCl

To a stirring solution of AG-01 (30 mg, in pyridine), *p*-TsCl solution (200 mg) in pyridine was added. The reaction mixture was stirred at room temperature for 12 h. Reaction mixture quenched with water and extracted three times with 100 mL EtOAc. Crude product was purified by silica gel column chromatography (cyclohexane:EtOAc; 8:2) to give **AG-05** (13.9 mg lyophilized white-off powder). ^1^H NMR (400 MHz, CDCl_3_): δ = 7.77 (dd, J = 8.2, 1.7 Hz, 2H), 7.32 (dd, J = 8.2, 1.7 Hz, 2H), 5.44 (dd, J = 6.3, 1.7 Hz, 1H), 4.68 (q, J = 6.8 Hz, 1H), 4.07 (m, 1H), 3.75 (td, J = 7.4, 1.6 Hz, 1H), 2.69 (brs, 1H), 2.56 (q, J = 10.4 Hz, 1H), 2.43 (s, 3H), 2.34 (d, J = 5 Hz, 1H), 2.32 (brs, 1H), 2.19 (m, 1H), 2.17 (brs, 1H), 2.15 (m, 1H), 1.96 (m, 1H), 1.95 (m, 1H), 1.91 (m, 2H), 1.88 (d, J = 4.24 Hz, 1H), 1.85 (d, J = 1.5 Hz, 1H), 1.68 (m, 1H), 1.56 (dt, J = 12.6 Hz, 1H), 1.43 (dd, J = 12.9, 6.3 Hz, 1H), 1.29 (s, 3H), 1.27 (d, J = 1.3 Hz, 1H), 1.22 (s, 3H), 1.14 (s, 3H), 1.04 (s, 3H), 0.90 (s, 3H), 0.84 (s, 6H). ^13^C NMR (100 MHz, _CDCl3_): 210.4, 144.6, 144.5, 134.6, 129.8, 129.8, 127.8, 127.8, 118.1, 89.4, 86.9, 81.4, 73.0, 72.0, 61.7, 56.3, 44.3, 44.3, 44.0, 43.8, 44.2, 44.2, 37.7, 37.4, 35.9, 34.5, 28.0, 27.8, 27.8, 26.8, 25.8, 25.1, 23.8, 21.8, 18.9, 18.2, 16.1. HRMS (ESI): 665.35653 m*/z* [M + Na]^+^ (C_37_H_54_O_7_SNa, calcd. 665.34879).

### Reaction of AG with N-Acetylsulfanilyl chloride (N-Ac-Sulfa)

40 mg AG was dissolved in pyridine and 80 mg N-Ac-Sulfa (Acros Organics) reagent was added. Reaction was continued for 6 h at room temperature and quenched by addition of distilled water. Following the extraction via 50 ml ethyl acetate (3 times), the extract was evaporated at 60 °C in the rotary evaporator. The residue was purified by silica gel column chromatography (cyclohexane:EtOAc; 7:3) to give **AG-06** (8.3 mg lyophilized white-off powder)**.** ^1^H NMR (400 MHz, CDCl_3_): δ = 7.8 (d, J = 8.4 Hz, 2H), 7.7 (d, J = 8.8 Hz, 2H), 5.58 (d, 10.4 Hz, 1H), 5.53 (dt, J = 10.4, 3 Hz, 1H), 5.13 (t, J = 5.3 Hz, 1H), 4.69 (ddd, J = 6.5, 6.5, 6.0 Hz, 1 H), 4.15 (dd, J = 9.8, 6.6, 1H), 3.74 (t, J = 7.1 Hz, 1H), 2.8 (brs, 1H), 2.55 (q, J = 10.5 Hz, 1H), 2.18 (s, 1H), 2.12 (d, J = 7.7 Hz, 1H), 2.0 (m, 2H), 1.99 (m, 2H), 1.91 (m, 1H), 1.9 (m, 2H), 1.69 (d, J = 4.2), 1.59 (m, 1H), 1.55 (m, 2H), 1.53 (m, 1H), 1.28 (s, 3H), 1.22 (s, 3H), 1.14 (s, 3H), 0.97 (s, 3H), 0.95 (s, 3H), 0.83 (s, 3H), 0.73 (s, 3H), 0.62 (s, 3H). ^13^C NMR (100 MHz, CDCl_3_): δ = 169.3, 144.8, 143. 2, 131.8, 129.2, 129.0, 129.0, 126.1, 119.3, 119.3, 113.8, 90.9, 86.9, 81.5, 73.3, 72.0, 56.3, 52.1, 44.5, 43.8, 43.6, 43.4, 38.6, 38.2, 37.8, 34.5, 33.9, 28.0, 27.7, 27.6, 26.7, 25.8, 25.4, 24.8, 20.0, 18.8, 18.6, 16.5. HRMS (ESI): 692.36893 m*/z* [M + Na]^+^ (C_38_H_55_O_7_S Na, calcd. 692.35969).

### Reaction of AG with Dansyl Chloride

100 mg AG was dissolved in pyridine and 450 mg dansyl chloride (400 mg) reagent was added. Reaction was continued for 6 h at 70 °C and quenched by addition of distilled water. Following the extraction via 500 ml ethyl acetate (3 times), the extract was evaporated at 60 °C in the rotary evaporator. Crude product was purified by silica gel column chromatography (*n*-hexane:EtOAc; 7:3) to give **AG-07** (3.5 mg lyophilized white-off powder). ^1^H NMR (400 MHz, CDCl_3_): *δ* = 8.56 (d, J = 8.5 Hz, 1H), 8.32 (d, J = 8.6 Hz, 1H), 8.25 (dd, J = 7.3, 1.3 Hz, 1H), 7.59 (dd, J = 8.7, 7.6 Hz, 1H), 7.52 (dd, J = 8.6, 7.3 Hz, 1H), 7.19 (dd, J = 7.6, 0.9 Hz, 1H), 5.56 (m, 2H), 5.10 (m, 1H), 4.69 (ddd, J = 7.9, 7,9, 6.0 Hz, 1H), 4.28 (dd, J = 11.3, 5.2 Hz, 1H), 3.75 (dd, J = 8.7, 6.5 Hz, 1H), 2.88 (d, J = 1.0 Hz, 6H), 2.79 (s, 1H), 2.55 (q, J = 10.5 Hz, 1H), 2.21 (d, J = 7.6 Hz, 1H), 2.04 (m, 2H), 2.0 (m, 1H), 1.97 (m, 1H), 1.93 (m, 1H), 1.82 (m, 1H),1.79 (m, 1H),1.7 (brs, 1H), 1.59 (m, 1H), 1.52 (m, 1H),1.51 (m, 2H), 1.29 (s, 3H), 1.25 (s, 3H), 1.23 (s, 3H), 1.14 (s, 3H), 0.95 (s, 3H), 0.86 (s, 3H), 0.71 (s, 3H), 0.62 (s, 3H). ^13^C NMR (100 MHz, CDCl_3_): δ = 151.8, 144.9, 133.7, 131.2, 130.2, 129.89, 129.4, 129.2, 128.6, 126.2, 123.2, 120.0, 115.5, 113.7, 91.5, 87.0, 81.5, 73.3, 72.0, 56.3, 52.1, 45.6, 45.6, 44.5, 44.0, 43.6, 43.5, 38.6, 38.1, 37.8, 34.6, 33.9, 27.9, 27.8, 27.6, 26.7, 25.7, 25.3, 20.0, 18.8, 18.6, 16.6. HRMS (ESI): 728.40520 m*/z* [M + Na]^+^ (C_42_H_59_NO_6_SNa, calcd. 728.39608).

### Reaction of CG with p-TsCl

To a stirring solution of CG (200 mg, in pyridine) and 5 mg DMAP, *p*-TsCl solution (450 mg) in pyridine was added. The reaction mixture was stirred at reflux for 6 h. Reaction mixture quenched with water and extracted three times with 100 mL EtOAc. Crude product was purified on a silica gel chromatography column (cyclohexane:EtOAc; 8:2) to give **CG-02** (8.6 mg lyophilized white-off powder) and **CG-03** (95.1 mg lyophilized white-off powder). For **CG-02**; ^1^H NMR (400 MHz, CDCl_3_): *δ* = 5.63 (d, J = 10.6, 1H), 5.48 (brs, 1H), 4.69 (q, J = 7.2, 1H), 3.74 (dd, J = 7.1, 7.1, 1H), 3.32 (dd, J = 11.4, 4.6, 1H), 2.71 (d, J = 7.9, 1H), 2.59 (q, J = 10.6, 1H), 2.27 (d, J = 7.6, 1H), 2.0 (m, 2H), 1.88 (m, 1H), 1.85 (m, 1H), 1.84 (m, 1H), 1.82 (m, 1H), 1.62 (m, 1H), 1.6 (m, 1H), 1.59 (m, 1H), 1.52 (m, 1H), 1.42 (m, 1H), 1.41 (m, 1H), 1.40 (m, 1H), 1.31 (s, 3H), 1.25 (s, 1H), 1.23 (s, 6H), 1.22 (s, 3H), 1.15 (s, 3H), 1.05 (s, 2H), 0.78 (s, 3H), 0.74 (m, 1H), − 0.16 (d, J = 4.3, 1H). ^13^C NMR (100 MHz, CDCl_3_): δ = 129.2, 126.6, 87.3, 81.4, 78.5, 73.5, 72.1, 56.4, 48.5, 46.6, 45.2, 43.6, 43.4 ,40.3, 34.6, 33.4, 30.1, 29.8, 28.4, 28.1, 27.9, 26.7, 25.9, 25.6, 25.2, 20.6, 18.7, 18.2, 18.1, 14.5. HRMS (ESI): *m/z* 495.34984 [M + Na]^+^ (C_30_H_48_O_4_Na, calcd. 495.34503)_._ For **CG-03**; ^1^H NMR (400 MHz, CDCl_3_): δ = 7.79 (d, J = 8.2 Hz, 2H), 7.32 (d, J = 8.2 Hz, 2H), 5.51 (d, J = 10.7 Hz, 1H), 5.46 (dd, J = 10.7, 6, 3 Hz, 1H), 4.68 (ddd, J = 7.7, 7.7, 6.1 Hz, 1H), 4.31 (dd, J = 11.8, 4.6 Hz, 1H), 3.74 (t, J = 7.1 Hz, 1H), 2.68 (dd, J = 6.0, 2.5 Hz, 1H), 2.58 (q, J = 10.5 Hz, 1H), 2.43 (s, 3H), 2.26 (d, J = 7.7 Hz, 1H), 2.0 (td, J = 10.5, 9.5 Hz, 2H), 1.89 (m, 1H), 1.86 (m, 1H), 1.85 (m, 1H), 1.79 (m, 1H), 1.77 (m, 1H), 1.58 (m, 1H), 1.57 (m, 1H), 1.56 (m, 1H), 1.48 (m, 1H), 1.41 (m, 1H), 1.38 (m, 1H), 1.34 (m, 1H), 1.29 (s, 3H), 1.21 (s, 3H), 1.19 (s, 3H), 1.13 (s, 3H), 0.81 (s, 3H), 0.8 (s, 3H), 0.72 (d, J = 4.2 Hz, 1H), 0.71 (s, 3H), -0.17 (d, J = 4.2 Hz, 1H). ^13^C NMR (100 MHz, CDCl_3_): δ = 144.5, 134.9, 129.8, 127.8, 125.7, 90.2, 87.2, 81.3, 73.4, 72.0, 56.4, 48.4, 46.7, 45.1, 43.5, 43.3, 40.0, 34.5, 33.3, 29.5, 28.2, 27.9, 27.8, 26.7, 25.9, 25.4, 25.2, 21.8, 20.7, 18.6, 18.2, 18.1, 15.4. HRMS (ESI): *m/z* 649.36046 [M + Na]^+^ (C_37_H_54_O_6_SNa, calcd. 649.35388).

### Reaction of CG with MsCl

MsCl (140 µL) and TEA (294 µL) was added to a solution of 350 mg CG in dichloromethane. The reaction mixture was stirred at 0 °C for 3 h. The reaction solvent was evaporated under reduced pressure and the residue was precipitated in methanol to give **CG-04** (193 mg lyophilized white-off powder). ^1^H NMR (400 MHz, CDCl_3_): δ = 5.58 (d, J = 10.6 Hz, 1H), 5.49 (ddd, J = 10.6, 6.1, 3.1 Hz, 1H), 4.7 (ddd, J = 7.7, 7.7, 6.0 Hz, 1H), 4.43 (dd, J = 11.9, 4.6 Hz, 1H), 3.73 (t, J = 7.1 Hz, 1H), 3.01 (s, 3H), 2.71 (dd, J = 6.2, 2.6 Hz, 1H), 2.58 (d, J = 10.6 Hz, 1H), 2.27 (d, J = 7.6 Hz, 1H), 2.12 (dd, J = 12.5, 3.9 Hz, 1H), 2 (m, 2H), 1.96 (m, 1H), 1.9 (m, 1H), 1.89 (m, 1H), 1. 81 (m, 1H), 1.68 (m, 1H), 1.61 (m, 1H), 1.6 (m, 1H), 1.51 (m, 1H), 1.47 (m, 1H), 1.43 (m, 1H), 1.38 (m, 1H), 1.29 (s, 3H), 1.22 (s, 3H), 1.20 (s, 3H), 1.14 (s, 3H), 1.05 (s, 3H), 0.86 (s, 3H), 0.77 (d, J = 3.6 Hz, 1H), 0.74 (s, 3H), -0.12 (d, J = 4.3 Hz, 1H). ^13^C NMR (100 MHz, CDCl_3_): δ = 129.9, 125.6, 89.7, 87.2, 81.3, 73.4, 72.0, 56.4, 48.4, 46.7, 45.1, 43.5, 43.3, 40.0, 38.9, 34.5, 33.3, 29.5, 28.2, 28.2, 27.9, 27.8, 26.7, 25.9, 25.8, 25.2, 20.8, 18.6, 18.2, 18.1, 15.2. HRMS (ESI): *m/z* 573.32624 [M + Na]^+^ (C_31_H_50_O_6_SNa, calcd. 573.32258).

### Reaction of CG-01 with p-TsCl

100 mg CG was dissolved in pyridine and 300 mg *p-*TsCl reagent was added. Reaction was continued for 12 h at room temperature and quenched by addition of distilled water. Following the extraction via 500 ml ethyl acetate (3 times), the extract was evaporated at 60 °C in the rotary evaporator. Crude product was purified on a silica gel chromatography column (cyclohexane:EtOAc; 7:3) to give **CG-05** (33 mg lyophilized white-off powder). ^1^H NMR (400 MHz, CDCl_3_): δ = 7.79 (d, J = 8.3 Hz, 2H), 7.3 (d, J = 8.3 Hz, 2H), 4.69 (ddd, J = 7.8, 7.8, 6.1 Hz, 1H), 4.21 (m, 1H), 3.75 (dd, J = 8.3, 6.1 Hz, 1H), 2.66 (dd, J = 8.5, 4.0 Hz, 1H), 2.57 (q, J = 10.8 Hz, 1H), 2.43 (s, 3H), 2.32 (d, J = 7.6 Hz, 1H), 2.3 (brs, 1H), 2.17 (m, 1H), 2.12 (m, 1H), 1.98 (m, 1H),1.94 (m, 1H), 1.85 (m, 1H), 1.84 (m, 1H), 1.79 (m, 1H), 1.76 (m, 1H), 1.6 (m, 1H), 1.47 (m, 1H), 1.46 (m, 1H), 1.37 (m, 1H), 1.21 (m, 1H), 1.21 (s, 3H), 1.57 (m, 1H), 1.42 (m, 1H), 1.3 (s, 3H), 1.14 (s, 3H), 1.02 (s, 3H), 1.0 (s, 3H), 0.89 (s, 3H), 0.21 (d, J = 5.5 Hz, 1H), 0.6 (d, J = 5.5 Hz, 1H). ^13^C NMR (100 MHz, CDCl_3_): δ = 210, 144.6, 134.7, 129.8, 129.8, 127.8, 127.8, 89.3, 87.1, 81.3, 72.9, 72.1, 57.2, 56.9, 47.1, 45.3, 43.8, 42.4, 41.2, 40.0, 34.5, 33.0, 30.0, 29.7, 28.1, 27.8, 27.3, 26.8, 26.5, 26.3, 25.9, 22.2, 21.8, 21.7,19.1, 18.4, 14.8. HRMS (ESI): *m/z* 665.35109 [M + Na]^+^ (C_37_H_54_O_7_SNa, calcd. 665.34879).

### Reaction of CG with N-Ac-Sulfa

50 mg CG was dissolved in pyridine and 150 mg N-Ac-Sulfa (Acros Organics) reagent was added. Reaction was continued for 11 h at room temperature and quenched by addition of distilled water. Following the extraction via 50 ml ethyl acetate (3 times), the extract was evaporated at 60 °C in the rotary evaporator. The residue was purified by silica gel column chromatography (CHCl_3_:MeOH; 95:5) to give **CG-06** (12.1 mg). ^1^H NMR (400 MHz, CDCl_3_): δ = 7.82 (d, J = 8.5 Hz, 2H), 7.3 (d, J = 8.5 Hz, 2H), 5.50 (d, J = 10.7 Hz, 1H), 5.43 (ddd, J = 0.2, 6.2, 2.9 Hz, 1H), 4.67 (q, J = 4.2 Hz, 1H)), 4.26 (dd, J = 11.8, 4.7 Hz, 1H), 3.73 (dd, J = 6.9 Hz, 1H), 2.66 (m, 1H), 2.57 (q, J = 10.8 Hz, 1H), 2.26 (d, J = 7.5, Hz, 1H), 2.17 (s, 3H), 1.98 (m, 2H), 1.87 (m, 1H), 1.85 (m, 1H), 1.78 (m, 1H), 1.77 (m, 1H), 1.57 (m, 1H), 1.56 (m, 1H), 1.56 (m, 1H), 1.37 (m, 1H), 1.33 (m, 1H), 1.85 (m, 1H), 1.48 (m, 1H), 1.40 (m, 1H), 1.27 (s, 3H), 1.18 (s, 3H), 1.18 (s, 3H), 1.13 (s, 3H), 0.8 (s, 3H), 0.78 (s, 3H), 0.69 (s, 3H), 0.69 (d, J = 4.1 Hz, 1H), − 0.18 (d, J = 4.1 Hz, 1H). ^13^C NMR (100 MHz, CDCl_3_): δ = 169.3, 142.2, 131.8, 129.7, 129.0, 129.0, 125.6, 119.3, 119.3, 90.4, 87.1, 81.3, 73.4, 72.0, 56.3, 48.3, 46.6, 45.0, 43.4, 43.2, 40.0, 34.5, 33.2, 29.5, 28.2, 27.8, 27.7, 27.7, 26.7, 25.9, 25.4, 25.1, 24.7, 20.7, 18.6, 18.1, 18.1, 15.3. HRMS (ESI): *m/z* 692.36865 [M + Na]^+^ (C_38_H_57_NO_8_SNa, calcd. 692.35969).

### Reaction of SCG with TsCl

500 mg SCG was dissolved in pyridine and 1000 mg *p*-TsCl reagent was added. The reaction mixture was stirred at reflux for 6 h and quenched by addition of distilled water. Following the extraction via 500 ml ethyl acetate (3 times), the extract was evaporated at 60 °C in the rotary evaporator. Crude product was purified on a silica gel chromatography column (*n*-hexane:EtOAc; 90:10) to give **SCG-01** (15.4 mg lyophilized white-off powder), **SCG-02** (8.6 mg lyophilized white-off powder), **SCG-03** (13 mg lyophilized white-off powder), **SCG-04** (19.2 mg lyophilized white-off powder), **SCG-05** (15.2 mg lyophilized white-off powder). For **SCG-01**; ^1^H NMR (400 MHz, CDCl_3_): δ = 7.75 (d, J = 8.2 Hz, 2H), 7.31 (d, J = 8.2 Hz, 2H), 1.2 (d, J = 3.2 Hz, 1H), 5.06 (ddd, J = 15.4, 7.9, 1.4 Hz, 1H), 3.51 (ddd, J = 9.1, 9.1, 4.2 Hz, 1H), 3.29 (dd, J = 11.3, 4.6 Hz, 1H), 2.44 (s, 3H), 1.92 (m, 1H), 1.88 (m, 2H), 1.86 (m, 1H), 1.78 (m, 1H), 1.64 (d, J = 1.4 Hz, 1H), 1.6 (m, 2H), 1.57 (m, 1H), 1.56 (m, 1H), 1.44 (m, 1H), 1.4 (m, 1H), 1.34 (d, J = 1.97 Hz, 1H), 1.3 (m, 1H), 0.95 (s, 3H), 1.25 (m, 1H), 1.22 (s, 3H), 1.04 (s, 3H), 0.92 (s, 3H), 0.45 (d, J = 4.6 Hz, 1H), 0.28 (d, J = 4.6 Hz, 1H). ^13^C NMR (100 MHz, CDCl_3_): δ = 144.6, 134.4, 129.9, 129.9, 127.8, 127.8, 82.9, 78.4, 68.5, 53.5, 46, 45.9, 45.8, 44.5, 44.4, 41.6, 37.5, 32.0, 30.3, 30.3, 30.0, 29.8, 28.0, 26.1, 25.0, 21.8, 20.8, 19.8, 15.3. HRMS (ESI): *m/z* 525.2699 [M + Na]^+^ (C_29_H_42_O_5_SNa, calcd. 525.26506).

For **SCG-02;** ^1^H NMR (400 MHz, CDCl_3_): δ = 5.62 (d, J = 10.5 Hz, 1H), 5.44 (ddd, J = 10.6, 6.1, 3.2 Hz, 1H), 4.55 (ddd, J = 14.5, 7.7, 1.4 Hz, 1H), 3.3 (dd, J = 11.2, 4.4 Hz, 1H), 2.48 (dd, J = 6.2, 2.6 Hz, 1H), 2.05 (dd, J = 13.7, 8.2 Hz, 1H), 1.89 (m, 1H), 1.86 (m, 1H), 1.85 (m, 1H), 1.82 (m, 1H), 1.69 (m, 1H), 1.62 (m, 1H), 1.6 (m, 1H), 1.46 (m, 1H), 1.42 (m, 1H), 1.27 (m, 1H), 1.25 (m, 1H), 1.22 (m, 1H), 1.05 (s, 3H), 0.96 (s, 3H), 0.92 (s, 3H), 0.77 (s, 3H), 0.73 (d, J = 4.5 Hz, 1H), − 0.15 (d, J = 4.1 Hz, 1H). ^13^C NMR (100 MHz, CDCl_3_): δ = 129.1, 126.3, 78.5, 72.2, 48.5, 47.8, 46.5, 45.4, 45.3, 43.7, 40.3, 31.4, 30.1, 29.8, 28.3, 25.6, 25.2, 22.2, 21.2, 18.5, 18.4, 14.5. HRMS (ESI): *m/z* 331.26321 [M + H]^+^ calcd for C_22_H_34_O_2_. For **SCG-03**; ^1^H NMR (400 MHz, CDCl_3_): δ = 7.74 (dd, J = 8.2, 3.6 Hz, 2H), 7.31 (dd, J = 8.2, 3.6 Hz, 2H), 5.49 (ddd, J = 9.9, 5.8, 2.0 Hz, 1H), 5.3 (dd, J = 9.8, 2.7 Hz, 1H), 5.04 (q, J = 7.4 Hz, 1H), 3.45 (td, J = 9.8, 4.7 Hz, 1H), 2.44 (s, 3H), 2.30 (m, 1H), 2.07 (m, 1H), 1.90 (m, 1H), 1.88 (m, 1H), 1.87 (m, 1H), 1.65 (m, 1H), 1.62 (m, 1H),1.58 (m, 1H), 1.54 (m, 1H), 1.47 (m, 1H), 1.39 (m, 1H), 1.34 (m, 1H), 1.23 (s, 3H), 1.1 (m, 1H), 1.05 (s, 3H), 1.03 (s, 3H), 0.99 (s, 3H), 0.51 (d, J = 5.0 Hz, 1H), 0.34 (d, J = 4.5 Hz, 1H). ^13^C NMR (100 MHz, CDCl_3_): δ = 144.6, 140.7, 134.2, 129.9, 129.9, 127.8, 127.8, 122.8, 83.0, 70.7, 52.6, 47.9, 46.1, 45.9, 44.9, 44.4, 38.0, 34.9, 33.1, 31.7, 30.2, 28.4, 28.2, 25.8, 25.7, 23.5, 21.8, 20.2, 19.2. HRMS (ESI): *m/z* 507.2578 [M + Na]^+^ (C_29_H_40_O_4_SNa, calcd. 507.25450). For **SCG-04**; ^1^H NMR (400 MHz, CDCl_3_): δ = 7.8 (d, J = 7.7 Hz, 2H), 7.75 (d, J = 7.8 Hz, 2H), 7.32 (d, J = 7.8 Hz, 4H), 5.52 (d, J = 10.7 Hz, 1H), 5.36 (ddd, J = 9.6, 5.9, 2.9 Hz, 1H), 5.06 (q, J = 7.5 Hz, 1H), 4.30 (dd, J = 11.6, 4.5 Hz, 1H), 2.44 (s, 6H), 2.41 (m, 1H), 2.0 (m, 1H), 1.9 (m, 1H), 1.89 (m, 1H), 1.81 (m, 2H), 1.8 (m, 1H), 1.8 (m, 1H), 1.61 (m, 1H), 1.6 (m, 1H), 1.5 (m, 1H), 1.4 (m, 1H), 1.39 (m, 1H), 1.16 (m, 1H), (4.1), 0.88 (s, 3H), 0.83 (s, 3H), 0.82 (s, 3H), 0.79 (s, 3H), 0.71 (d, J = 4.1 Hz, 1H), -0.16 (d, J = 4.1 Hz, 1H). ^13^C NMR (100 MHz, CDCl_3_): δ = 144.7, 144.4, 134.7, 134.2, 128.9, 129.7, 129.7, 129.7, 129.7, 127.7, 127.7, 127.7, 127.7, 125.7, 89.9, 82.4, 48.1, 46.5, 43.1, 44.9, 44.4, 41.9, 39.8, 30.7, 29.4, 27.7, 27.6, 25.3, 24.8, 21.8, 21.6, 21.6, 20.9, 18.3, 17.6, 15.2. HRMS (ESI): *m/z*.656.2970 [M + NH_4_]^+^ (C_36_H_46_O_6_S_2_NH_4_, calcd. 656.30795). For **SCG-05**; ^1^H NMR (400 MHz, CDCl_3_): δ = 7.76 (d, J = 8.1 Hz, 2H), 7.32 (d, J = 7.9 Hz, 2H), 5.61 (d, J = 10.5 Hz, 1H), 5.37 (m, 1H), 5.07 (dd, J = 14.7, 7.2 Hz, 1H), 3.33 (dd, J = 10.8, 3.9 Hz, 1H), 2.44 (m, 4H), 2.01 (m, 1H), 1.86 (d, J = 2.4 Hz, 1H), 1.83 (m, 2H), 1.82 (m, 1H), 1.81 (d, J = 5.6 Hz, 1H), 1.63 (d, J = 12.9 Hz, 1H), 1.62 (m, 1H), 1.53 (d, J = 14.7 Hz, 1H), 1.43 (m, 1H), 1.42 (m, 1H), 1.23 (s, 3H), 1.2 (dd, J = 12.7, 4.3 Hz, 1H), 1.05 (s, 3H), 0.9 (s, 3H), 0.85 (s, 3H), 0.77 (s, 3H), 0.72 (d, J = 3.1 Hz, 1H), -0.15 (d, J = 3.9 Hz, 1H). ^13^C NMR (100 MHz, CDCl_3_): δ = 144.5, 134.5, 129.9, 129.9, 128.5, 127.8, 127.8, 126.9, 82.7, 78.5, 48.4, 46.5, 45.2, 44.6, 43.3, 42.1, 40.4, 31.0, 30.1, 29.8, 28.3, 25.6, 25.0, 21.8, 21.6, 21.1, 18.5, 17.9, 14.5. HRMS (ESI): *m/z* 502.2940 [M + NH_4_]^+^ (C_29_H_40_O_4_SNH_4_, calcd. 502.29910).

### Reaction of SCG with MsCl

130 µL MsCl was added to a solution of SCG (150 mg) in pyridine. The reaction mixture was stirred at reflux for 6 h. The reaction solvent was then removed under reduced pressure, and the residue was purified by silica gel column chromatography (n-hexane:EtOAc; 90:10) to give **SCG-06** (13 mg lyophilized white-off powder), and **SCG-07** (4.3 mg lyophilized white-off powder). For **SCG-06;** ^1^H NMR (400 MHz, CDCl_3_): δ = 5.61 (d, J = 10.5 Hz, 1H), 5.45 (ddd, J = 10.3, 6.1, 3.0 Hz, 1H), 5.29 (q, J = 7.6 Hz, 1H), 4.45 (dd, J = 12.0, 4.6 Hz, 1H), 3.03 (s, 3H), 2.97 (s, 3H), 2.51 (dd, J = 6.0, 2.6 Hz, 1H), 2.2 (m, 1H), 2.14 (m, 1H), 2.04 (m, 1H), 2.0 (m, 1H), 1.93 (m, 1H), 1.92 (m, 1H), 1.92 (m, 1H),1.89 (m, 1H), 1.70 (m, 1H), 1.69 (m, 1H), 1.63 (m, 1H), 1.43 (m, 1H), 1.27 (dd, J = 13, 5.1 Hz, 1H), 1.10 (s, 3H), 0.98 (s, 3H), 0.87 (s, 3H), 0.86 (s, 3H), 0.77 (d, J = 4.3 Hz, 1H), -0.07 (d, J = 4.3 Hz, 1H). ^13^C NMR (100 MHz, CDCl_3_): *δ* = 129.2, 125.9, 89.5, 82.0, 48.4, 46.7, 45.2, 44.7, 43.2, 42.3, 40.0, 39.0, 38.5, 30.9, 29.5, 28.1, 27.8, 25.8, 25.0, 21.2, 22.0, 18.4, 17.9, 15.3. HRMS (ESI): *m/z* 995.3974 [2M + Na]^+^ (C_48_H_76_O_12_S_4_Na, calcd. 995.41173). **For SCG-07**; ^1^H NMR (400 MHz, CDCl_3_): δ = 5.26 (q, J = 8.0 Hz, 1H), 3.56 (ddd, J = 9.2, 9.2, 4.1 Hz, 1H), 3.22 (dd, J = 11.2, 4.2 Hz, 1H), 2.97 (s, 3H,) 2.11 (m, 1H), 2.06 (m, 1H), 2.01 (m, 1H), 1.97 (m, 1H), 1.8 (m, 1H), 1.79 (m, 1H), 1.71 (m, 1H), 1.63 (m, 1H), 1.58 (m, 1H), 1.55 (m, 1H), 1.48 (m, 1H), 1.38 (m, 1H), 1.37 (m, 1H), 1.31 (m, 1H), 1.26 (m, 1H), 1.23 (s, 3H), 1.10 (s, 3H), 1.03 (s, 3H), 0.94 (s, 3H), 0.5 (d, J = 4.7 Hz, 1H), 0.31 (d, J = 4.7 Hz, 1H). ^13^C NMR (100 MHz, CDCl_3_): δ = 82.4, 78.5, 68.4, 53.4, 46.2, 46.0, 45.6, 44.7, 44.6, 41.6, 37.6, 31.3, 30.3, 30.2, 29.0, 27.9, 26.7, 26.1, 24.9, 20.7, 19.8, 38.5, 15.3. HRMS (ESI): *m/z* 449.2366 [M + Na]^+^ (C_23_H_38_O_5_SNa, calcd. 449.23376).

### Biological studies

#### Chemicals

Pitstop II (Sigma; SML1169), methyl β cyclodextrin (Cayman; 21,633), chlorpromazine (Cayman; 16,129), dynasore (Cayman, 14,062) and cytochalasin D (Cayman; 11,330) were used as endocytosis inhibitors.

Derivatives dissolved in anhydride DMSO as a 1000-fold concentrated stock. The dissolved compounds are immediately used for the treatment of cells.

#### Cell culture

HCC1937 (Human breast cancer line), HeLa (Human endometrial carcinoma), A549 (Human lung adenocarcinoma), MRC-5 (Human lung fibroblasts), MCF7 (Human breast cancer cells), A549 (human lung carcinoma) were obtained from American Type Culture Collection and maintained as exponentially growing monolayers by culturing according to the supplier’s instructions. MCF7, HeLa, MRC-5, and A549 cell lines were cultured and routinely passaged in DMEM media containing 10% FBS, while HCC1937 cell lines were propagated in RPMI 1640 containing 10% FBS.

#### Cytotoxicity analysis

Following the treatment with compounds or vehicle for 48 h, the 10% WST-1 (Roche, Switzerland) in medium was replaced in each 96 well. After 4 h incubation with WST-1 reagent at 37 °C and 5% CO_2_, the absorbance was measured using a microplate reader at 440 nm (Varioscan, Thermo Fisher Scientific, US). Graph Pad Prism 5 (San Diego, CA, US.) was used to calculate the IC_50_, which represents the concentration of compounds that is required for 50% inhibition in comparison to the vehicle-treated controls. The experiments were repeated three times independently.

#### LDH (lactate dehydrogenase) releasing assay

LDH-Cytotoxicity Colorimetric Assay Kit II (Biovision) was used to detect LDH releasing. Briefly, HCC1937 were seeded on 96 well plate. After treatment with compounds, cells were centrifuged at 600×*g* for 10 min. 10 µl supernatant were transferred into clean 96 well plate and added into 100 µl LDH Reaction Mix. Following 30 min incubation at room temperature, 10 µl stop solution were added, and absorbance was measured at 450 nm (Varioscan, Thermo Fisher Scientific, US).

#### Immunofluorescence studies

Cells were grown on glass coverslips and treated with AG-08. At the end of treatments, cells were washed twice with ice-cold PBS and fixed with 4% paraformaldehyde in PBS for 30 min at 4 °C. After washing 6 times with PBS, cells were permeabilized and blocked with 0.01% saponin and 0,01% BSA in PBS. Then fixed cells were incubated with primary antibodies for 1 h at 37 °C. Antibodies were used at the following dilutions: Calnexin (Sigma Aldrich-C7617, UK), 1:200; EEA1 (CST-3288, US), 1:100. Cells were then incubated with secondary antibodies (1:400) for 1 h at 37 °C. Mounted samples were analyzed by fluorescence microscopy (Olympus IX70, Japan).

#### Total RNA isolation and expression analysis by quantitative RT-PCR

The total RNA was isolated using Total RNA Isolation Kit (Bio-Rad, US) following the manufacturer’s instructions. cDNAs were synthesized using The iScript cDNA synthesis kit (Bio-Rad, US) according to the manufacturer’s instructions. To gene expression analysis, specific primers were designed against CHOP (forward: AGTCTAAGGCACTGAGCGTATCAT, reverse: CTTTCAGGTGTGGTGATGTATGAA ). Quantitative RT-PCR (qRT-PCR) was performed using The SYBR Green I Mastermix (Bio-Rad, US) and LightCycler480 thermocycler (Roche). Fold change for the transcripts were normalized to the housekeeping gene TBP1 (TATA-Box Binding Protein1) (forward: GAGTTCTGGGATTGTACCGCA, reverse: CGTGGTTCGTGGCTCTCTT). For relative quantification, reaction efficiency incorporated ΔΔCq formula was used. Six independent biological replicates with two technical replicates per experiment were used for each PCR.

#### Western blot analysis

Cell lysates were prepared by RIPA buffer (1XPBS, 1% nonidet P-40, 0.5% sodium deoxycholate, and 0.1% SDS, pH 8.0). Protein concentrations were determined by bicinchoninic acid (BCA) protein assay (Thermo Fisher Scientific, US). After equal amounts of proteins were loaded to the gels, proteins were separated by SDS-PAGE electrophoresis and transferred to PVDF membranes (EMD Millipore, Thermo Fisher Scientific, US). Following the classic immunoblotting steps (blocking, incubating with primary and secondary antibodies), chemiluminescence signals were detected using Clarity ECL substrate solution (Bio-Rad, US) by Fusion-FX7 (Vilber Lourmat, Thermo Fisher Scientific, US). Monoclonal antibodies used in this study were anti-LC3 (CST-12741, USA), anti-Atg-5/12 (CST-12994, USA), anti-Atg-7 (CST-8558, US), anti-actin (Sigma-Aldrich-A5316, UK), anti-caspase-3 (CST-9665, US), Anti-CHOP (CST-2895, UK). The experiments were repeated three times independently; with one representative result shown.

#### Microaaray analysis

Microarray analyses were conducted using the human Clariom D Affymetrix platform following the manufacturer’s instructions (902915, Thermo Fisher Scientific). Briefly, the total RNA was isolated using Total RNA Isolation Kit (Bio-Rad, US). Isolated total RNA was amplified, labeled, and hybridized on Clariom D Affymetrix platform. The arrays were stained by using GeneChip Fluidics Station 450. The cartridge was scanned with the GeneChip Scanner 3000 7G System (Affymetrix CA, USA).

#### Protein‐protein interaction network and enrichment

Protein network analysis was performed using with Search Tool for the Retrieval of Interacting Genes/Proteins (STRING 11; https://string-db.org/). The confidence score for selection was ≥ 0.4. Disconnected nodes in the network were hidden to simplify the display.

The Metascape online tool was used to thoroughly analyze enrichment information (Metascape; www.metascape.org/). DE gene lists were supplied to the Metascape tool to produce enriched GO terms, KEGG pathways^34^. Terms with P < 0.01 were considered significantly enriched. Additionally, Reactome database was used for additional enrichment analysis (https://reactome.org/)^35^.

### Self-aggregation molecular dynamics simulations

All simulations were conducted using Gromacs version 2018.1^36^. AG and AG-08 molecules were first defined in mol2 file format and submitted for ligand parametrization at the Automated Topology Builder (ATB) server^37^. For each simulation, a pre-defined amount of molecules were first randomly placed within a pre-defined box, and the system was solvated. For each simulation, a minimization was for a maximum of 50,000 steps until the maximum force was below 10 kJ/mol. Here, cutoff values of 1.2, 1.4, and 1.4 nm were used for neighbor lists and as Coulomb and Van der Waals interactions, respectively. Particle Mesh Ewald (PME) method was used for treatment of long-range electrostatic interactions. Following energy minimization, the systems were subjected to NVT and NPT equilibration for 50,000 steps each at 300 K and 1 bar using the same cutoff values and settings as in minimization. Finally, NPT simulations were conducted for 250 ns ns to test self-aggregation behavior.

### Characterization of particles

#### STEM microscopy

Firstly, AG-08 was dissolved in DMSO as a 1000-fold concentrated stock and immediately added in filtered PBS. Then the solution was placed on grids (formvar/carbon-coated 400 mesh copper grids). After completely dry, images were taken via scanning transmission electron microscope (STEM-Zeiss Sigma 500). Additionally, 1% uranyl acetate (pH 7.4) was used to achieve picture with negative staining. After washing of uranyl acetate via water, images were recorded via STEM.

#### Nile red staining

AG-08 and AG were dissolved in DMSO as a 1000-fold concentrated stock added into the filtered PBS, which contain 2.5 µM Nile red. After 3 h incubation at room temperature, the fluorescence intensity was measured with a microplate reader (550 nm for excitation and 635 nm for the emission wavelength) (Varioscan, Thermo Fisher Scientific, US).

#### Measurement of particle diameter via Zeta Sizer

AG-08 was dissolved in DMSO a 1000-fold concentrated stock and added into the filtered PBS. Then particle diameter was immediately measured via zeta-sizer (DLS–Particulate Systems). Measurement was repeated in triplicate.

### Statistical analysis

The statistical significance of differences between groups was assessed by two-tailed equal variance Student’s t-test or one-way ANOVA using GraphPad Prism software.

## Supplementary Information

Supplementary Information.

## Data Availability

The data that support the findings of this study are available from the corresponding authors (EB and PBK) upon reasonable request.
