# Supplementary material for: Non-apoptotic cell death induction via sapogenin based supramolecular particles
Source: Sci Rep. 2022 Aug 16;12:13834. doi: 10.1038/s41598-022-17977-4 (PMC9381536; doi:10.1038/s41598-022-17977-4)
Supplement: Supplementary file 1 — Supplementary Information. [file 41598_2022_17977_MOESM1_ESM.pdf]

Non-apoptotic cell death induction via sapogenin based supramolecular particles

Göklem Üner[1], Erdal Bedir[1]*, Onur Serçinoğlu[2], Petek Ballar Kırmızıbayrak[3]*

[1] Department of Bioengineering, Faculty of Engineering, İzmir Institute of Technology, 35430 Urla, İzmir, Turkey

[2] Department of Bioengineering, Faculty of Engineering, Gebze Technical University, 41400 Gebze, Kocaeli, Turkey

[3] Department of Biochemistry, Faculty of Pharmacy, Ege University, 35100 Bornova, İzmir, Turkey

***Corresponding Authors:**

Erdal Bedir: İzmir Institute of Technology, 35430 Urla/İzmir, Turkey; phone: +90(232) 750-7389 email: erdal.bedir@iyte.edu.tr

Petek Ballar Kırmızıbayrak: Ege University, 35100 Bornova/İzmir, Turkey; phone: +90(232) 311 3141; email: petek.ballar@ege.edu.tr

CONTENT

Supplementary Figure 1. AG-08 particles affect endosomal pathway 8

Supplementary Table 1. Gene expression level, p value and FDR p value of AG-08 and control. 8

Supplementary Figure 2. Cytotoxic compounds similarly affect LC3-II, caspase 3 and Atg7 proteins.. 15

Supplementary Figure 3. Chemical Structure of AG-02 16

Supplementary Table 2. The ^{13}C and ^{1}H NMR data of AG-02 (100/400 MHz, δ ppm, in $CDCl_3$)16

Spectrum 1. HR-ESI-MS Spectrum of AG-02 (positive mode) 17

Spectrum 2. ^{1}H NMR Spectrum of AG-02. 18

Spectrum 3. ^{13}C NMR Spectrum of AG-02. 18

Spectrum 4. DEPT135 spectrum of AG-02 19

Spectrum 5. COSY spectrum of AG-02 19

Spectrum 6. HMQC spectrum of AG-02. 20

Spectrum 7. HMBC spectrum of AG-02. 20

Supplementary Figure 4. Chemical Structure of AG-03 21

Supplementary Table 3. The ^{13}C and ^{1}H NMR data of AG-03 (100/400 MHz, δ ppm, in $CDCl_3$). 21

Spectrum 8. HR-ESI-MS Spectrum of AG-03 (positive mode). 22

Spectrum 9. 1H NMR Spectrum of AG-03 22

Spectrum 10. ^{13}C NMR Spectrum of AG-03. 23

Spectrum 11. DEPT135 spectrum of AG-03. 23

Spectrum 12. COSY spectrum of AG-03. 24

Spectrum 13. HMQC spectrum of AG-03 24

Spectrum 14. HMBC spectrum of AG-03. 25

Supplementary Figure 5. Chemical Structure of AG-04 25

Supplementary Table 4. The ^{13}C and ^{1}H NMR data of AG-04 (100/400 MHz, δ ppm, in CDCl3). 26

Spectrum 15. HR-ESI-MS Spectrum of AG-04 (positive mode). 27

Spectrum 16. ^{1}H NMR Spectrum of AG-04. 27

Spectrum 17. ^{13}C NMR Spectrum of AG-04. 28

Spectrum 18. DEPT135 spectrum of AG-04 28

Spectrum 19. COSY spectrum of AG-04. 29

Spectrum 20. HMQC spectrum of AG-04 29

Spectrum 21. HMBC spectrum of AG-04 30

Supplementary Figure 6. Chemical Structure of AG-05 30

Supplementary Table 5. The ^{13}C and ^{1}H NMR data of AG-05 (100/400 MHz, δ ppm, in CDCl3). 30

Spectrum 22. HR-ESI-MS Spectrum of AG-05 (positive mode). 31

Spectrum 23. ^{1}H NMR Spectrum of AG-05. 32
Spectrum 24. ^{13}C NMR Spectrum of AG-05. 32
Spectrum 25. COSY spectrum of AG-05 33
Spectrum 26. HSQC spectrum of AG-05. 33
Spectrum 27. HMBC spectrum of AG-05. 34
Spectrum 28. HMBC spectrum of AG-05. 34
Supplementary Figure 7. Chemical Structure of AG-06 35
Supplementary Table 6. The ^{13}C and ^{1}H NMR data of AG-06 (100/400 MHz, δ ppm, in CDCl3). 35
Spectrum 29. HR-ESI-MS Spectrum of AG-06 (positive mode). 36
Spectrum 30. ^{1}H NMR Spectrum of AG-06. 37
Spectrum 31. ^{13}C NMR Spectrum of AG-06. 37
Spectrum 32. DEPT135 spectrum of AG-06 38
Spectrum 33. COSY spectrum of AG-06 38
Spectrum 34. HSQC spectrum of AG-06. 39
Spectrum 36. HMBC spectrum of AG-06 40
Supplementary Figure 8. Chemical Structure of AG-07 40
Spectrum 37. HR-ESI-MS Spectrum of AG-07 (positive mode). 41
Spectrum 38. ^{1}H NMR Spectrum of AG-07. 41
Spectrum 39. ^{13}C NMR Spectrum of AG-07. 42
Supplementary Figure 9. Chemical Structure of CG-02 42
Supplementary Table 7. The ^{13}C and ^{1}H NMR data of CG-02 (100/400 MHz, δ ppm, in CDCl3). 42
Spectrum 40. HR-ESI-MS Spectrum of CG-02 (positive mode). 44
Spectrum 41. ^{1}H NMR Spectrum of CG-02. 44
Spectrum 42. ^{13}C NMR Spectrum of CG-02. 45
Spectrum 43. DEPT135 spectrum of CG-02. 45
Spectrum 44. COSY spectrum of CG-02. 46
Spectrum 45. HMQC spectrum of CG-02. 46
Spectrum 46. HMBC spectrum of CG-02. 47
Supplementary Figure 10. Chemical Structure of CG-03 47
Supplementary Table 8. The ^{13}C and ^{1}H NMR data of CG-03 (100/400 MHz, δ ppm, in CDCl3). 48
Spectrum 47. HR-ESI-MS Spectrum of CG-03 (positive mode). 49
Spectrum 48. ^{1}H NMR Spectrum of CG-03. 49
Spectrum 49. ^{13}C NMR Spectrum of CG-03. 50
Spectrum 50. DEPT135 spectrum of CG-03. 50
Spectrum 51. COSY spectrum of CG-03. 51

Spectrum 52. HMQC spectrum of CG-03. 51
Spectrum 53. HMBC spectrum of CG-03. 52
Supplementary Figure 11. Chemical Structure of CG-04 52
Supplementary Table 9. The ^{13}C and ^{1}H NMR data of CG-04 (100/400 MHz, δ ppm, in CDCl3). 53
Spectrum 54. HR-ESI-MS Spectrum of CG-04 (positive mode). 54
Spectrum 55. ^{1}H NMR Spectrum of CG-04. 54
Spectrum 56. ^{13}C NMR Spectrum of CG-04. 55
Spectrum 57. DEPT135 spectrum of CG-04. 55
Spectrum 58. COSY spectrum of CG-04. 56
Spectrum 59. HMQC spectrum of CG-04. 56
Spectrum 60. HMBC spectrum of CG-04. 57
Supplementary Figure 12. Chemical Structure of CG-05 57
Supplementary Table 10. The ^{13}C and ^{1}H NMR data of CG-05 (100/400 MHz, δ ppm, in CDCl3). 57
Spectrum 61. HR-ESI-MS Spectrum of CG-05 (positive mode). 58
Spectrum 62. ^{1}H NMR Spectrum of CG-05. 59
Spectrum 63. ^{13}C NMR Spectrum of CG-05. 59
Spectrum 64. DEPT135 spectrum of CG-05. 60
Spectrum 65. COSY spectrum of CG-05. 60
Spectrum 66. HMQC spectrum of CG-05. 61
Spectrum 67. HMBC spectrum of CG-05. 61
Supplementary Figure 13. Chemical Structure of CG-06 62
Supplementary Table 11. The ^{13}C and ^{1}H NMR data of CG-06 (100/400 MHz, δ ppm, in CDCl3). 62
Spectrum 68. HR-ESI-MS Spectrum of CG-06 (positive mode). 63
Spectrum 69. ^{1}H NMR Spectrum of CG-06. 63
Spectrum 70. ^{13}C NMR Spectrum of CG-06. 64
Spectrum 71. DEPT135 spectrum of CG-06 64
Spectrum 72. COSY spectrum of CG-06 65
Spectrum 73. HMQC spectrum of CG-06. 65
Spectrum 74. HMBC spectrum of CG-05. 66
Supplementary Figure 14. Chemical Structure of SCG-01 66
Supplementary Table 12. The ^{13}C and ^{1}H NMR data of SCG-01 (100/400 MHz, δ ppm, in CDCl3). 66
Spectrum 75.HR-ESI-MS Spectrum of SCG-01 (positive mode). 67
Spectrum 76. ^{1}H NMR Spectrum of SCG-01 68
Spectrum 77. ^{13}C NMR Spectrum of SCG-01. 68

Spectrum 78. DEPT135 spectrum of SCG-01. 69
Spectrum 79. COSY spectrum of SCG-01. 69
Spectrum 80. HMQC spectrum of SCG-01. 70
Spectrum 81. HMBC spectrum of SCG-01. 70
Supplementary Figure 15. Chemical Structure of SCG-02 71
Supplementary Table 13. The ^{13}C and ^{1}H NMR data of SCG-02 (100/400 MHz, δ ppm, in CDCl3). 71
Spectrum 82. HR-ESI-MS Spectrum of SCG-02 (positive mode). 72
Spectrum 83. ^{1}H NMR Spectrum of SCG-02. 72
Spectrum 84. ^{13}C NMR Spectrum of SCG-02. 73
Spectrum 85. DEPT135 spectrum of SCG-02. 73
Spectrum 86. COSY spectrum of SCG-02. 74
Spectrum 87. HSQC spectrum of SCG-02. 74
Spectrum 88. HMBC spectrum of SCG-02. 75
Supplementary Figure 16. Chemical Structure of SCG-03 75
Supplementary Table 14. The ^{13}C and ^{1}H NMR data of SCG-03 (100/400 MHz, δ ppm, in CDCl3). 75
Spectrum 89. HR-ESI-MS Spectrum of SCG-03 (positive mode). 76
Spectrum 90. ^{1}H NMR Spectrum of SCG-03. 77
Spectrum 91. ^{13}C NMR Spectrum of SCG-03. 77
Spectrum 92. DEPT135 spectrum of SCG-03. 78
Spectrum 93. COSY spectrum of SCG-03. 78
Spectrum 94. HMQC spectrum of SCG-03. 79
Spectrum 95. HMBC spectrum of SCG-03. 79
Supplementary Figure 17. Chemical Structure of SCG-04 80
Supplementary Table 15. The ^{13}C and ^{1}H NMR data of SCG-04 (100/400 MHz, δ ppm, in CDCl3). 80
Spectrum 96. HR-ESI-MS Spectrum of SCG-04 (positive mode). 81
Spectrum 97. ^{1}H NMR Spectrum of SCG-04. 81
Spectrum 98. ^{13}C NMR Spectrum of SCG-04. 82
Spectrum 99. COSY spectrum of SCG-04. 82
Spectrum 100. HMQC spectrum of SCG-04. 83
Spectrum 101. HMBC spectrum of SCG-04. 83
Spectrum 102. HMBC spectrum of SCG-04. 84
Supplementary Figure 18. Chemical Structure of SCG-05 84
Supplementary Table 16. The ^{13}C and ^{1}H NMR data of SCG-05 (100/500 MHz, δ ppm, in CDCl3). 85
Spectrum 103. HR-ESI-MS Spectrum of SCG-05 (positive mode). 85

Spectrum 104. ^{1}H NMR Spectrum of SCG-05. 86
Spectrum 105. ^{13}C NMR Spectrum of SCG-05. 86
Spectrum 106. DEPT135 spectrum of SCG-05. 87
Spectrum 107. COSY spectrum of SCG-05. 87
Spectrum 108. HMQC spectrum of SCG-05. 88
Spectrum 109. HMBC spectrum of SCG-05. 88
Supplementary Figure 19. Chemical Structure of SCG-06 89
Supplementary Table 17. The ^{13}C and ^{1}H NMR data of SCG-06 (100/500 MHz, δ ppm, in CDCl3). 89
Spectrum 110. HR-ESI-MS Spectrum of SCG-06 (positive mode). 90
Spectrum 111. ^{1}H NMR Spectrum of SCG-06. 90
Spectrum 112. ^{13}C NMR Spectrum of SCG-06. 91
Spectrum 113. DEPT135 spectrum of SCG-06. 91
Spectrum 114. COSY spectrum of SCG-06. 92
Spectrum 115. HMQC spectrum of SCG-06. 92
Spectrum 116. HMBC spectrum of SCG-06. 93
Supplementary Figure 20. Chemical Structure of SCG-07 93
Supplementary Table 18. The ^{13}C and ^{1}H NMR data of SCG-07 (100/500 MHz, δ ppm, in CDCl3). 94
Spectrum 117. HR-ESI-MS Spectrum of SCG-07 (positive mode). 94
Spectrum 118. ^{1}H NMR Spectrum of SCG-07. 95
Spectrum 119. ^{13}C NMR Spectrum of SCG-07. 95
Spectrum 120. DEPT135 spectrum of SCG-07. 96
Spectrum 121. COSY spectrum of SCG-07. 96
Spectrum 122. HMQC spectrum of SCG-07. 97
Spectrum 123. HMBC spectrum of SCG-07. 97
Supplementary Figure 4. Display of original blots 98

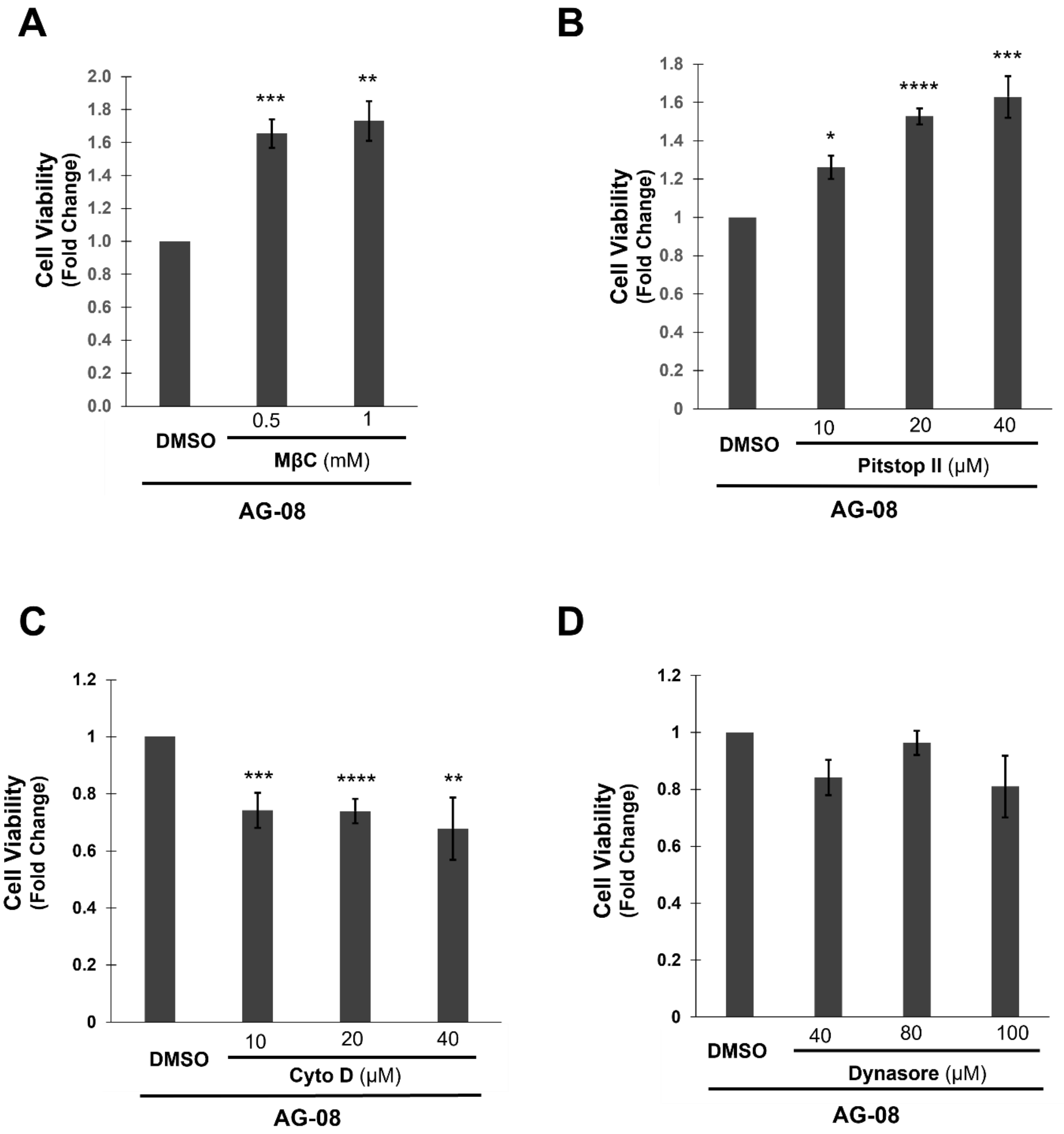
A
Cell Viability (Fold Change)
2.0
1.8
1.6
1.4
1.2
1.0
0.8
0.6
0.4
0.2
0.0
***
**
DMSO
0.5
1
MβC (mM)
AG-08
B
Cell Viability (Fold Change)
1.8
1.6
1.4
1.2
1
0.8
0.6
0.4
0.2
0
*
****
***
DMSO
10
20
40
Pitstop II (μM)
AG-08
C
Cell Viability (Fold Change)
1.2
1
0.8
0.6
0.4
0.2
0
***
****
**
DMSO
10
20
40
Cyto D (μM)
AG-08
D
Cell Viability (Fold Change)
1.2
1
0.8
0.6
0.4
0.2
0
DMSO
40
80
100
Dynasore (μM)
AG-08

E

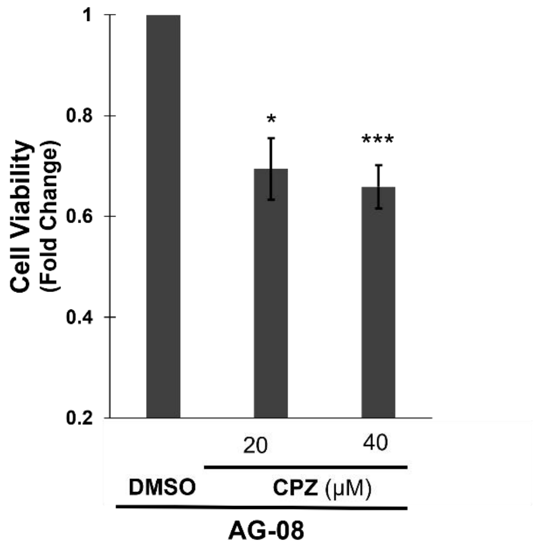

F

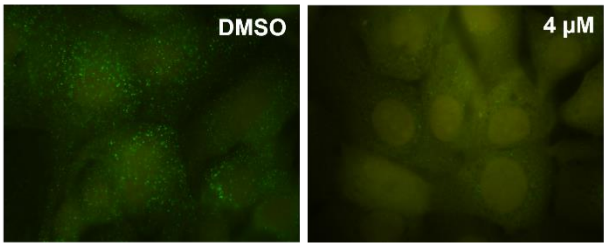

Supplementary Figure 1. AG-08 particles affect endosomal pathway (A-E) HCC1937 cells were pre-treated with different concentration of pitstop II, MβC, dynasore, Cyto D or CPZ for 1 h and 8 μM AG-08 for 24 h. Reported values was normalized on cells treated with only AG-08. Error bars are the standard deviations (n=3). p-values were calculated with respect to AG-08 treated cells by two-tailed equal variance Student's t-test (*p<0.05, **p<0.005, ***p<0.001, ****p<0.0001). (F) Following 4 μM AG-08 treatment for 40 h, EEA1 proteins of HCC1937 cells were stained using anti-EEA1 antibody

Supplementary Table 1. Gene expression level, p value and FDR p value of AG-08 and control.

Gene Symbol	AG-08 (Log2)	Control (Log2)	P-val	FDR P-val

IL1RL1	10.06	6.82	0.013	0.8393
CXCL8	12.86	9.63	8.63E-06	0.2667
CCL20	15.79	12.58	6.31E-07	0.0857
DDIT3	8.56	5.64	2.22E-05	0.335
TNFAIP3	15.77	12.92	0.0009	0.8393
CREB5	11.14	8.35	0.0181	0.8393
AKAP12	9.04	6.27	0.0122	0.8393
CHAC1	9.98	7.22	0.0002	0.8393
IL1A	12.9	10.2	1.96E-06	0.1329
CXCL3	9.67	6.99	0.0152	0.8393
IL6	11.97	9.32	0.0021	0.8393
choygey	11.85	9.26	0.0009	0.8393
TNFAIP6	10.32	7.73	2.91E-05	0.3589
cheeju	4.53	7.11	0.0003	0.8393
AC068797.1	4.17	6.72	0.0038	0.8393
plergler	5.85	8.38	0.0083	0.8393
HSPA7	7.45	4.94	2.82E-05	0.3589
snawperbu	8.51	10.99	0.005	0.8393
CXCL2	10.58	8.19	0.0125	0.8393
FGF5	6.46	4.09	0.0036	0.8393
GADD45A	13.43	11.09	0.0001	0.8393
DAW1	7.07	4.73	0.0045	0.8393
dymabu	4.94	7.28	0.0002	0.8393
sworkaby	4.69	2.37	0.0123	0.8393
swawchu	10.51	12.82	0.0272	0.8393
FRMD6	9.43	7.12	0.0042	0.8393
sparsloy	7.2	9.51	0.0006	0.8393
HSP90B1	10.67	8.38	0.0007	0.8393
KRT5	9.49	11.75	0.0017	0.8393
flyspabu	4.45	6.68	0.0001	0.8393
HSPA1A	15.75	13.53	9.73E-05	0.8393
NR4A1	8.84	6.63	0.0295	0.8393
MIR6515	6.96	4.75	0.0009	0.8393
PTGS2	11.9	9.7	0.0005	0.8393
DNAJB9	10.33	8.13	5.34E-05	0.558

kluky	4.18	6.37	0.0058	0.8393
SPRR2A	8.31	6.12	0.0024	0.8393
steybleebu	7.5	9.69	0.0032	0.8393
slukeebu	5.7	7.87	0.0385	0.8393
chostarby	6.86	9.01	0.0058	0.8393
borflaw	6.24	4.1	0.0471	0.8393
vyglabu	4.99	2.86	0.0099	0.8393
SPRY4	6.6	4.5	0.0146	0.8393
RNA18S5	12.08	10	0.0034	0.8393
sportobo	9.07	11.14	0.0166	0.8393
MAP1B	9.32	7.26	0.004	0.8393
AC079949.1	7.8	5.75	0.0038	0.8393
floynabo	5.07	3.01	0.0245	0.8393
jawrubu	4.94	6.99	0.0289	0.8393
runara	4.96	7	0.0265	0.8393
RP11-253I19.3	3.7	5.74	0.0188	0.8393
RCAN1	13.43	11.4	0.0013	0.8393
HERPUD1	15.89	13.87	0.0002	0.8393
AL161626.1	4.86	2.85	0.0021	0.8393
rersharbu	6.82	8.82	0.0083	0.8393
GEM	6.45	4.45	0.0079	0.8393
RP11-74J13.9	3.93	5.92	0.0026	0.8393
beyly	3.88	5.86	0.0105	0.8393
RP5-1180E21.5	7.94	9.91	0.0066	0.8393
MIR1284	3.95	5.93	1.51E-05	0.335
SERPINE1	9.03	7.07	0.0079	0.8393
ferchor	4.51	6.47	0.0031	0.8393
sneykleebu	5.94	7.89	0.02	0.8393
ETS1	13.17	11.21	0.0054	0.8393
RNU11-6P	3.79	5.74	0.0073	0.8393
bonawbu	4.47	6.42	0.0096	0.8393
AC006548.19	4.15	6.1	0.0083	0.8393
keyfybu	4.42	6.35	0.0053	0.8393
ERO1B	8.83	6.9	0.0032	0.8393
hunimo	4.9	2.97	0.0261	0.8393

flyleyby	3.87	5.79	0.0017	0.8393
CSF3	5.21	3.29	0.0068	0.8393
Y_RNA	4.88	2.96	0.0148	0.8393
RP11-632K20.8	6.86	8.77	0.001	0.8393
nerure	6.39	8.3	0.0013	0.8393
temire	3.91	5.81	0.0215	0.8393
RP5-890E16.5	3.64	5.54	0.0346	0.8393
METTL7A	12.34	14.23	0.0073	0.8393
mawry	4.91	6.8	0.0007	0.8393
DUSP1	12.84	10.95	0.0014	0.8393
chergy	6.6	8.48	0.0057	0.8393
spaforby	8.45	10.31	0.0004	0.8393
skoytoyby	5.73	3.86	0.0028	0.8393
leeklerbu	2.34	4.21	0.0158	0.8393
spernawbo	5.32	7.18	0.0061	0.8393
skaskoyby	6.69	8.54	0.0084	0.8393
mazybu	3.22	5.07	0.0143	0.8393
swyjee	6.02	7.88	0.0353	0.8393
ATF3	7.04	5.19	0.0003	0.8393
teesnarby	4.07	5.92	0.0064	0.8393
SIRPG-AS1	4.97	6.81	0.0181	0.8393
myklar	4.37	6.22	0.0223	0.8393
TNFRSF9	8.07	6.22	2.00E-05	0.335
slorfley	3.4	5.24	0.0038	0.8393
JUN	12.32	10.47	0.0028	0.8393
glupuby	6.47	8.3	0.0118	0.8393
muneme	3.26	5.09	0.0154	0.8393
LRRC49	8.59	6.77	4.06E-05	0.4595
CSRNP1	7.09	5.26	0.0052	0.8393
guzobu	3.12	4.94	0.0124	0.8393
skeymeybo	5.88	4.08	0.0442	0.8393
RN7SKP36	6.69	4.9	0.0009	0.8393
vybu	4.27	2.47	0.0043	0.8393
ACSM3	4.97	6.76	0.0089	0.8393
klobly	6.7	8.49	0.0015	0.8393

doymabu	4.76	6.54	0.0007	0.8393
LOC100128914	4.93	3.15	0.044	0.8393
IFI44	12.67	14.45	0.0489	0.8393
cherstarby	6.42	8.19	0.0067	0.8393
hosimu	5.79	4.01	0.0018	0.8393
kosey	5.44	3.66	0.0124	0.8393
riyare	3.31	5.08	0.0374	0.8393
skopoybo	4.26	6.02	0.0001	0.8393
FP671120.3	8.3	10.07	0.0051	0.8393
FP236383.2	8.3	10.07	0.0051	0.8393
AL353644.7	8.3	10.07	0.0051	0.8393
AL592188.5	8.3	10.07	0.0051	0.8393
luwarbu	3.42	5.19	0.007	0.8393
goylobo	6.56	8.33	0.0304	0.8393
wardaw	3.08	4.84	0.0067	0.8393
starblaw	4.63	6.39	0.0077	0.8393
blerverby	4.88	6.64	0.0187	0.8393
RP4-802A10.1	8.32	10.07	0.019	0.8393
gamabo	4.18	5.94	0.0266	0.8393
nureebo	8.33	10.08	0.0112	0.8393
blaployby	2.32	4.06	0.0022	0.8393
sworoy	3.06	4.81	0.0069	0.8393
sharjer	4.32	6.06	0.0003	0.8393
STC2	12.61	10.87	0.0002	0.8393
tunemo	7.64	5.91	0.0102	0.8393
neyzubu	4.44	6.17	0.0008	0.8393
kloloy	3.91	2.19	0.0138	0.8393
CLCA2	5.92	7.65	0.0007	0.8393
barjoybu	4.66	6.38	0.0142	0.8393
stuloybu	7.4	9.12	0.0029	0.8393
rekare	4.03	5.75	0.0368	0.8393
AL158839.1	5.7	3.98	0.0059	0.8393
blonawbo	5.21	6.92	0.0269	0.8393
harero	5.11	6.81	0.0002	0.8393
AL137800.1	4.33	6.03	0.0048	0.8393

LRRC8C	11.05	9.35	0.0075	0.8393
GLIPR1	12.79	11.09	0.0281	0.8393
wosnawby	7.09	8.78	0.0147	0.8393
snawspuby	7.99	9.68	0.0347	0.8393
SNORD2	5.29	3.6	0.003	0.8393
plarbey	5.68	3.98	0.0165	0.8393
klawlaw	6.35	8.03	0.0089	0.8393
shagly	8.29	9.98	0.0091	0.8393
rusame	4.71	6.4	0.0002	0.8393
bleymawby	5.86	7.54	0.0016	0.8393
AC007272.3	4.36	6.04	0.0077	0.8393
RABEPK	8.03	6.35	0.0325	0.8393
skoytyby	6.43	8.1	0.0002	0.8393
cherfoy	10.56	12.23	0.0052	0.8393
BAG3	15.02	13.35	0.0294	0.8393
AC010139.1	12.83	11.17	0.0222	0.8393
UAP1	15.84	14.17	0.0349	0.8393
ALDH3A1	10.37	12.03	0.0004	0.8393
vamo	6.72	8.38	0.0034	0.8393
ferraw	6.36	8.02	0.0316	0.8393
klorpla	9.12	10.78	0.044	0.8393
HSPA1B	14	12.34	0.0008	0.8393
terbabu	5.12	3.46	0.0044	0.8393
RPL17P28	6.52	8.17	0.0065	0.8393
HYOU1	12.17	10.52	0.0003	0.8393
RP11-325K4.3	9.91	8.27	0.0028	0.8393
geeraw	7.51	9.14	0.0041	0.8393
jernobu	5	6.63	0.0067	0.8393
RP11-187C18.2	6.09	7.72	0.0393	0.8393
AC110813.1	9.52	11.14	0.0105	0.8393
sharflu	4.05	5.67	0.001	0.8393
weymubu	3.7	5.32	0.0084	0.8393
steynarbu	5.53	7.14	0.0297	0.8393
MSANTD3-TMEFF1	9.84	8.22	0.0398	0.8393
chershee	7.19	8.8	0.0058	0.8393

LOC646762	9.37	10.98	0.0136	0.8393
snarnerbu	4.83	6.43	0.0471	0.8393
SGPP2	7.81	6.2	0.0152	0.8393
GPX2	6.23	7.83	0.0012	0.8393
tosuru	5.42	7.02	0.004	0.8393
tawsweebu	7.72	9.32	0.0105	0.8393
KLRC3	3.09	4.68	0.0062	0.8393
PLD1	6.82	8.42	0.0108	0.8393
plerder	3.65	5.24	0.0114	0.8393
sterda	5.92	7.51	0.0045	0.8393
varger	6.16	7.75	0.0056	0.8393
nygleeby	3.73	5.31	0.0215	0.8393
AC008391.1	13.25	11.66	0.0175	0.8393
SMUG1	6.89	8.48	0.0037	0.8393
HSPD1P11	5.88	7.47	0.006	0.8393
RP4-620F22.2	4.44	2.85	0.0459	0.8393
HSPA6	7.79	3.65	3.84E-06	0.1738
garskeyby	9.6	16.43	0.0483	0.8393

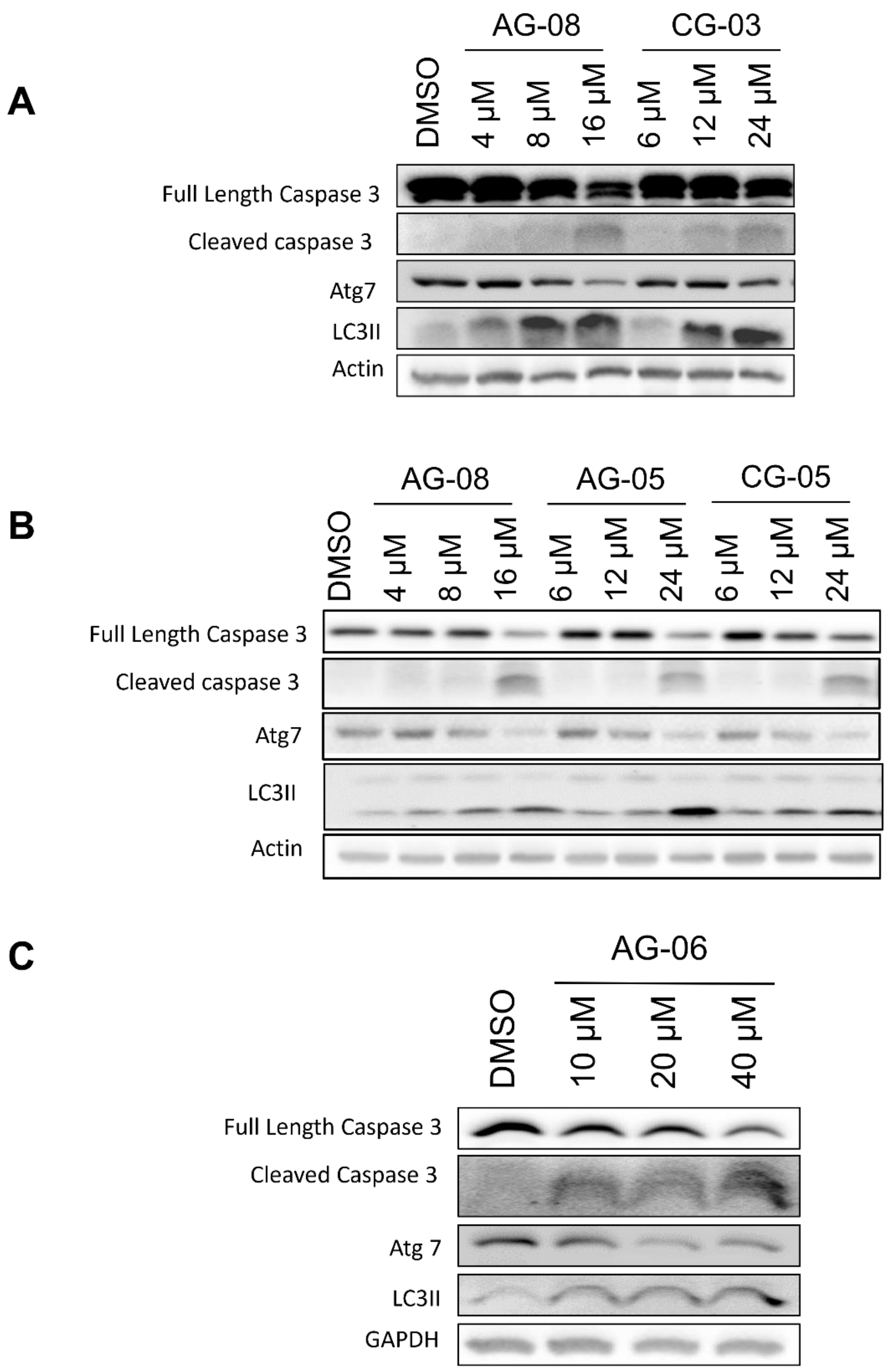

Supplementary Figure 2. Cytotoxic compounds similarly affect LC3-II, caspase 3 and Atg7 proteins. HCC1937 cells with cytotoxic compounds or vehicle (DMSO). The levels of LC3II, Atg-7, caspase 3 and cleaved caspase 3 were detected by immunoblotting using antibodies against them. β-Actin and GAPDH were used as the loading control.

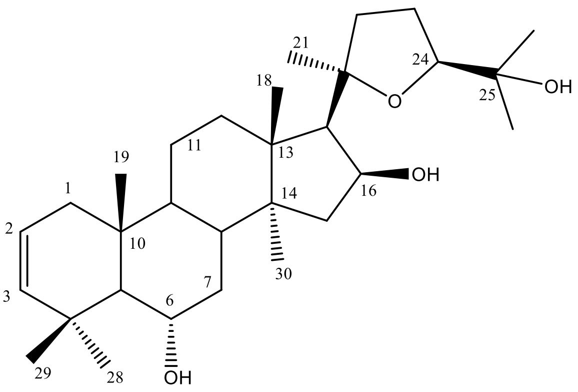

Supplementary Figure 3. Chemical Structure of AG-02

Supplementary Table 2. The ^{13}C and ^{1}H NMR data of AG-02 (100/400 MHz, δ ppm, in $CDCl_3$)

**H/C**	**δ_C (ppm)**	**δ_H (ppm), *J* (Hz)**
**1**	37.9 t	2.04 m, 2.11 m
**2**	120.3 d	5.5 ddd (10.1, 5.4, 3)
**3**	139.6 d	5.31 m
**4**	36 s	-
**5**	55.4 d	1.28 m
**6**	70.2 d	4.06 ddd (10.8, 10.8, 3.9)
**7**	38.6 t	1.44 m, 1.86 m
**8**	40.9 d	2.4 m
**9**	145.8 s	-
**10**	40.3 s	-
**11**	116.3 d	5.3 m
**12**	37.8 t	1.89 m, 2.14 m
**13**	44.3 s	-
**14**	43.9 s	-
**15**	45.1 t	1.51 dd (12.8, 6.2), 2.05 m
**16**	73.4 d	4.72 ddd (7.9, 7.9, 6.3)
**17**	56.2 d	2.36 d (7.8)
**18**	18.1 q	0.94 s
**19**	23.75 q	1.05 s
**20**	87.2 s	-

**21**	28 q	1.23 s
**22**	34.6 t	1.6 m, 2.59 q (10.4)
**23**	25.9 t	2 m
**24**	81.51 d	3.75 dd (8.1, 6.2)
**25**	72 s	-
**26**	26.7 q	1.14 s
**27**	27.81 q	1.3 s
**28**	34.9	1.2 s
**29**	23.0 q	1.17 s
**30**	19.2 q	0.79 s

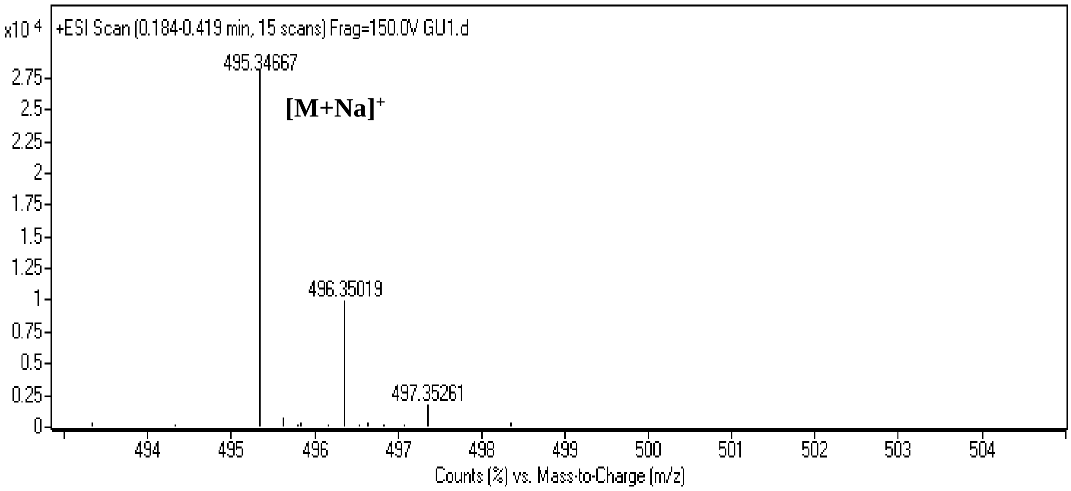

Spectrum 1. HR-ESI-MS Spectrum of AG-02 (positive mode)

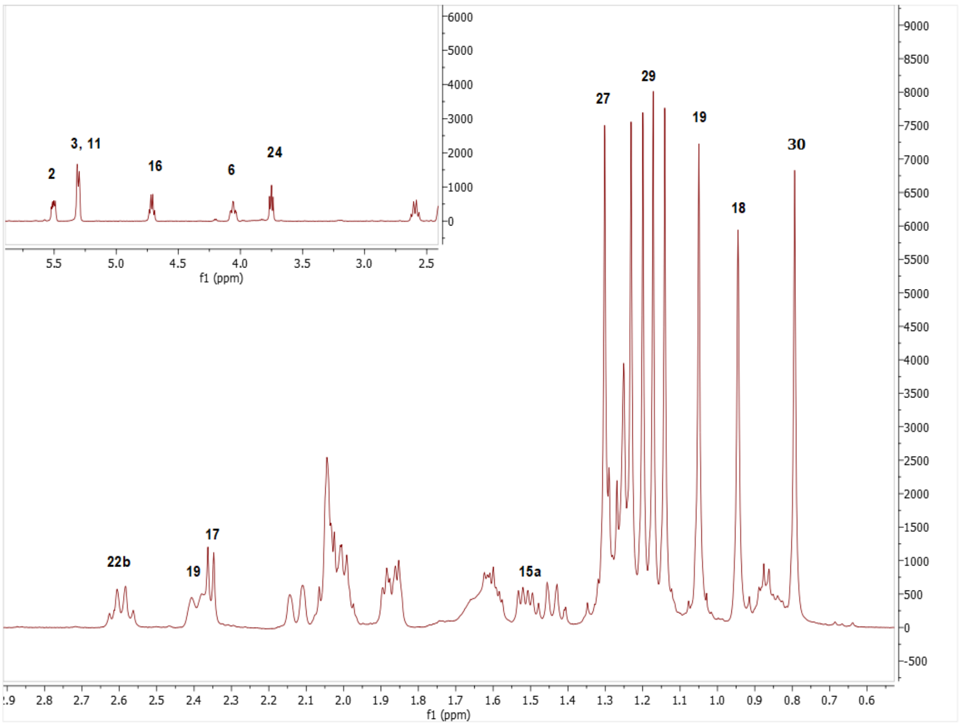

Spectrum 2. ^{1}H NMR Spectrum of AG-02.

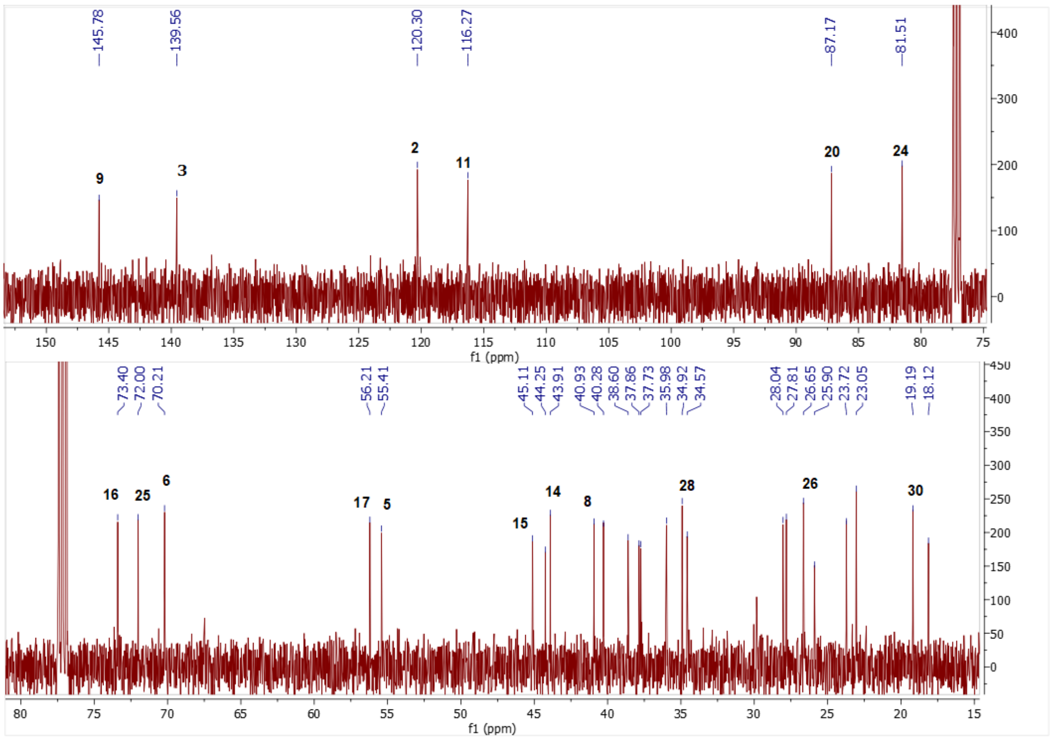

Spectrum 3. ^{13}C NMR Spectrum of AG-02.

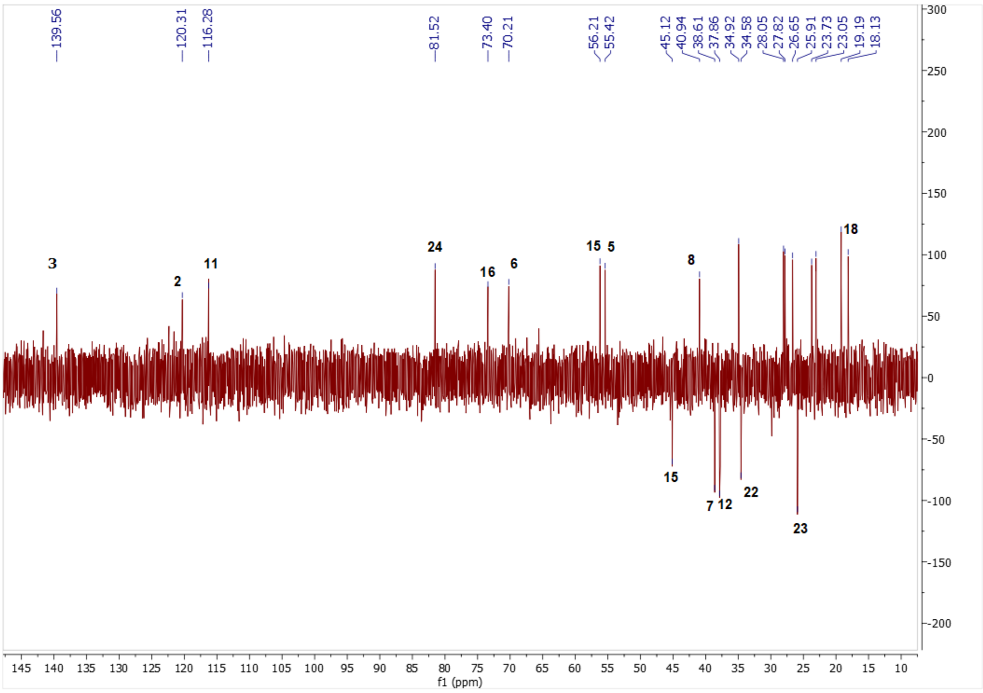

Spectrum 4. DEPT135 spectrum of AG-02

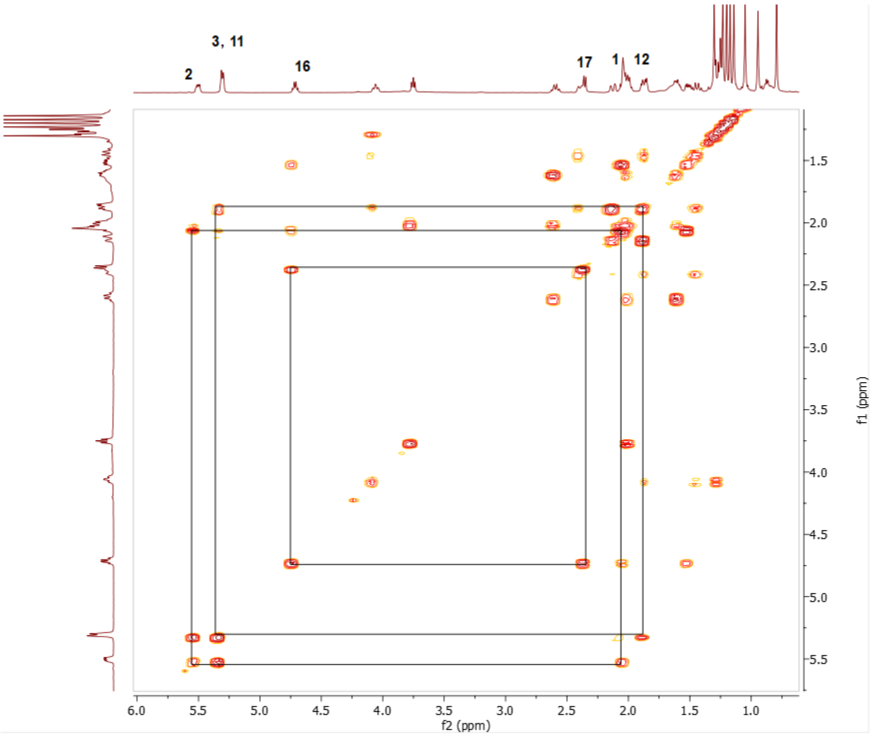

Spectrum 5. COSY spectrum of AG-02

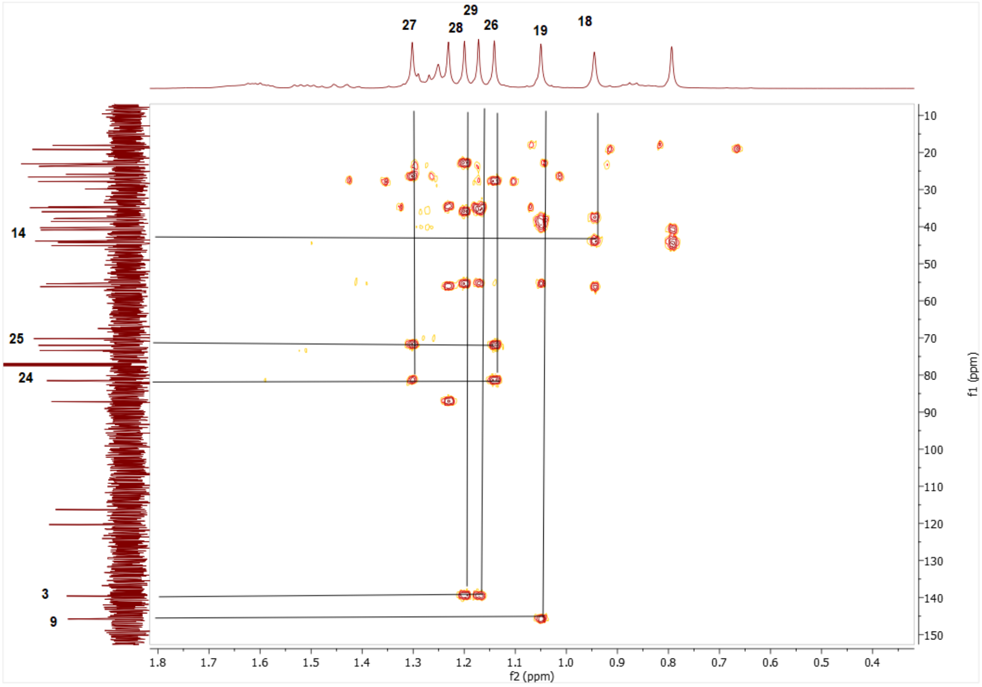

Spectrum 6. HMQC spectrum of AG-02.

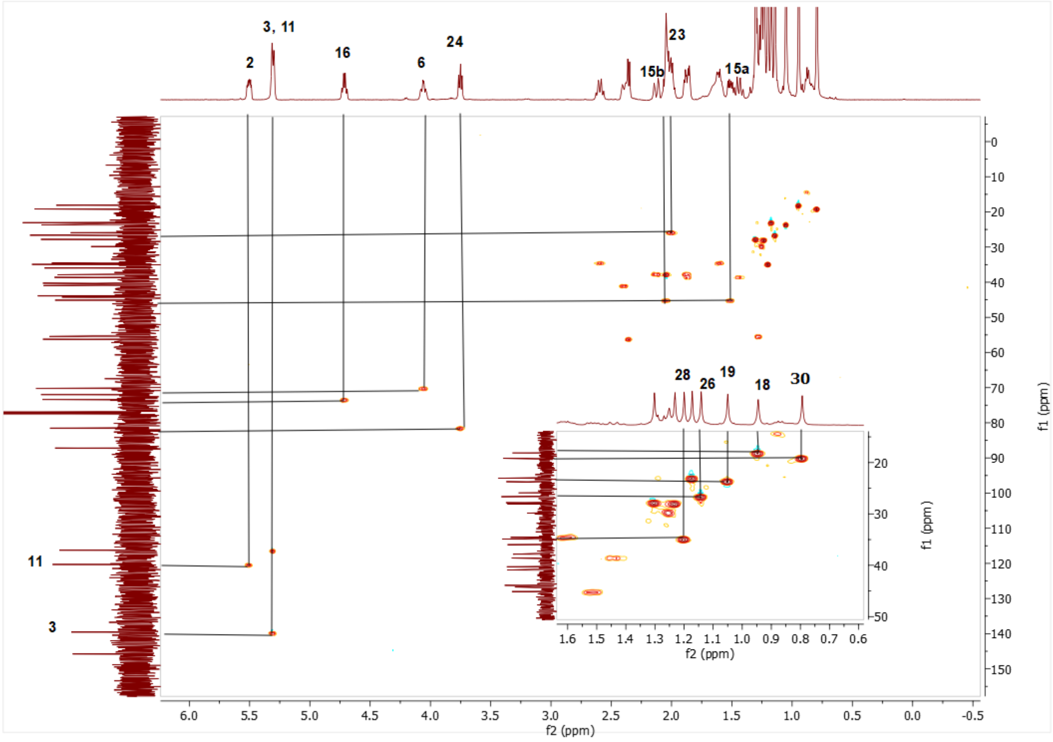

Spectrum 7. HMBC spectrum of AG-02.

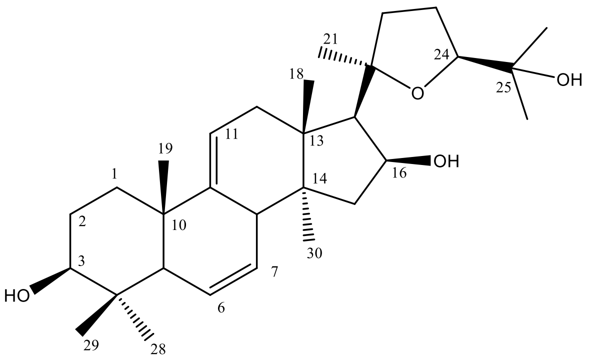

Supplementary Figure 4. Chemical Structure of AG-03

Supplementary Table 3. The ^{13}C and ^{1}H NMR data of AG-03 (100/400 MHz, δ ppm, in $CDCl_3$).

**H/C**	**δ_C (ppm)**	**δ_H (ppm), *J* (Hz)**
**1**	34.4 t	1.59 m
**2**	28.2 t	1.78 m
**3**	79.3 d	3.23 dd (11.8, 4.8)
**4**	38.8 s	-
**5**	52.2 d	1.70 d (12.2)
**6**	127.3 d	5.71 m
**7**	128.9 d	5.57 dt (10.2, 3.2)
**8**	43.9 d	2.84 brs
**9**	145.9 s	-
**10**	39.2 s	-
**11**	113.6 d	5.16 brs
**12**	38.2 t	2.04 m, 1.92 m
**13**	43.8 s	-
**14**	44.8 s	-
**15**	44.3 t	2.07 m, 1.57 m
**16**	73.6 d	4.69 m
**17**	56.6 d	2.24 dd (11.8, 6.1)
**18**	19.1 q	0.98 s
**19**	20.4 q	1.01
**20**	87.3 s	-
**21**	28.2 d	1.23 s
**22**	34.8 d	1.58 m, 2.56 m
**23**	26.1 t	2 m
**24**	81.8 d	3.75 t (7.2)
**25**	72.2 s	-
**26**	28.2 q	1.29 s
**27**	26.9 q	1.14 s
**28**	28.2 q	1.01 s
**29**	16 q	0.83 s
**30**	18.91	0.64 s

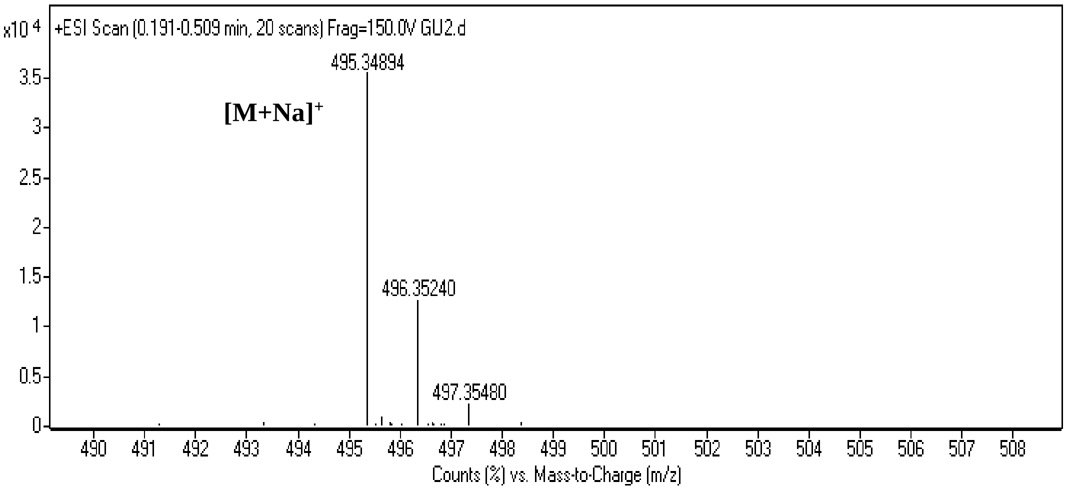

Spectrum 8. HR-ESI-MS Spectrum of AG-03 (positive mode).

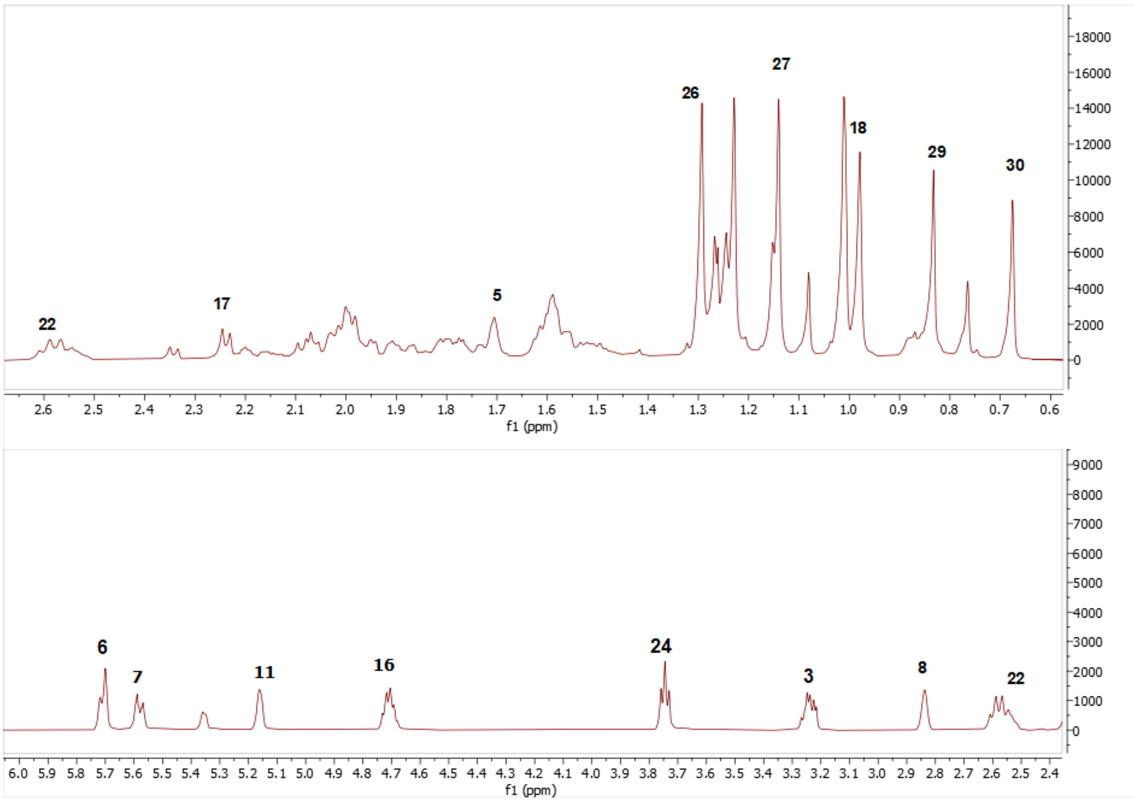

Spectrum 9. 1H NMR Spectrum of AG-03

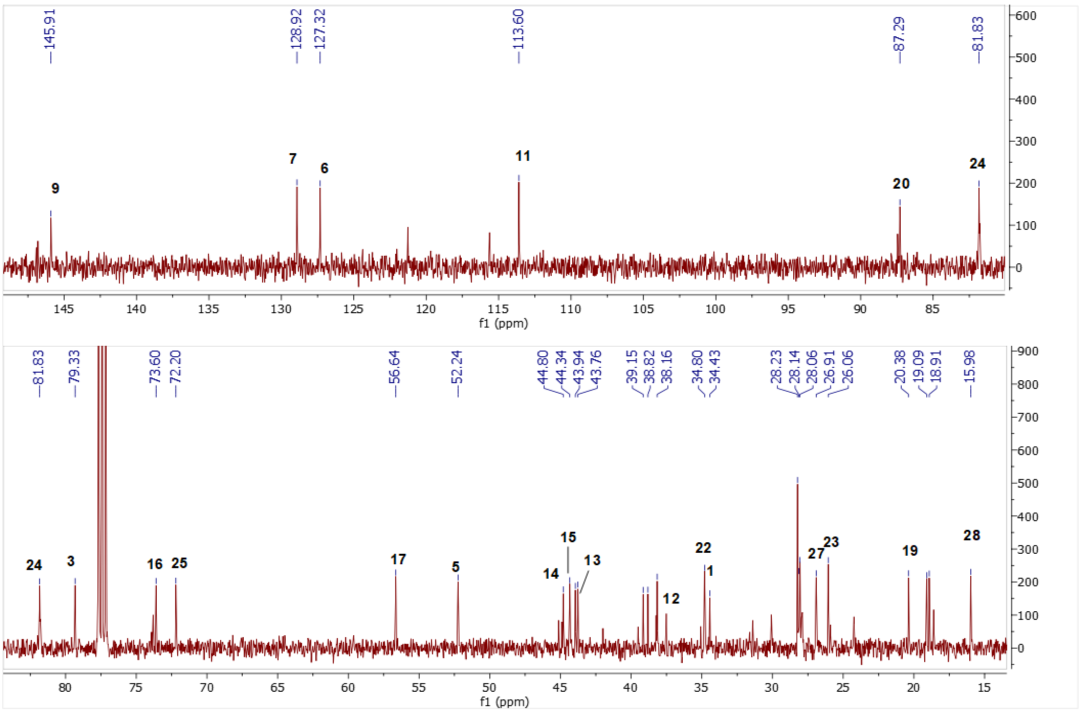

Spectrum 10. ^{13}C NMR Spectrum of AG-03.

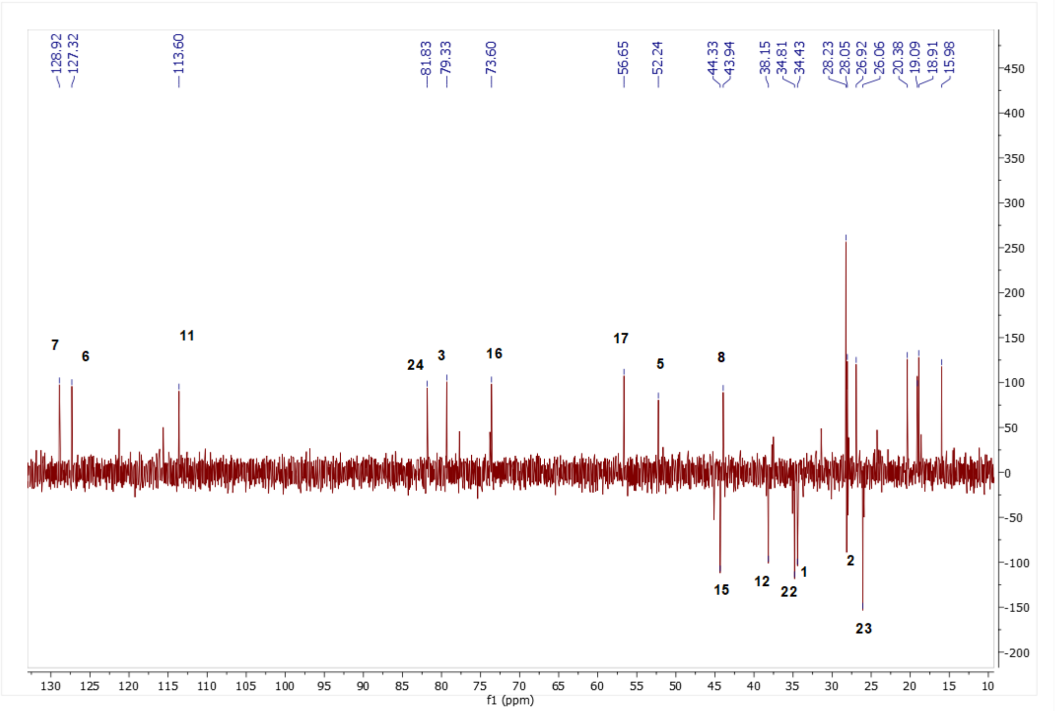

Spectrum 11. DEPT135 spectrum of AG-03.

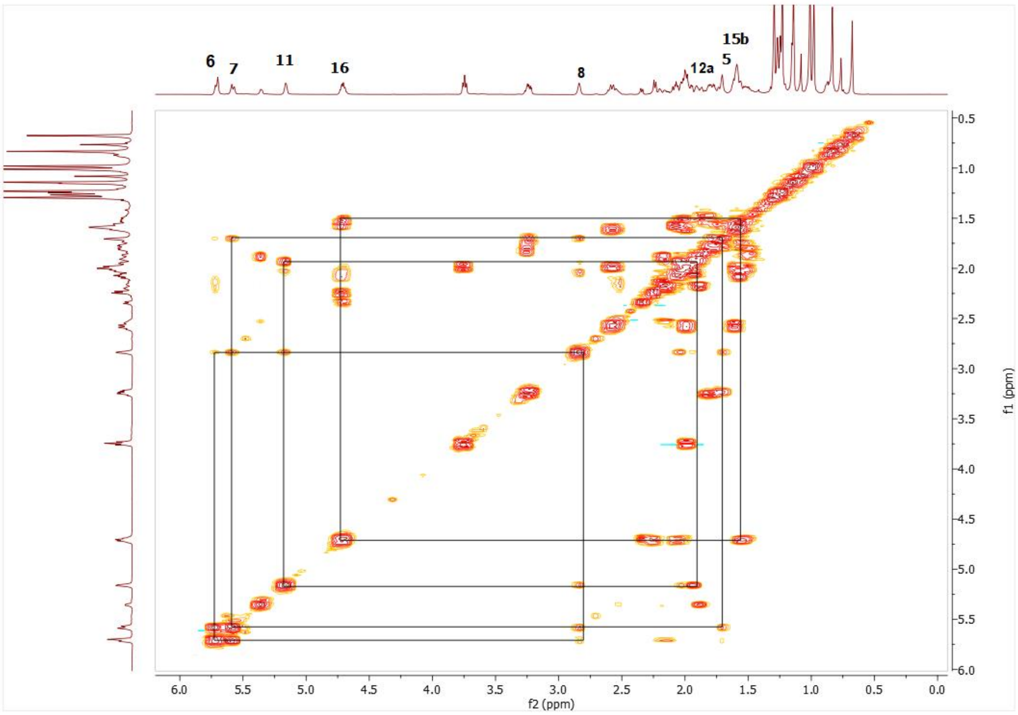

Spectrum 12. COSY spectrum of AG-03.

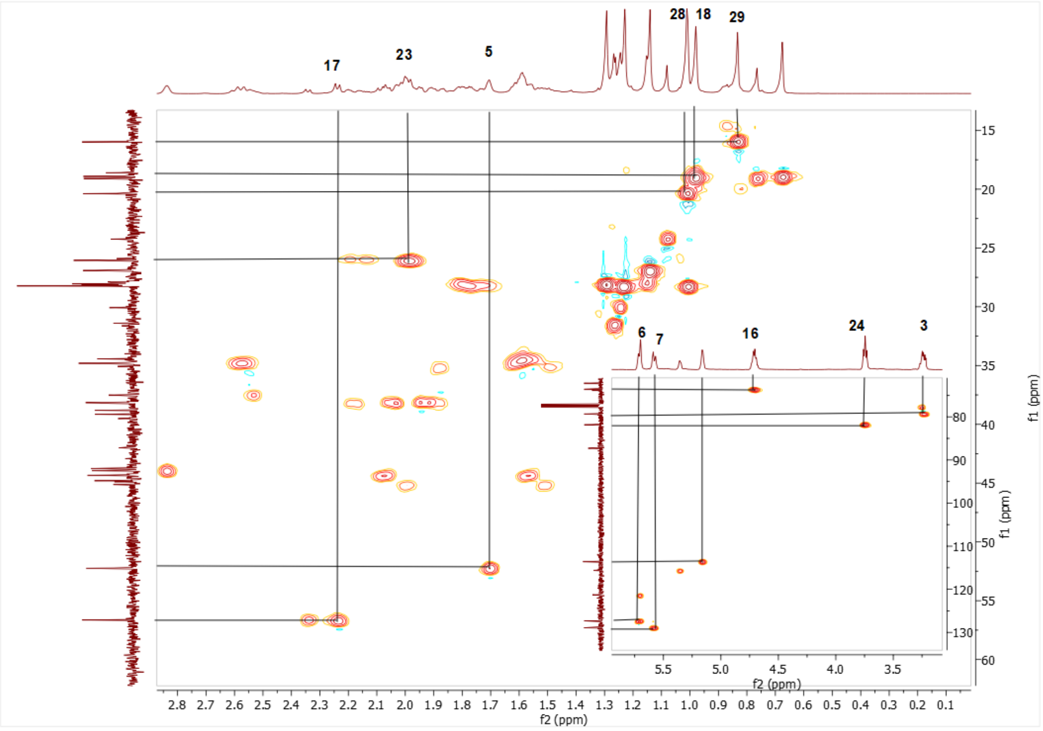

Spectrum 13. HMQC spectrum of AG-03

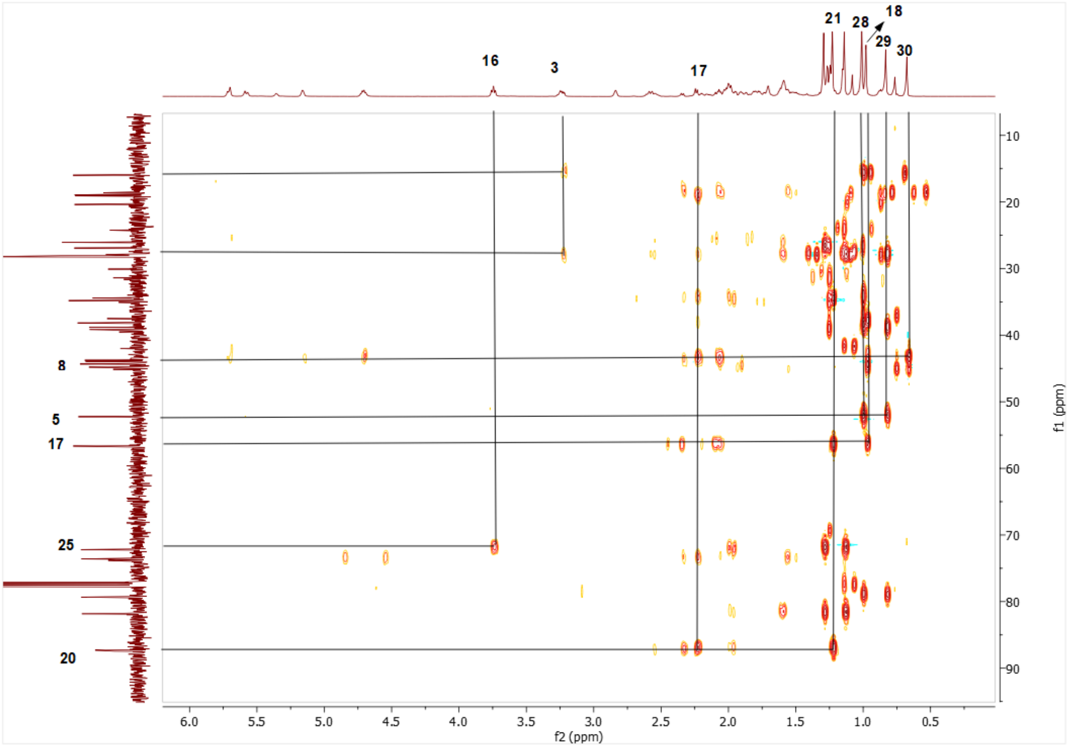

Spectrum 14. HMBC spectrum of AG-03.

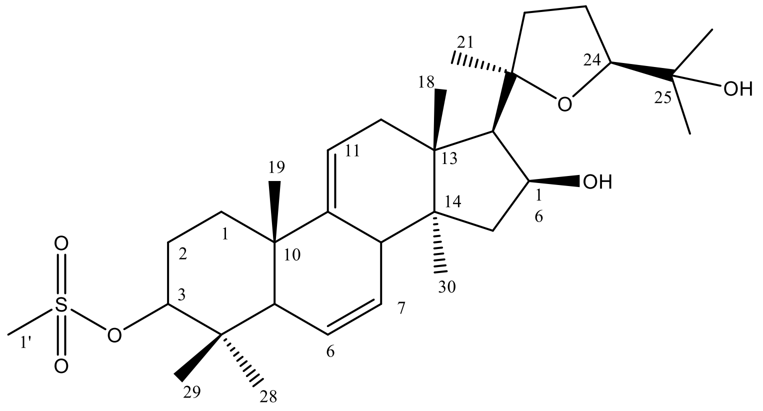

Supplementary Figure 5. Chemical Structure of AG-04

Supplementary Table 4. The ^{13}C and ^{1}H NMR data of AG-04 (100/400 MHz, δ ppm, in CDCl3).

**H/C**	**δ_C (ppm)**	**δ_H (ppm), *J* (Hz)**
**1**	44.0 t	1.65 m
**2**	25.74 t	2.06 m, 2.2 m
**3**	90.2 d	4.35 dd (11.6, 4.9)
**4**	38.6 s	-
**5**	52.2 d	1.81 brs
**6**	126.1 d	5.67 dt (10.1, 2.1)
**7**	128.4 d	5.63 m
**8**	43.7 d	2.85 brs
**9**	144.8 s	-
**10**	38.3 s	-
**11**	113.9 d	5.17 dt (5.3, 2.5)
**12**	37.9 t	1.96 m, 2.05 m
**13**	44.6 s	-
**14**	43.5 s	-
**15**	44.0 t	1.57 m, 2.08 m
**16**	73.3 d	4.71 ddd (7.7, 7.7, 5.9)
**17**	56.4 d	2.24 d (7.6)
**18**	18.7 q	0.67 s
**19**	20.1 q	1.05 s
**20**	87.0 s	-
**21**	27.9 q	1.24 s
**22**	34.6 t	1.64 m, 2.57 q (10.6)
**23**	25.81 t	2.0 m
**24**	81.6 d	3.76 t (7.2)
**25**	72 s	-
**26**	27.8 q	1.3 s
**27**	26.7 q	1.15 s
**28**	28.1 q	1.05 s
**29**	16.5 q	0.92 s
**30**	18.84 q	0.98 s
**1'**	39 q	3.03 s

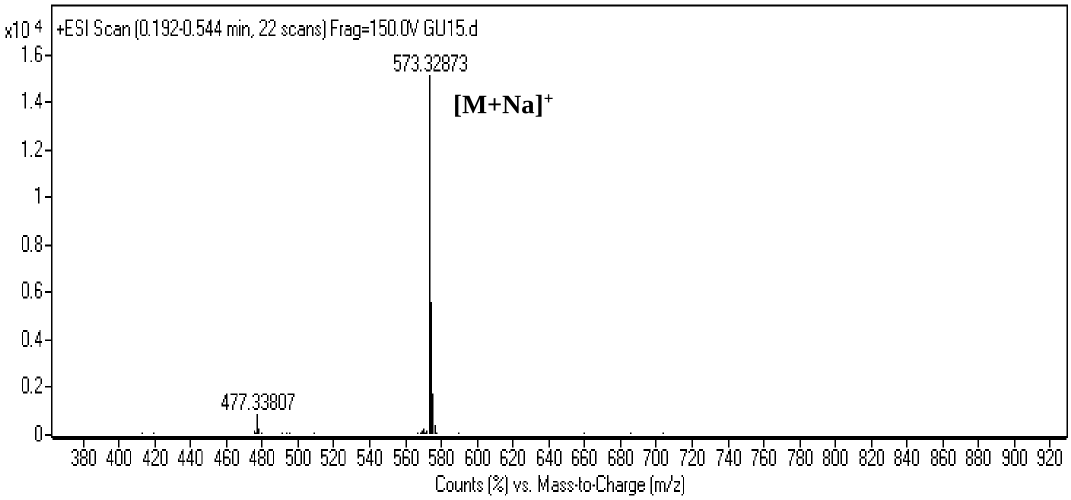

Spectrum 15. HR-ESI-MS Spectrum of AG-04 (positive mode).

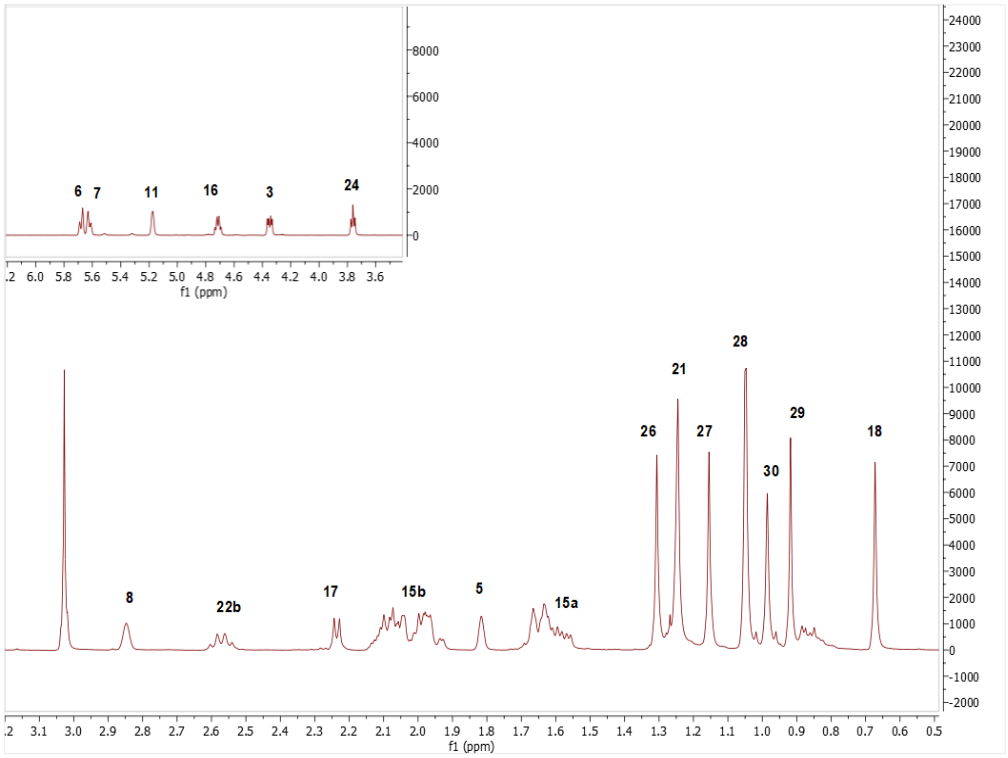

Spectrum 16. ^{1}H NMR Spectrum of AG-04.

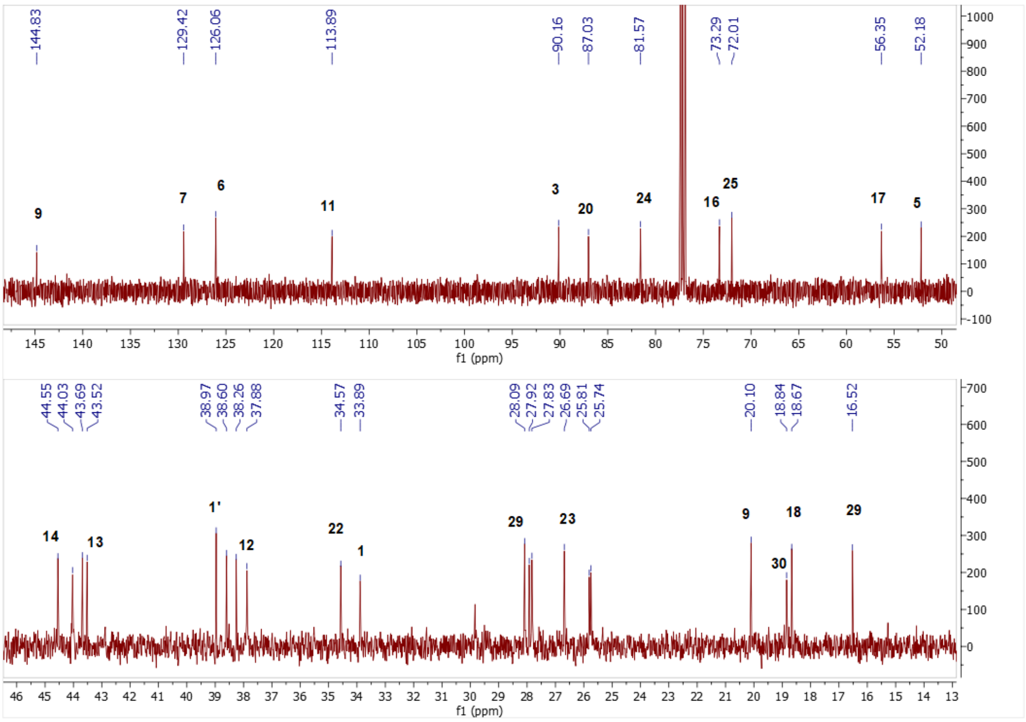

Spectrum 17. ^{13}C NMR Spectrum of AG-04.

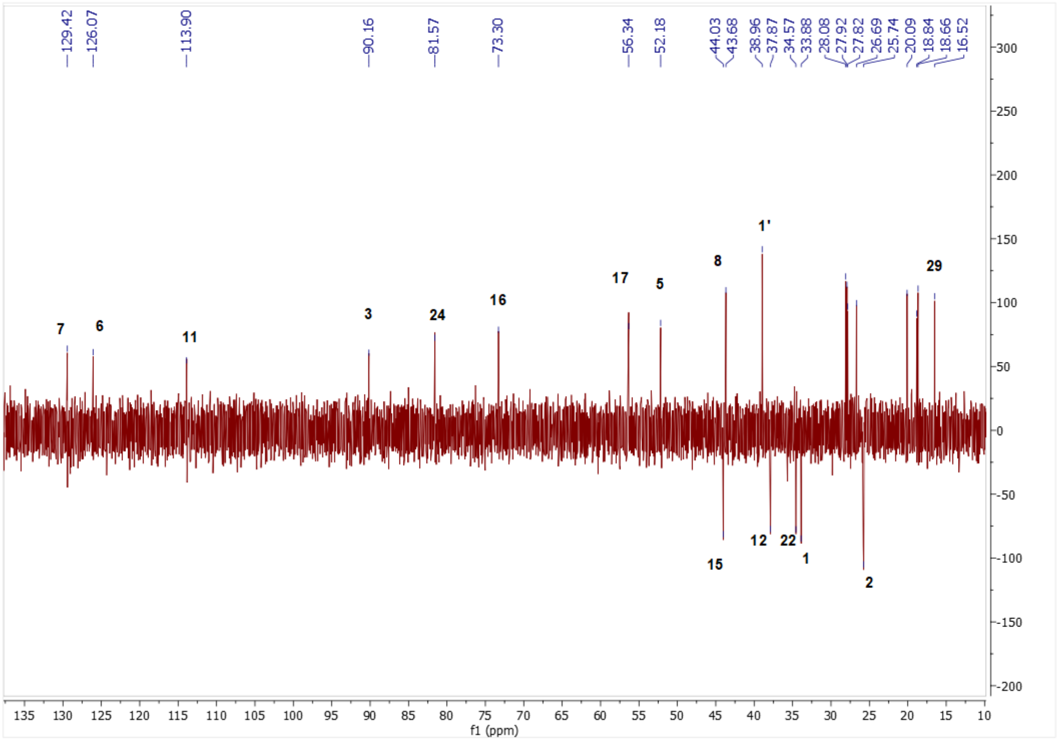

Spectrum 18. DEPT135 spectrum of AG-04

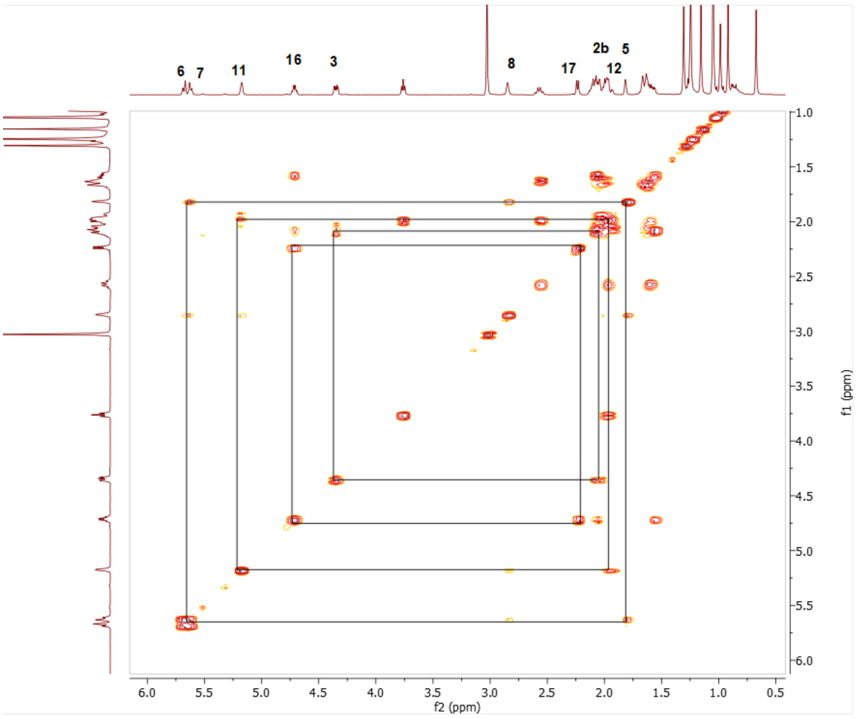

Spectrum 19. COSY spectrum of AG-04.

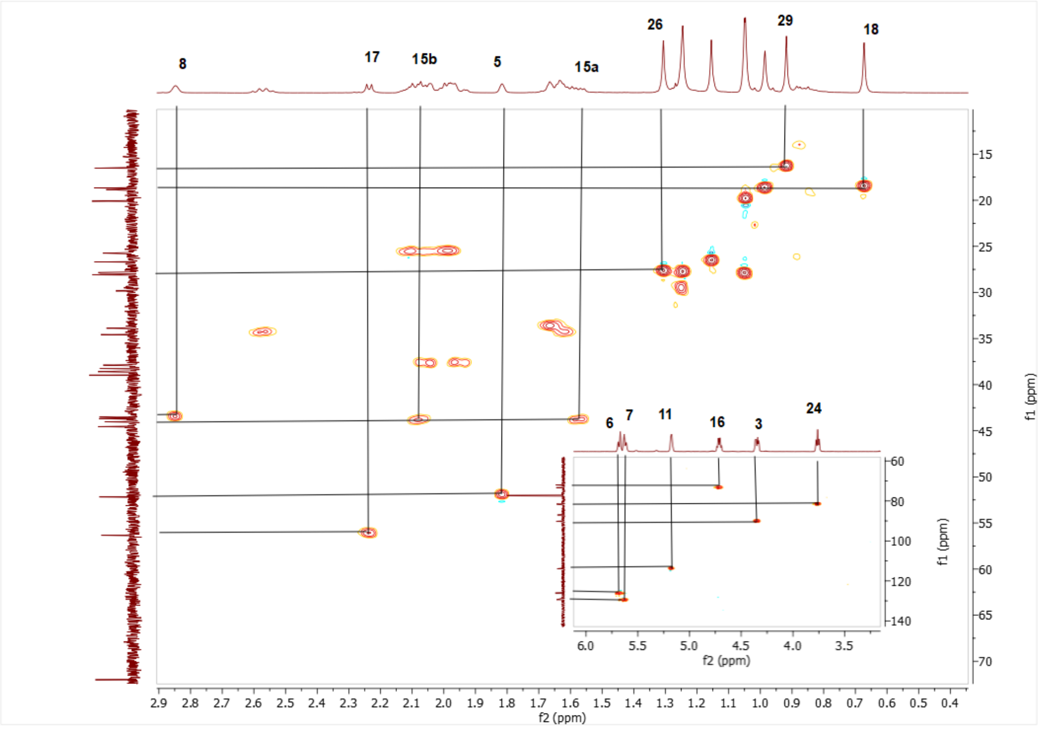

Spectrum 20. HMQC spectrum of AG-04

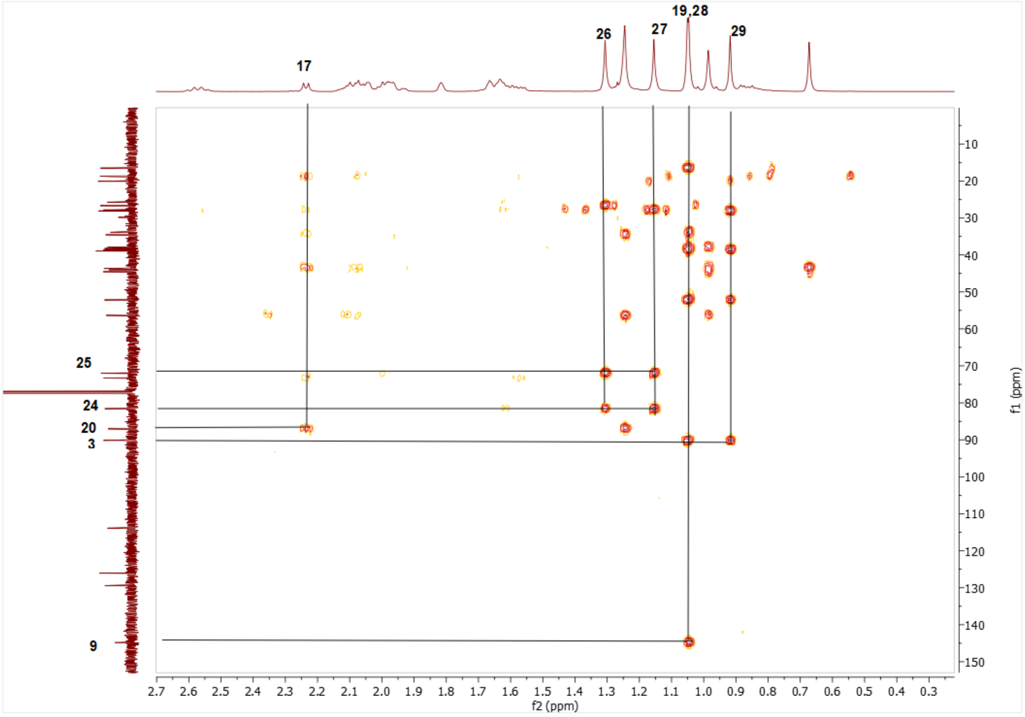

Spectrum 21. HMBC spectrum of AG-04

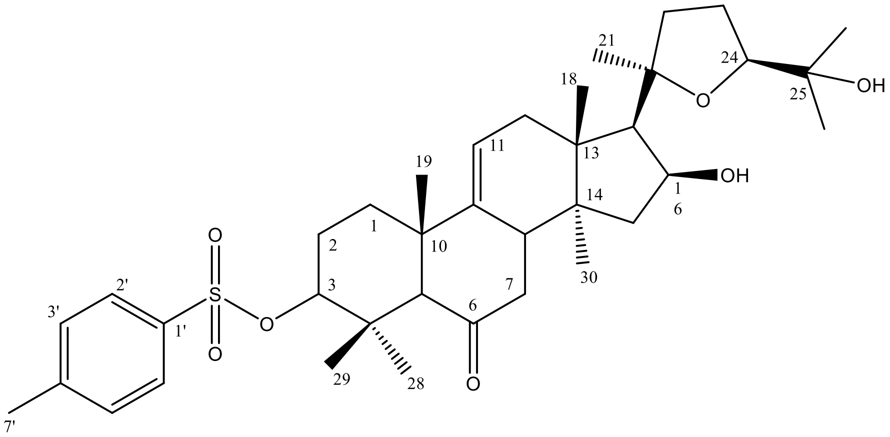

Supplementary Figure 6. Chemical Structure of AG-05

Supplementary Table 5. The ^{13}C and ^{1}H NMR data of AG-05 (100/400 MHz, δ ppm, in CDCl3).

H/C	δ_C (ppm)	δ_H (ppm), *J* (Hz)
**1**	35.9 t	1.68 m, 1.85 d (1,5)
**2**	25.1 t	1.91 m
**3**	89.4 d	4.07 m
**4**	37.7 s	-
**5**	61.7 d	2.17 brs
**6**	210.4 s	-
**7**	43.98 t	2.19 m, 2.34 d (5)
**8**	43.8 d	2.69 brs
**9**	144.5 s	-
**10**	44.3 s	-

**11**	118.1 d	5.44 dd (6.3, 1.7)
**12**	44.2 t	1.43 dd (12.9, 6.3), 1.88 d (4.24)
**13**	44.2 s	-
**14**	44.3 s	-
**15**	37.4 t	1.95 m, 2.15 m
**16**	73.0 d	4.68 q (6.8)
**17**	56.3 d	2.32 brs
**18**	18.2 q	0.9 s
**19**	23.8 q	1.04 s
**20**	86.9 s	-
**21**	28.0 q	1.22 s
**22**	34.5 t	1.56 dt (12,6), 2.56 q (10.4)
**23**	25.8 d	1,96 m
**24**	81.4 d	3.75 td (7.4, 1.6)
**25**	72.0 s	-
**26**	26.8 q	1.14 s
**27**	27.8 q	1.29 s
**28**	27.8 q	0.84 s
**29**	16.1 q	1.27 d (1.3)
**30**	18.9 q	0.84 s
**1’**	134.6 s	-
**2’**	127.8 d	7.77 dd (8.2, 1.7)
**3’**	129.8 d	7.32 dd (8.4, 1.8)
**4’**	144.6 s	-
**5’**	129.8 d	7.32 dd (8.4, 1.8)
**6’**	127.8 d	7.77 dd (8.2, 1.7)
**7’**	21.78 q	2.43 s

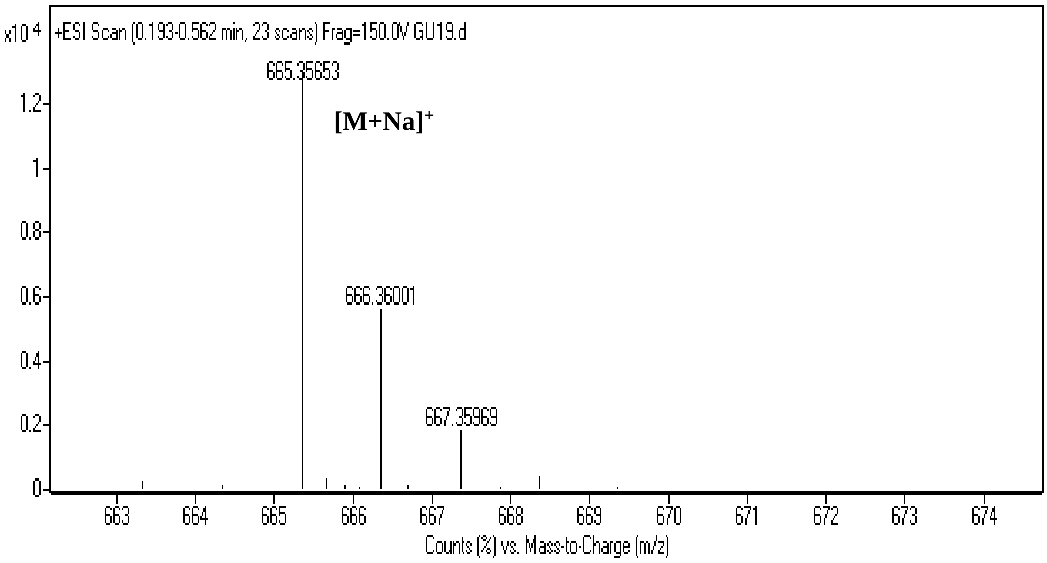

Spectrum 22. HR-ESI-MS Spectrum of AG-05 (positive mode).

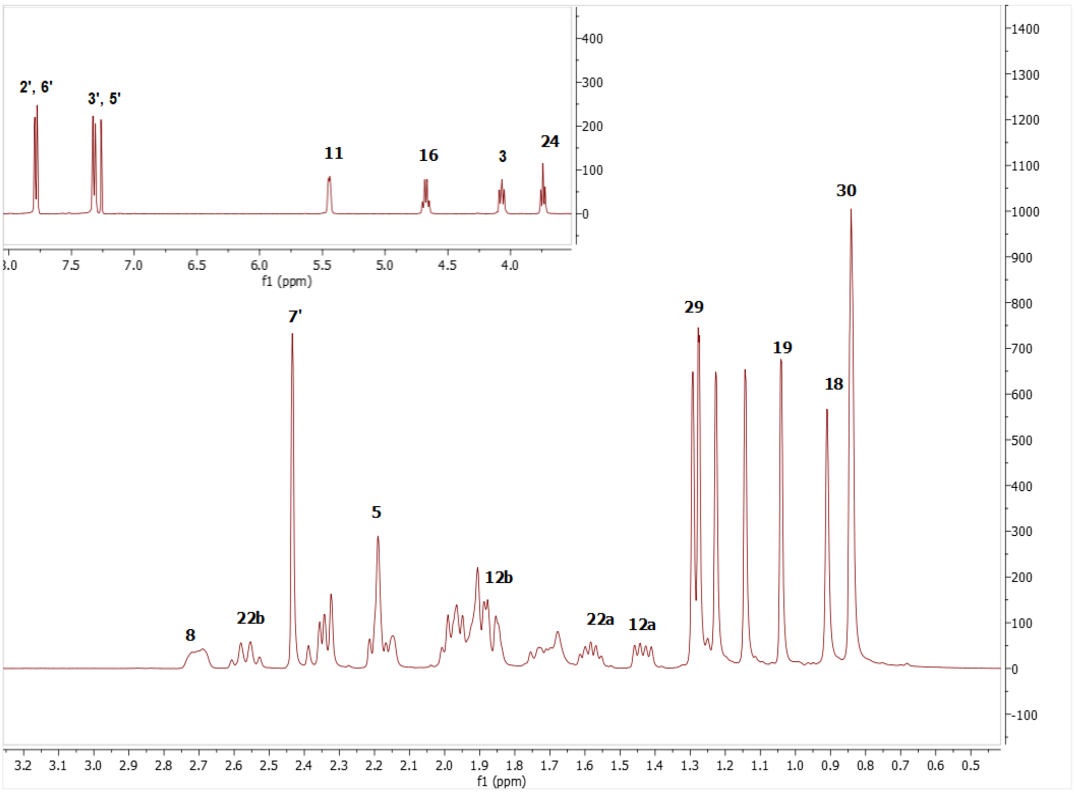

Spectrum 23. ^{1}H NMR Spectrum of AG-05.

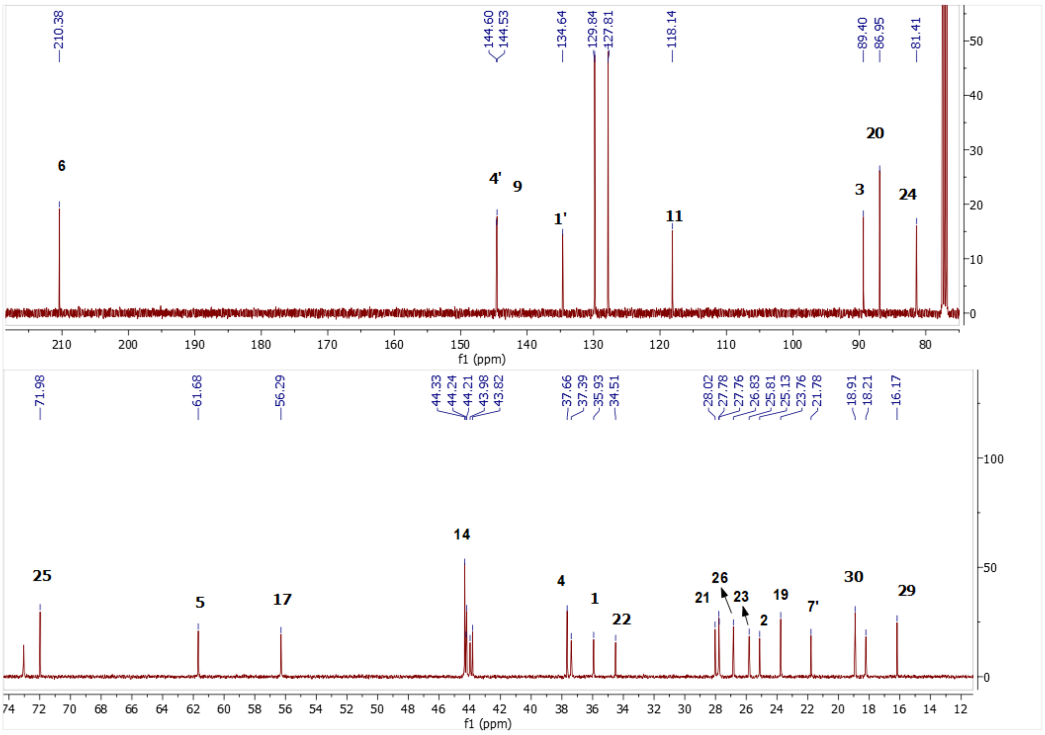

Spectrum 24. ^{13}C NMR Spectrum of AG-05.

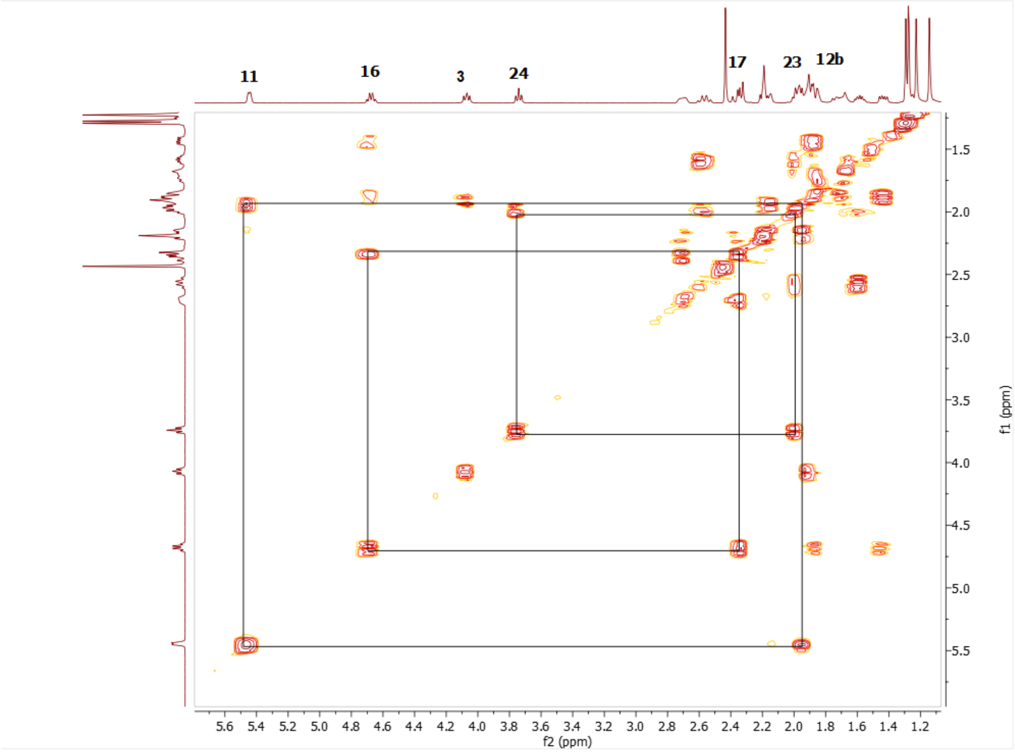

Spectrum 25. COSY spectrum of AG-05

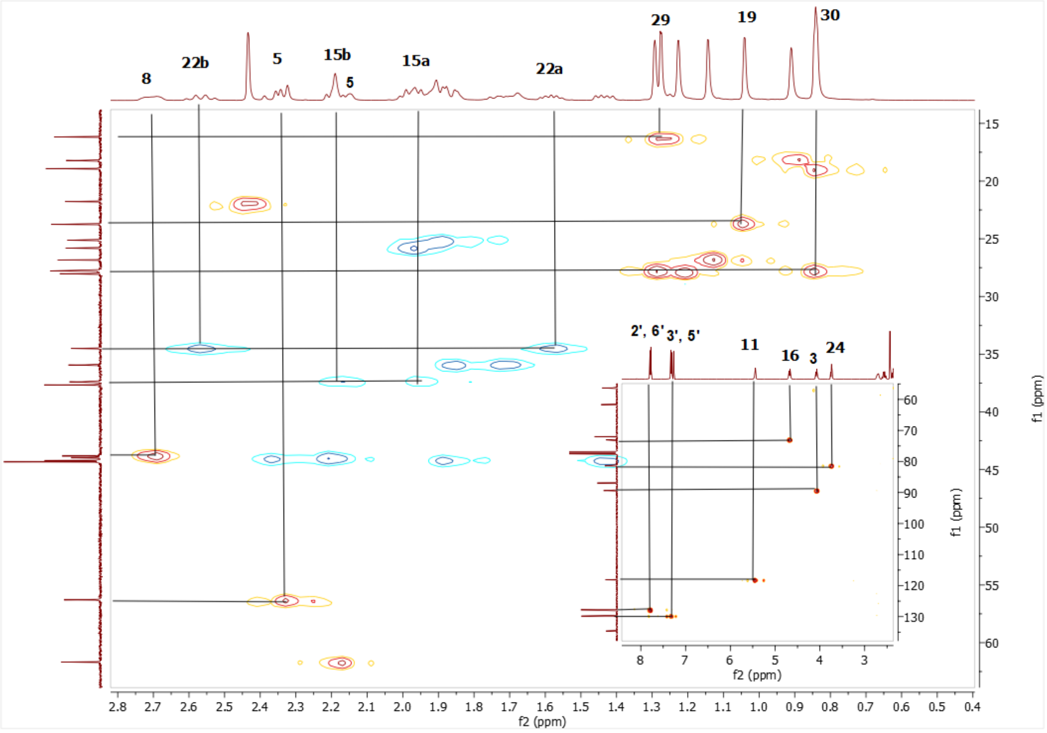

Spectrum 26. HSQC spectrum of AG-05.

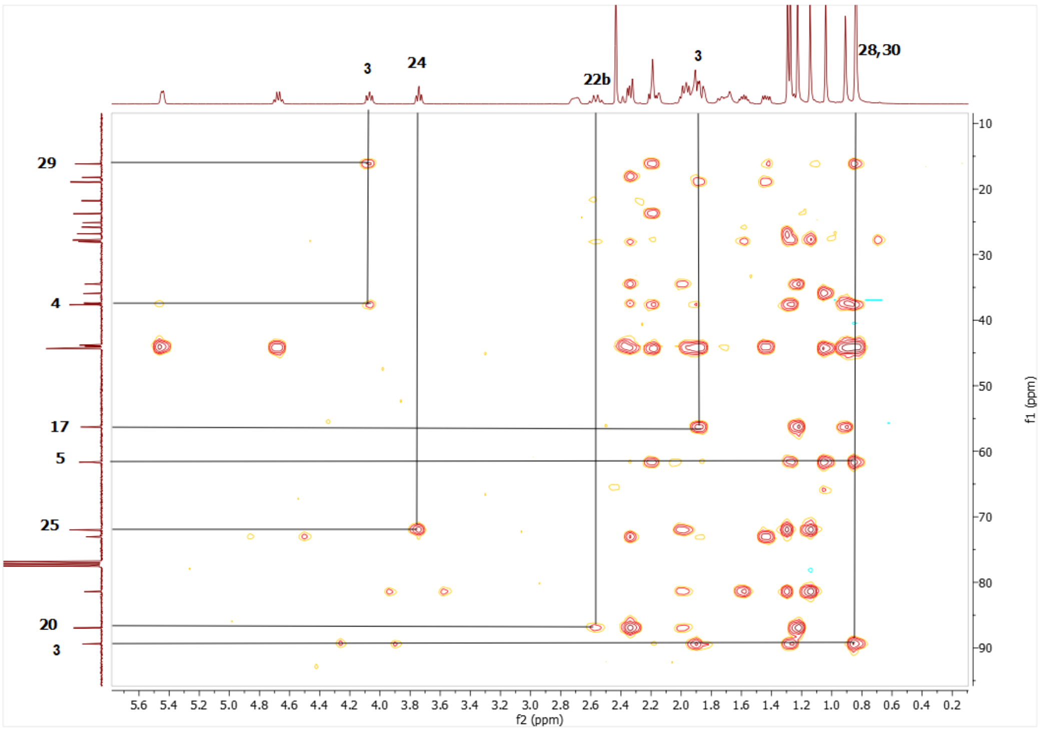

Spectrum 27. HMBC spectrum of AG-05.

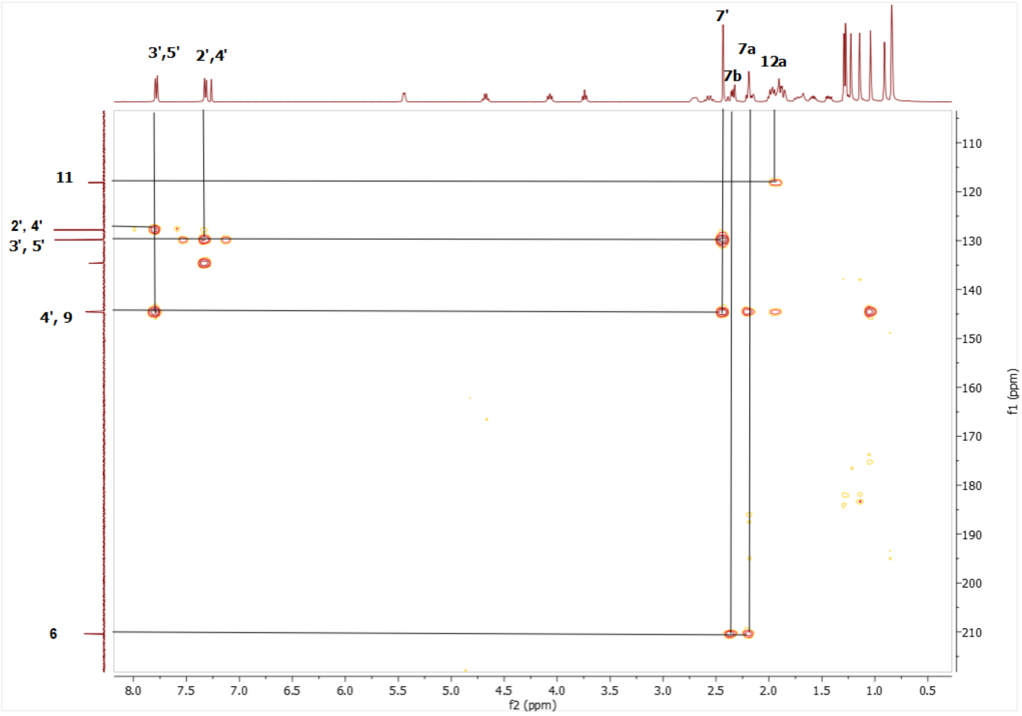

Spectrum 28. HMBC spectrum of AG-05.

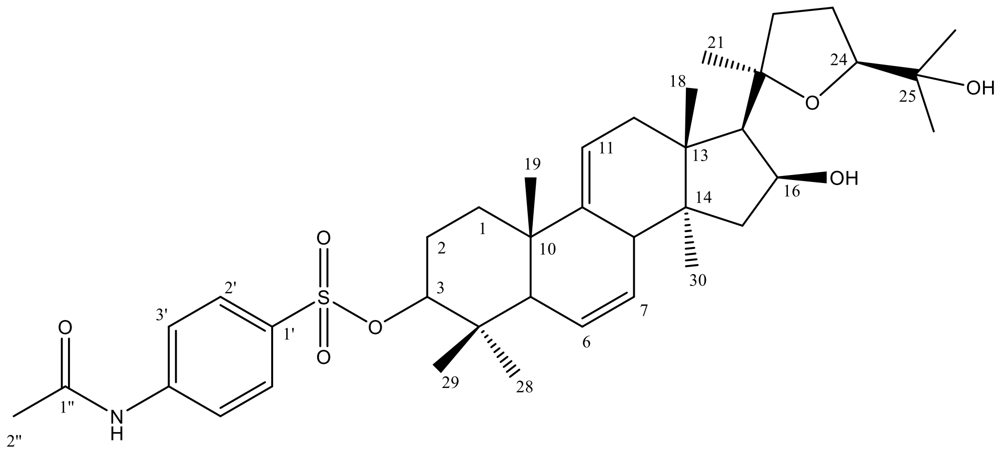

Supplementary Figure 7. Chemical Structure of AG-06

Supplementary Table 6. The ^{13}C and ^{1}H NMR data of AG-06 (100/400 MHz, δ ppm, in CDCl3).

**H/C**	**δ_C (ppm)**	**δ_H (ppm), *J* (Hz)**
**1**	33.9 t	1.55 m
**2**	25.4 t	1.91 m
**3**	90.9 d	4.15 dd (9.8, 6.6)
**4**	38.6 s	-
**5**	52.1 d	1.69 d (4.2)
**6**	126.1 d	5.58 d (10.4)
**7**	129.2 d	5.53 dt (10.4, 3)
**8**	43.6 d	2.8 brs
**9**	144.8 s	
**10**	38.2 s	-
**11**	113.8 d	5.13 t (5.3)
**12**	37.8 d	1.9 m, 2.0 m
**13**	44.5 s	-
**14**	43.4 s	-
**15**	43.8 t	1.53 m, 2.0 m
**16**	73.3 d	4.69 ddd (6.5, 6.5, 6)
**17**	56.3 d	2.12 d (7.7)
**18**	18.6 q	0.62 s
**19**	20.0 q	0.97 s
**20**	86.9 s	-
**21**	28.0 q	1.22 s
**22**	34.5 t	1.59 m, 2.55 q (10.5)
**23**	25.8 t	1.99 m
**24**	81.5 d	3.74 t (7.1, 7.1)
**25**	72.0 s	-
**26**	27.6 q	1.28 s
**27**	26.7 q	1.14 s
**28**	27.7 q	0.73 s

**29**	16.5 q	0.83 s
**30**	18.8 q	0.95 s
**1'**	131.8 s	
**2'**	129.0 d	7.8 d (8.4)
**3'**	119.3 d	7.7 d (8.8)
**4'**	143.2 s	
**5'**	119.3 d	7.7 d (8.8)
**6'**	129.0 d	7.8 d (8.4)
**1''**	169.3	-
**2''**	24.8 q	2.18 s

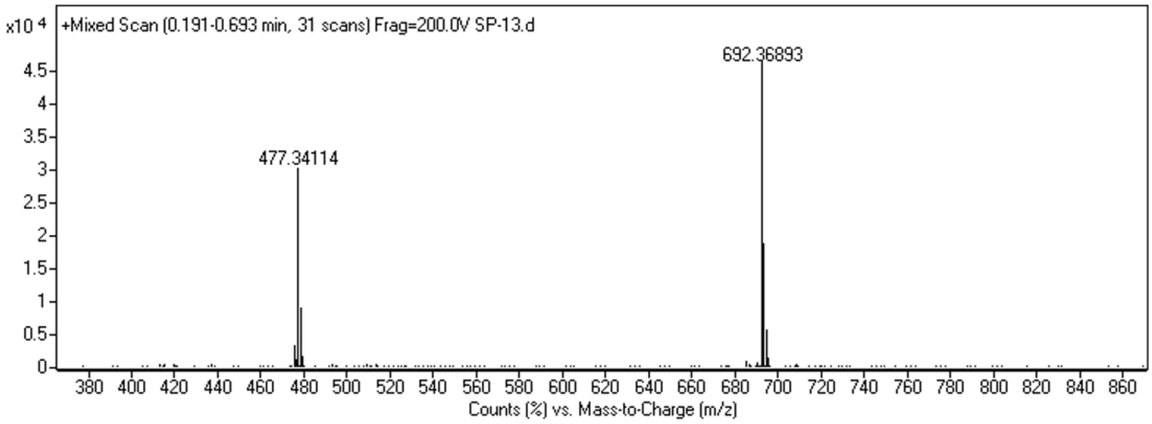

Spectrum 29. HR-ESI-MS Spectrum of AG-06 (positive mode).

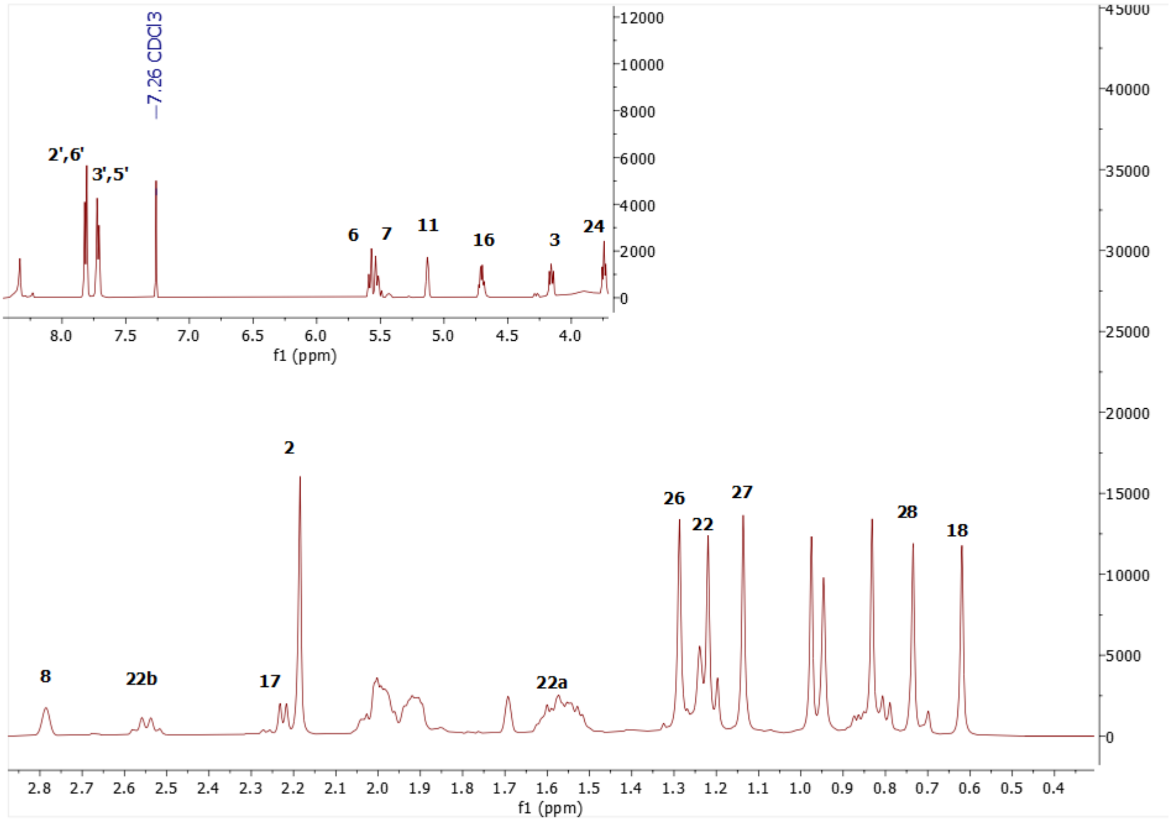

Spectrum 30. ^{1}H NMR Spectrum of AG-06.

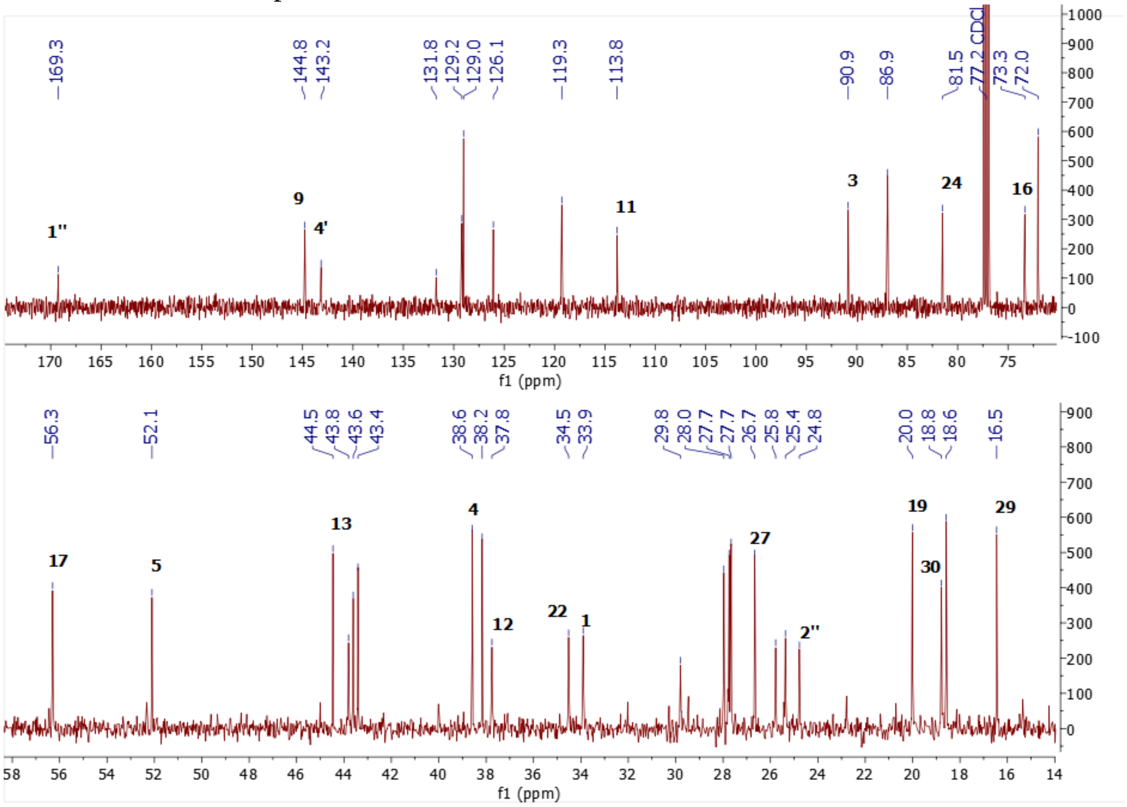

Spectrum 31. ^{13}C NMR Spectrum of AG-06.

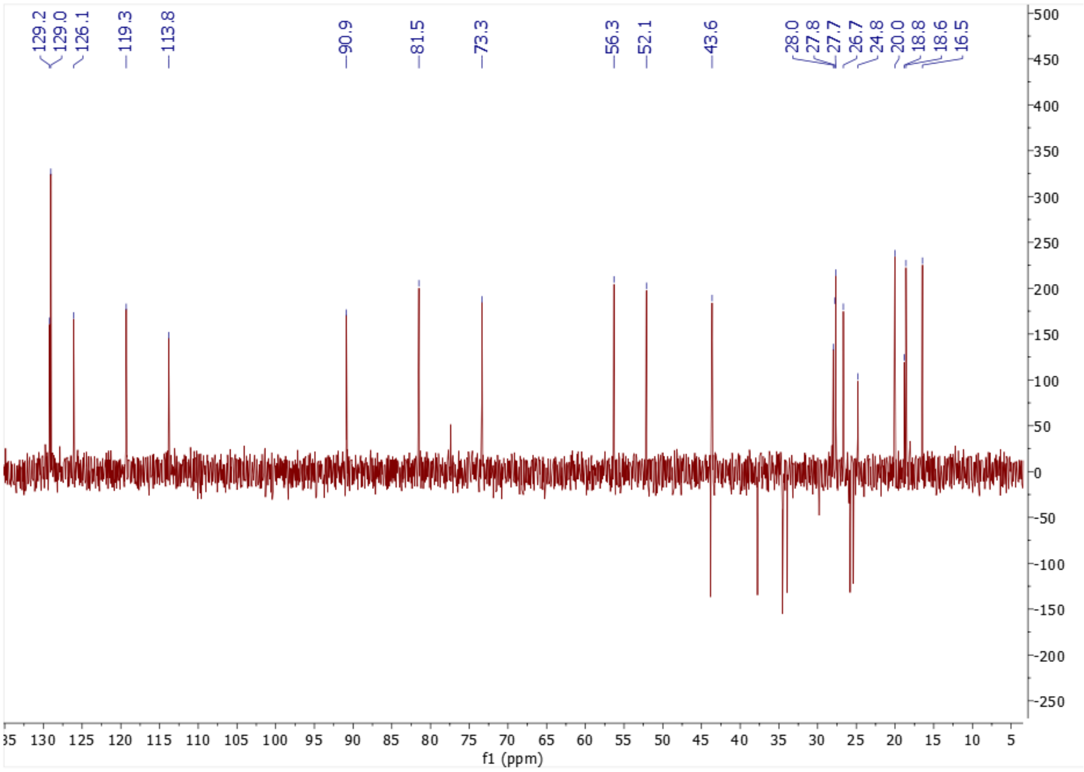

Spectrum 32. DEPT135 spectrum of AG-06

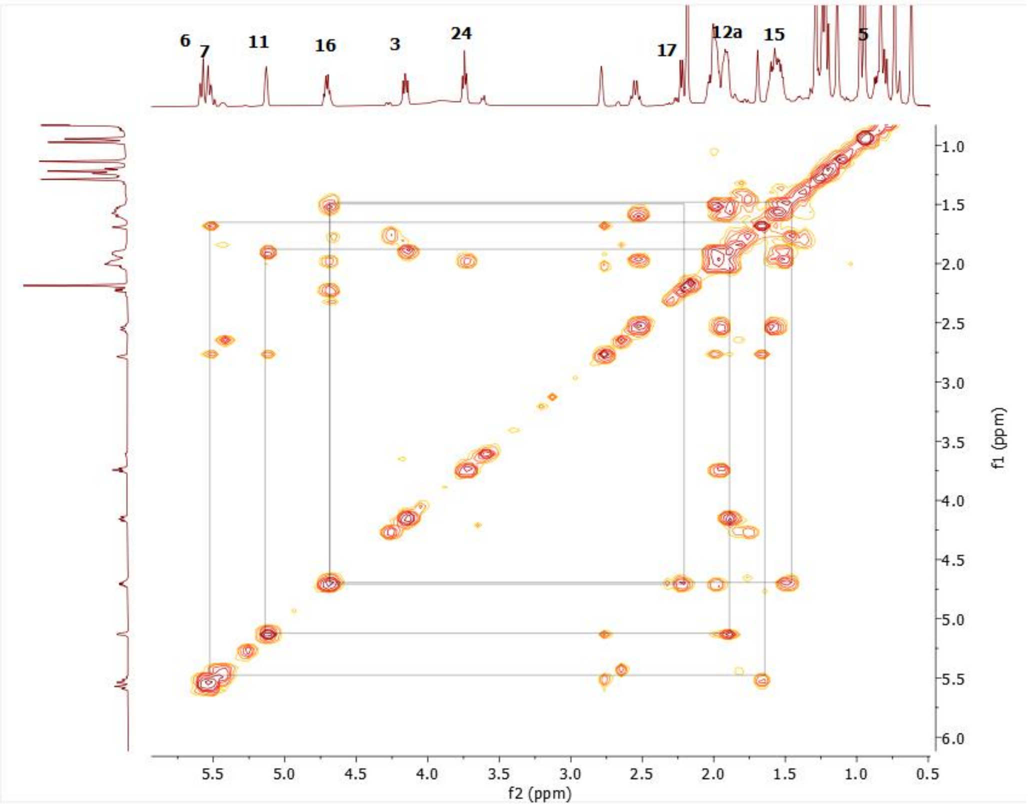

Spectrum 33. COSY spectrum of AG-06

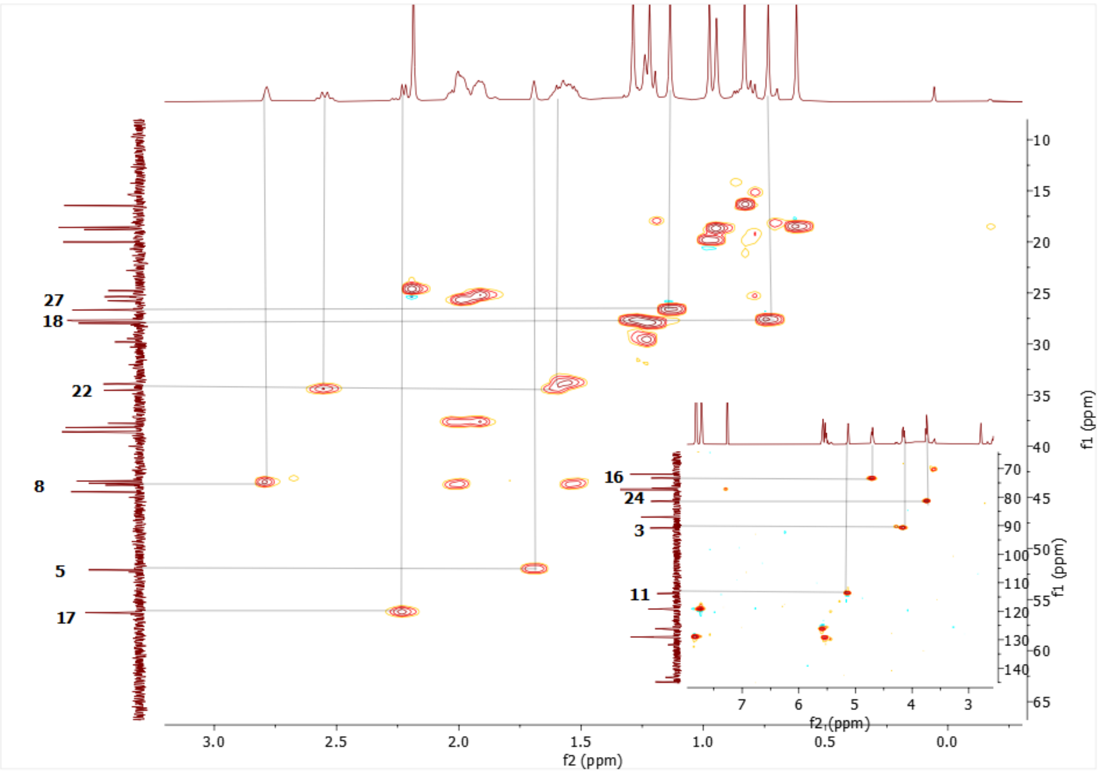

Spectrum 34. HSQC spectrum of AG-06.

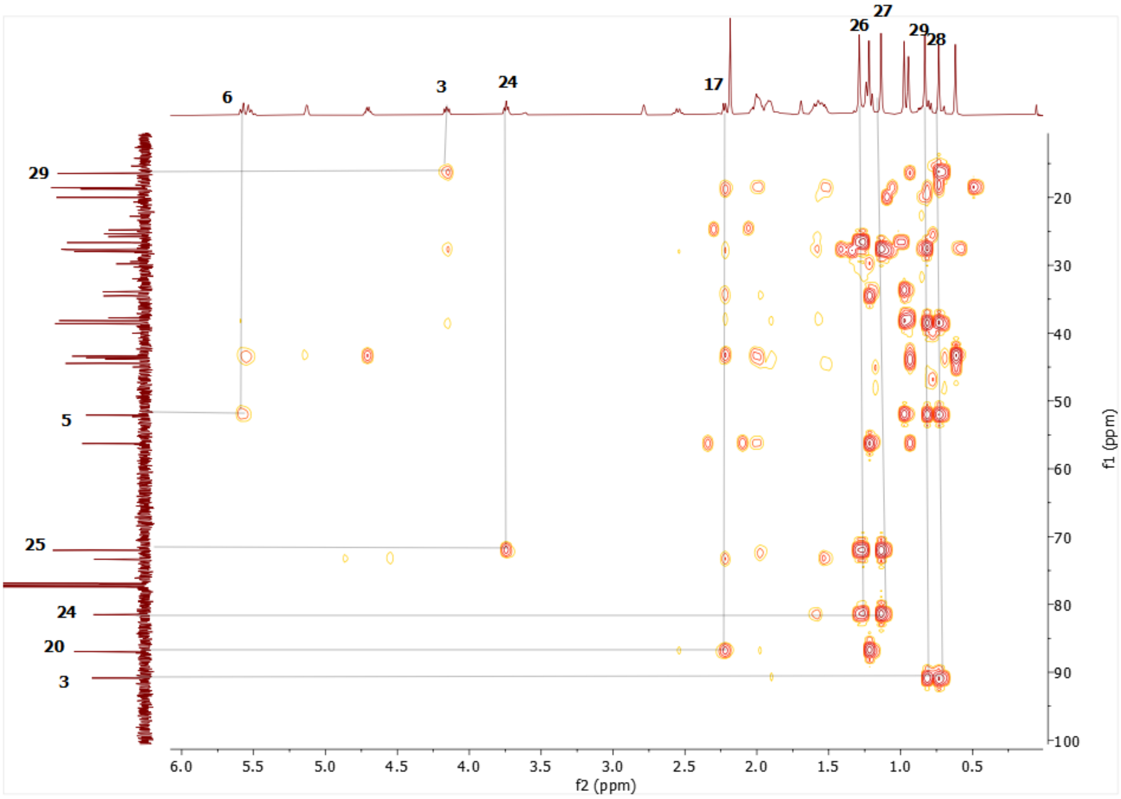

Spectrum 35. HMBC spectrum of AG-06

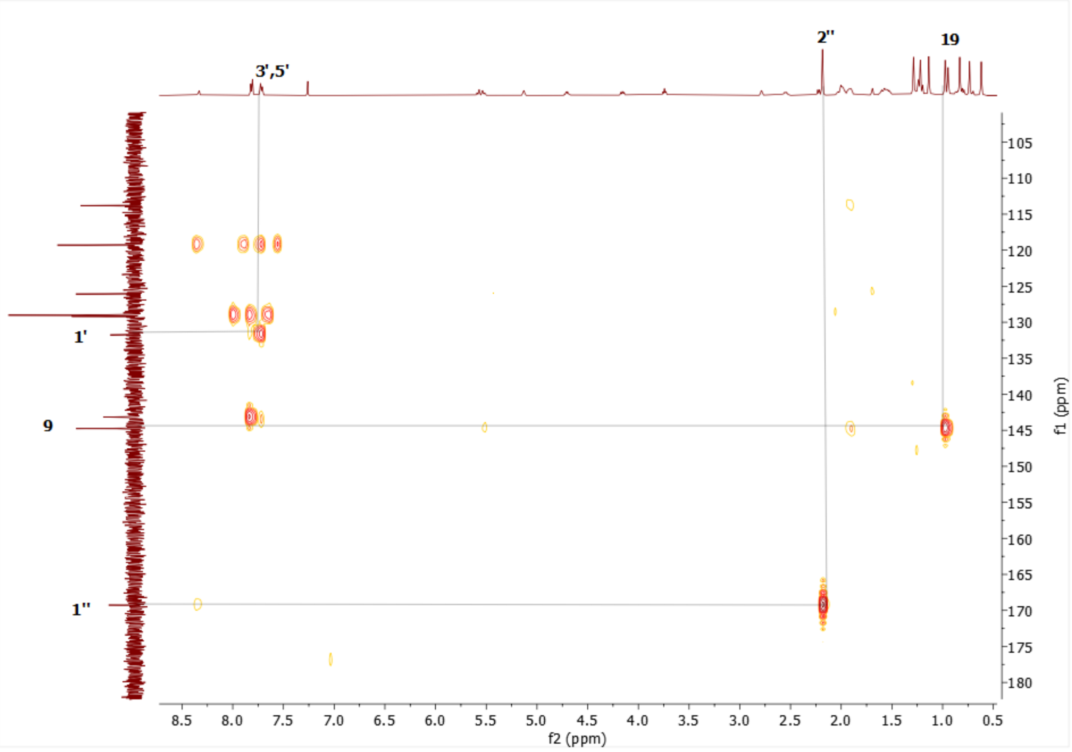

Spectrum 36. HMBC spectrum of AG-06

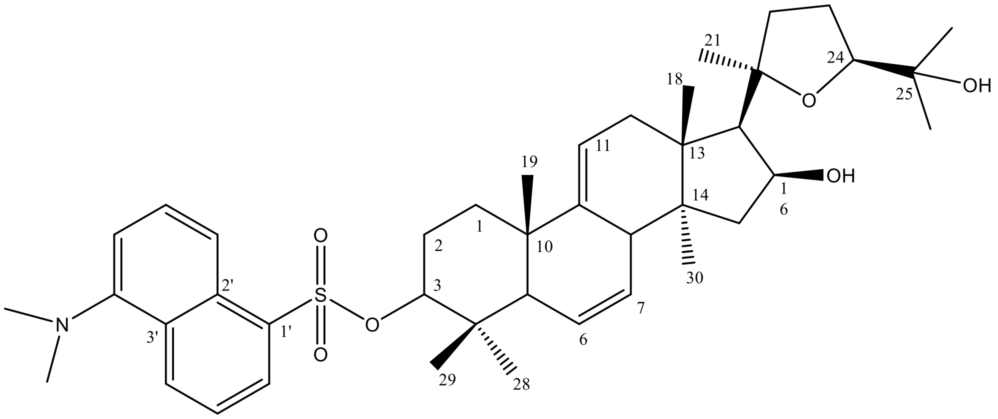

Supplementary Figure 8. Chemical Structure of AG-07

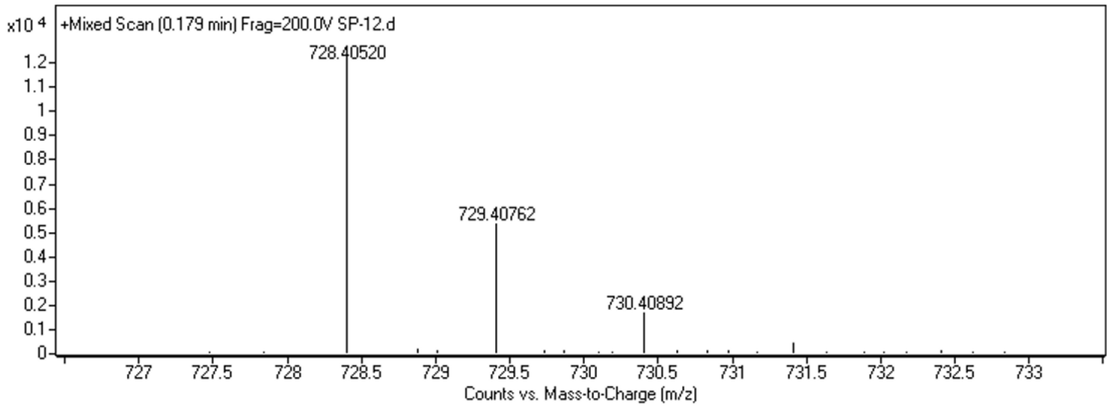

Spectrum 37. HR-ESI-MS Spectrum of AG-07 (positive mode).

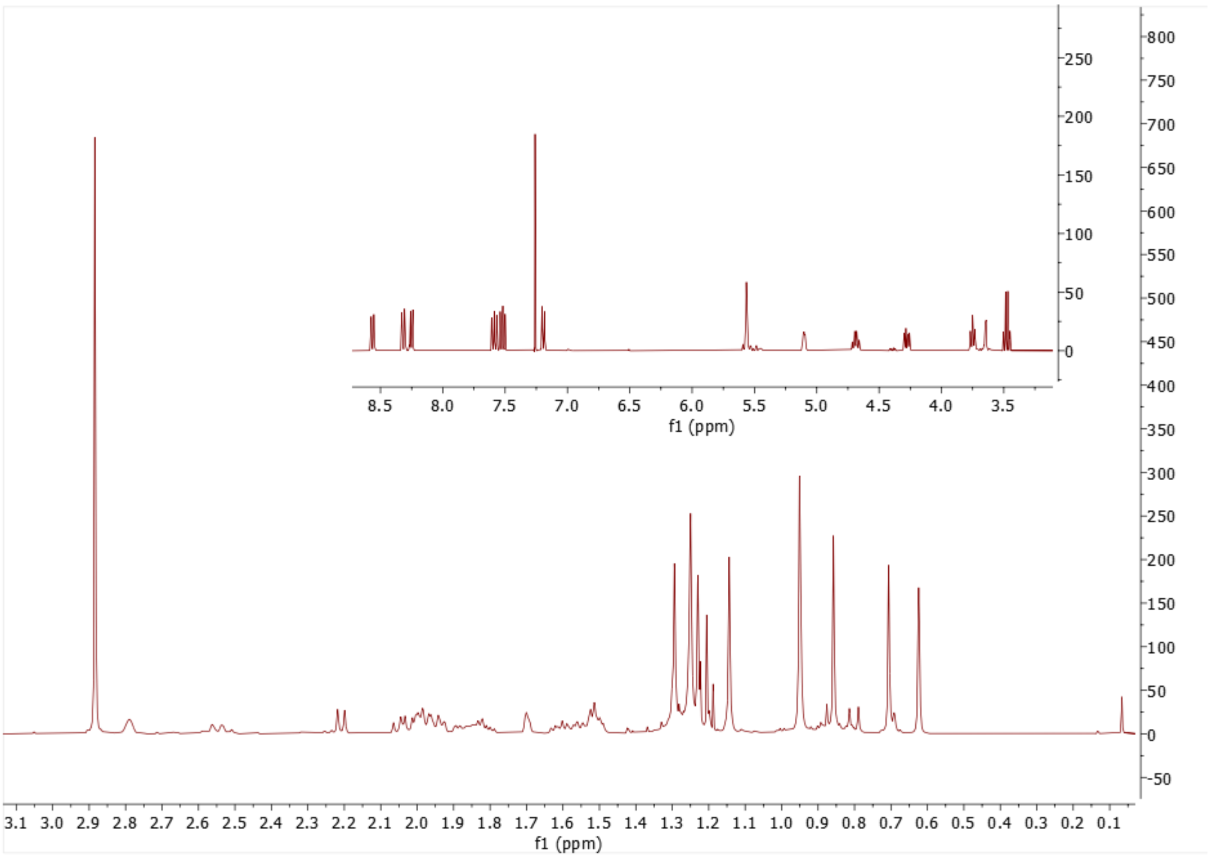

Spectrum 38. ^{1}H NMR Spectrum of AG-07.

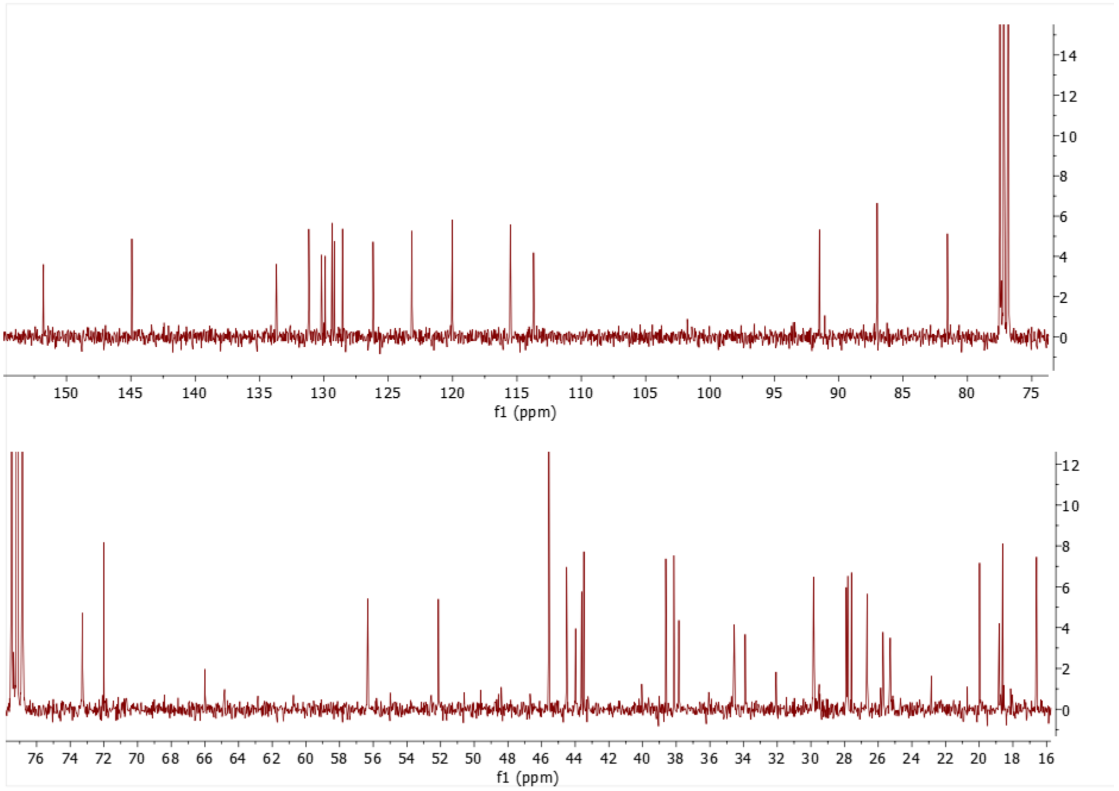

Spectrum 39. ^{13}C NMR Spectrum of AG-07.

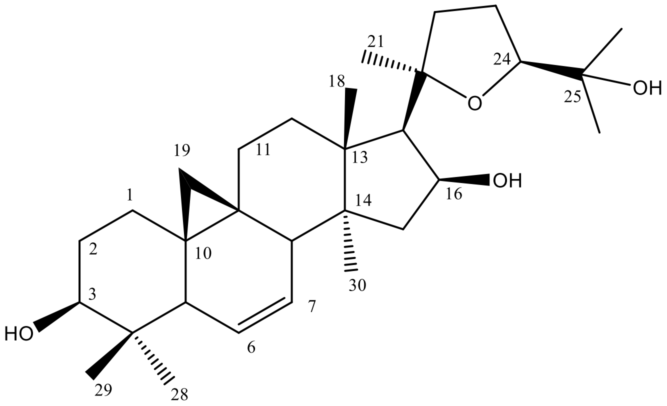

Supplementary Figure 9. Chemical Structure of CG-02

Supplementary Table 7. The ^{13}C and ^{1}H NMR data of CG-02 (100/400 MHz, δ ppm, in CDCl3).

**H/C**	**δC (ppm)**	**δH (ppm), *J* (Hz)**
**1**	29.8 t	1.25 s, 1.41 m

**2**	30.1 t	1.82 m, 1.59 m
**3**	78.5 d	3.32 dd (11.4, 4.6)
**4**	40.3 s	-
**5**	46.6 d	1.85 m
**6**	126.6 d	5.63 d (10.6)
**7**	129.2 d	5.48 brs
**8**	43.4 d	2.71 d (7.9)
**9**	20.6 s	-
**10**	28.4 s	-
**11**	25.2 t	1.40 m, 1.88 m
**12**	33.4 t	1.62 m, 1.42 m
**13**	45.2 s	-
**14**	48.5 s	-
**15**	43.6 t	1.84 m, 1.52 m
**16**	73.5 d	4.69 q (7.2)
**17**	56.4 d	2.27 d (7.6)
**18**	18.1 q	1.23 s
**19**	18.7 t	-0.16 d (4.3), 0.74 m
**20**	87.3 s	-
**21**	28.1 q	1.22 s
**22**	34.6 t	1.6 m, 2.59 q (10.6)
**23**	25.9 t	2 m
**24**	81.4 d	3.74 t (7.1)
**25**	72.1 s	-
**26**	27.9 q	1.31 s
**27**	26.7 q	1.15 s
**28**	14.5 q	0.78 s
**29**	25.6	1.05 s
**30**	18.2	1.23 s

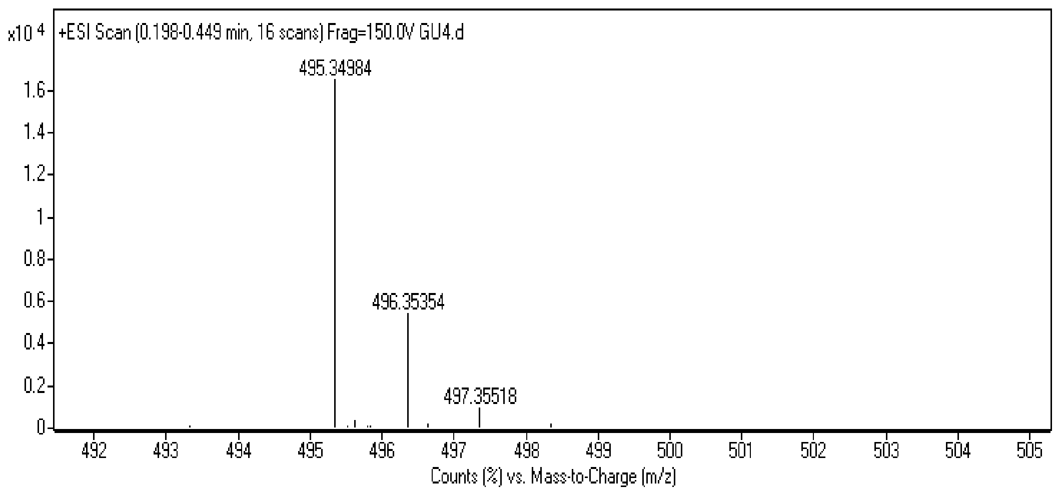

Spectrum 40. HR-ESI-MS Spectrum of CG-02 (positive mode).

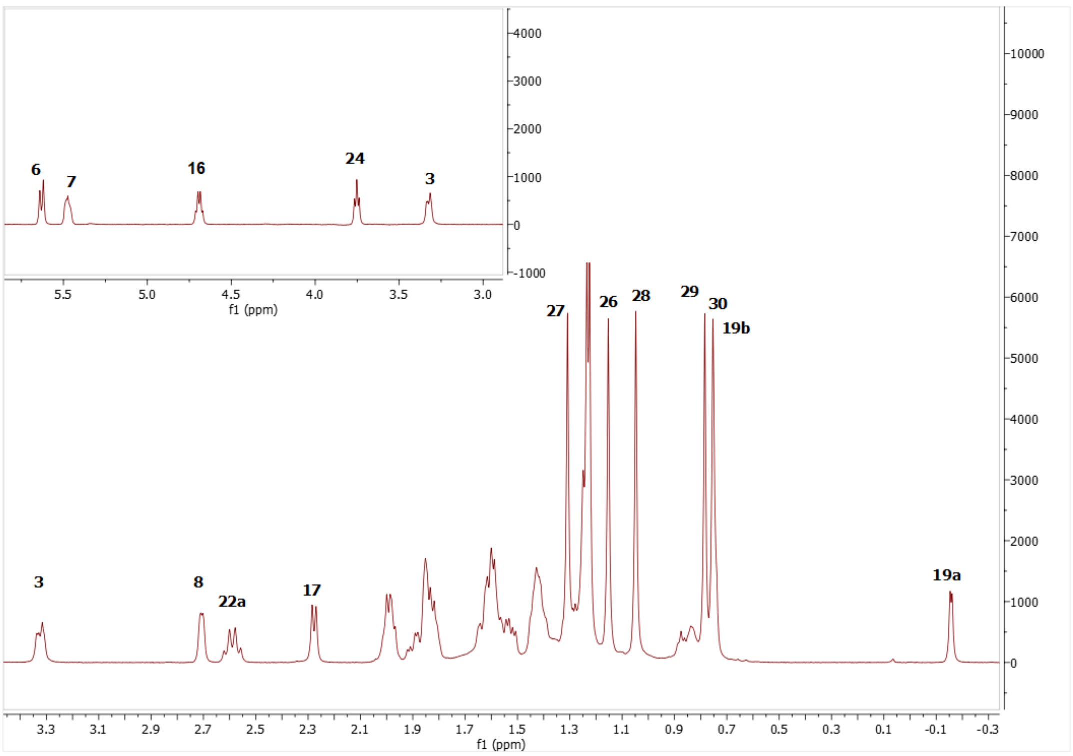

Spectrum 41. ^{1}H NMR Spectrum of CG-02.

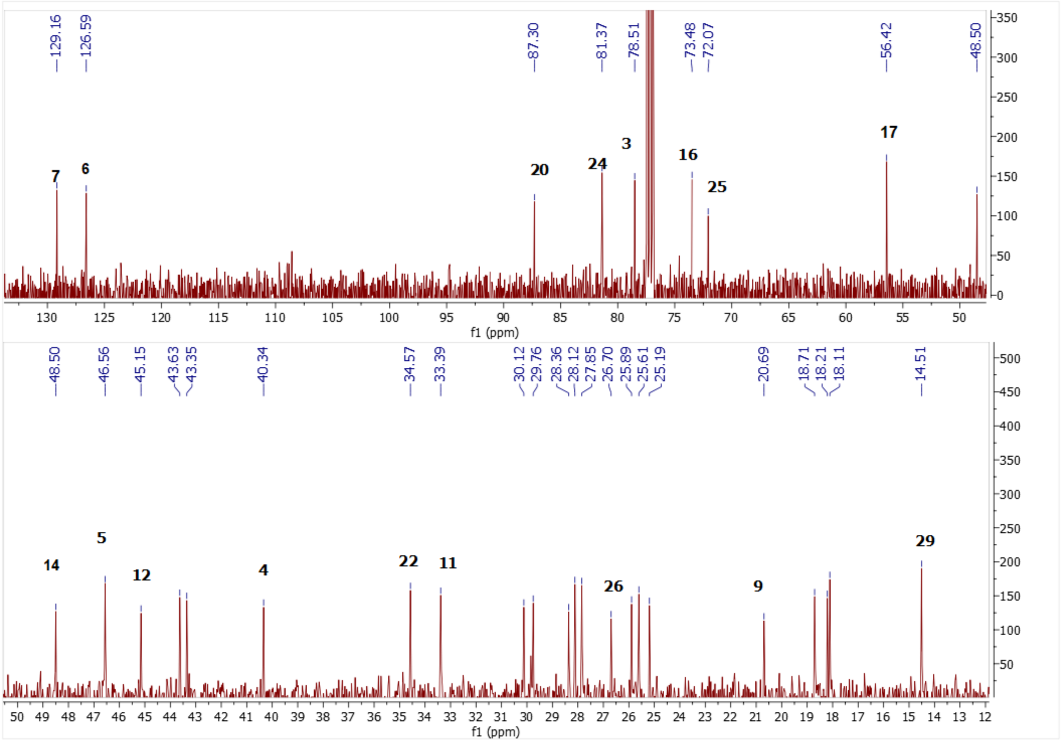

Spectrum 42. ^{13}C NMR Spectrum of CG-02.

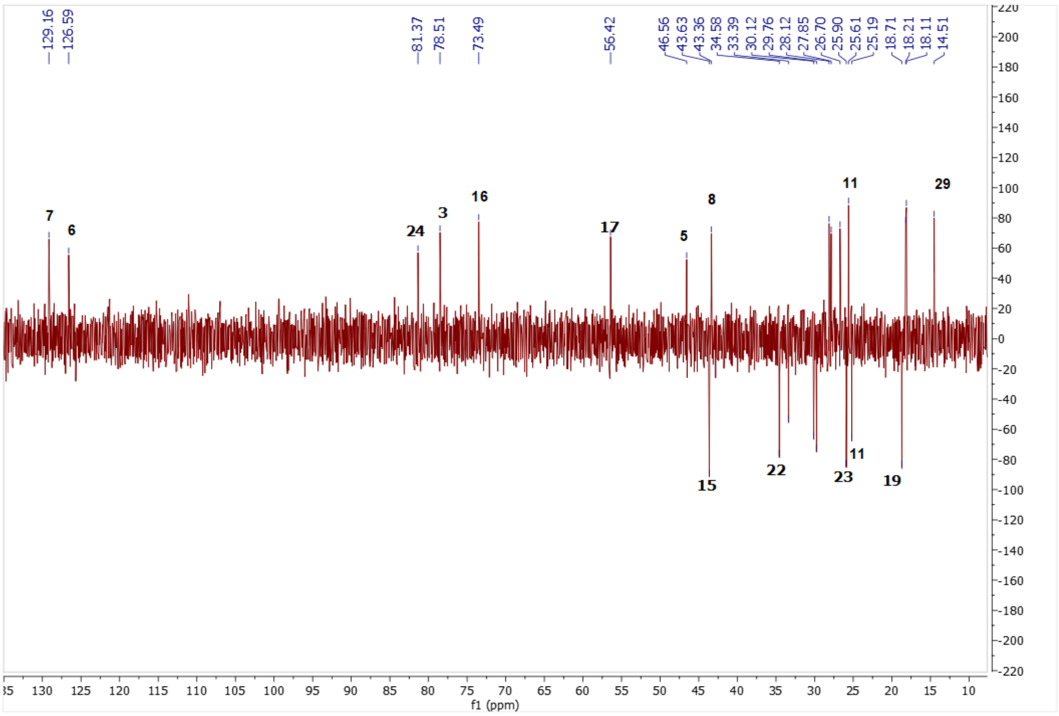

Spectrum 43. DEPT135 spectrum of CG-02.

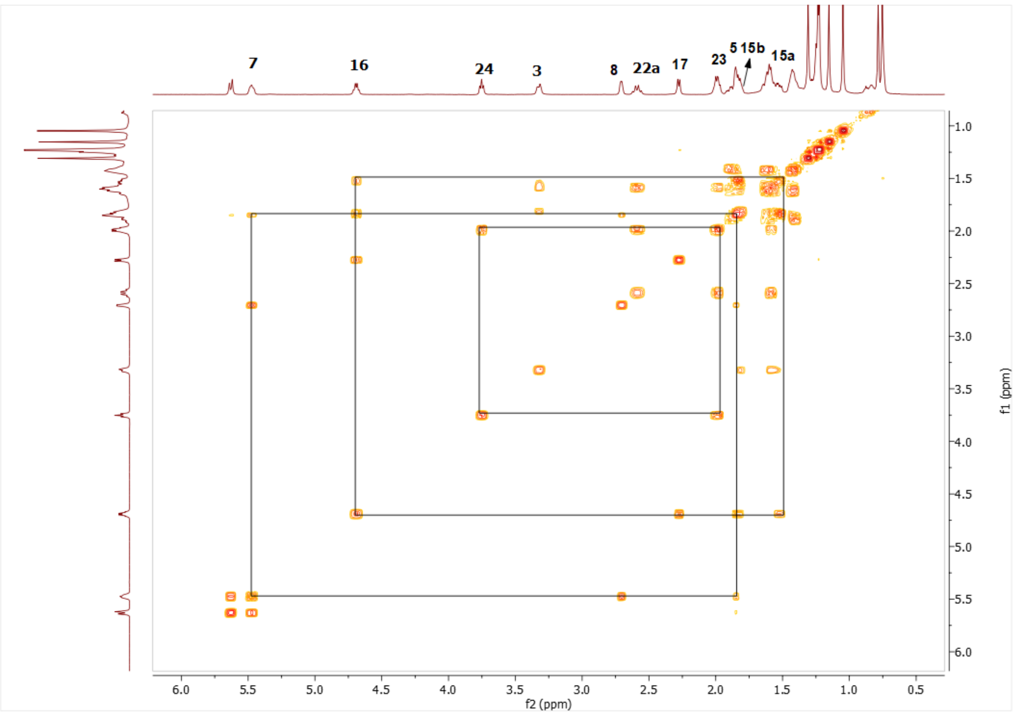

Spectrum 44. COSY spectrum of CG-02.

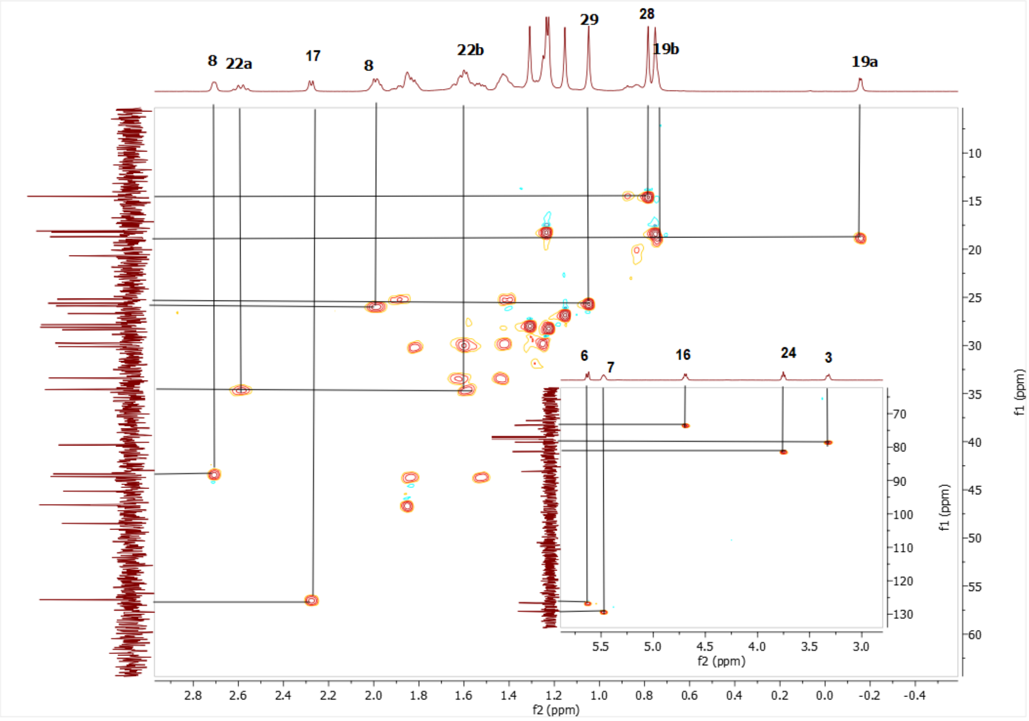

Spectrum 45. HMQC spectrum of CG-02.

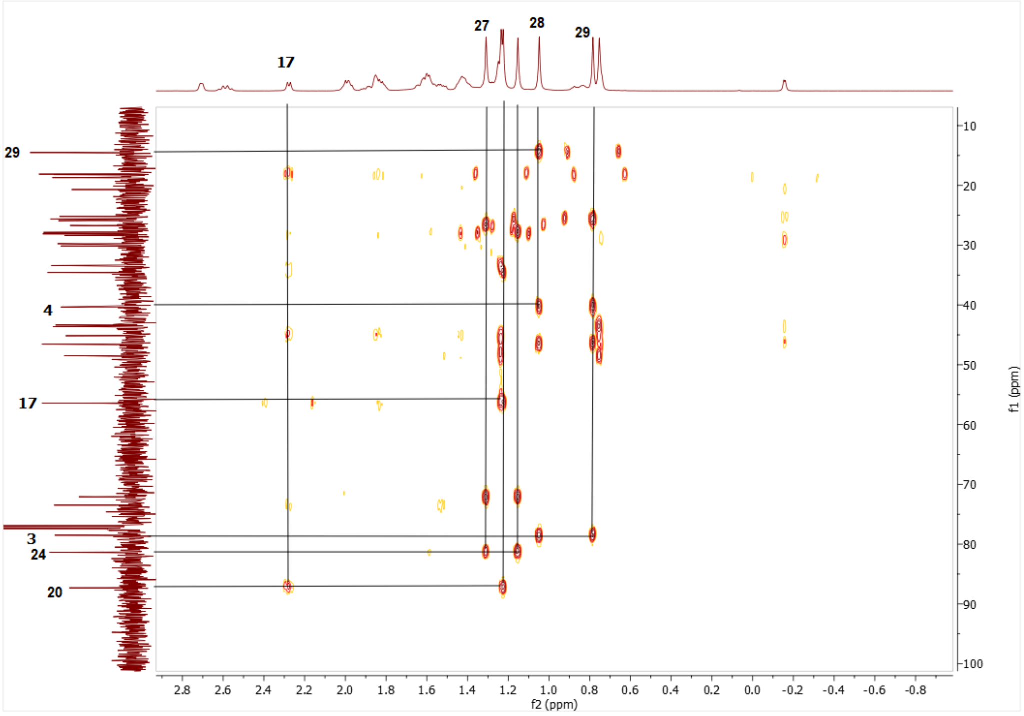

Spectrum 46. HMBC spectrum of CG-02.

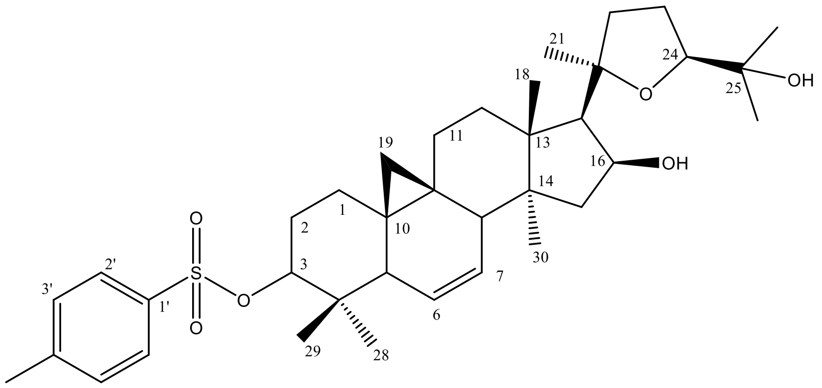

Supplementary Figure 10. Chemical Structure of CG-03

Supplementary Table 8. The ^{13}C and ^{1}H NMR data of CG-03 (100/400 MHz, δ ppm, in CDCl3).

H/C	δC (ppm)	δH (ppm), *J* (Hz)
**1**	29.5 t	1.38 m, 1.58 m
**2**	27.8 t	1.77 m, 1.89 m
**3**	90.2 s	4.31 dd (11.8, 4.6)
**4**	40 s	-
**5**	46.7 d	1.86 m
**6**	125.6 d	5.51 d (10.7)
**7**	129.8 d	5.46 ddd (10.7 ,6, 3)
**8**	43.3 d	2.68 dd (6, 2.5)
**9**	20.7 s	-
**10**	27.9 s	-
**11**	25.2 t	1.34 m, 1.85 m
**12**	33.3 t	1.41 m,1.57 m
**13**	48.6 s	-
**14**	45.1 s	-
**15**	43.5 t	1.48 m, 1.79 m
**16**	73.4 d	4.68 ddd (7.7, 7.7, 6.1)
**17**	56.4 d	2.26 d (7.7)
**18**	18.1 q	1.21 s
**19**	18.6 t	-0.17, 0.72 d (4.2)
**20**	87.2 s	-
**21**	28.2 q	1.19 s
**22**	34.5 t	1.56 m, 2.58 q (10.5)
**23**	25.9 t	2 td (10.5, 9, 5)
**24**	81.3 d	3.74 (7.1)
**25**	72.0 s	-
**26**	28.8 q	1.29 s
**27**	26.7 q	1.13 s
**28**	25.4 q	0.81 s
**29**	15.4 q	0.8 s
**30**	18.2 q	0.71 s
**1’**	132.9 s	-
**2’**	127.8 d	7.79 d (8.2)
**3’**	129.8 d	7.32 d (8.2)
**4’**	144.5 s	-
**5’**	129.8 d	7.32 d (8.2)
**6’**	127.8 d	7.79 d (8.2)
**7’**	21.2 q	2.43 s

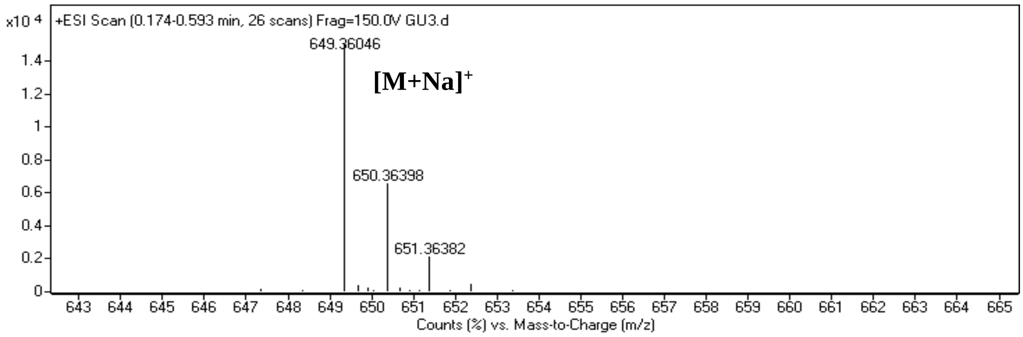

Spectrum 47. HR-ESI-MS Spectrum of CG-03 (positive mode).

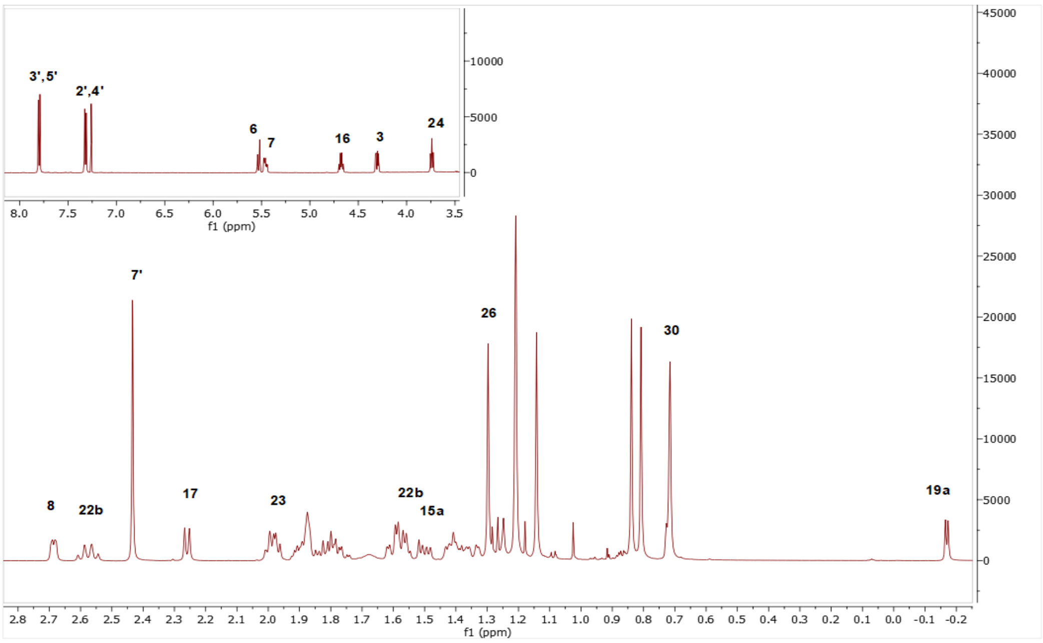

Spectrum 48. ^{1}H NMR Spectrum of CG-03.

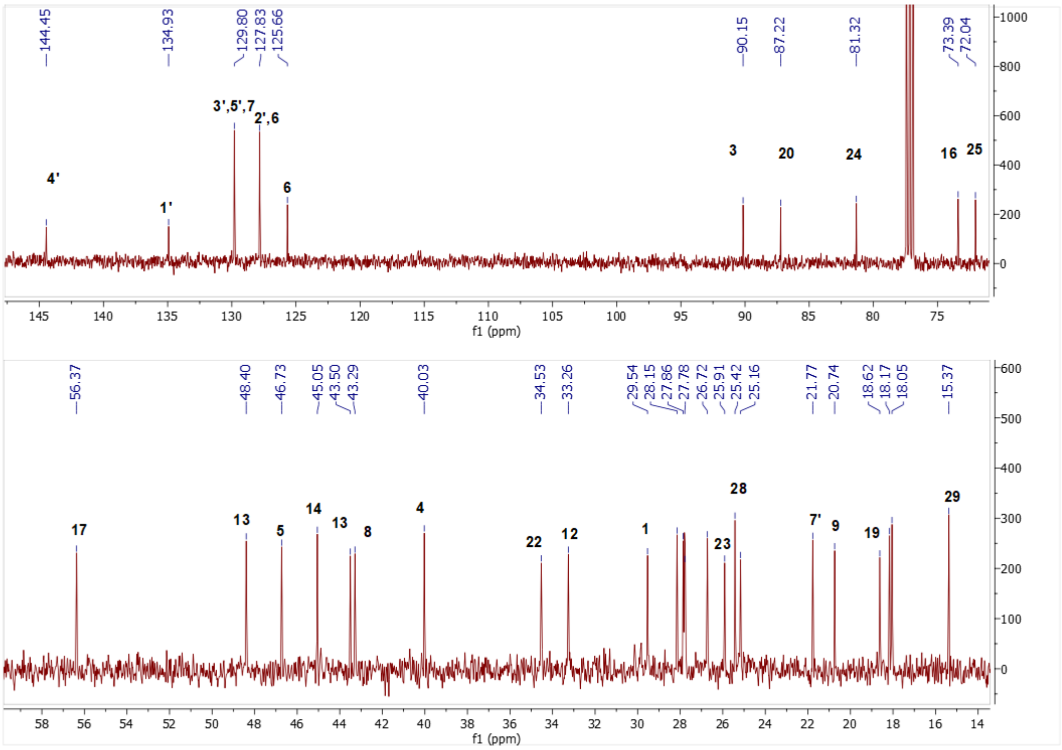

Spectrum 49. ^{13}C NMR Spectrum of CG-03.

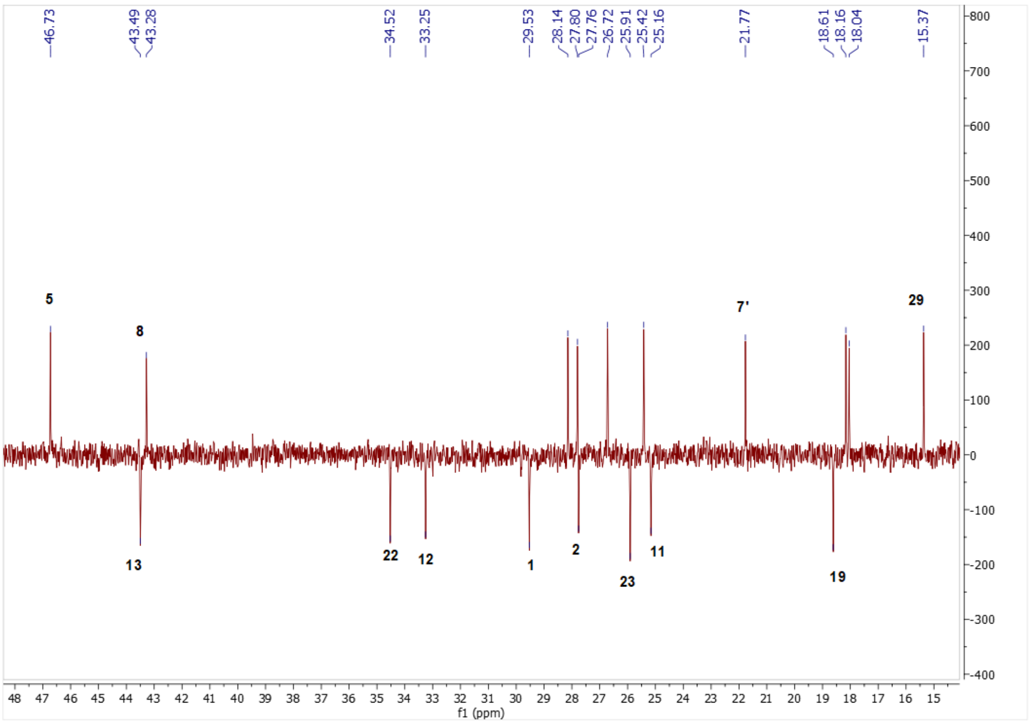

Spectrum 50. DEPT135 spectrum of CG-03.

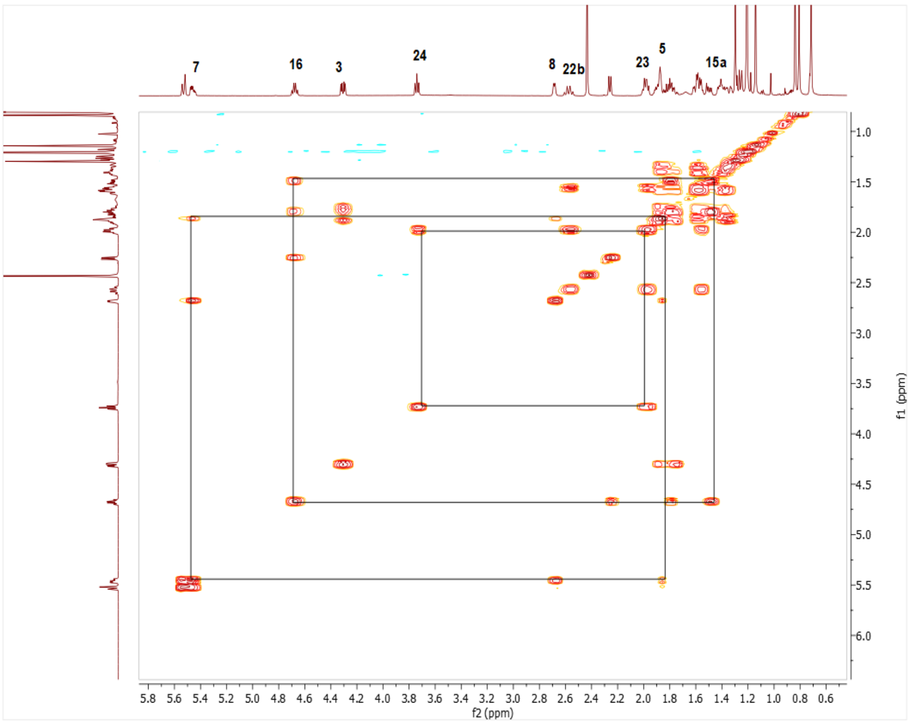

Spectrum 51. COSY spectrum of CG-03.

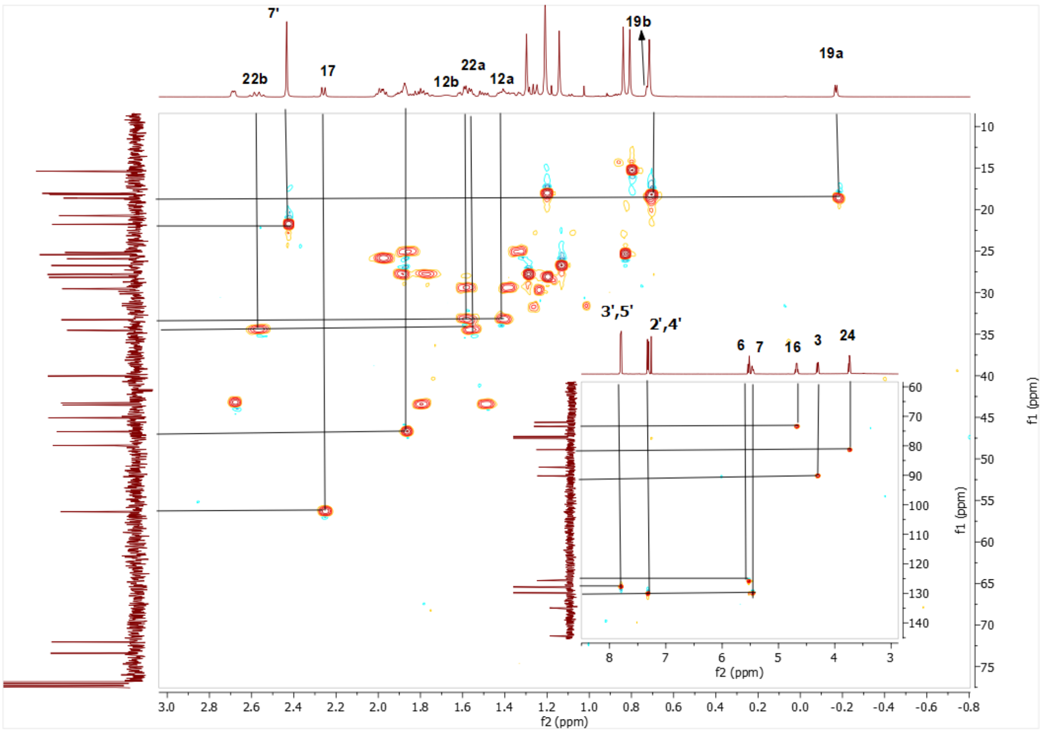

Spectrum 52. HMQC spectrum of CG-03.

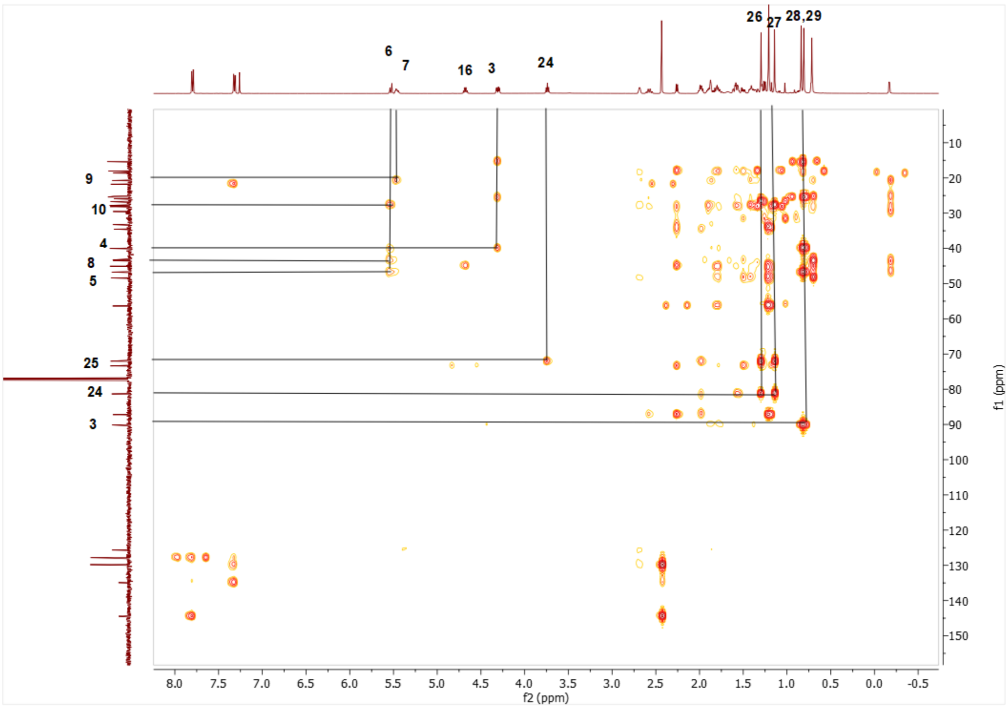

Spectrum 53. HMBC spectrum of CG-03.

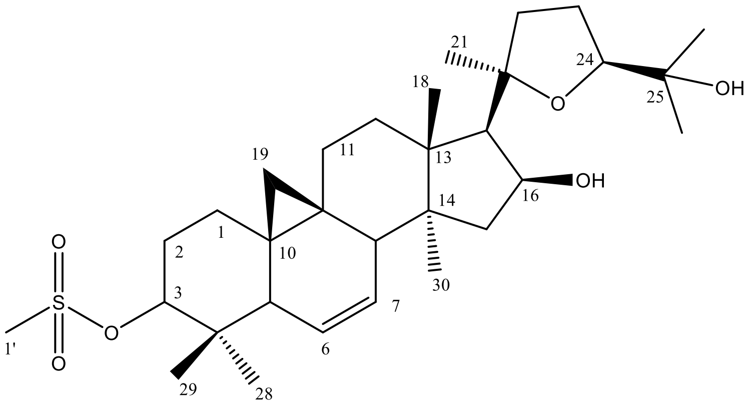

Supplementary Figure 11. Chemical Structure of CG-04

Supplementary Table 9. The ^{13}C and ^{1}H NMR data of CG-04 (100/400 MHz, δ ppm, in CDCl3).

**H/C**	**δ_C (ppm)**	**δ_H (ppm), *J* (Hz)**
**1**	29.5 d	1.47 m, 1.68 m
**2**	28.2 t	1.9 m, 2.12 dd (12.5, 3.9)
**3**	89.7 d	4.43 dd (11.9, 4.6)
**4**	40.1 s	-
**5**	46.7 d	1.96 m
**6**	125.6 d	5.58 d (10.6)
**7**	129.9 d	5.49 ddd (10.6, 6.1, 3.1)
**8**	43.3 d	2.71 dd (6.2, 2.6)
**9**	20.8 s	-
**10**	27.9 s	-
**11**	25.2 t	1.38 m, 1.89 m
**12**	33.3 t	1.43 m, 1.61m
**13**	48.4 s	-
**14**	45.1 s	-
**15**	43.5 t	1.51m, 1.81m
**16**	73.4 d	4.7 ddd (7.7, 7.7, 6)
**17**	56.4 d	2.27 d (7.6)
**18**	18.1 q	1.22 s
**19**	18.6 t	-0.12 d (4.3), 0.77 d (3.6)
**20**	87.2 s	-
**21**	28.2 q	1.2 s
**22**	34.5 t	1.6 m, 2.58 d (10.6)
**23**	25.8 t	2.0 m
**24**	81.3 d	3.73 t (7.1)
**25**	72.0 s	-
**26**	27.8 q	1.29 s
**27**	26.7 q	1.14 s
**28**	25.7 q	1.05 s
**29**	15.4 q	0.86 s
**30**	18.2 q	0.74 s
**1’**	38.9 q	3.01 s

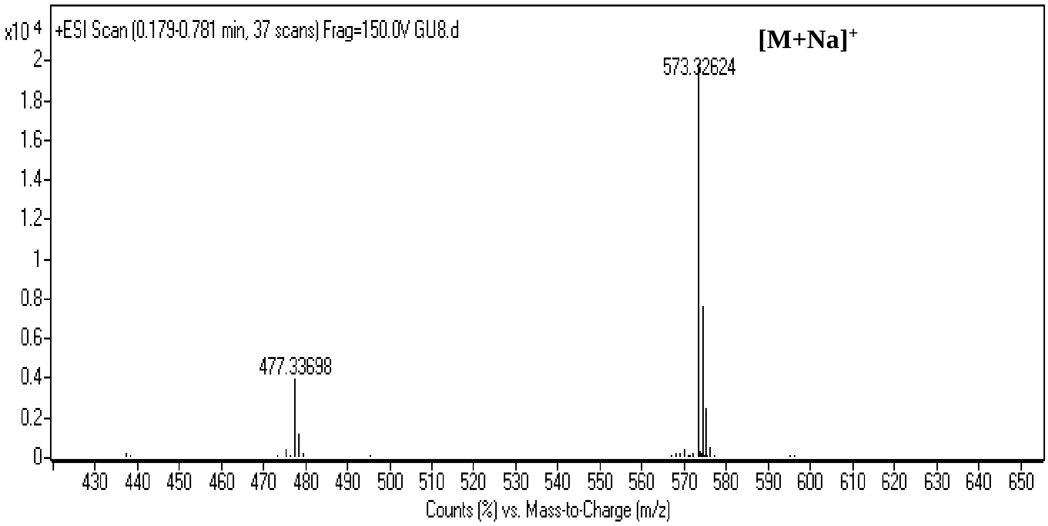

Spectrum 54. HR-ESI-MS Spectrum of CG-04 (positive mode).

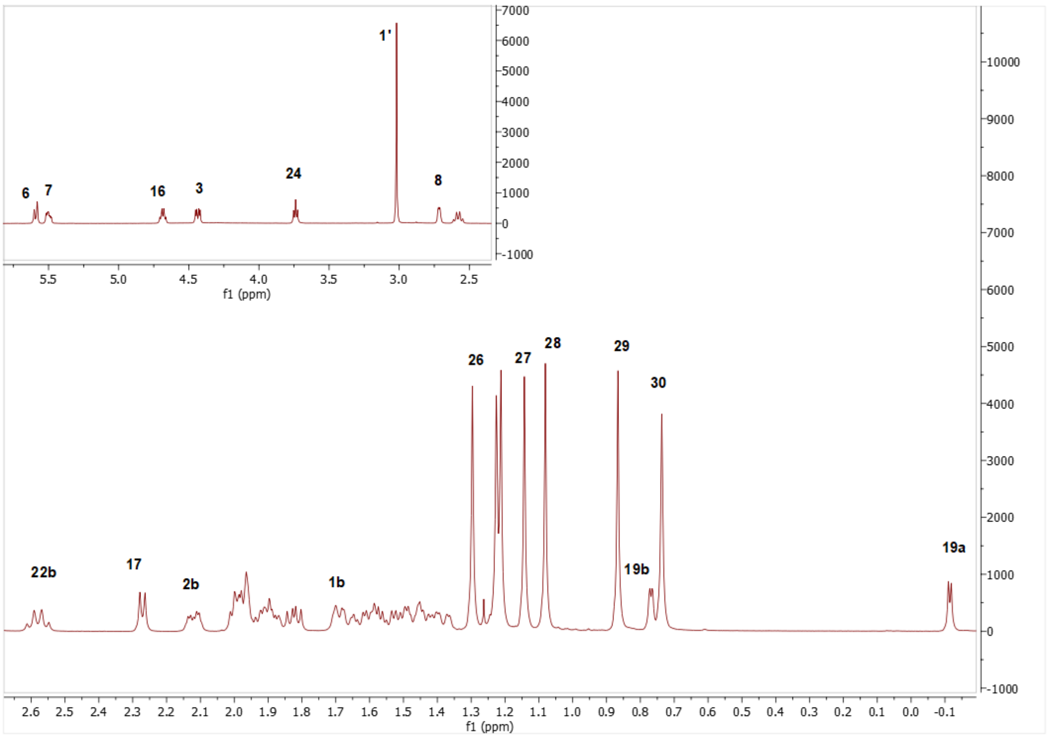

Spectrum 55. ^{1}H NMR Spectrum of CG-04.

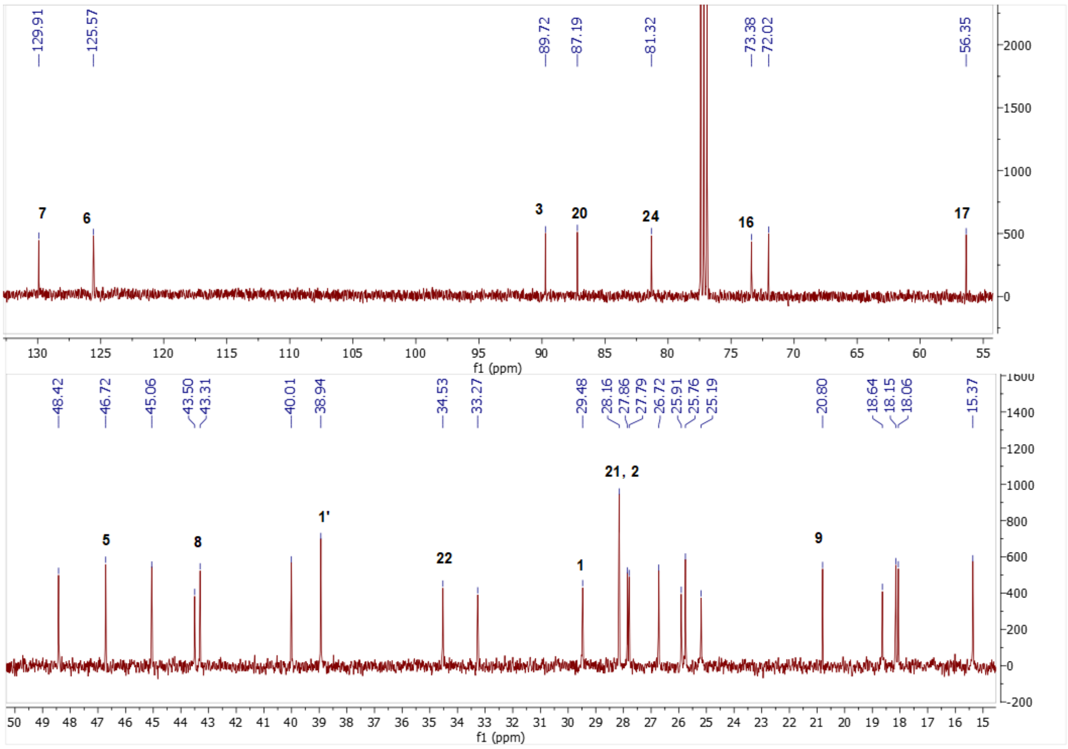

Spectrum 56. ^{13}C NMR Spectrum of CG-04.

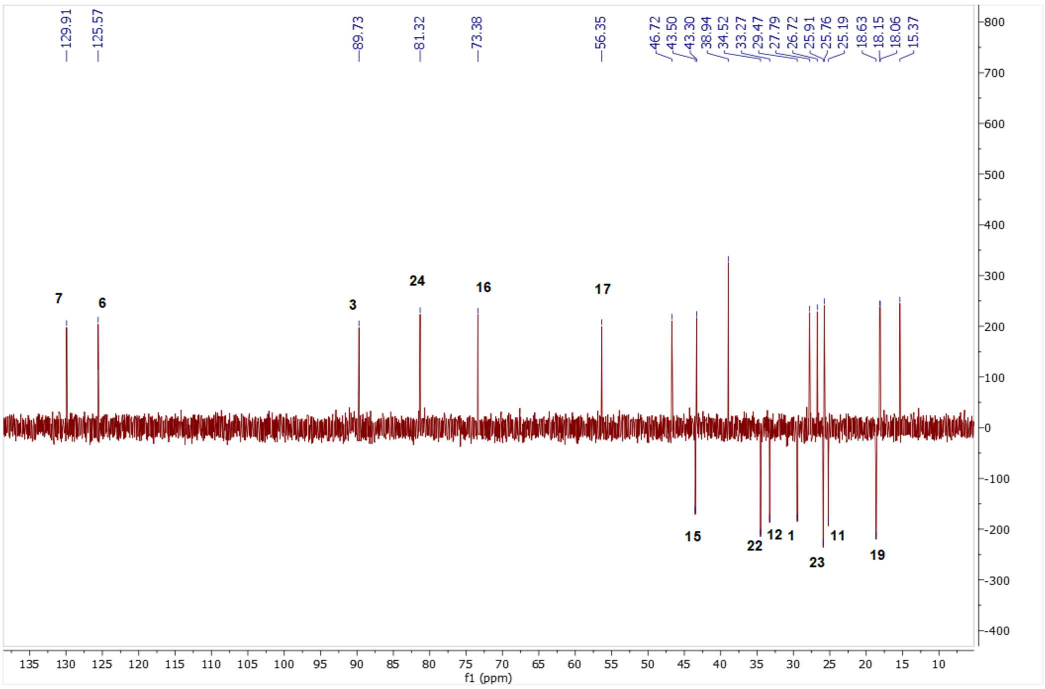

Spectrum 57. DEPT135 spectrum of CG-04.

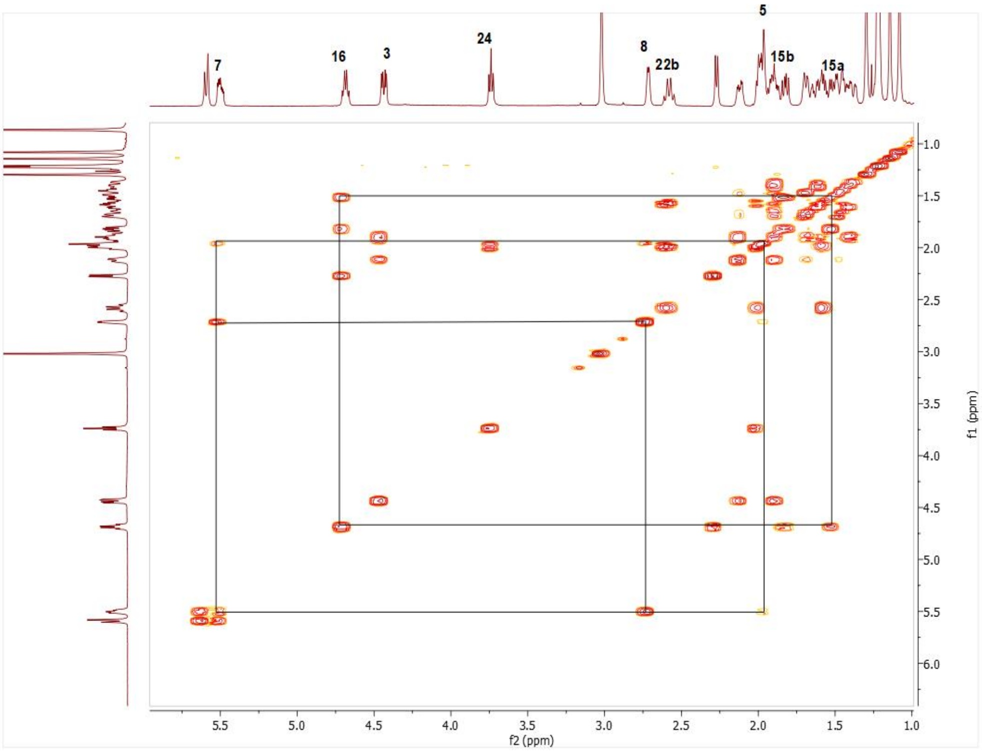

Spectrum 58. COSY spectrum of CG-04.

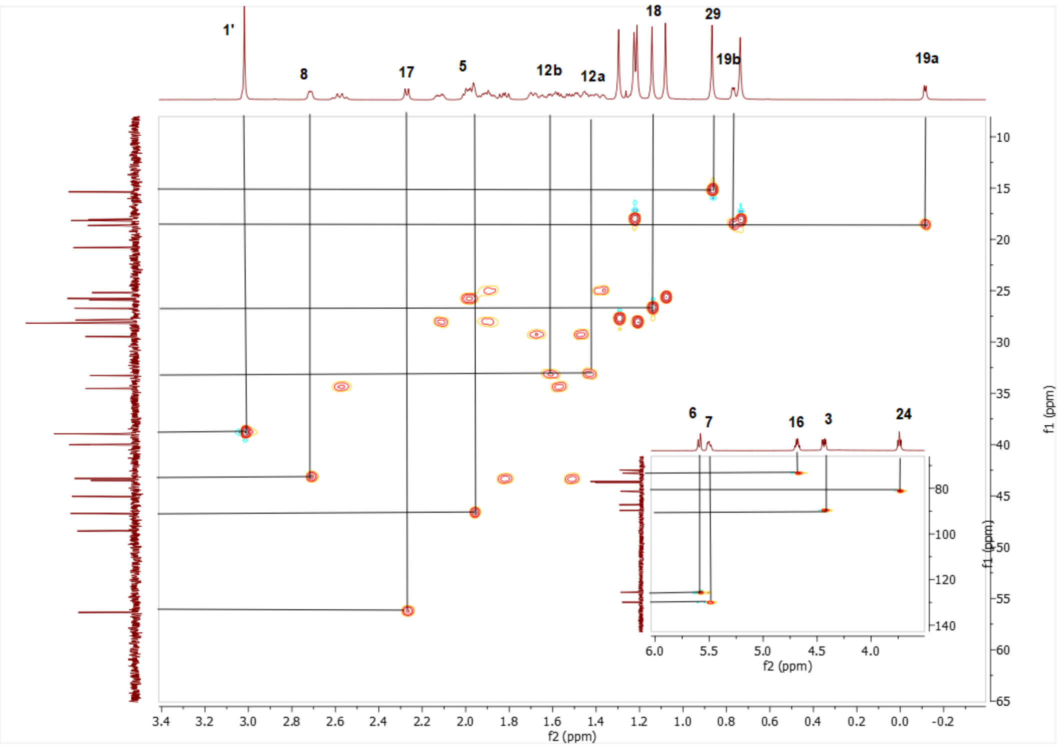

Spectrum 59. HMQC spectrum of CG-04.

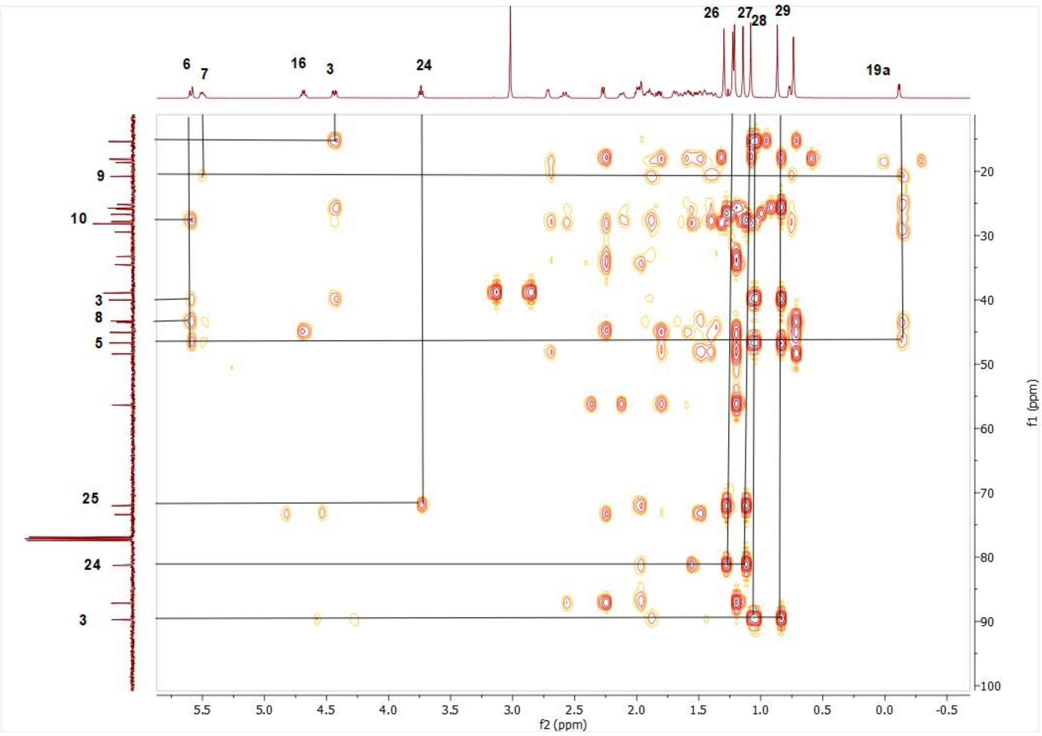

Spectrum 60. HMBC spectrum of CG-04.

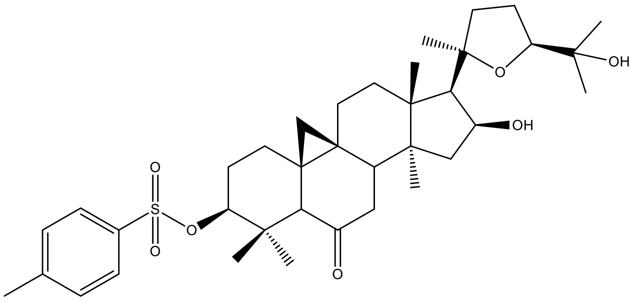

Supplementary Figure 12. Chemical Structure of CG-05

Supplementary Table 10. The ^{13}C and ^{1}H NMR data of CG-05 (100/400 MHz, δ ppm, in CDCl3).

H/C	δ_C (ppm)	δ_H (ppm), *J* (Hz)
**1**	30.0 t	1.42 m, 1.76 m
**2**	27.3 t	1.79 m, 1.94 m
**3**	89.3	4.21 m
**4**	40.0 s	-
**5**	57.2 d	2.3 brs
**6**	210 s	-
**7**	41.2 t	2.12 m, 2.17 m

**8**	42.4 d	2.66 dd (8.5, 4)
**9**	21.7 s	-
**10**	29.7 s	-
**11**	26.5 t	1.46 m, 1.85 m
**12**	33.0	1.47 m, 1.6 m
**13**	47.1 s	-
**14**	45.3 s	-
**15**	43.8 t	1.37 m, 1.84 m
**16**	72.9 d	4.69 ddd (7.8, 7.8, 6.1)
**17**	56.9 d	2.32 d (7.6)
**18**	18.4 q	1.21 m
**19**	22.2 t	0.21, 0.6 d (5.5)
**20**	87.1 s	-
**21**	28.1	1.21 s
**22**	34.5 t	1.57 m, 2.57 q (10.8)
**23**	25.91 t	1.98 m
**24**	81.26 d	3.75 dd (8.3, 6.1)
**25**	72.1 s	-
**26**	26.8 q	1.14 s
**27**	27.8 q	1.3 s
**28**	26.3 q	1.02 s
**29**	14.8 q	1.0 s
**30**	19.1 q	0.89 s
**1’**	134.7 s	-
**2’**	127.8 d	7.79 d (8.3)
**3’**	129.8 d	7.3 d (8.1)
**4’**	144.6 s	-
**5’**	129.8 d	7.3 d (8.1)
**6’**	127.8 d	7.79 d (8.3)
**7’**	21.8 q	2.43 s

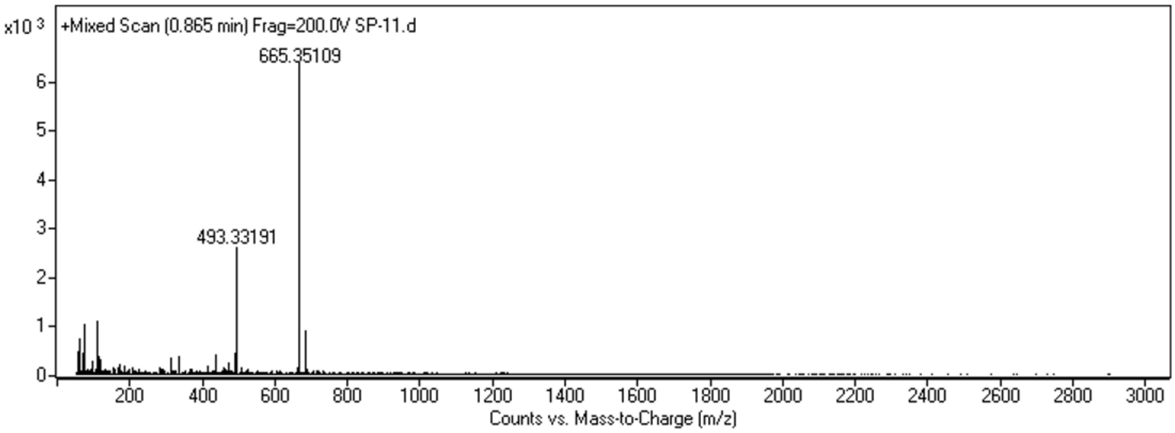

Spectrum 61. HR-ESI-MS Spectrum of CG-05 (positive mode).

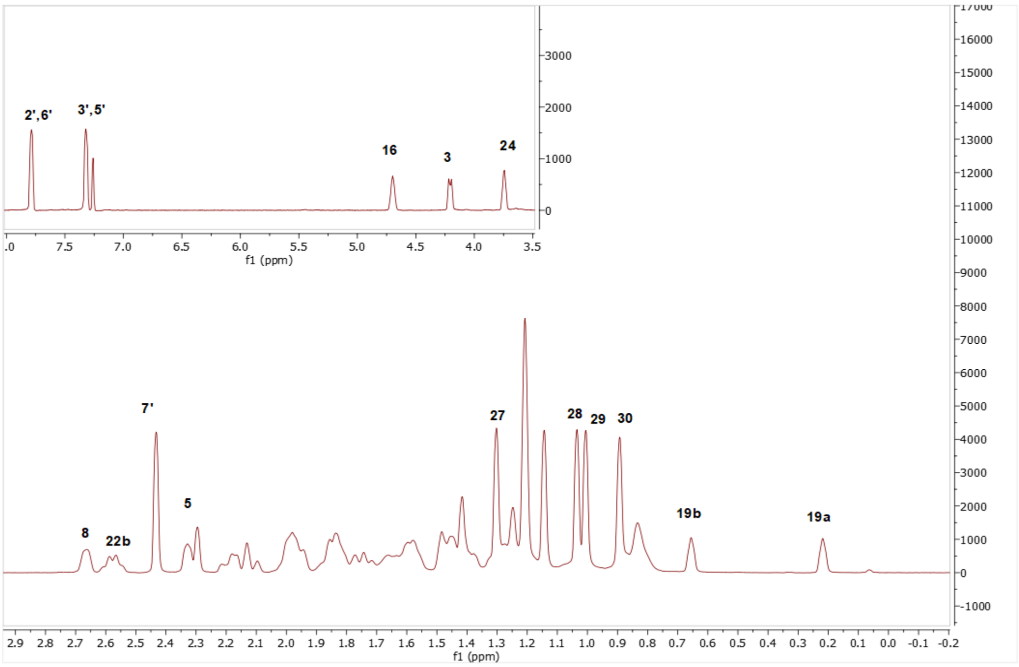

Spectrum 62. ^{1}H NMR Spectrum of CG-05.

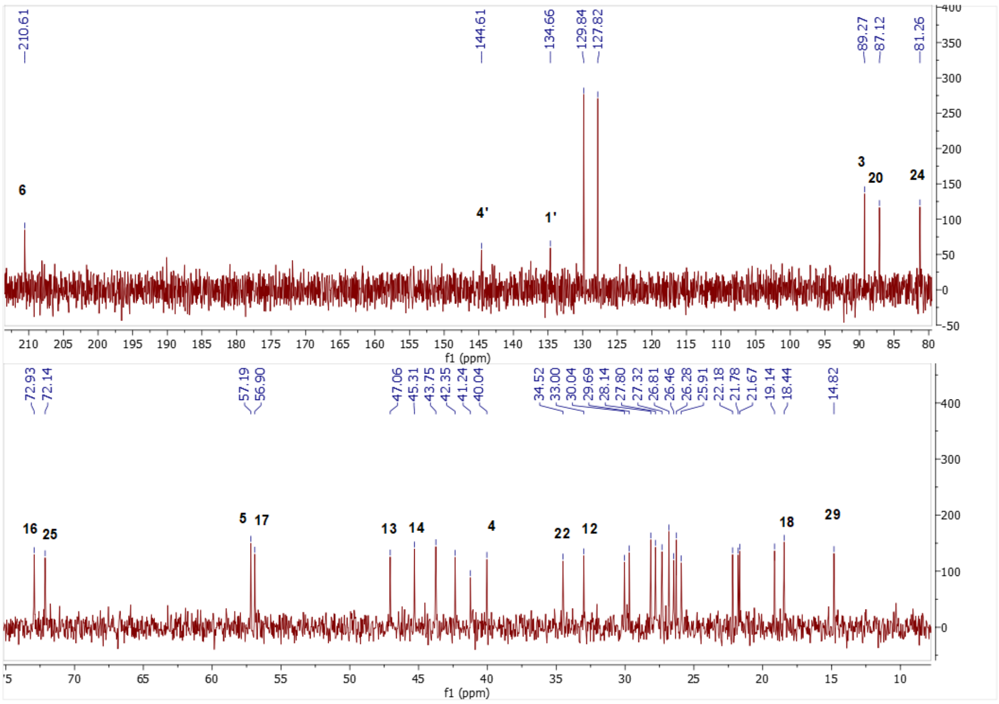

Spectrum 63. ^{13}C NMR Spectrum of CG-05.

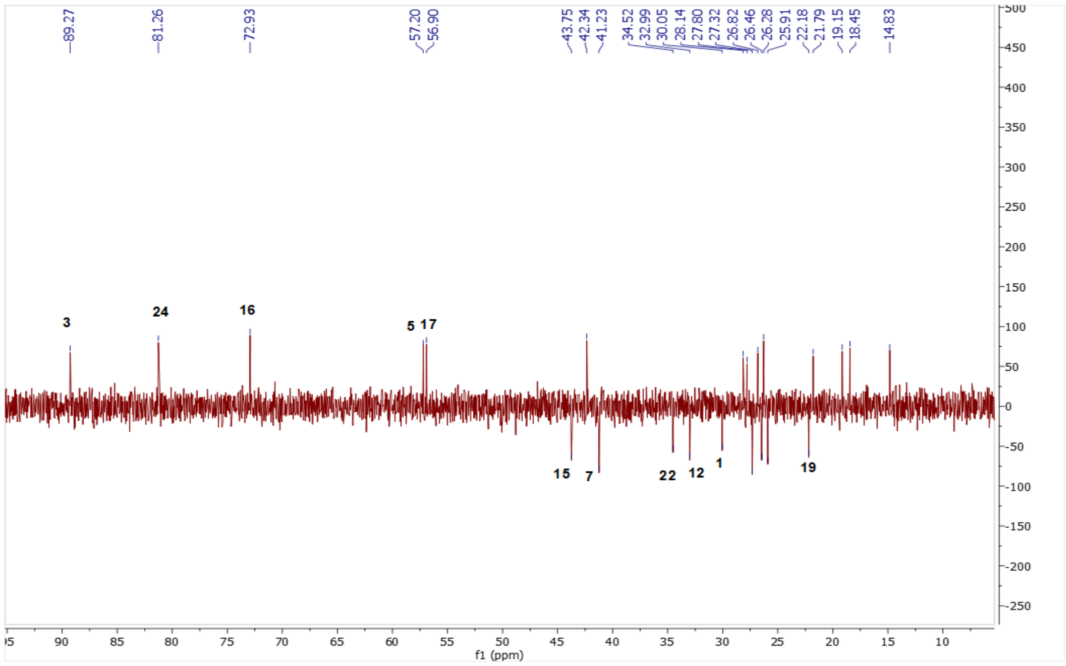

Spectrum 64. DEPT135 spectrum of CG-05.

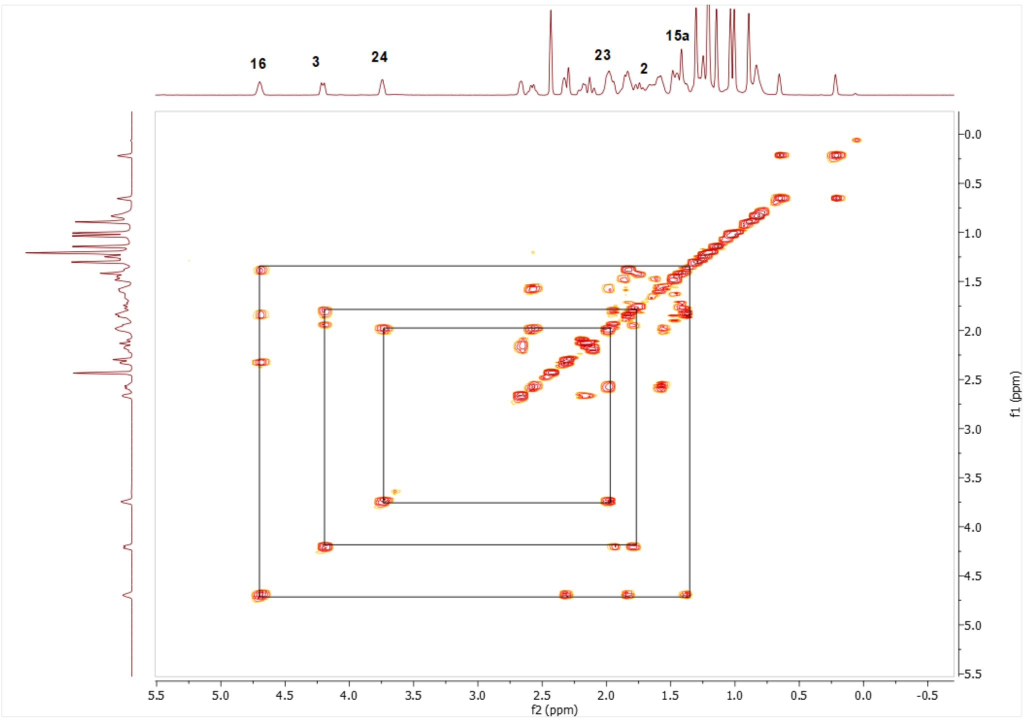

Spectrum 65. COSY spectrum of CG-05.

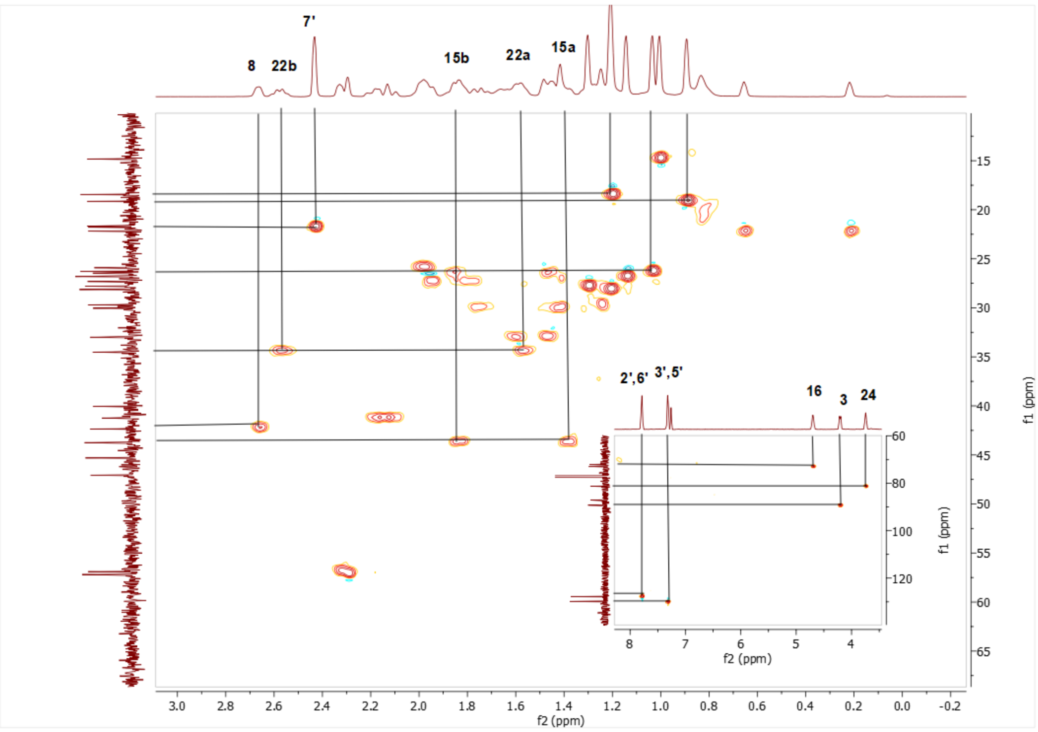

Spectrum 66. HMQC spectrum of CG-05.

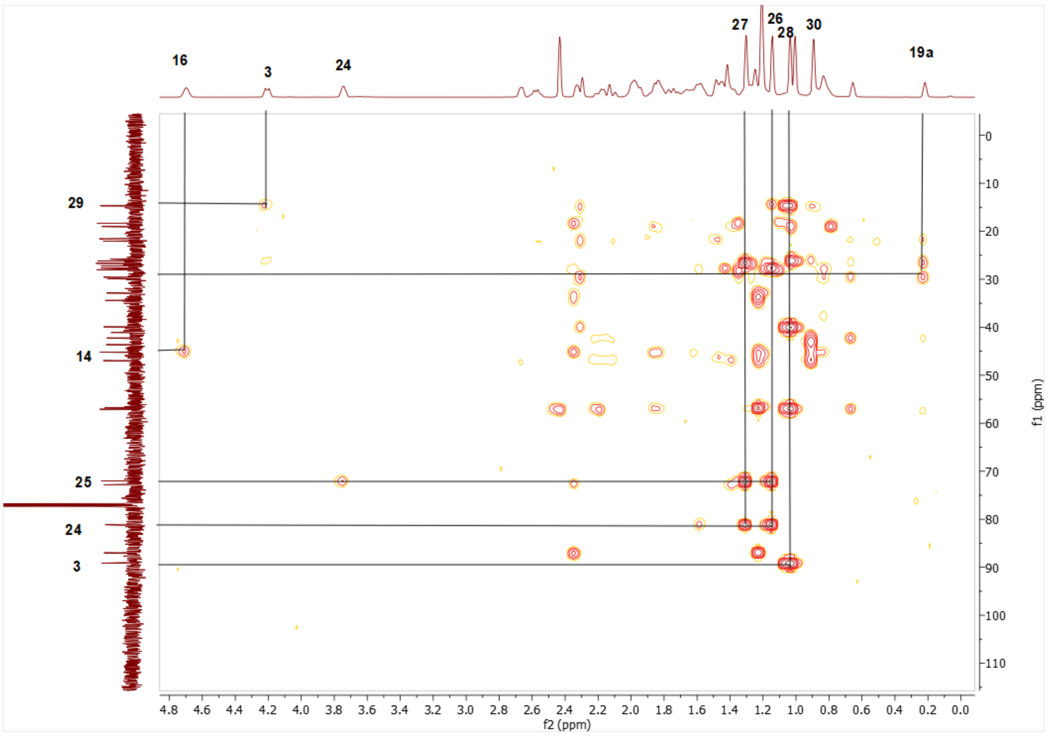

Spectrum 67. HMBC spectrum of CG-05.

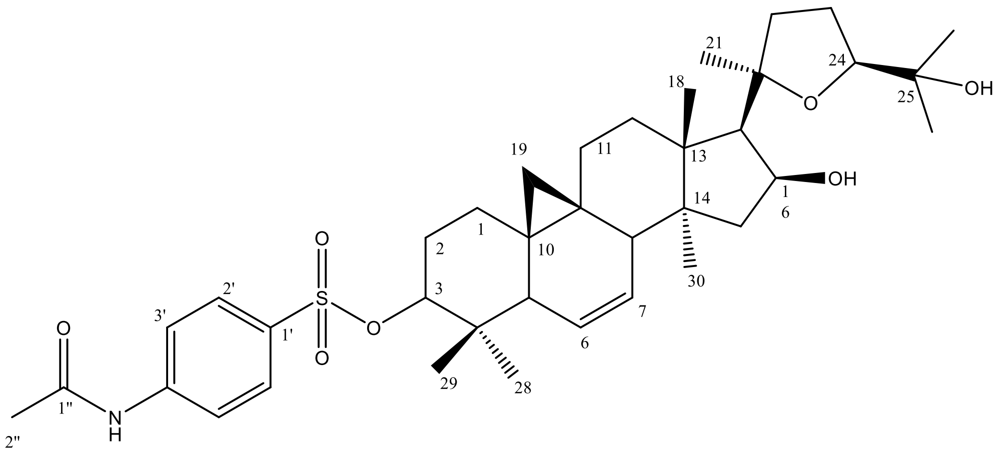

Supplementary Figure 13. Chemical Structure of CG-06

Supplementary Table 11. The ^{13}C and ^{1}H NMR data of CG-06 (100/400 MHz, δ ppm, in CDCl3).

H/C	δ_C (ppm)	δ_H (ppm), *J* (Hz)
**1**	29.5 t	1.37 m, 1.56 m
**2**	27.7 t	1.77 m, 1.87 m
**3**	90.4 d	4.26 dd (11.8, 4.7)
**4**	40.0 s	-
**5**	46.6 d	1.85 m
**6**	125.6 d	5.5 d (10.7)
**7**	129.7 d	5.43 ddd (0.2, 6.2, 2.9)
**8**	43.2 d	2.66 m
**9**	20.7 s	-
**10**	27.8 s	-
**11**	25.1 t	1.33 m, 1.85 m
**12**	33.2 t	1.40 m, 1.57 m
**13**	48.3 s	-
**14**	45.0 s	-
**15**	43.4 t	1.48 m, 1.78 m
**16**	73.4 d	4.67 q (4.2)
**17**	56.3 d	2.26 d (7.5)
**18**	18.1 q	1.18 s
**19**	18.6 t	-0.18, 0.69 d (4.1)
**20**	87.1 s	-
**21**	28.2 q	1.18 s
**22**	34.5 t	1.56 m, 2.57 q (10.8)
**23**	25.9 t	1.98 m
**24**	81.3 d	3.73 t (6.9)
**25**	72.0 s	-
**26**	27.7 q	1.27 s
**27**	26.7 q	1.13 s
**28**	25.4 q	0.8 s
**29**	15.3 q	0.78 s
**30**	18.1 q	0.69 s
**1’**	131.8 s	-

**2’**	129.0 d	7.82 d (8.5)
**3’**	119.3 d	7.3 d (8.5)
**4’**	143.2 s	-
**5’**	119.3 d	7.3 d (8.5)
**6’**	129.0 d	7.82 d (8.5)
**1’’**	169.3	-
**2’’**	24.7 s	2.17 s

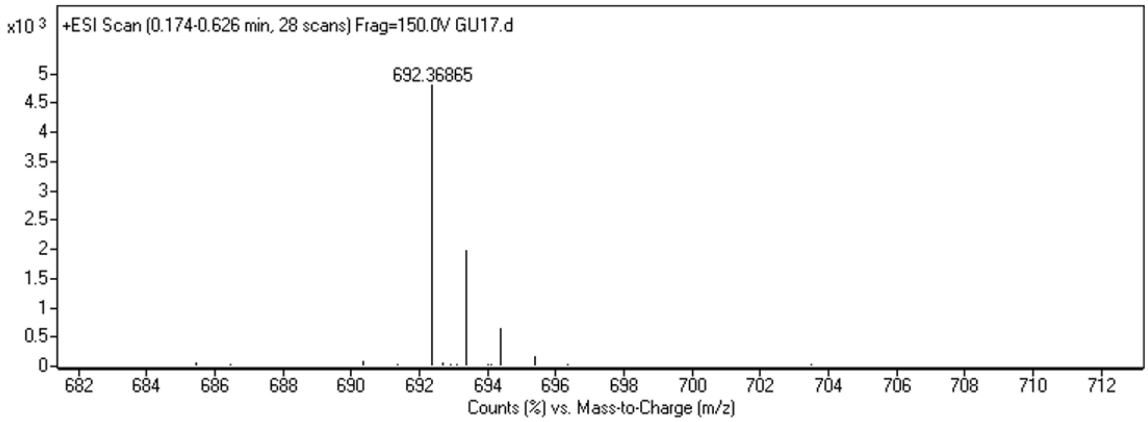

Spectrum 68. HR-ESI-MS Spectrum of CG-0*6* (positive mode).

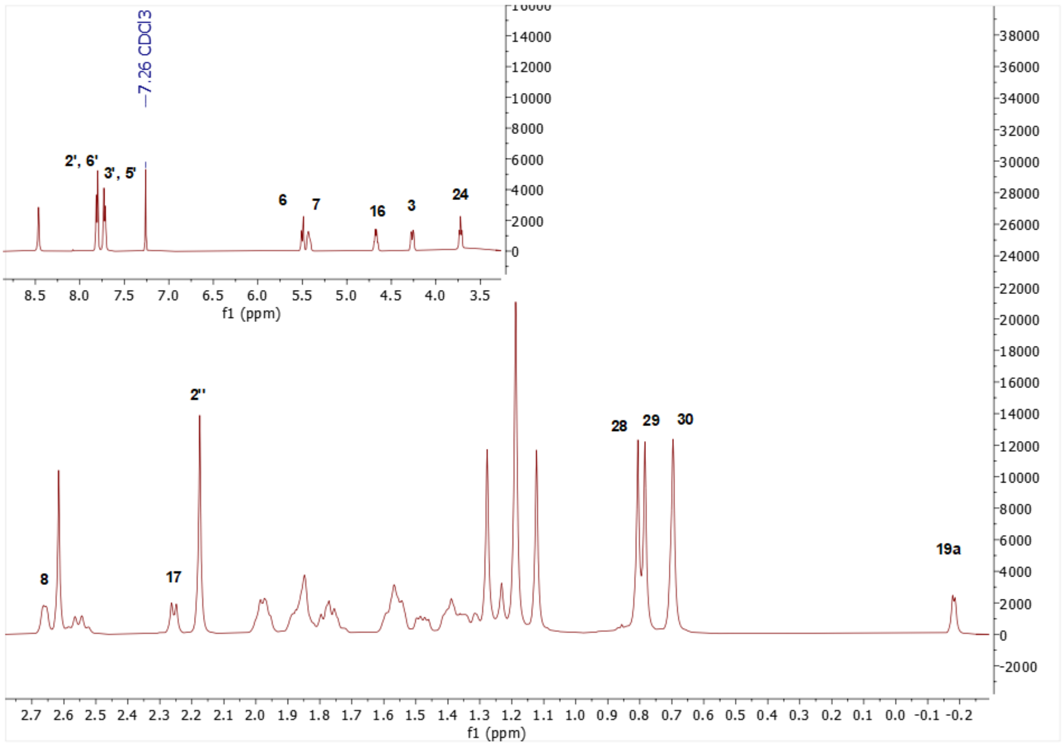

Spectrum 69. ^{1}H NMR Spectrum of CG-06.

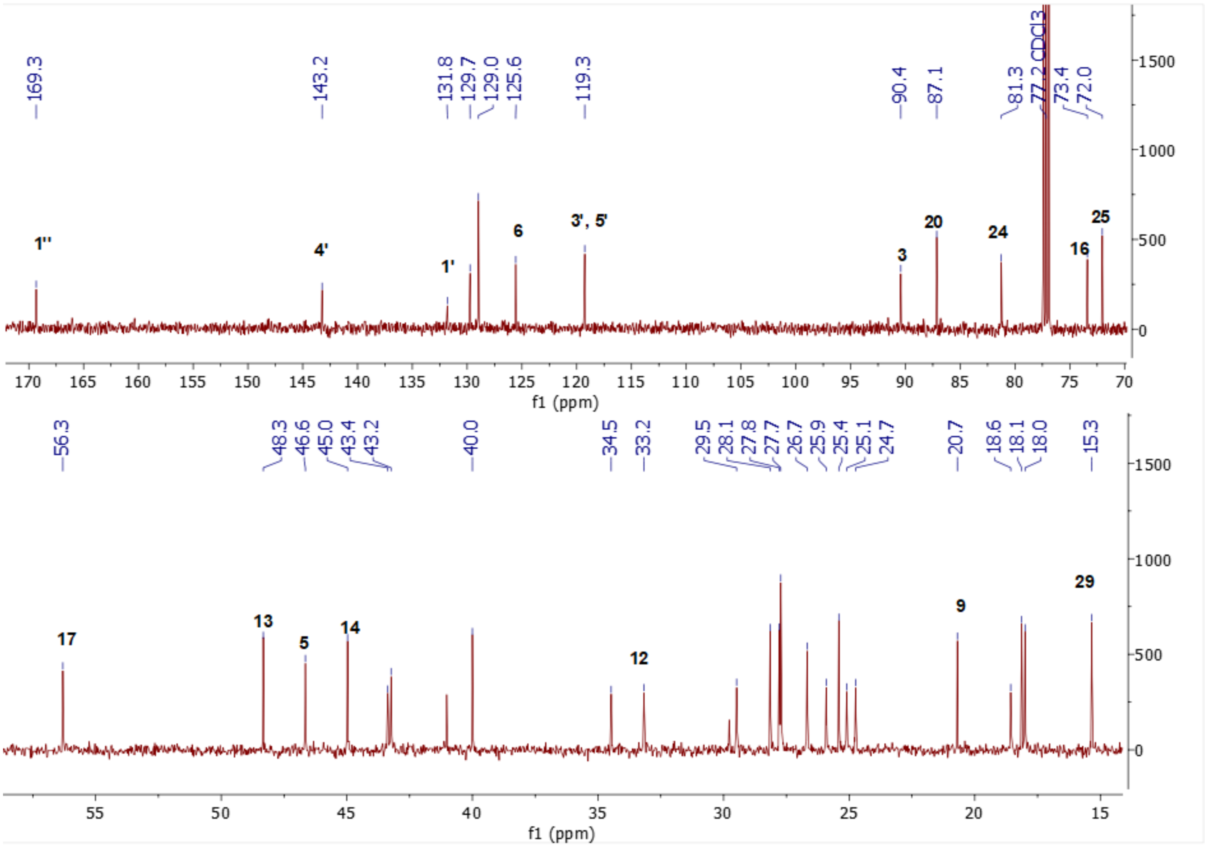

Spectrum 70. ^{13}C NMR Spectrum of CG-06.

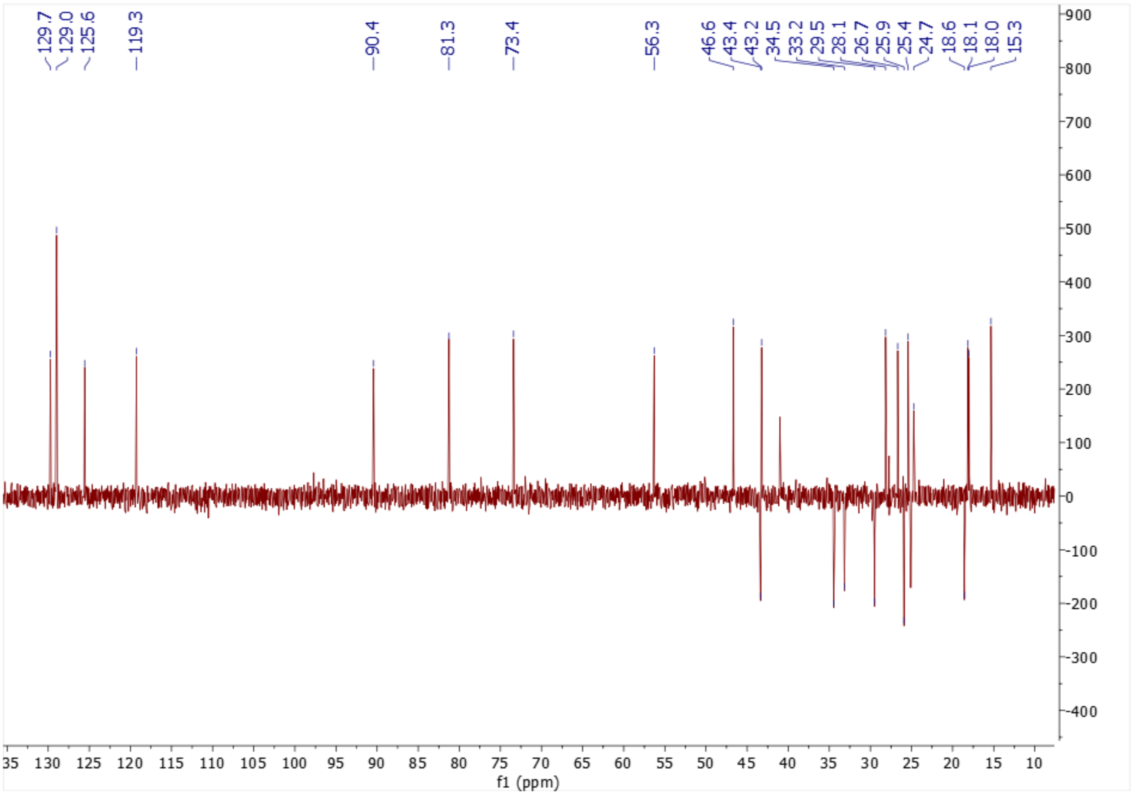

Spectrum 71. DEPT135 spectrum of CG-06

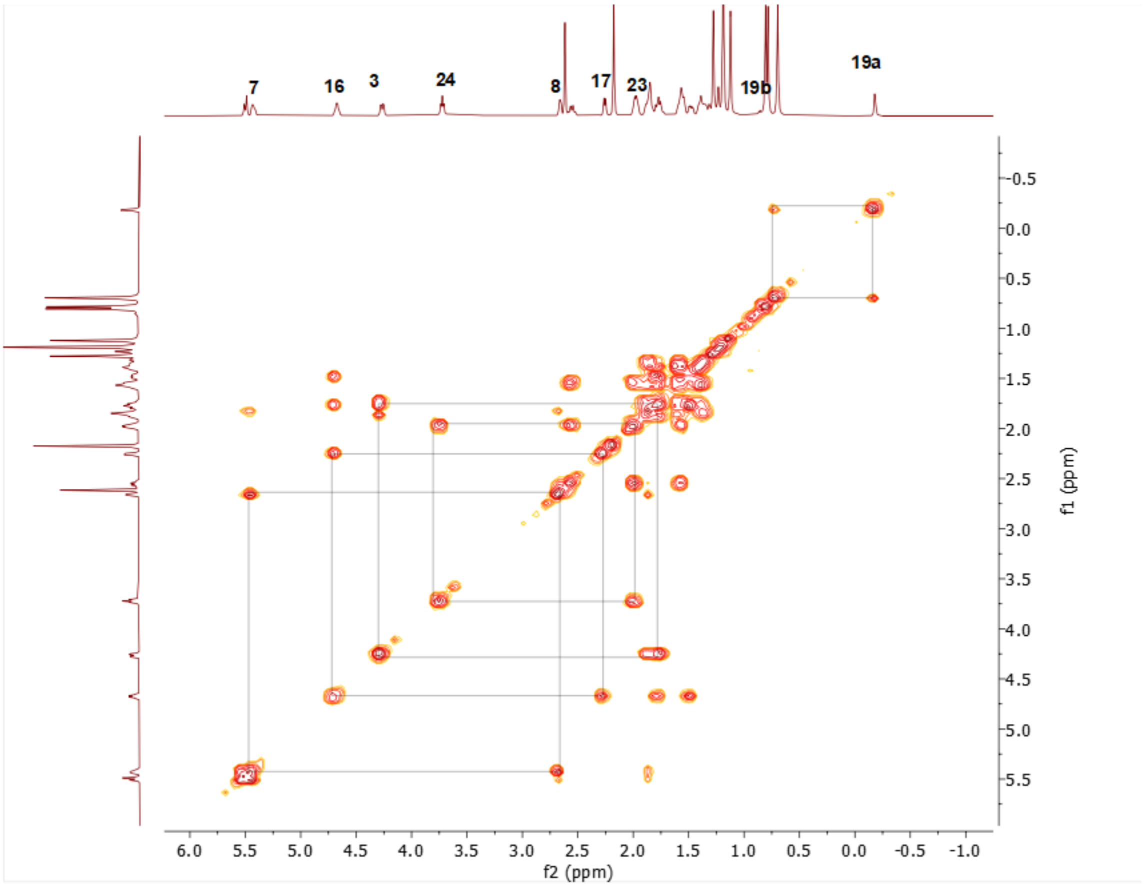

Spectrum 72. COSY spectrum of CG-06

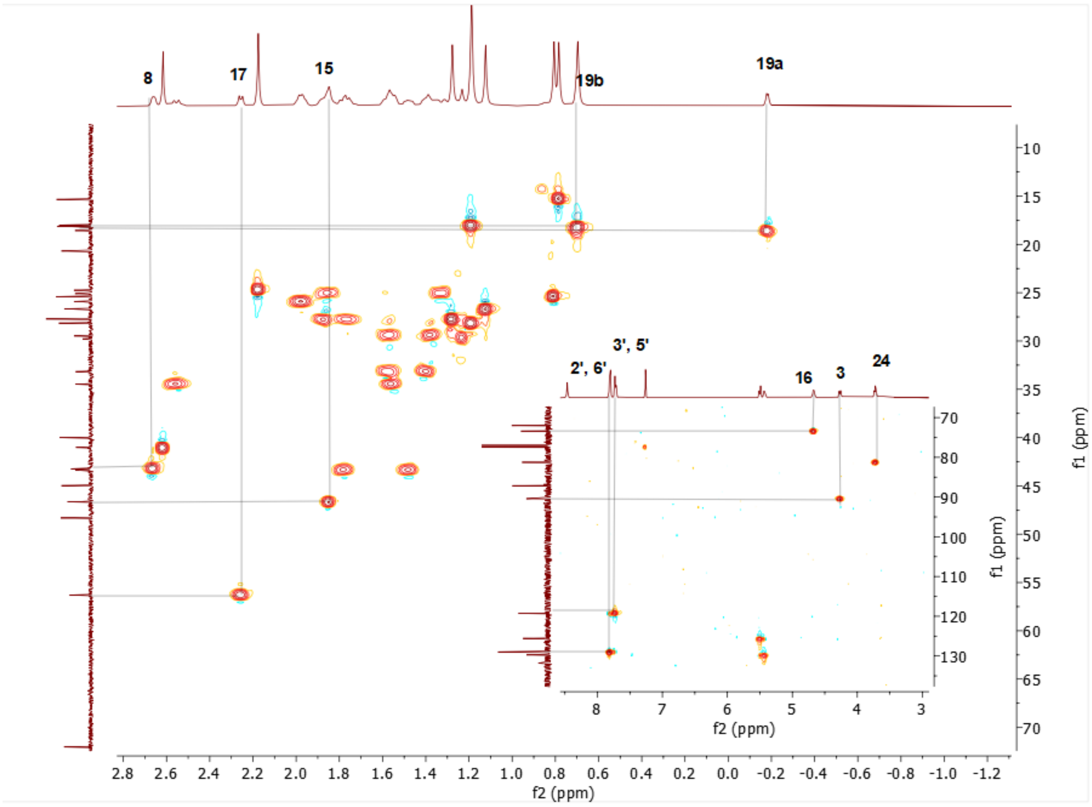

Spectrum 73. HMQC spectrum of CG-06.

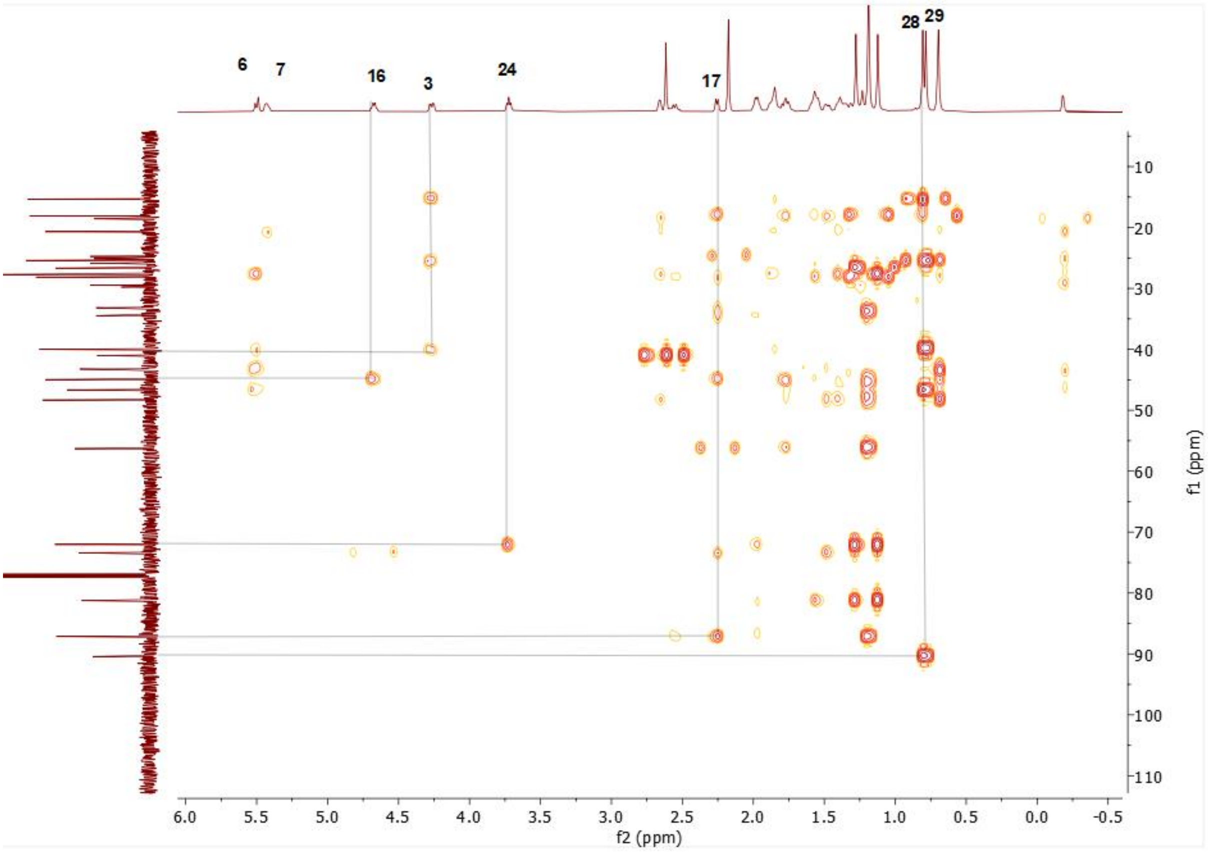

Spectrum 74. HMBC spectrum of CG-05.

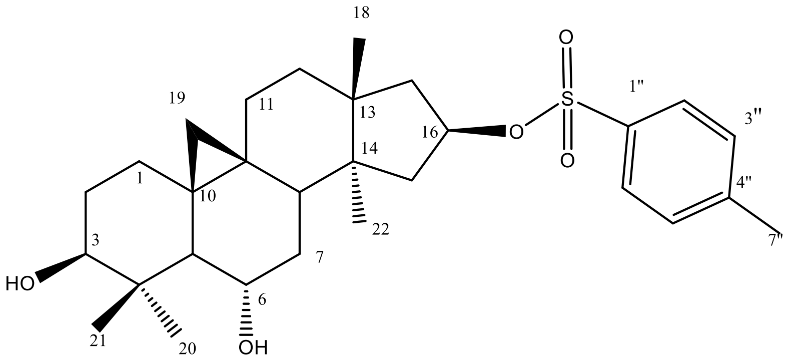

Supplementary Figure 14. Chemical Structure of SCG-01

Supplementary Table 12. The ^{13}C and ^{1}H NMR data of SCG-01 (100/400 MHz, δ ppm, in CDCl3).

**H/C**	**δ_C (ppm)**	**δ_H (ppm), *J* (Hz)**
**1**	32.0 t	1.2 d (3.2), 1.57 m
**2**	30.3 t	1.56 m, 1.78 m

**3**	78.4 d	3.29 dd (11.3, 4.6)
**4**	41.6 s	-
**5**	53.5 d	1.34 d (1.97)
**6**	68.5 d	3.51 ddd (9.1, 9.1, 4.2)
**7**	37.5 t	1.3 m, 1.44 m
**8**	45.8 d	1.6 m
**9**	20.8 s	-
**10**	29.8 s	-
**11**	26.1 t	1.25 m, 1.92 m
**12**	30.3 t	1.4 m, 1.6 m
**13**	44.5 s	-
**14**	45.9 s	-
**15**	46.0 t	1.88 m
**16**	82.9 d	5.06 ddd (15.4, 7.9, 1.4)
**17**	44.4 t	1.86 m, 1.64 d (1.43)
**18**	25.0 q	0.95 s
**19**	30.0 t	0.28 d (4.6), 0.45 d (4.6)
**28**	28.0 q	1.22 s
**29**	15.3 q	0.92 s
**30**	19.8 q	1.04 s
**1’**	134.4 s	-
**2’**	127.8 d	7.75 d (8.2)
**3’**	129.9 d	7.31 d (8.2)
**4’**	144.6 s	-
**5’**	129.9 d	7.31 d (8.2)
**6’**	127.8 d	7.75 d (8.2)
**7’**	21.8 q	2.44 s

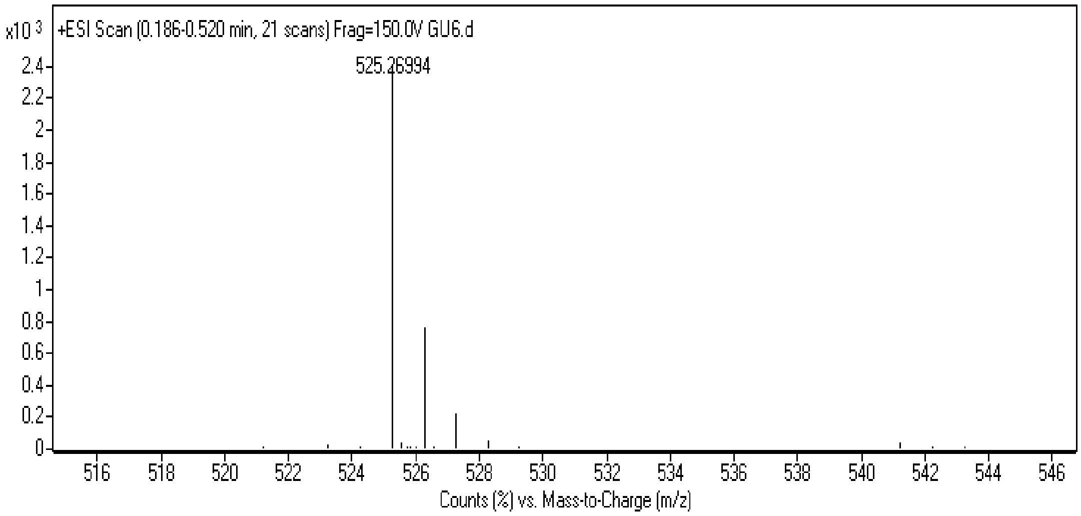

Spectrum 75.HR-ESI-MS Spectrum of SCG-01 (positive mode).

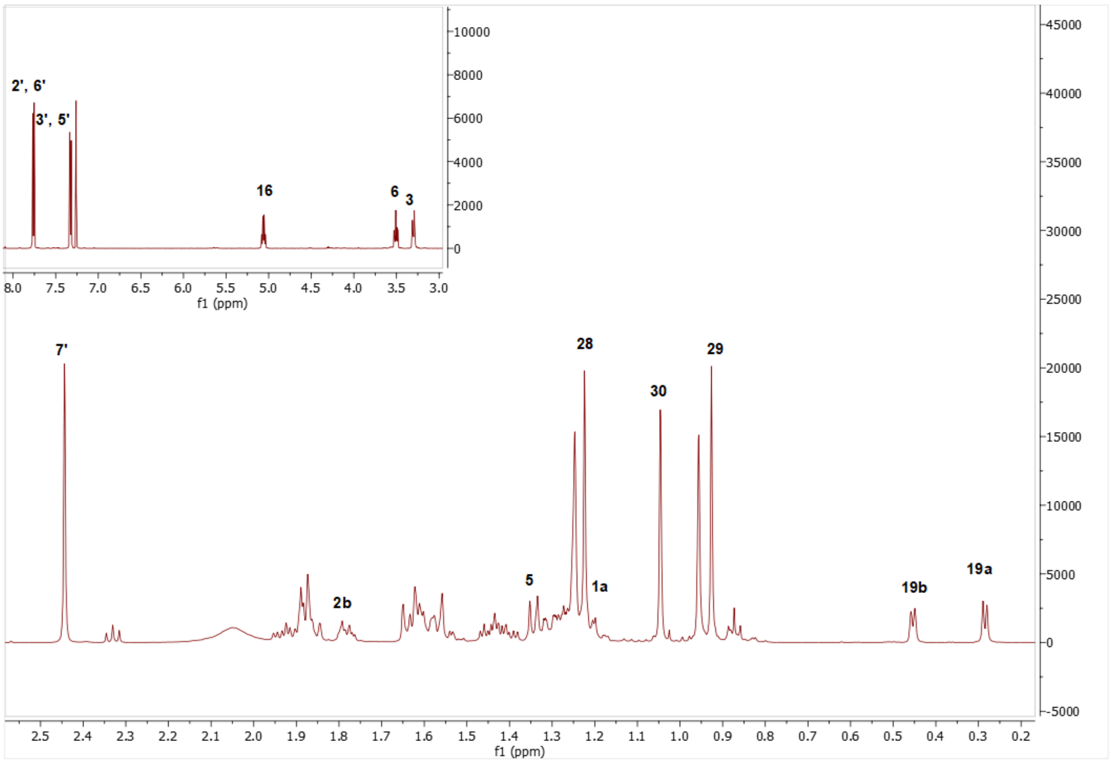

Spectrum 761. ^{1}H NMR Spectrum of SCG-01

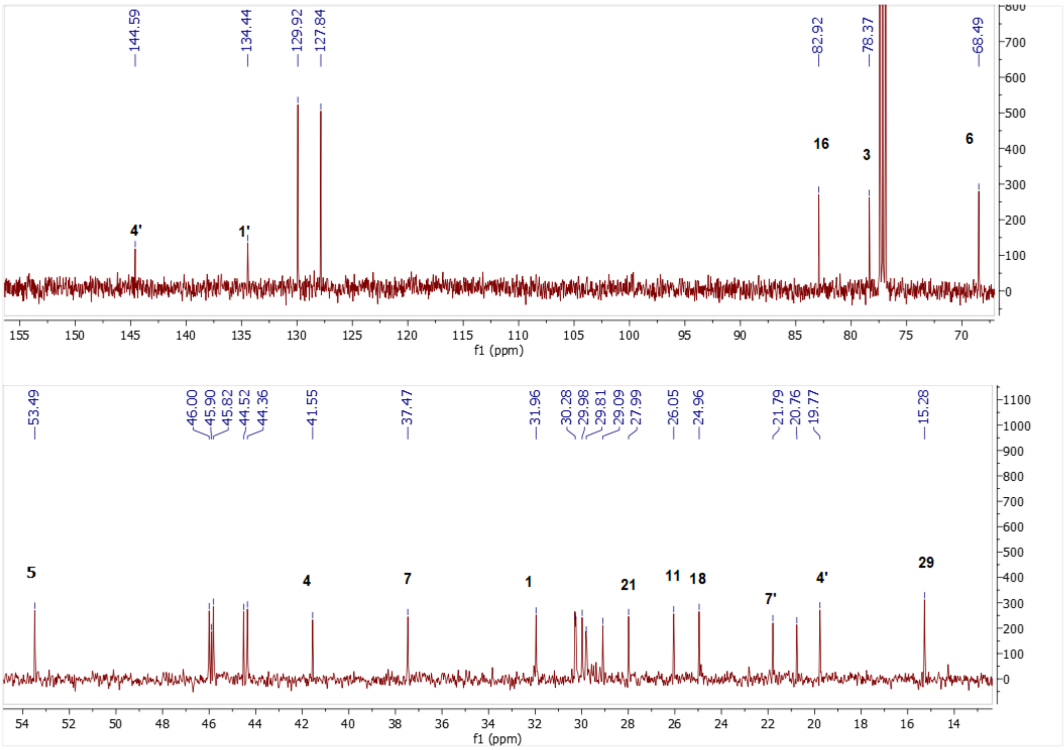

Spectrum 77. ^{13}C NMR Spectrum of SCG-01.

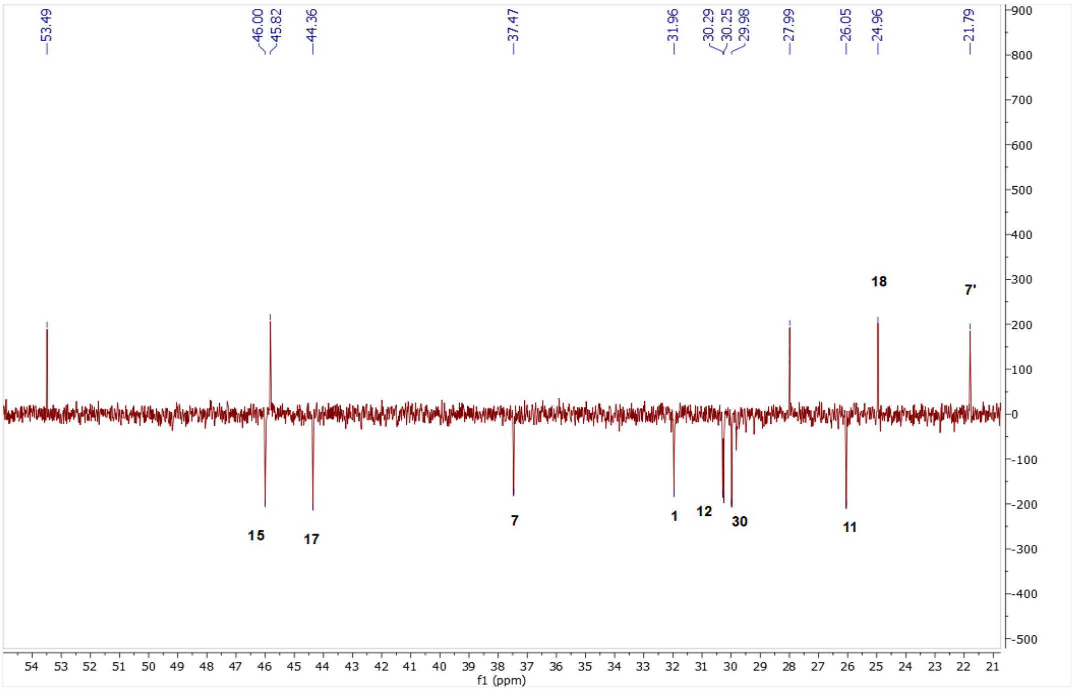

Spectrum 78. DEPT135 spectrum of SCG-01.

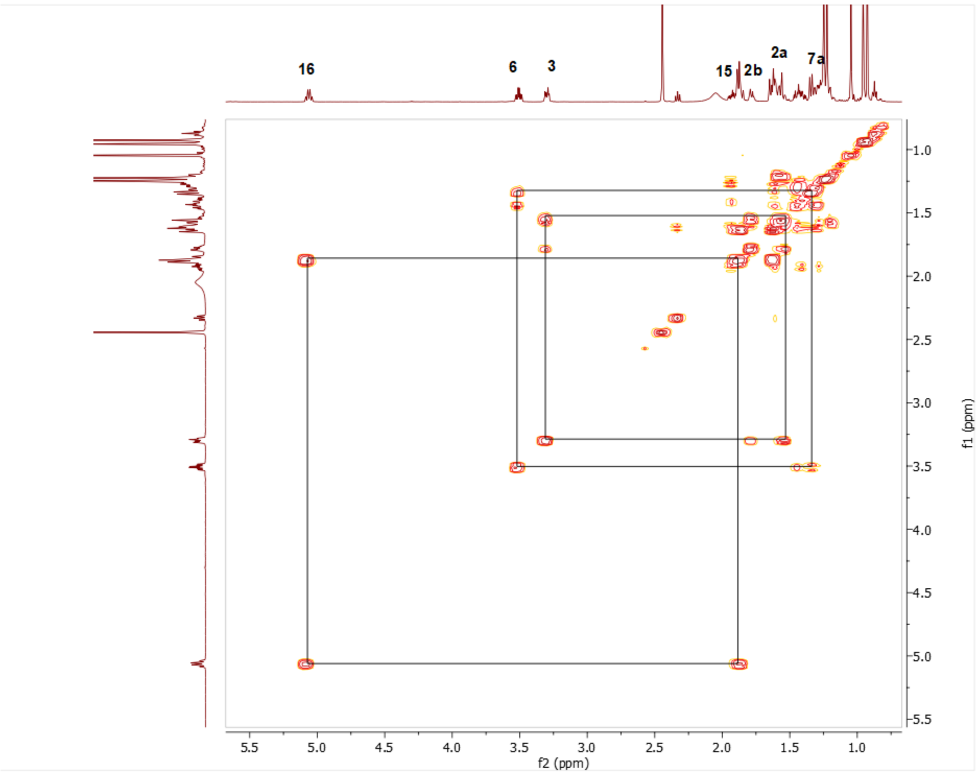

Spectrum 79. COSY spectrum of SCG-01.

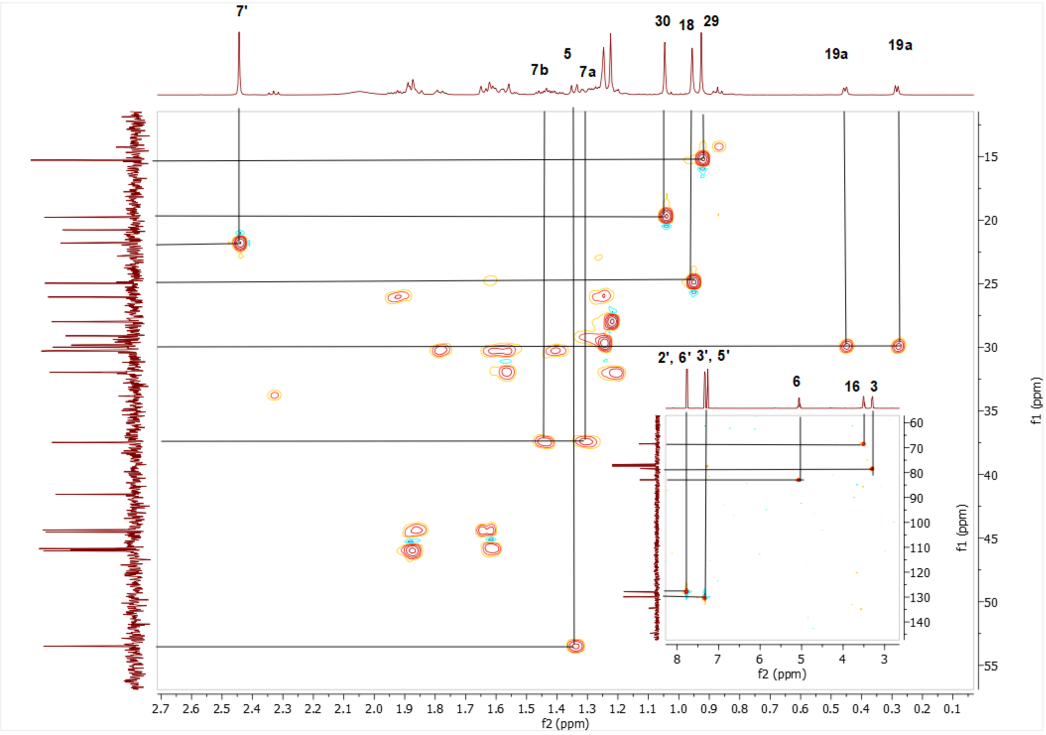

Spectrum 80. HMQC spectrum of SCG-01.

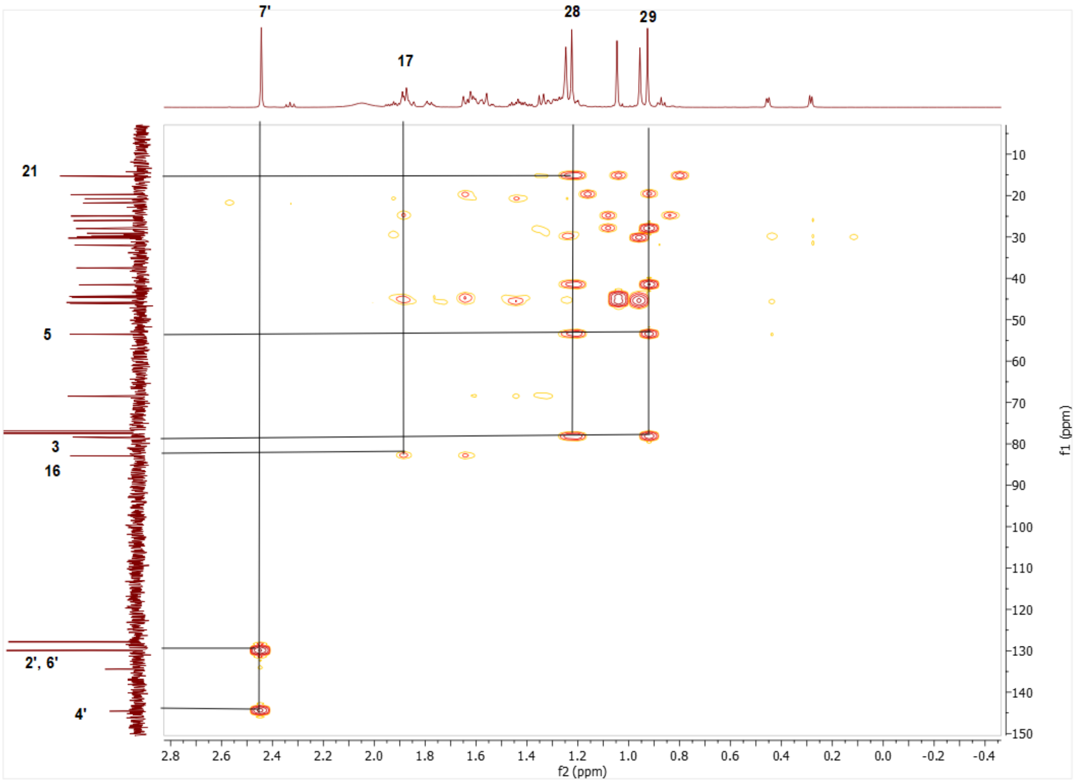

Spectrum 81. HMBC spectrum of SCG-01.

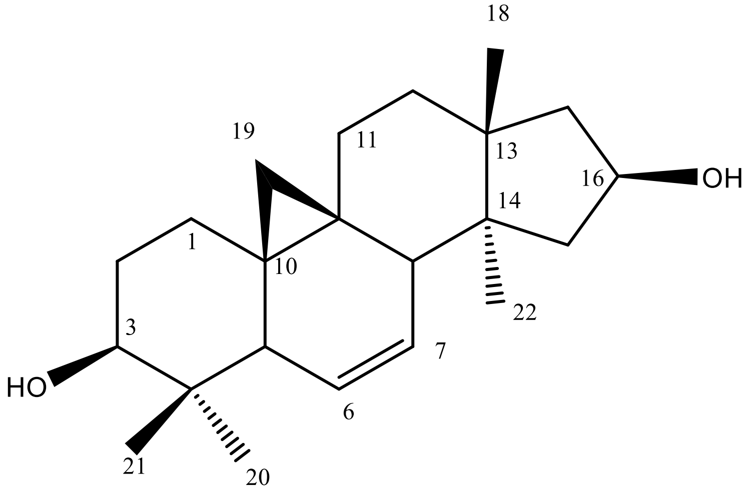

Supplementary Figure 15. Chemical Structure of SCG-02

Supplementary Table 13. The ^{13}C and ^{1}H NMR data of SCG-02 (100/400 MHz, δ ppm, in CDCl3).

H/C	δ_C (ppm)	δ_H (ppm), *J* (Hz)
**1**	29.8 t	1.25 m, 1.46 m
**2**	30.1 t	1.6 m, 1.82 m
**3**	78.5 d	3.3 dd (11.2, 4.4)
**4**	40.3 s	-
**5**	46.5 d	1.89 m
**6**	126.3 d	5.62 d (10.5)
**7**	129.1 d	5.44 ddd (10.6, 6.1, 3.2)
**8**	43.7 d	2.48 dd (6.2, 2.6)
**9**	21.2 s	-
**10**	28.3 s	-
**11**	25.2 t	1.42 m, 1.85 m
**12**	31.4 t	1.22 m, 1.69 m
**13**		
	48.5 s	-
**14**	45.3 s	-
**15**	45.4 t	1.27 m, 2.05 dd (13.7, 8.2)
**16**	72.2 d	4.55 ddd (14.5, 7.7, 1.4)
**17**	47.8 t	1.86 m, 1.62 m
**18**	22.2 q	0.96 s
**19**	18.5 t	-0.15 d (4.1), 0.73 d (4.5)
**28**	25.6 q	1.05 s
**29**	14.5 q	0.77 s
**30**	18.4 q	0.92 s

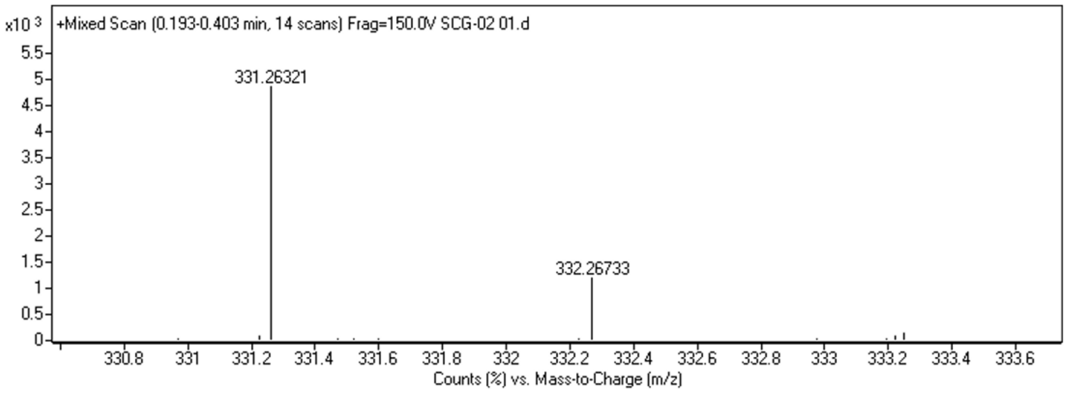

Spectrum 82. HR-ESI-MS Spectrum of SCG-02 (positive mode).

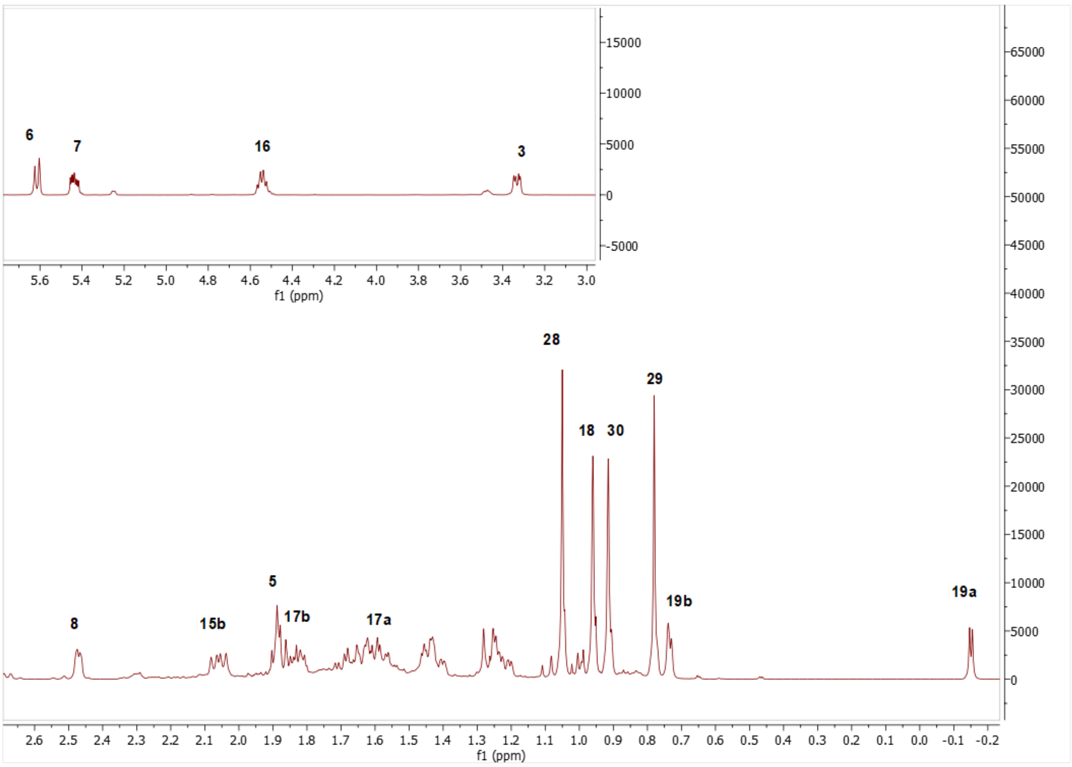

Spectrum 83. ^{1}H NMR Spectrum of SCG-02.

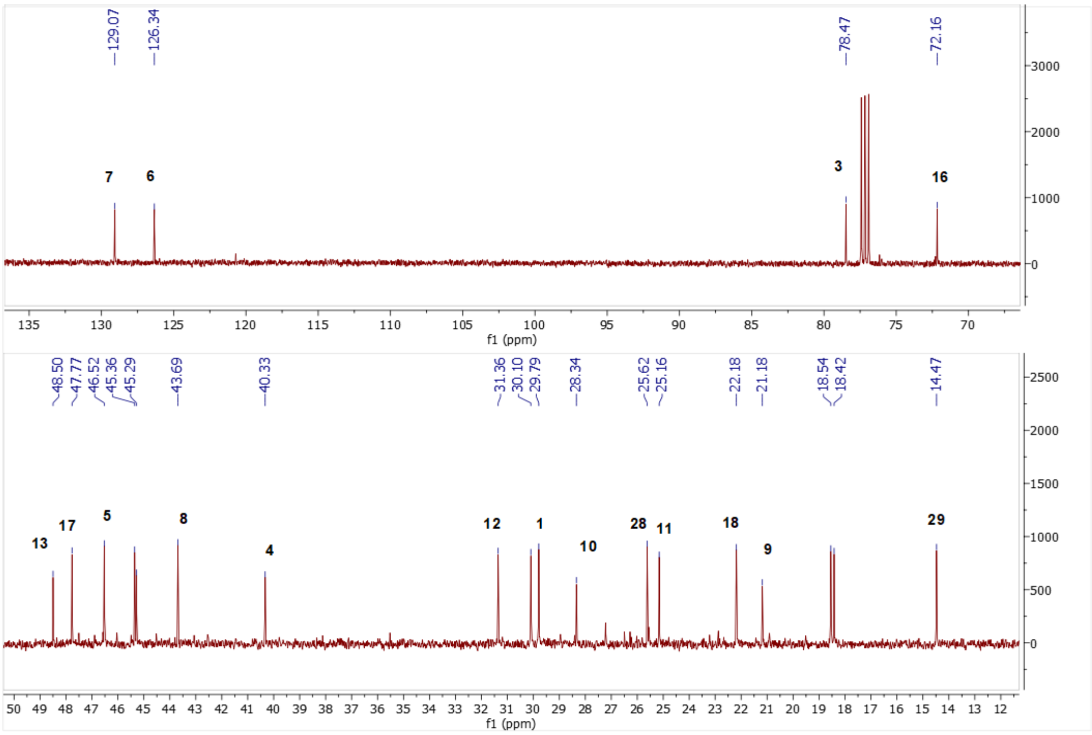

Spectrum 84. ^{13}C NMR Spectrum of SCG-02.

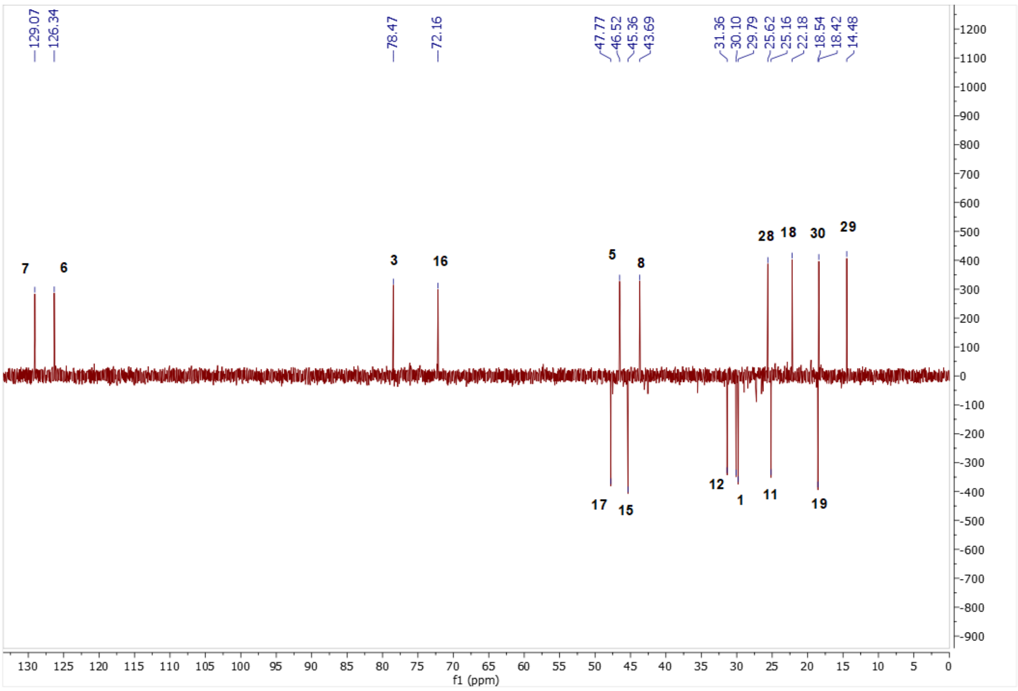

Spectrum 85. DEPT135 spectrum of SCG-02.

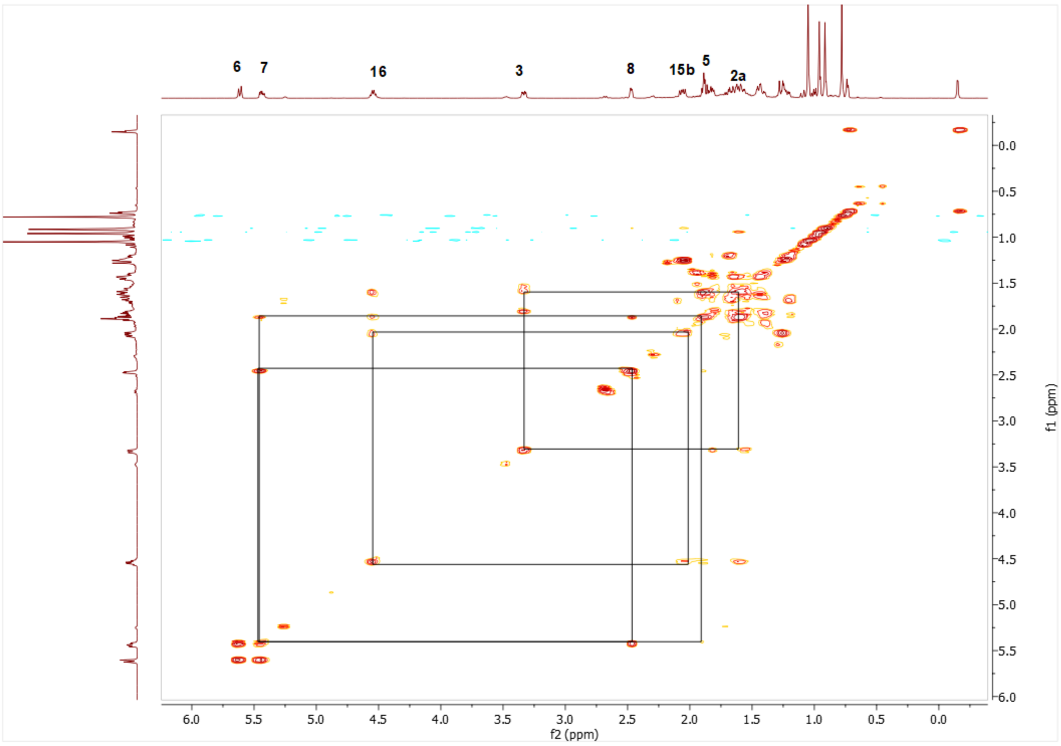

Spectrum 86. COSY spectrum of SCG-02.

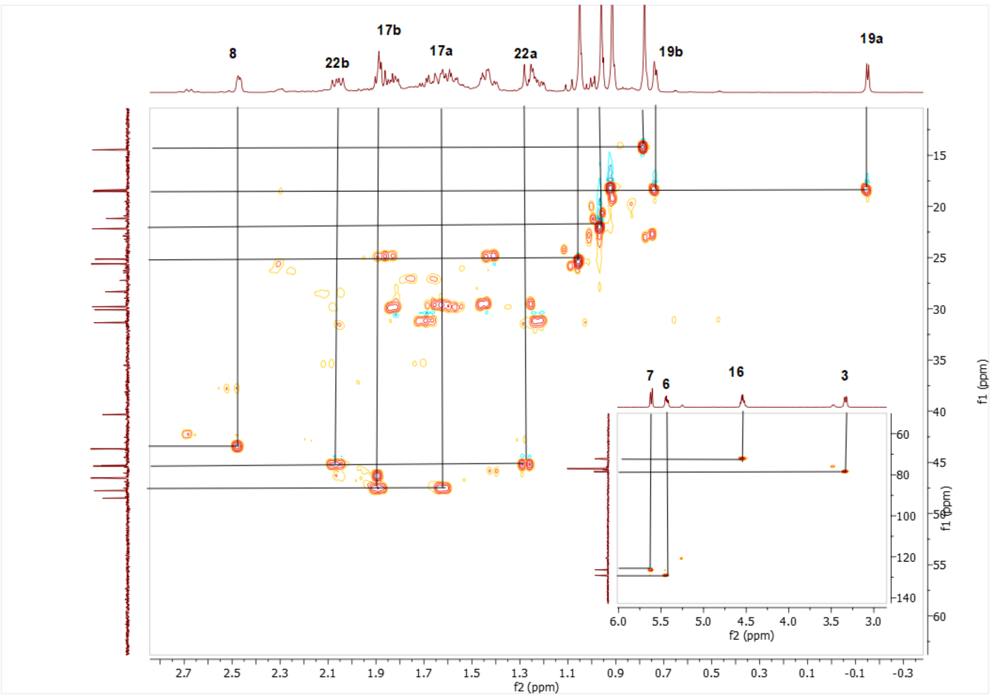

Spectrum 87. HSQC spectrum of SCG-02.

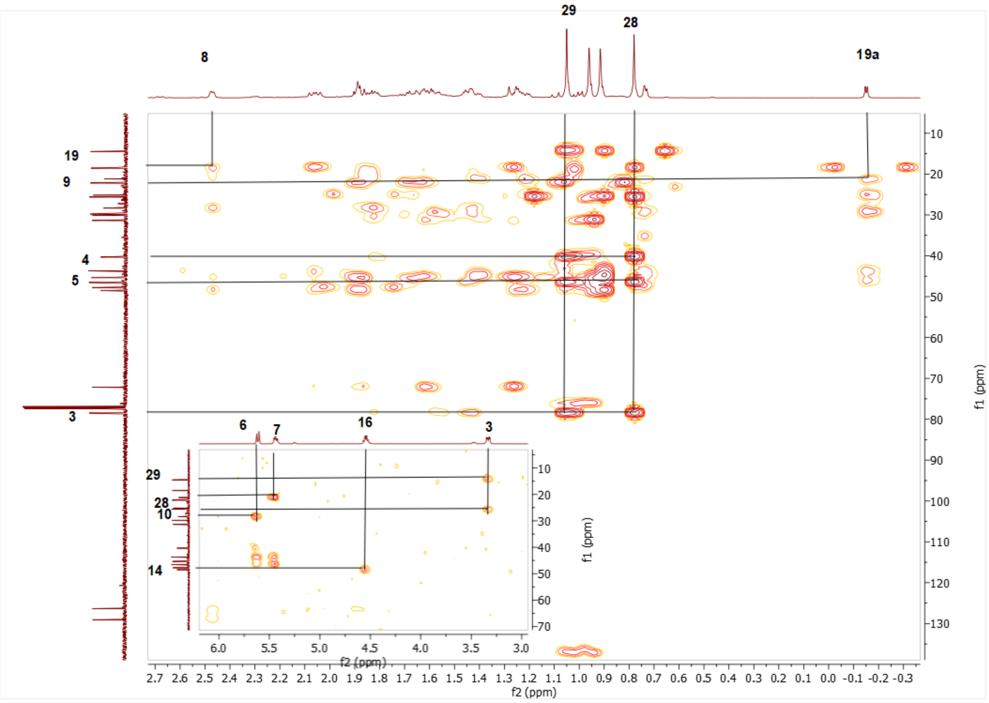

Spectrum 882. HMBC spectrum of SCG-02.

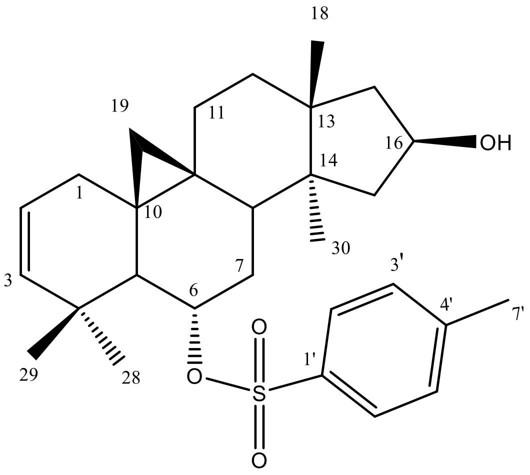

Supplementary Figure 16. Chemical Structure of SCG-03

Supplementary Table 14. The ^{13}C and ^{1}H NMR data of SCG-03 (100/400 MHz, δ ppm, in CDCl3).

**H/C**	**δC (ppm)**	**δH (ppm), *J* (Hz)**
**1**	34.9 t	1.39 m, 2.30 m
**2**	122.8 d	5.49 ddd (9.9, 5.8, 2)
**3**	140.7	5.3 dd (9.8, 2.7)
**4**	38.0 s	-
**5**	52.6 d	1.58 m
**6**	83 d	5.04 q (7.4)

**7**	28.4 t	1.34 m, 1.90 m
**8**	47.9 d	1.54 m
**9**	19.2 s	-
**10**	28.2 s	-
**11**	25.8 t	1.1 m, 2.07 m
**12**	30.16	1.47 m, 1.62 m
**13**	44.4 s	-
**14**	45.9 s	-
**15**	44.9 t	1.65 m, 1.87 m
**16**	70.7 d	3.45 td (9.8, 4.7)
**17**	46.1 t	1.88 m
**18**	25.7 q	0.99 s
**19**	31.7 q	0.34 d (4.5), 0.51 d (5)
**28**	33.1 q	1.23 s
**29**	23.5 q	1.05 s
**30**	20.2 q	1.03 s
**1’**	134.2 s	-
**2’**	127.8 d	7.74 dd (8.2, 3.6)
**3’**	129.9 d	7.31 dd (8.2, 3.6)
**4’**	144.6 s	-
**5’**	129.9 d	7.31 dd (8.2, 3.6)
**6’**	127.8 d	7.74 dd (8.2, 3.6)
**7’**	21.8 q	2.44 s

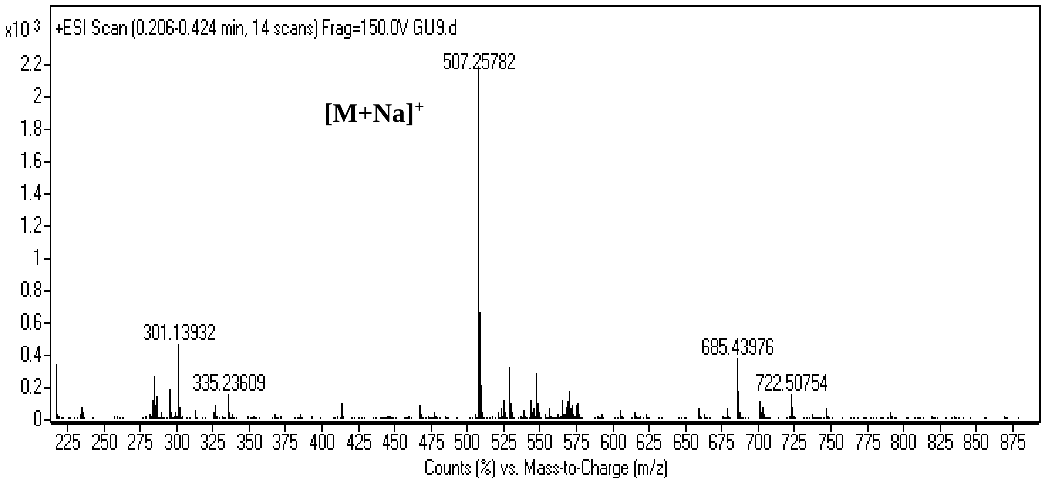

Spectrum 89. HR-ESI-MS Spectrum of SCG-03 (positive mode).

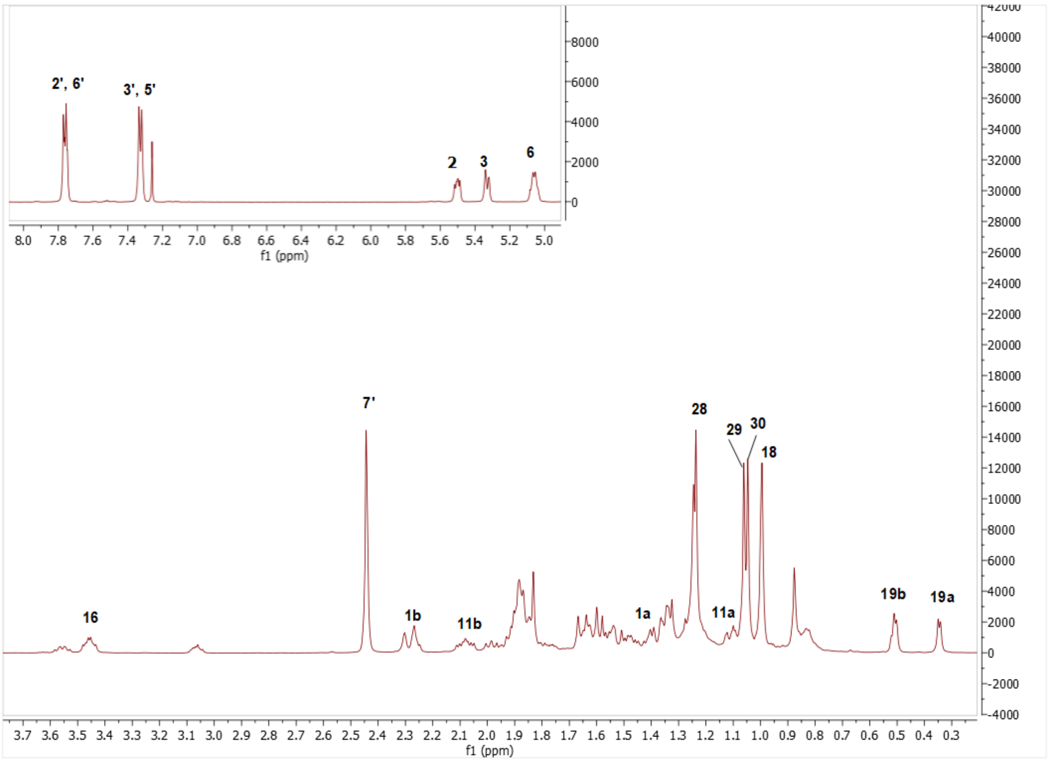

Spectrum 90. ^{1}H NMR Spectrum of SCG-03.

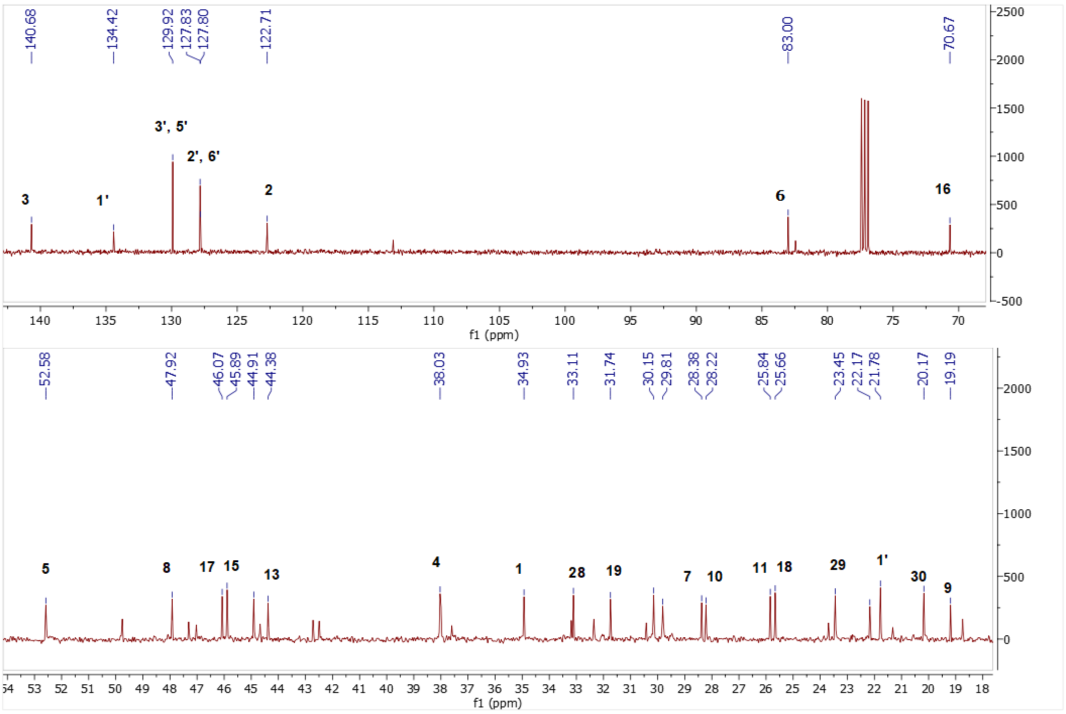

Spectrum 91. ^{13}C NMR Spectrum of SCG-03.

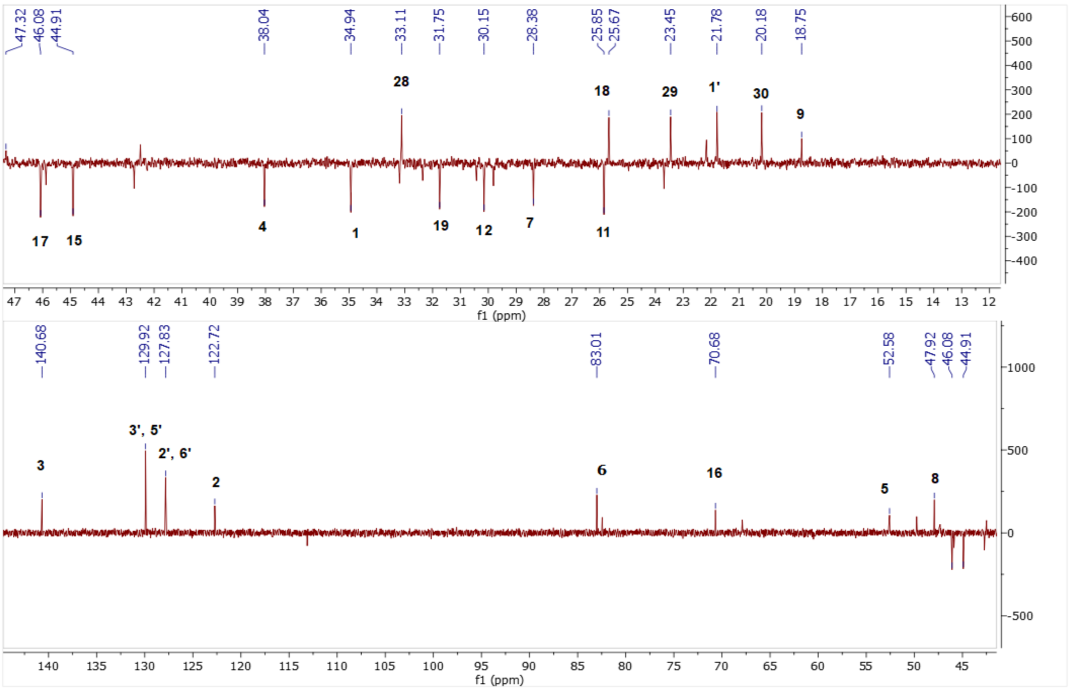

Spectrum 92. DEPT135 spectrum of SCG-03.

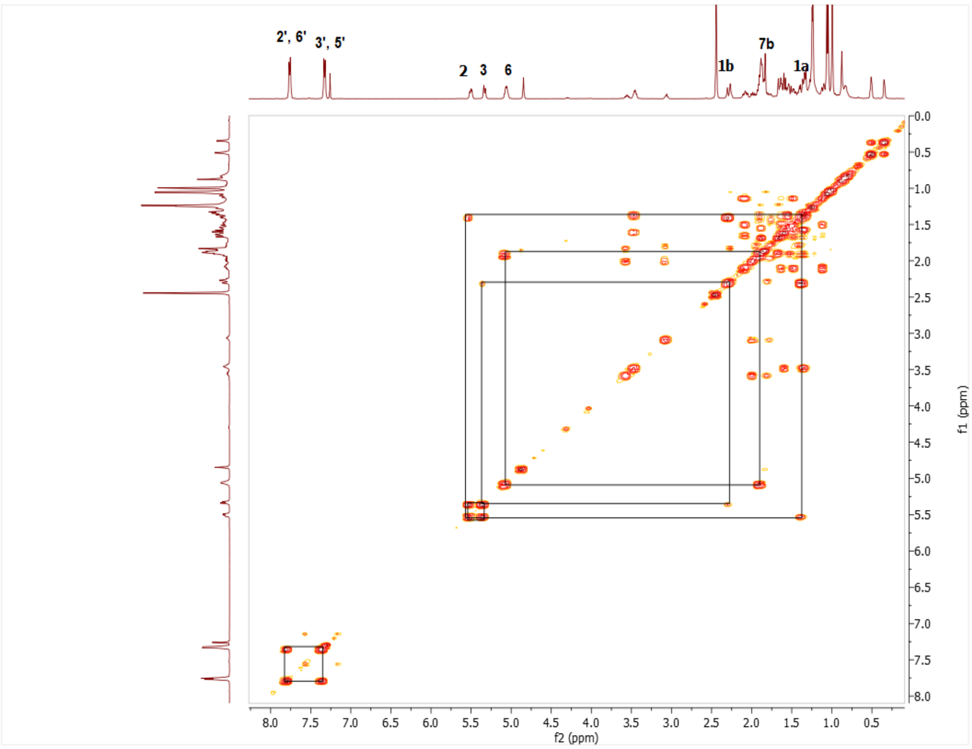

Spectrum 93. COSY spectrum of SCG-03.

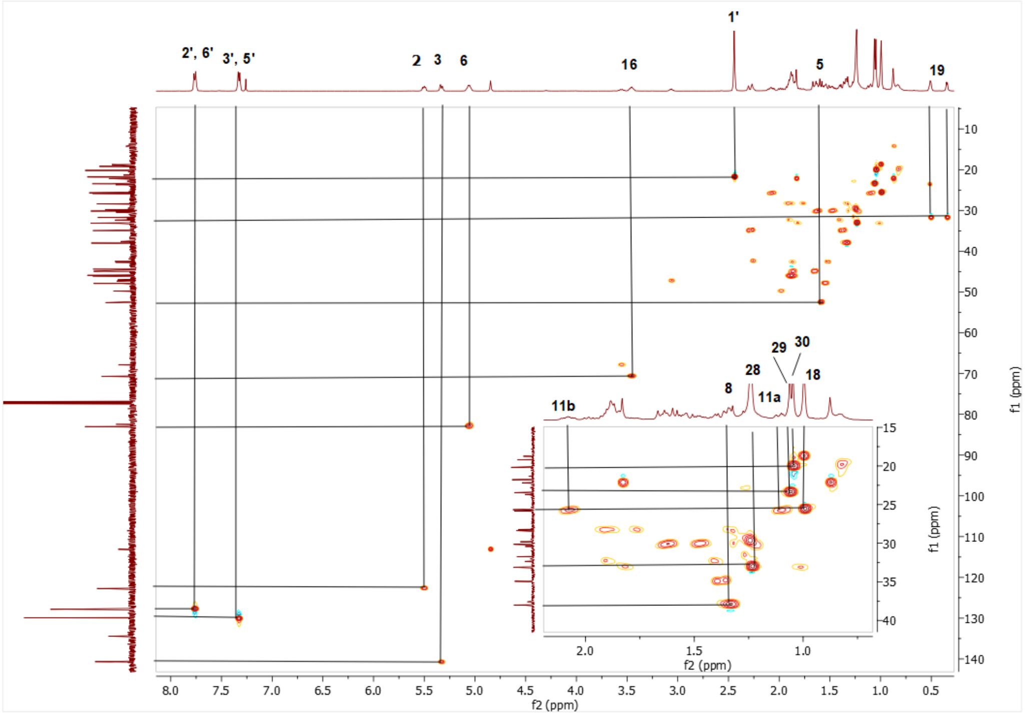

Spectrum 94. HMQC spectrum of SCG-03.

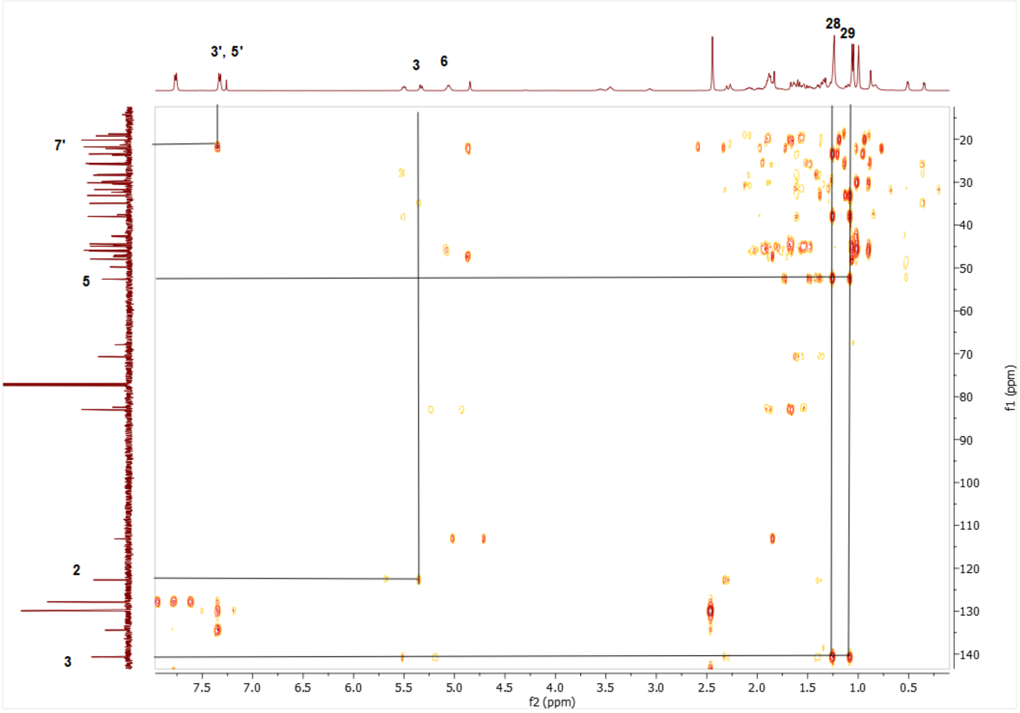

Spectrum 95. HMBC spectrum of SCG-03.

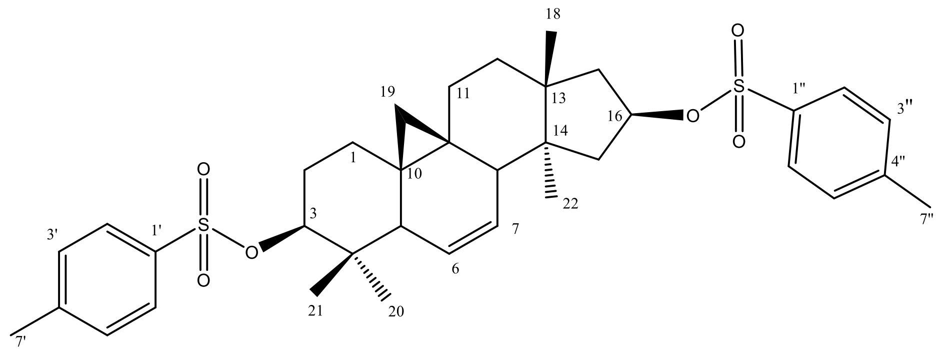

Supplementary Figure 17. Chemical Structure of SCG-04

Supplementary Table 15. The ^{13}C and ^{1}H NMR data of SCG-04 (100/400 MHz, δ ppm, in CDCl3).

H/C	δC (ppm)	δH (ppm), *J* (Hz)
**1**	29.4 t	1.4 m,1.6 m
**2**	27.6 t	1.8 m, 1.9 m
**3**	89.9 d	4.3 dd (11.6, 4.5)
**4**	39.8 s	-
**5**	46.5 d	1.89 m
**6**	125,7 d	5.52 d (10.7)
**7**	128.9 d	5.36 ddd (9.6, 5.9, 2.9)
**8**	43.1 d	2.41 m
**9**	20.9 s	-
**10**	27.7 s	-
**11**	24.79	1.8 m, 1.39 m
**12**	30.7	1.16 m, 1.61
**13**	44.4 s	-
**14**	48.1 s	-
**15**	44.9 t	1.81 m
**16**	82.4 d	5.06 q (7.5)
**17**	41.9 t	2 m, 1.5 m
**18**	21.8 q	0.88 s
**19**	18.3 t	-0.16 d (4.1), 0.71 d (4.1)
**28**	25.3 q	0.83 s
**29**	15.2 q	0.79 s
**30**	17.6 q	0.82 s
**1'**	134.7* s	-
**2'**	127.7 d	7.75+ d (7.8)
**3'**	129.7' d	7.32 d (7.8)
**4'**	144.4- s	-
**5'**	129.7' d	7.32 d (7.8)
**6'**	127.7 d	7.75+ d (7.8)
**7'**	21.6 q	2.44 s
**1''**	134.2* s	-
**2''**	127.7 d	7.8+ d (7.7)

**3’’**	129.7’ d	7.32 d (7.8)
**4’’**	144.7- s	-
**5’’**	129.7’ d	7.32 d (7.8)
**6’’**	127.7 d	7.8+ d (7.7)
**7’’**	21.6 q	2.44 s

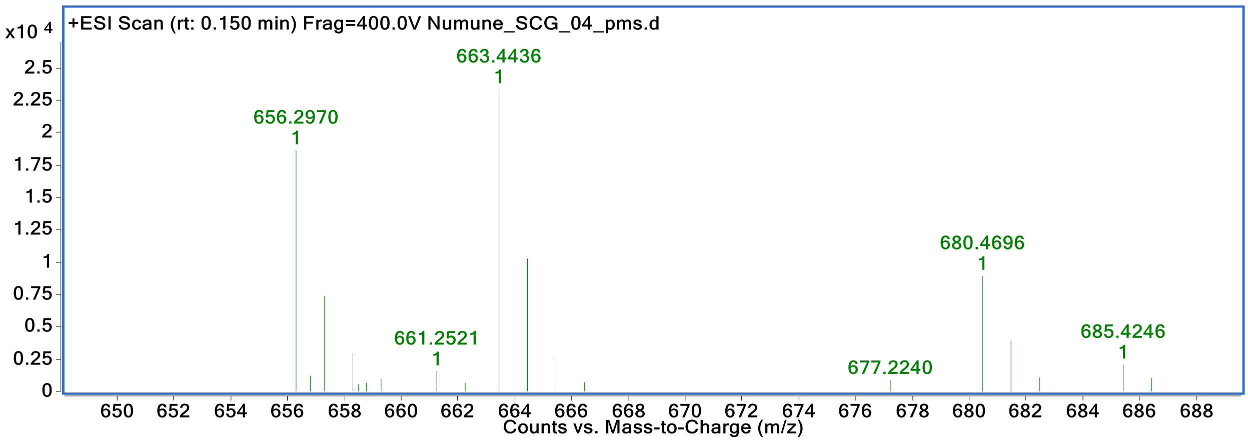

Spectrum 96. HR-ESI-MS Spectrum of SCG-04 (positive mode).

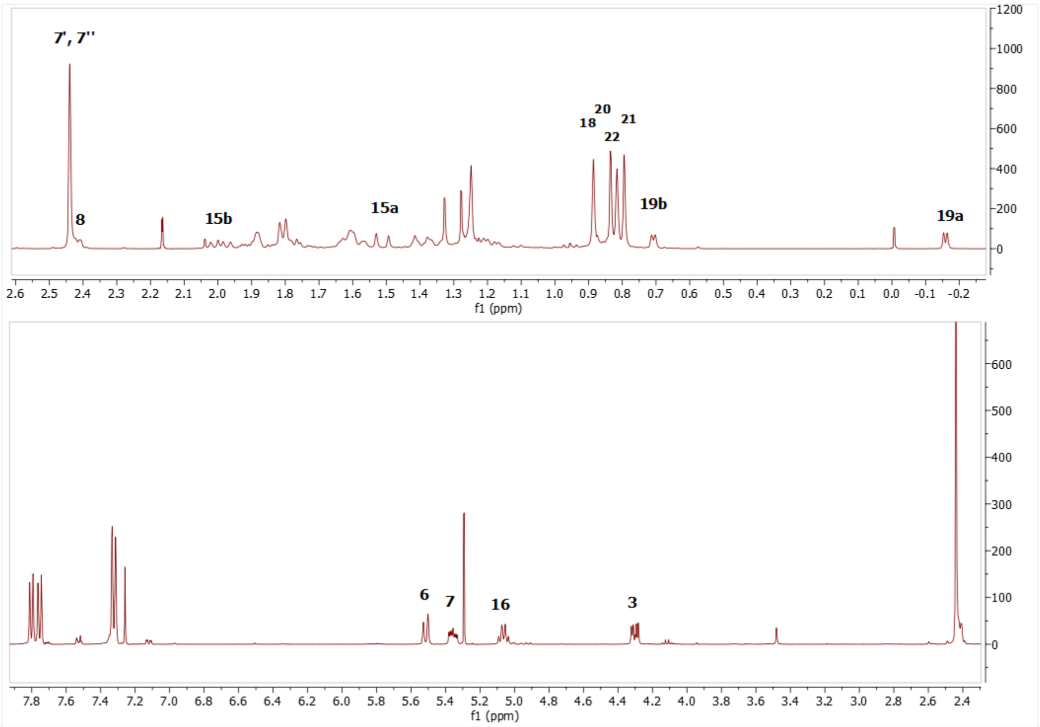

Spectrum 97. ^{1}H NMR Spectrum of SCG-04.

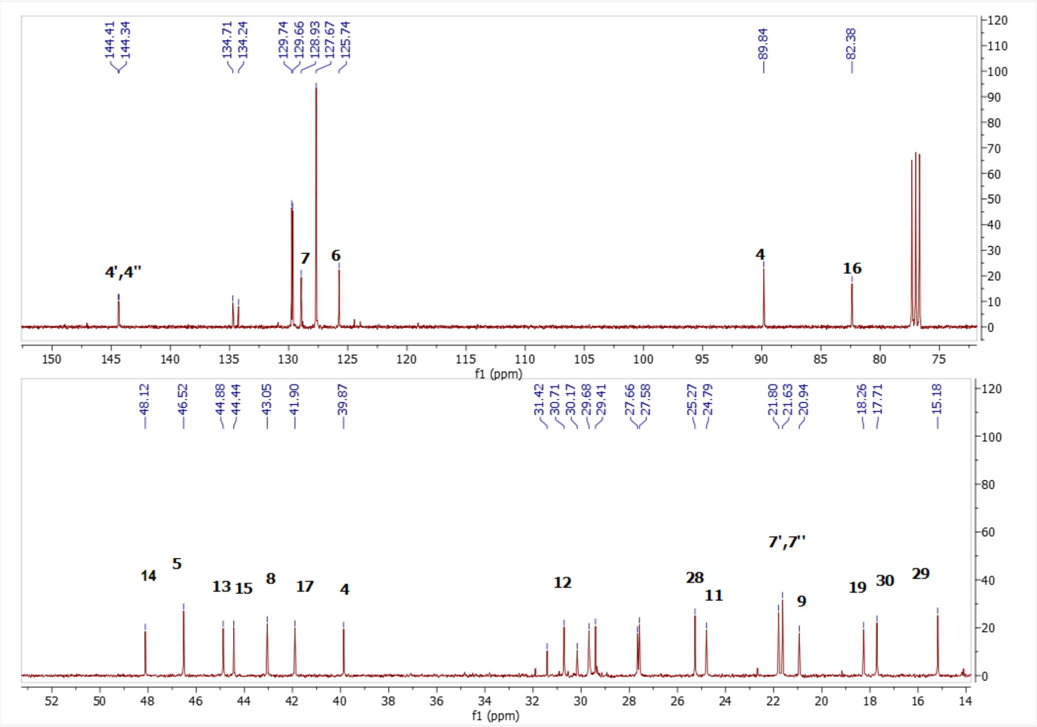

Spectrum 98. ^{13}C NMR Spectrum of SCG-04.

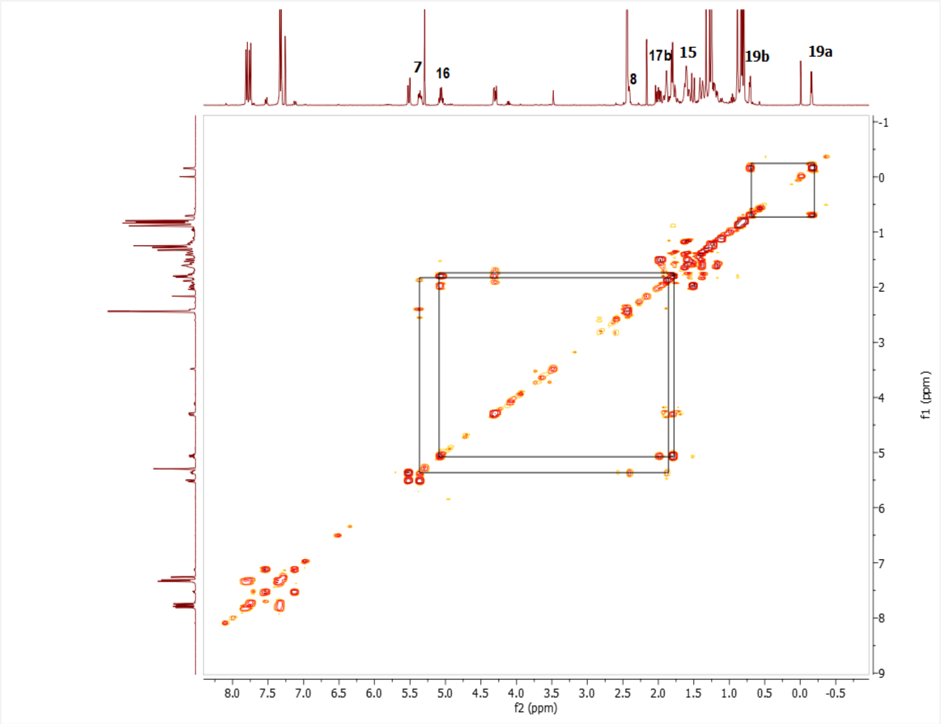

Spectrum 99. COSY spectrum of SCG-04.

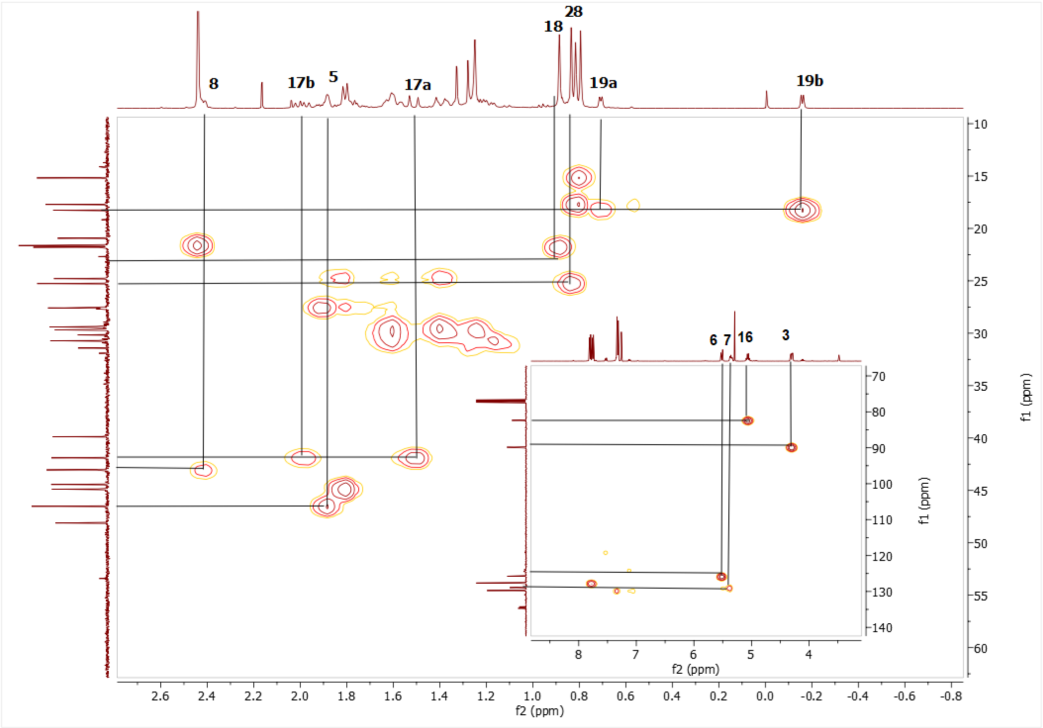

Spectrum 100. HMQC spectrum of SCG-04.

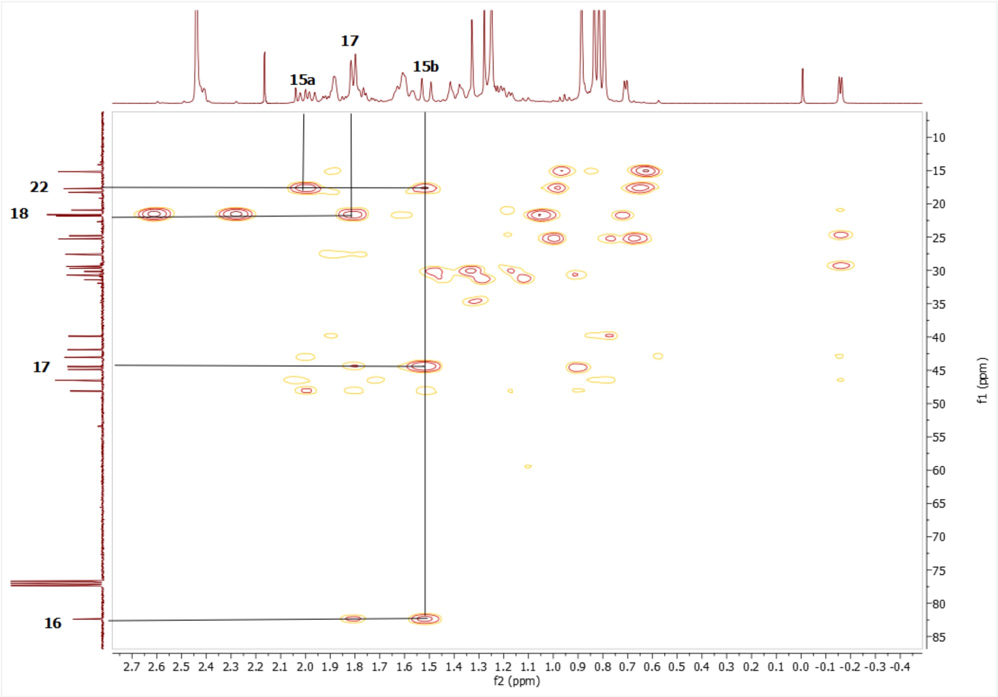

Spectrum 1013. HMBC spectrum of SCG-04.

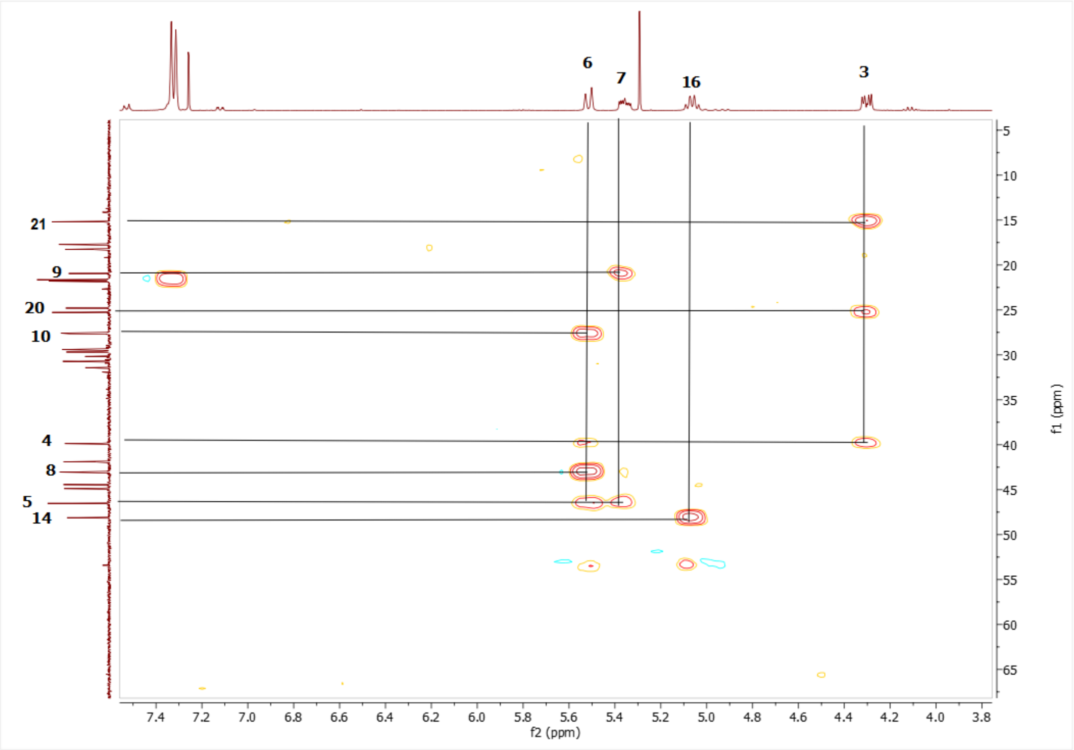

Spectrum 1024. HMBC spectrum of SCG-04.

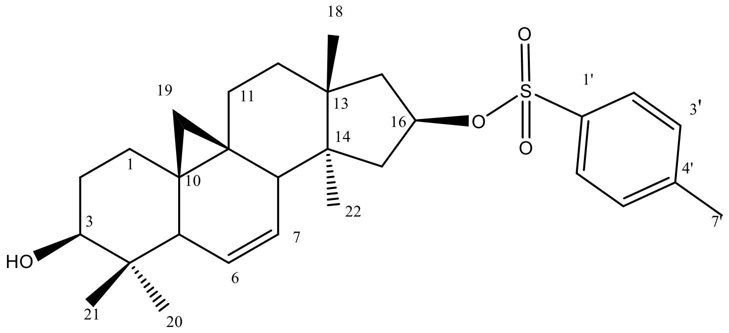

Supplementary Figure 18. Chemical Structure of SCG-05

Supplementary Table 16. The ^{13}C and ^{1}H NMR data of SCG-05 (100/500 MHz, δ ppm, in CDCl3).

**H/C**	**δ_C (ppm)**	**δ_H (ppm), *J* (Hz)**
**1**	29.8 t	1.23 s, 1.43 s
**2**	30.1 t	1.62 m, 1.82 s
**3**	78.5 d	3.33 dd (10.8, 3.9)
**4**	40.4 s	-
**5**	46.5 d	1.86 d (2.4)
**6**	126.9 d	5.61 d (10.5)
**7**	128.5 d	5.37 m
**8**	43.3 d	2.44 m
**9**	21.1 s	-
**10**	28.3 s	-
**11**	25.0 t	1.81 d (5.6), 1.42 m
**12**	31.0 t	1.63 d (12.9), 1.2 dd (12.7, 4.3)
**13**	48.4 s	-
**14**	44.6 s	-
**15**	45.2 t	1.83 s
**16**	82.7	5.07 dd (14.7, 7.2)
**17**	42.1 t	1.53 d (14.7), 2.01 dt (23.2, 11.6)
**18**	21.8 q	0.9 s
**19**	18.5 t	0.72d (3.1), -0.15d (3.9)
**28**	25.6 q	1.05 s
**29**	14.5 q	0.77 s
**30**	17.9 q	0.85 s
**1'**	134.5 s	-
**2'**	127.8 d	7.76 d (8.1)
**3'**	129.9 d	7.32 d (7.9)
**4'**	127.8 d	7.76 d (8.1)
**5'**	129.9 d	7.32 d (7.9)
**6'**	144.5 s	-
**7'**	21.6 q	2.44 s

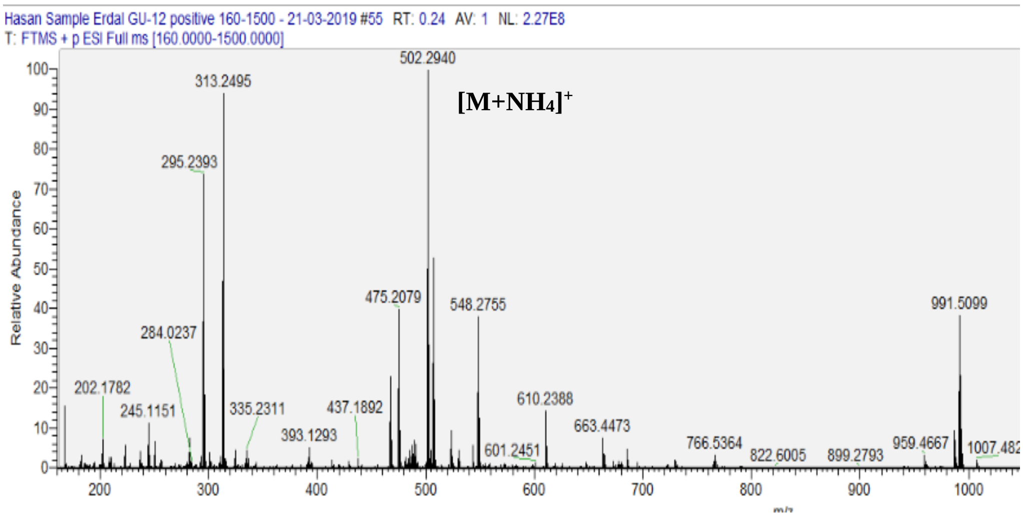

Spectrum 103. HR-ESI-MS Spectrum of SCG-05 (positive mode).

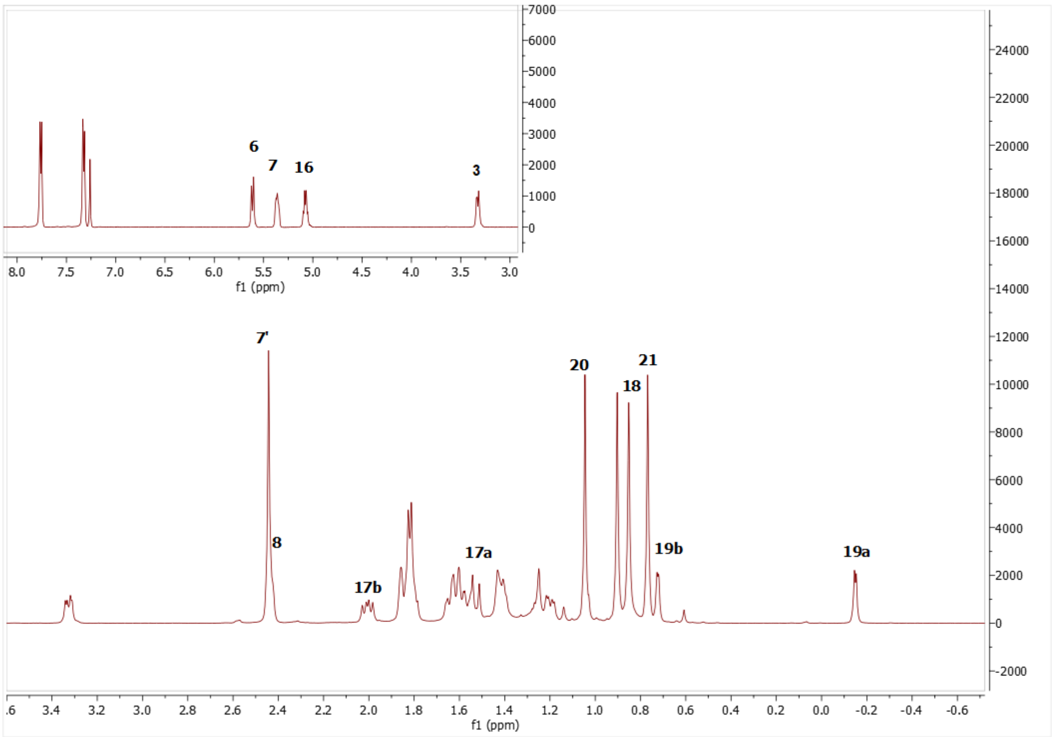

Spectrum 104. ^{1}H NMR Spectrum of SCG-05.

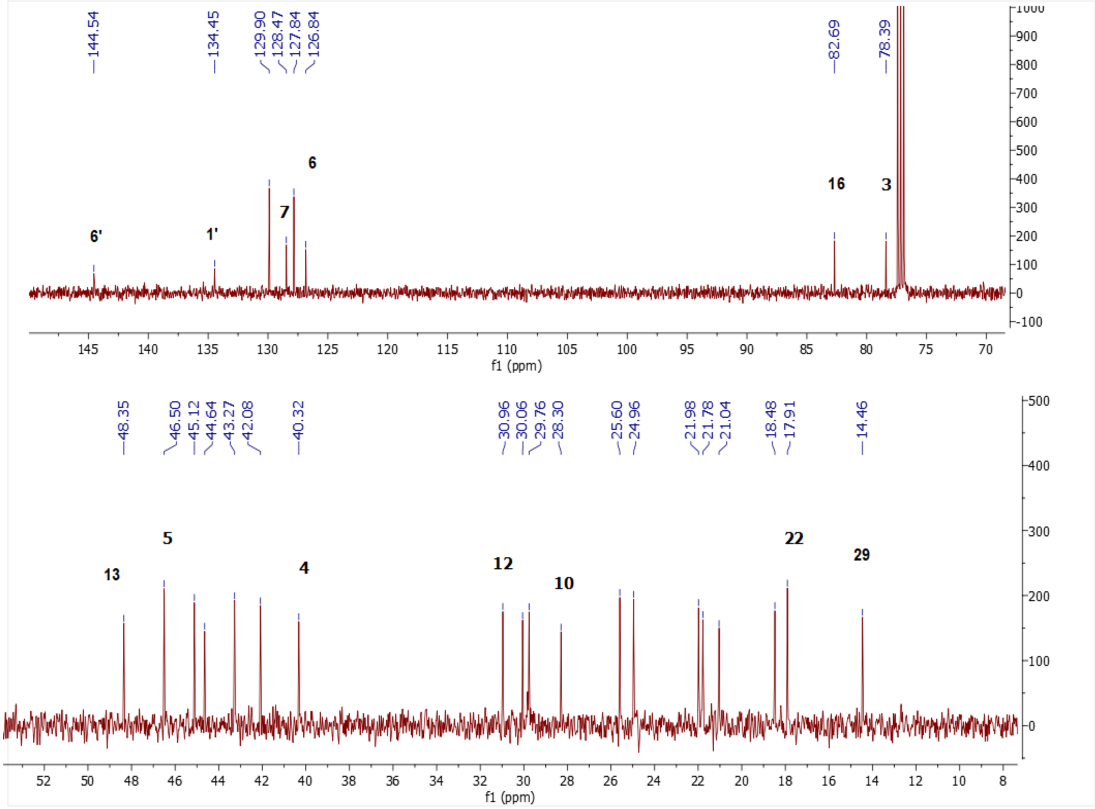

Spectrum 105. ^{13}C NMR Spectrum of SCG-05.

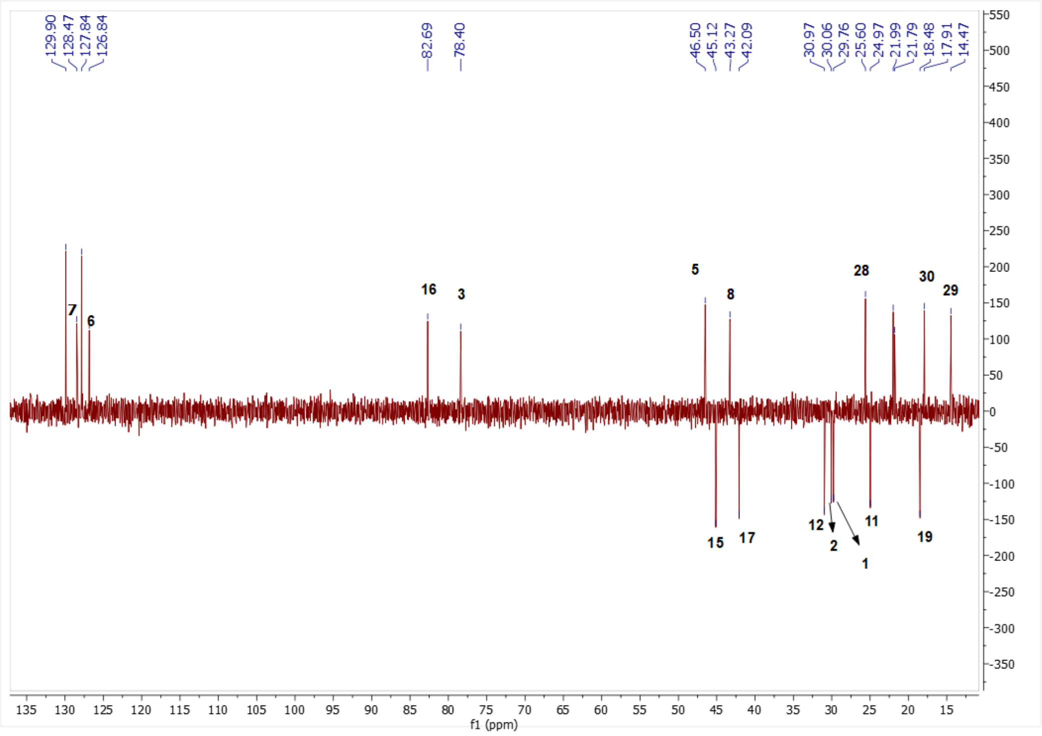

Spectrum 106. DEPT135 spectrum of SCG-05.

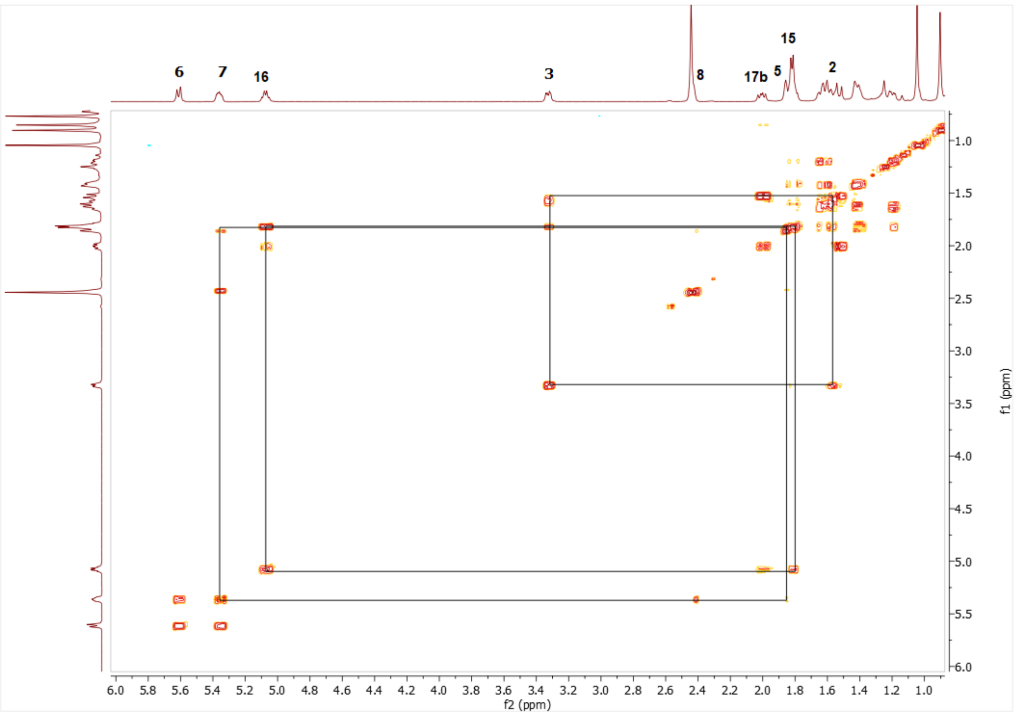

Spectrum 1075. COSY spectrum of SCG-05.

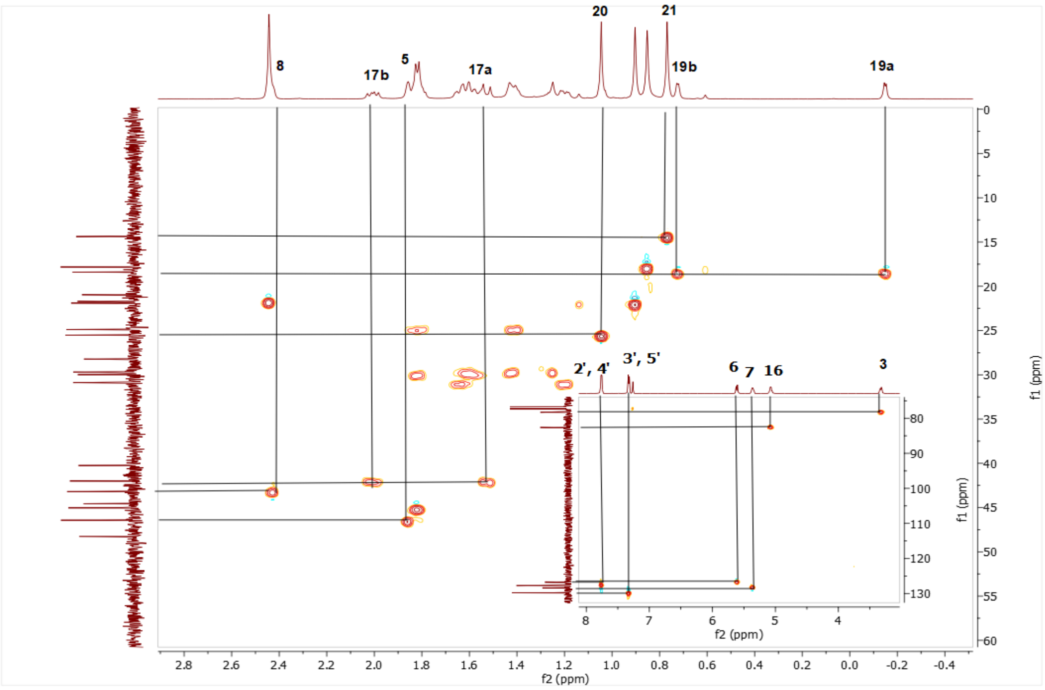

Spectrum 1086. HMQC spectrum of SCG-05.

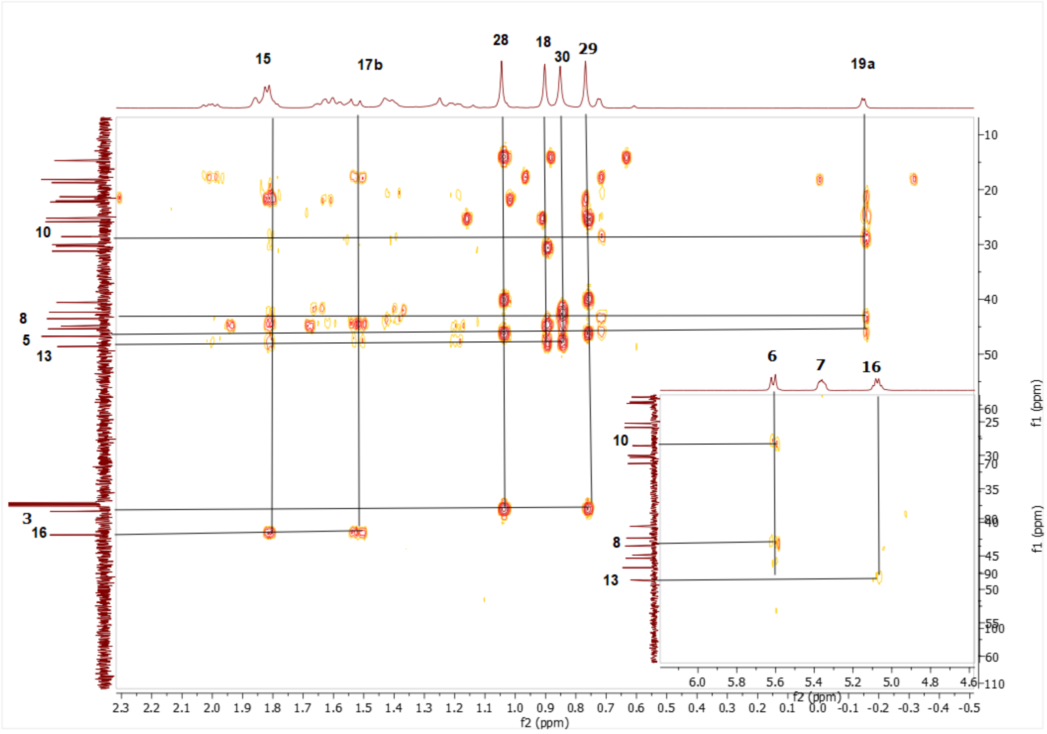

Spectrum 109. HMBC spectrum of SCG-05.

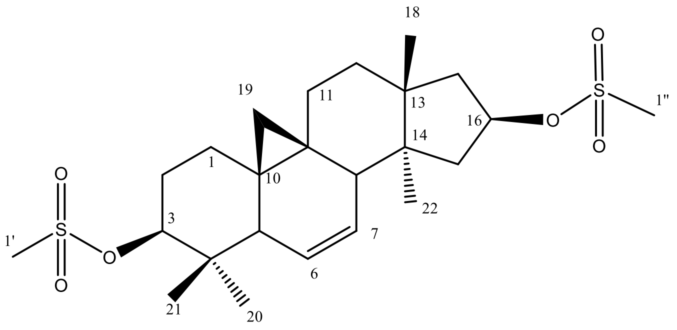

Supplementary Figure 19. Chemical Structure of SCG-06

Supplementary Table 17. The ^{13}C and ^{1}H NMR data of SCG-06 (100/500 MHz, δ ppm, in CDCl3).

**H/C**	**δ_C (ppm)**	**δ_H (ppm), *J* (Hz)**
**1**	29.5 t	1.7 m, 1.92 m
**2**	28.1 t	1.92 m, 2.14 m
**3**	89.5 d	4.45 dd (12, 4.6)
**4**	40.0 s	-
**5**	46.7 d	2.0 m
**6**	125.9 d	5.61 d (10.5)
**7**	129.2 d	5.45 ddd (10.3, 6.1, 3.0)
**8**	43.2 d	2.51 dd (6, 2.6)
**9**	21.2 s	-
**10**	27. 8 s	-
**11**	25.0 t	1.43 m, 1.89 m
**12**	30.9 t	1.27 dd (13, 5.1), 1.69 m
**13**	44.7 s	-
**14**	48.4 s	-
**15**	42.3 t	1.63 m, 2.2 m
**16**	82.0 d	5.29 q (7.6)
**17**	45.2 t	1.93 m, 2.04 m
**18**	22.0 q	0.98 s
**19**	18.4 t	-0.07 d (4.3), 0.77 d (4.3)
**28**	15.3 q	0.86 s
**29**	25.8 q	1.1 s
**30**	17.9 q	0.87 s
**1’**	39.0 q*	3.03 s’
**1’’**	38.5 q*	2.97 s’

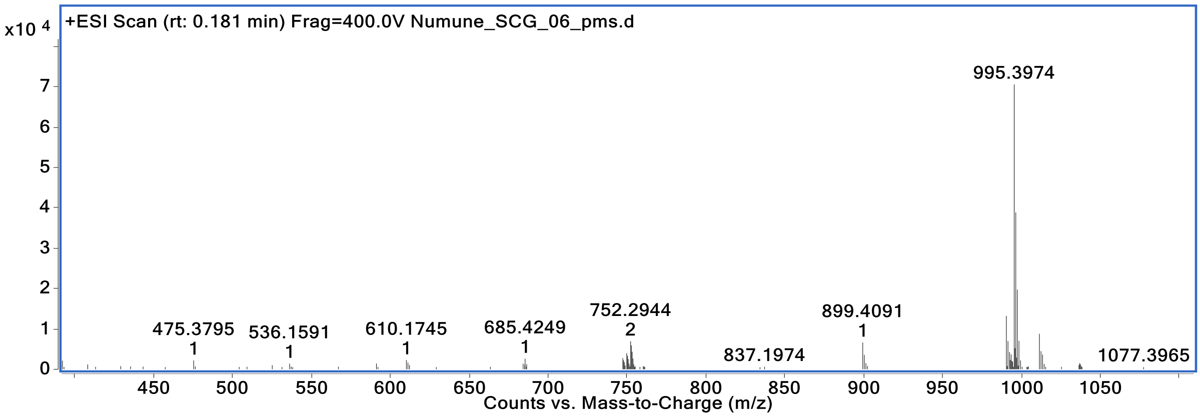

Spectrum 110. HR-ESI-MS Spectrum of SCG-06 (positive mode).

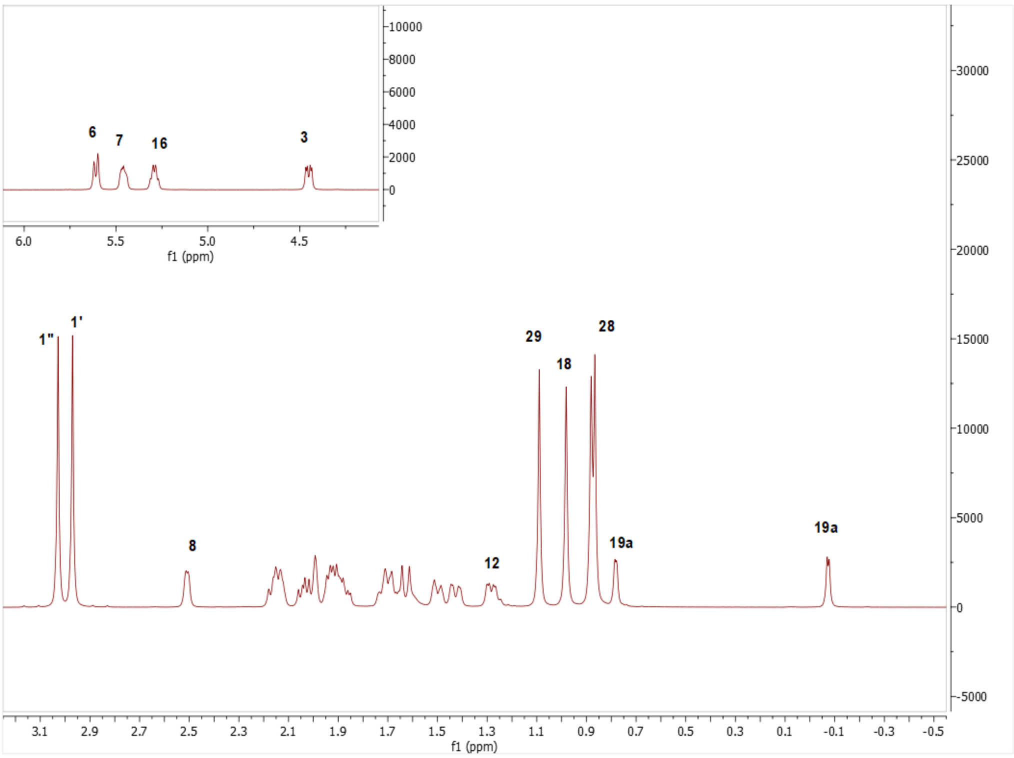

Spectrum 111. ^{1}H NMR Spectrum of SCG-06.

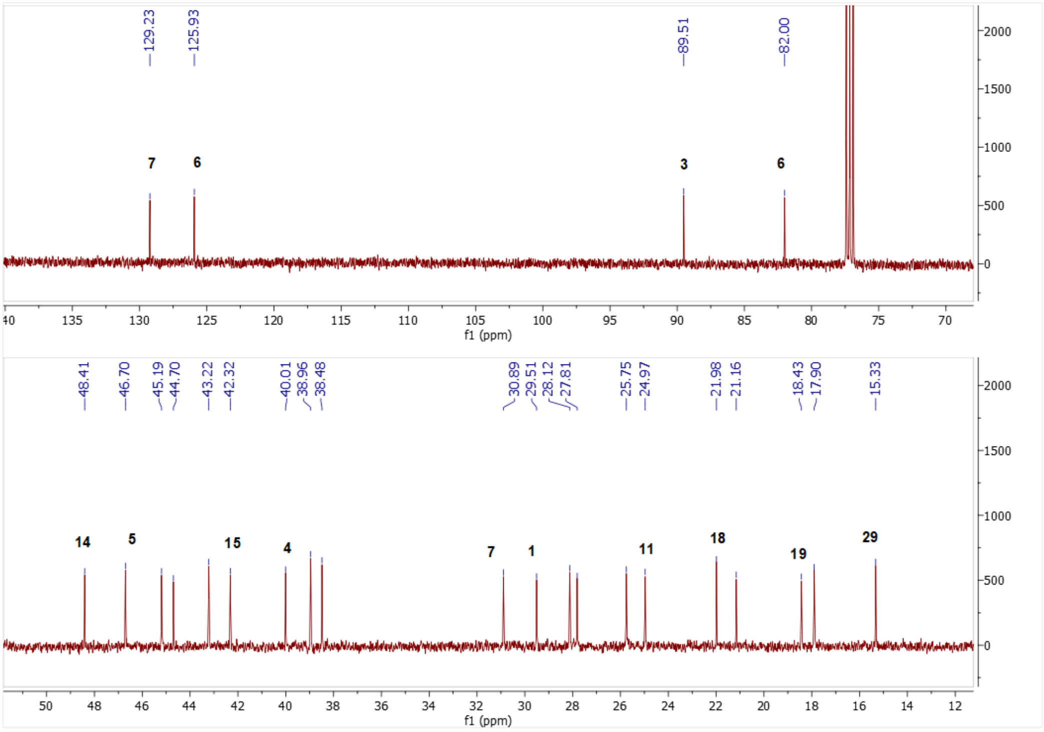

Spectrum 1127. ^{13}C NMR Spectrum of SCG-06.

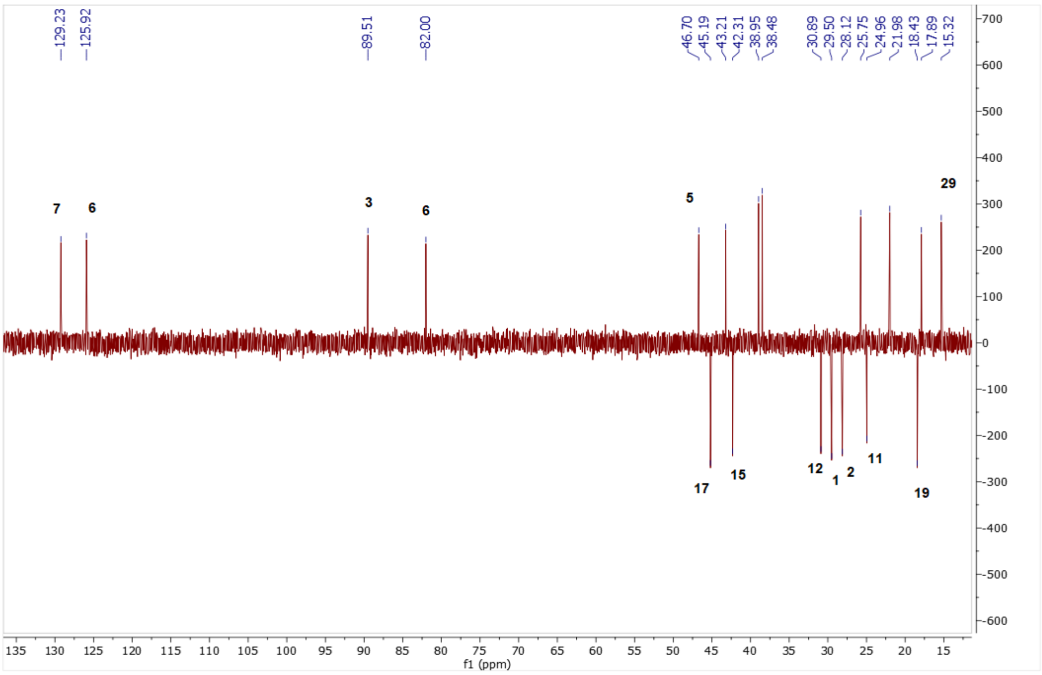

Spectrum 1138. DEPT135 spectrum of SCG-06.

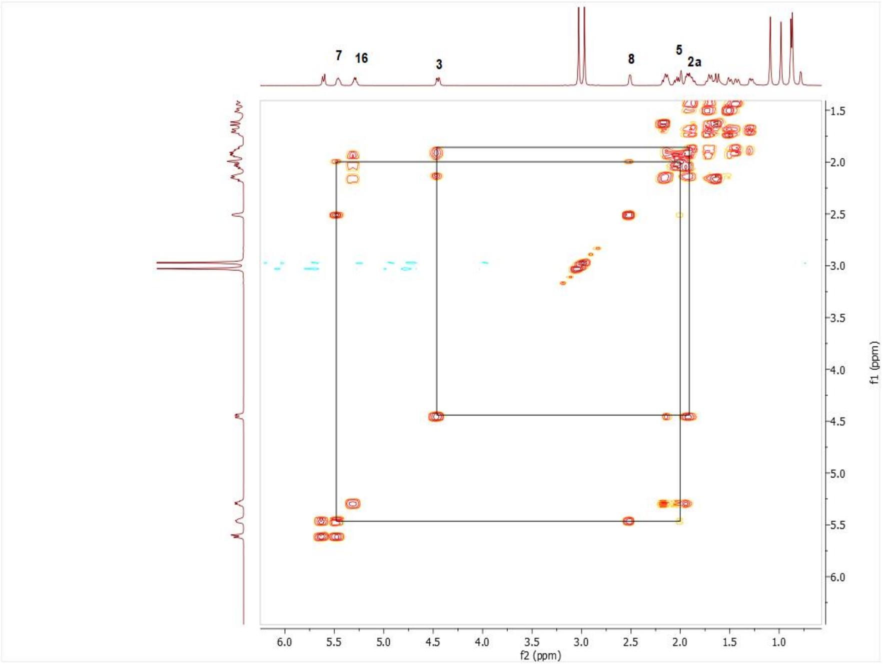

Spectrum 114. COSY spectrum of SCG-06.

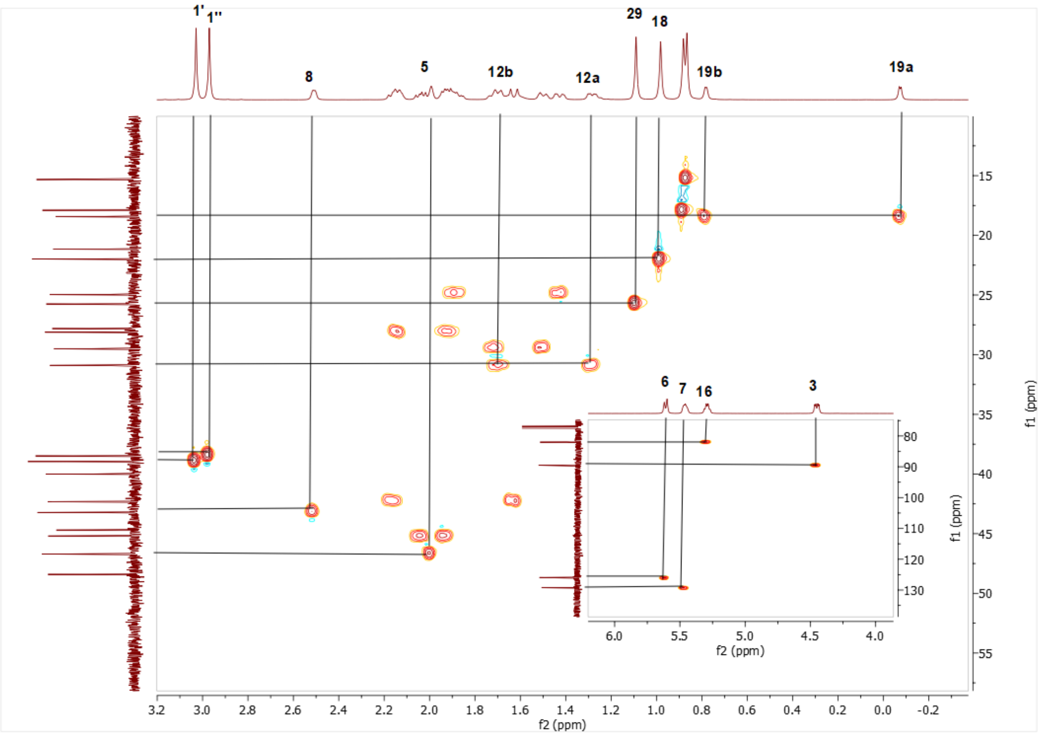

Spectrum 115. HMQC spectrum of SCG-06.

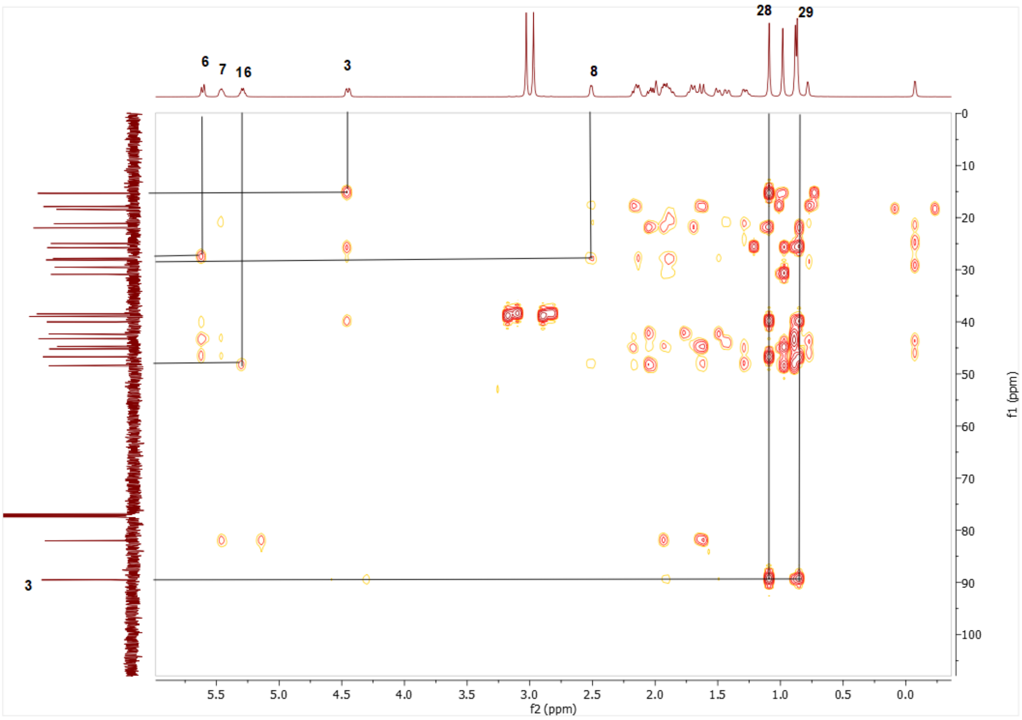

Spectrum 116. HMBC spectrum of SCG-06.

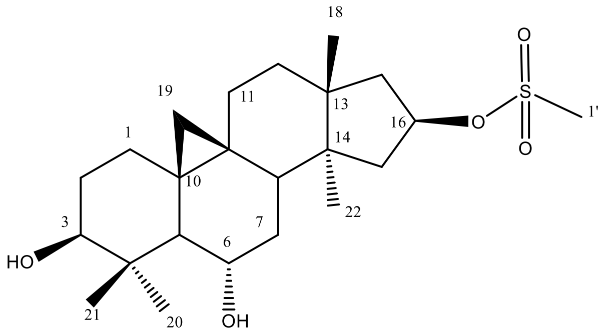

Supplementary Figure 20. Chemical Structure of SCG-07

Supplementary Table 18. The ^{13}C and ^{1}H NMR data of SCG-07 (100/500 MHz, δ ppm, in CDCl3).

H/C	δ_C (ppm)	δ_H (ppm), *J* (Hz)
**1**	31.3 t	1.26 m, 1.63 m
**2**	30.2 t	1.58 m, 1.8 m
**3**	78.48 d	3.22 dd (11.2, 4.2)
**4**	41.6 s	-
**5**	53.4 d	1.37 m
**6**	68.4 d	3.56 ddd (9.2, 9.2, 4.1)
**7**	37.6 t	1.38 m, 1.55 m
**8**	45.6 d	1.71 m
**9**	20.7 s	-
**10**	29.0 s	-
**11**	26.1 t	1.31 m, 1.97 m
**12**	30.3 t	1.48 m
**13**	44.7 s	-
**14**	46.0 s	-
**15**	44.6 t	1.79 m, 2.06 m
**16**	82.39 d	5.26 q (8)
**17**	46.2 t	2.01 m, 2.11 m
**18**	24.9 q	1.03 s
**19**	26.7 t	0.31 d, 0.5 d(4.7)
**28**	15.3 q	0.94 s
**29**	27.9 q	1.23 s
**30**	19.8 q	1.1 s
**1'**	38.5 q	2.97 s

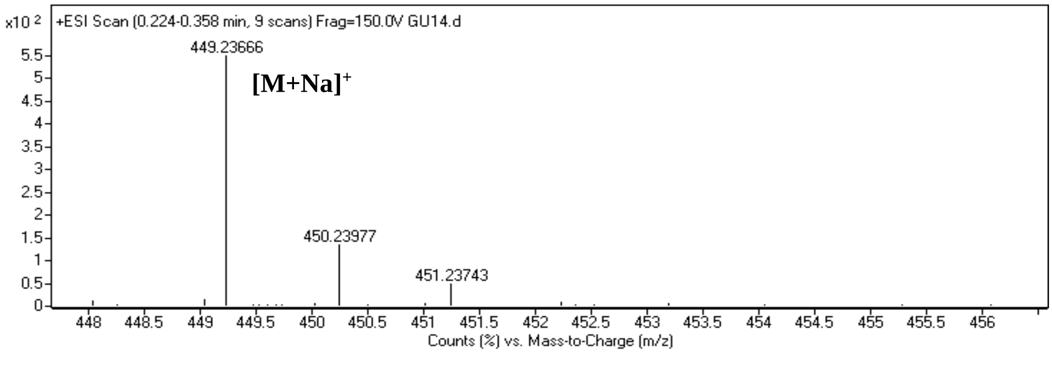

Spectrum 117. HR-ESI-MS Spectrum of SCG-07 (positive mode).

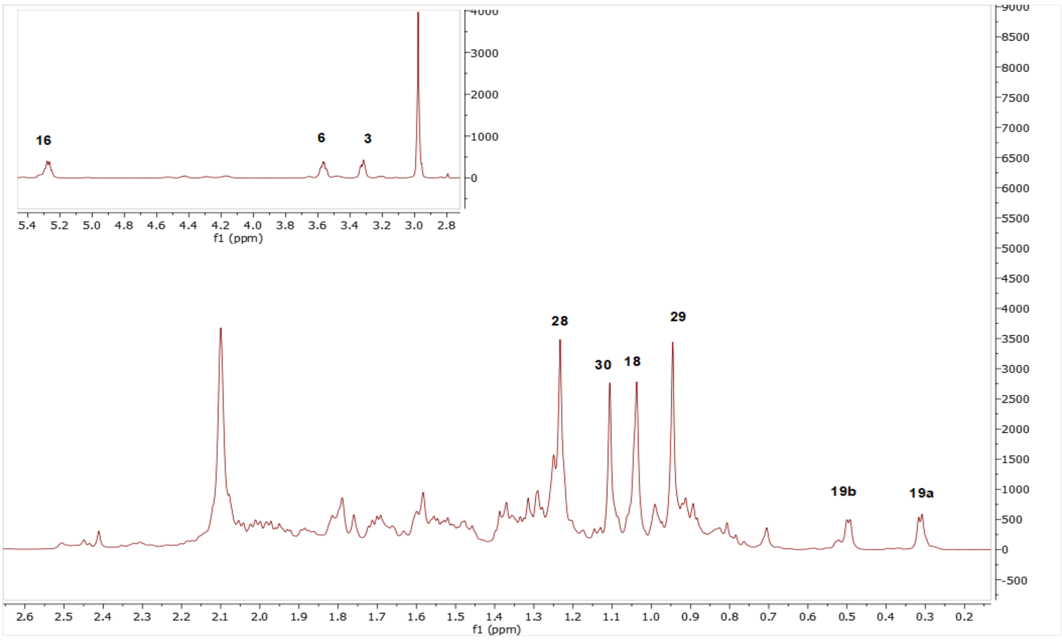

Spectrum 118. ^{1}H NMR Spectrum of SCG-07.

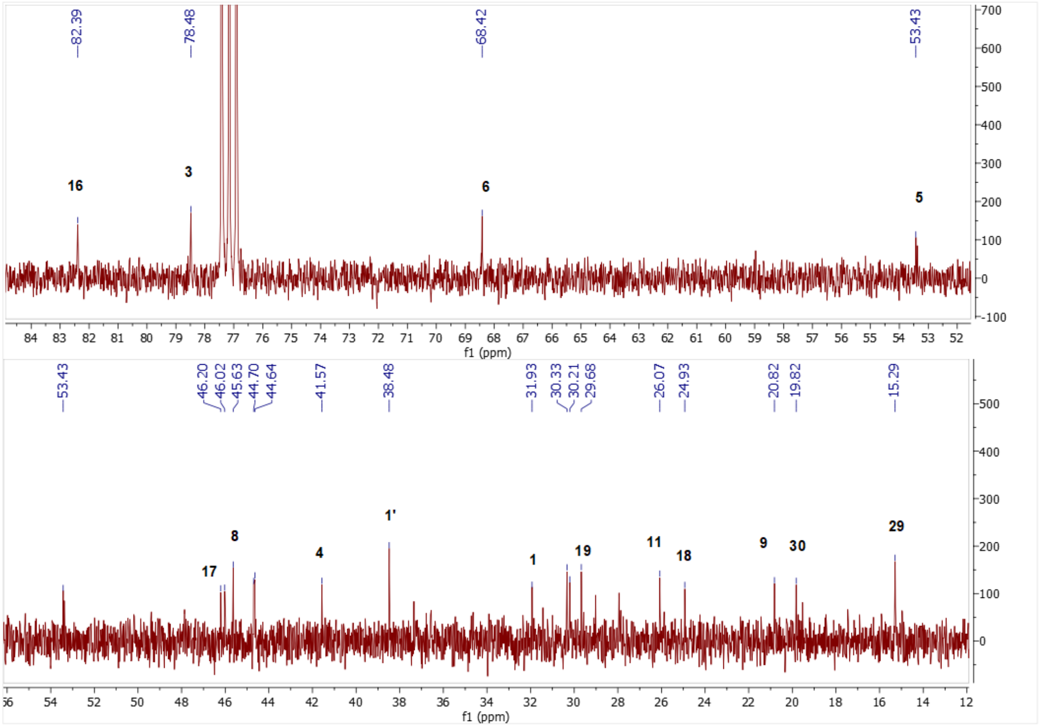

Spectrum 119. ^{13}C NMR Spectrum of SCG-07.

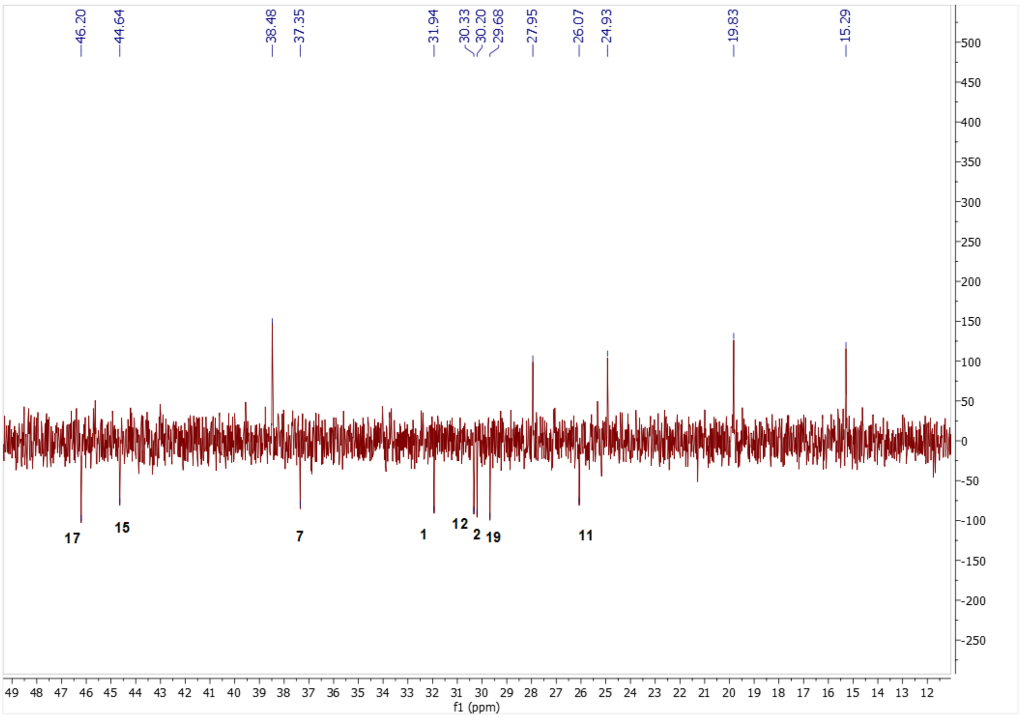

Spectrum 120. DEPT135 spectrum of SCG-07.

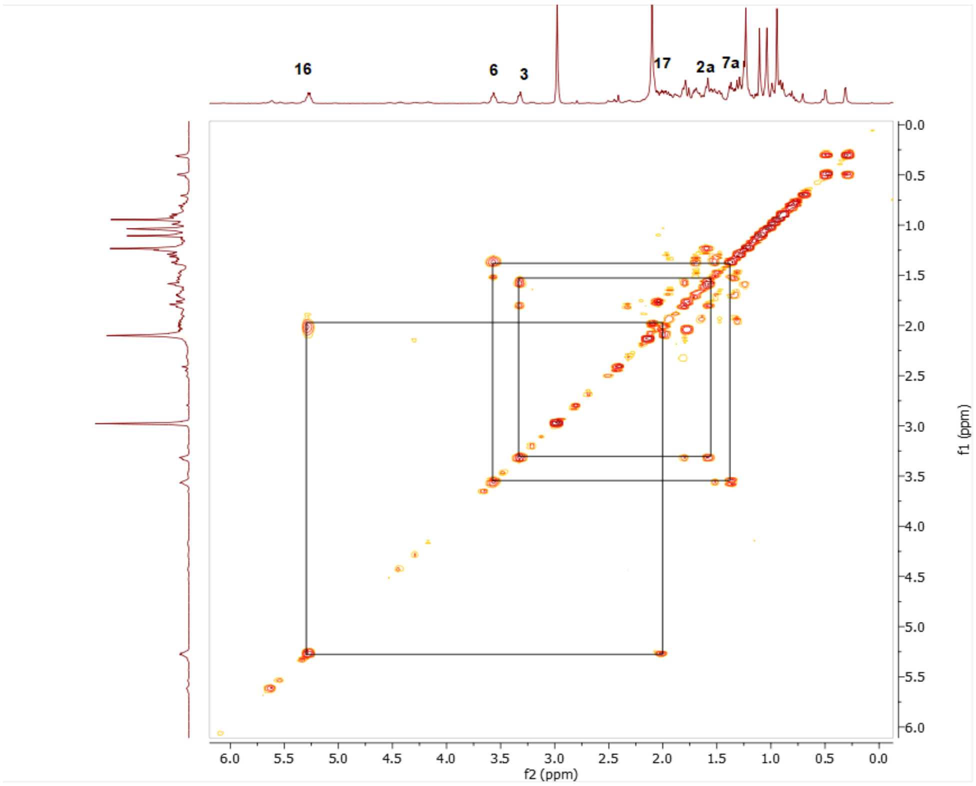

Spectrum 121. COSY spectrum of SCG-07.

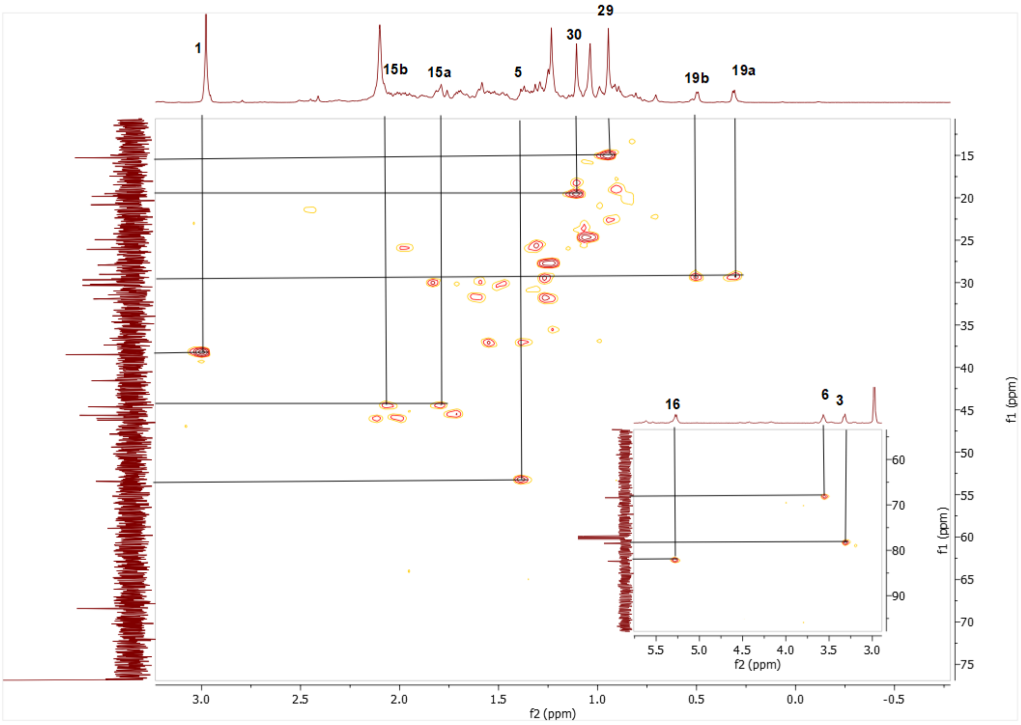

Spectrum 122. HMQC spectrum of SCG-07.

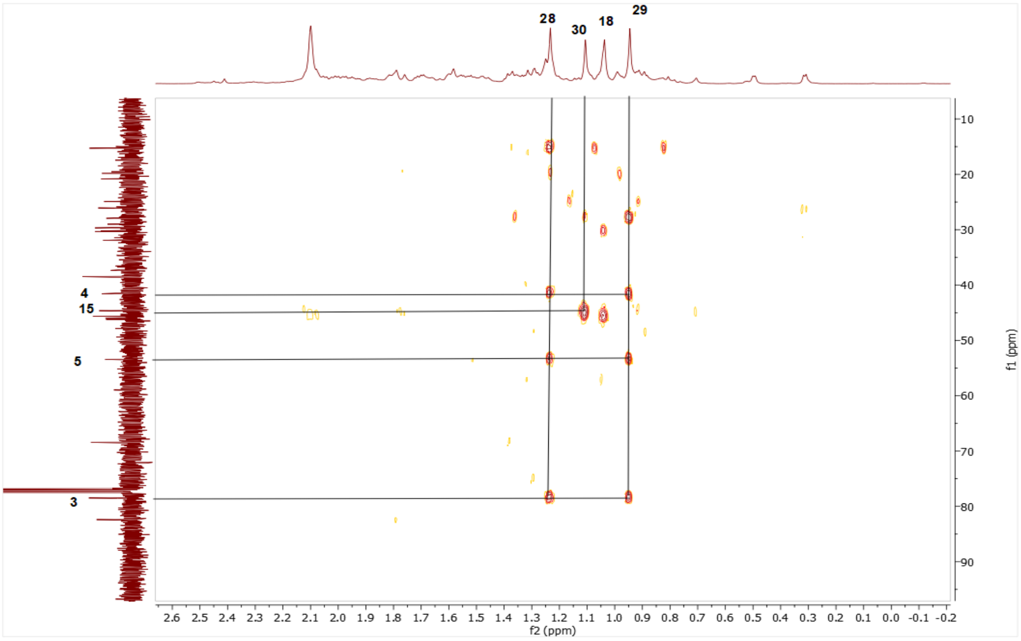

Spectrum 123. HMBC spectrum of SCG-07.

A

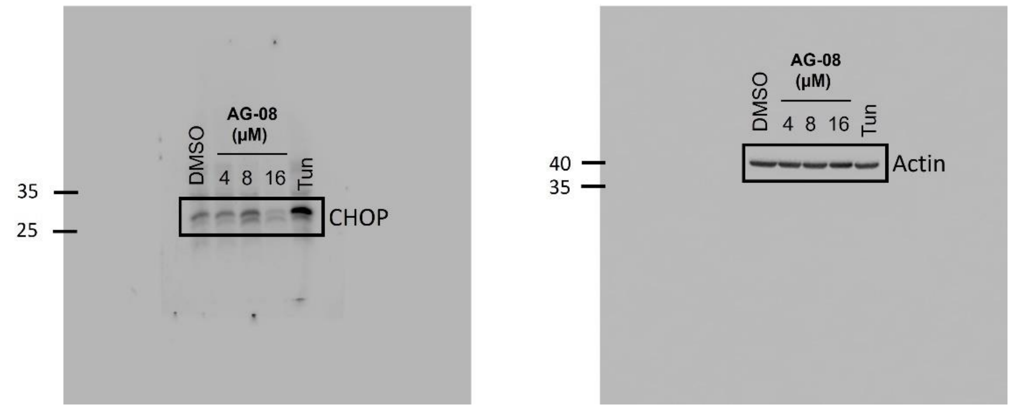
DMSO
AG-08
(μM)
4 8 16
Tun
35
25
CHOP
40
35
Actin

B

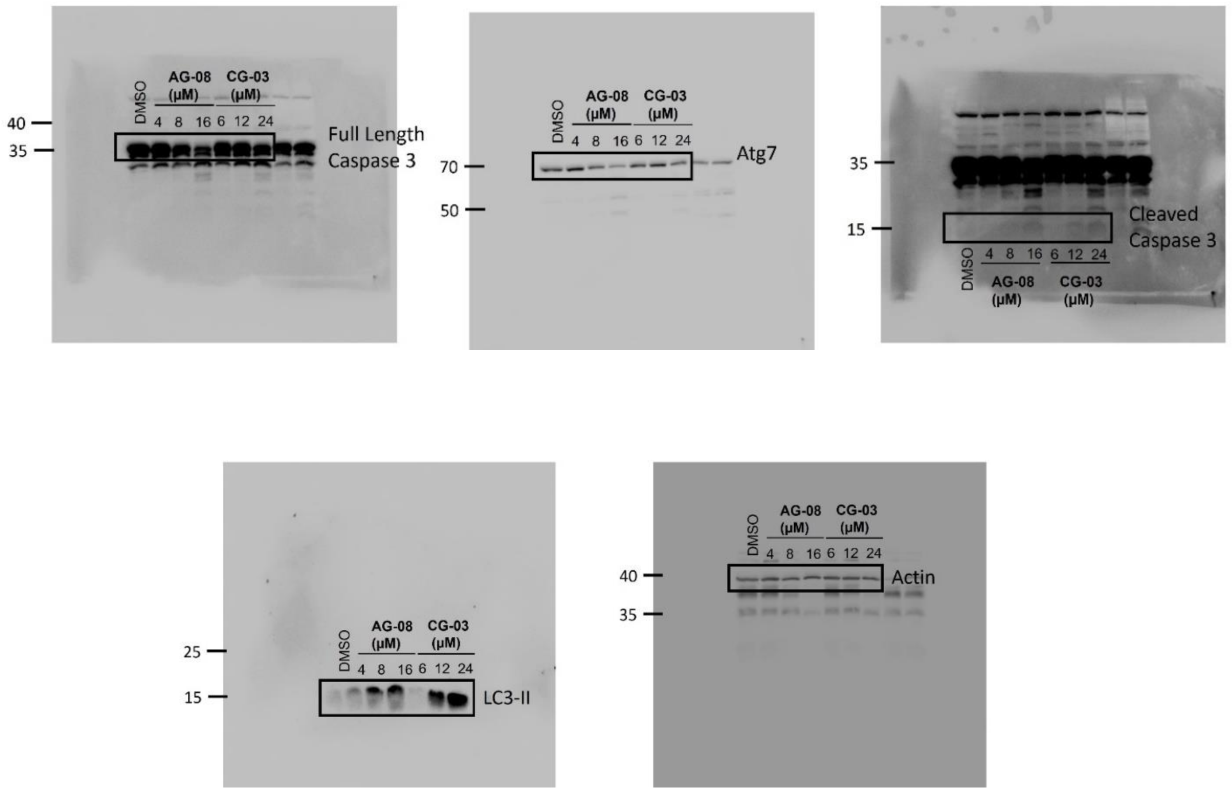
DMSO
AG-08
(μM)
CG-03
(μM)
4 8 16 6 12 24
40
35
Full Length
Caspase 3
70
50
Atg7
35
15
Cleaved
Caspase 3
25
15
LC3-II
40
35
Actin

C

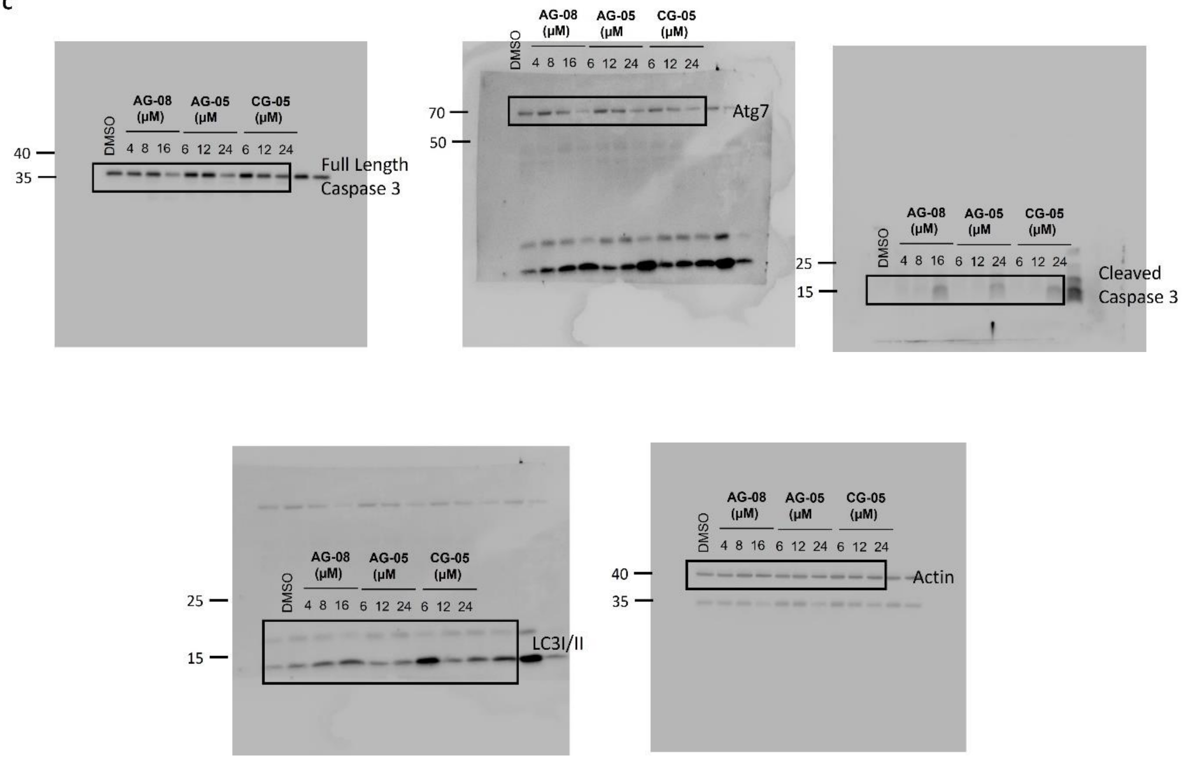

D

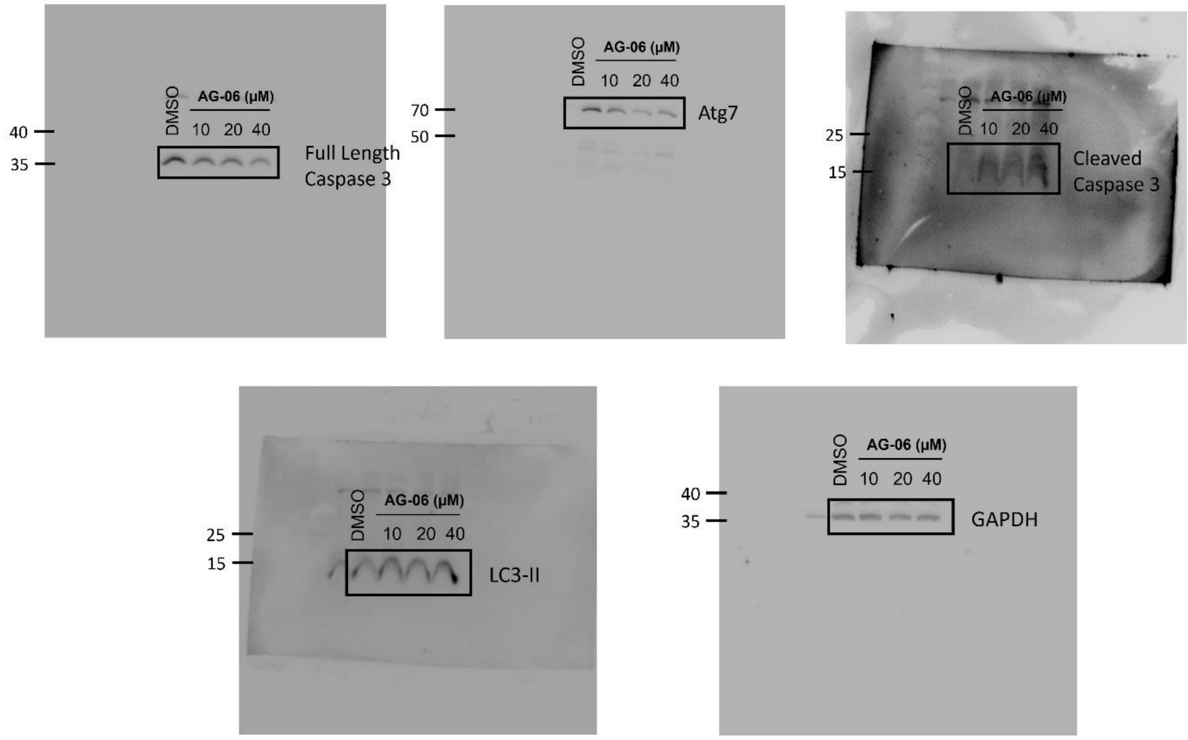

Supplementary Figure 4. Display of original blots. Images for (A) Figure 3E, (B) Supplementary Figure 2A, (C) Supplementary Figure 2C, (D) Supplementary Figure 2D.
